# Structurally constrained phosphonate internucleotide linkage impacts oligonucleotide-enzyme interaction, and modulates siRNA activity and allele specificity

**DOI:** 10.1093/nar/gkab1126

**Published:** 2021-11-25

**Authors:** Ken Yamada, Samuel Hildebrand, Sarah M Davis, Rachael Miller, Faith Conroy, Ellen Sapp, Jillian Caiazzi, Julia F Alterman, Loic Roux, Dimas Echeverria, Matthew R Hassler, Edith L Pfister, Marian DiFiglia, Neil Aronin, Anastasia Khvorova

**Affiliations:** RNA Therapeutics Institute, University of Massachusetts Medical School, 368 Plantation Street, Worcester, MA 01605, USA; RNA Therapeutics Institute, University of Massachusetts Medical School, 368 Plantation Street, Worcester, MA 01605, USA; RNA Therapeutics Institute, University of Massachusetts Medical School, 368 Plantation Street, Worcester, MA 01605, USA; RNA Therapeutics Institute, University of Massachusetts Medical School, 368 Plantation Street, Worcester, MA 01605, USA; Department of Medicine, University of Massachusetts Medical School, Worcester, MA, USA; RNA Therapeutics Institute, University of Massachusetts Medical School, 368 Plantation Street, Worcester, MA 01605, USA; Department of Medicine, University of Massachusetts Medical School, Worcester, MA, USA; Department of Neurology, Harvard Medical School and MassGeneral Institute for Neurodegenerative Disease, Charlestown, MA, USA; RNA Therapeutics Institute, University of Massachusetts Medical School, 368 Plantation Street, Worcester, MA 01605, USA; RNA Therapeutics Institute, University of Massachusetts Medical School, 368 Plantation Street, Worcester, MA 01605, USA; RNA Therapeutics Institute, University of Massachusetts Medical School, 368 Plantation Street, Worcester, MA 01605, USA; RNA Therapeutics Institute, University of Massachusetts Medical School, 368 Plantation Street, Worcester, MA 01605, USA; RNA Therapeutics Institute, University of Massachusetts Medical School, 368 Plantation Street, Worcester, MA 01605, USA; Department of Medicine, University of Massachusetts Medical School, Worcester, MA, USA; Department of Neurology, Harvard Medical School and MassGeneral Institute for Neurodegenerative Disease, Charlestown, MA, USA; RNA Therapeutics Institute, University of Massachusetts Medical School, 368 Plantation Street, Worcester, MA 01605, USA; Department of Medicine, University of Massachusetts Medical School, Worcester, MA, USA; RNA Therapeutics Institute, University of Massachusetts Medical School, 368 Plantation Street, Worcester, MA 01605, USA; Program in Molecular Medicine, University of Massachusetts Medical School, Worcester, MA, USA

## Abstract

Oligonucleotides is an emerging class of chemically-distinct therapeutic modalities, where extensive chemical modifications are fundamental for their clinical applications. Inter-nucleotide backbones are critical to the behaviour of therapeutic oligonucleotides, but clinically explored backbone analogues are, effectively, limited to phosphorothioates. Here, we describe the synthesis and bio-functional characterization of an internucleotide (*E*)-vinylphosphonate (^i^*E*-VP) backbone, where bridging oxygen is substituted with carbon in a locked stereo-conformation. After optimizing synthetic pathways for ^i^*E*-VP-linked dimer phosphoramidites in different sugar contexts, we systematically evaluated the impact of the ^i^*E*-VP backbone on oligonucleotide interactions with a variety of cellular proteins. Furthermore, we systematically evaluated the impact of ^i^*E*-VP on RNA-Induced Silencing Complex (RISC) activity, where backbone stereo-constraining has profound position-specific effects. Using Huntingtin (HTT*)* gene causative of Huntington's disease as an example, ^i^*E*-VP at position 6 significantly enhanced the single mismatch discrimination ability of the RISC without negative impact on silencing of targeting wild type *htt* gene. These findings suggest that the ^i^*E*-VP backbone can be used to modulate the activity and specificity of RISC. Our study provides (i) a new chemical tool to alter oligonucleotide-enzyme interactions and metabolic stability, (ii) insight into RISC dynamics and (iii) a new strategy for highly selective SNP-discriminating siRNAs.

## Graphical Abstract

**Graphical Abstract:**
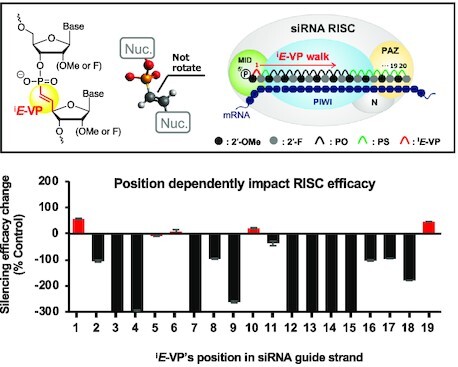
Structurally constrained phosphonate internucleotide linkage impacts oligonucleotide-enzyme interaction, and modulates siRNA activity and allele specificity.

## INTRODUCTION

Oligonucleotide therapeutics, such as small interfering RNAs (siRNAs), are a promising class of human therapeutics that directly and specifically modulate the expression of disease-causing messenger RNA (mRNA) (1–4). Several oligonucleotide drugs have been FDA-approved, and multiple compounds are in the late stages of clinical development (3,4). The clinical efficacy and *in vivo* stability of these drugs depends on chemical modifications to the nucleotide backbone (i.e. phosphodiester linkages and sugars), which is the major driving force behind the formation of specific nucleic acid-protein complexes (1,2).

Various chemically-modified phosphate backbones, including peptide nucleic acid and morpholino oligomers for splice-switching oligonucleotides, have been developed to modulate the functionality of oligonucleotides for biological and clinical applications (1–4). However, phosphorothioate (PS) is currently the only inter-nucleotide backbone modification applied to siRNAs and RNaseH-activating antisense oligonucleotides (ASOs) in the clinic (5–10). Unlike the other backbone variants, PS modifications have minimal structural impact and thus allow oligonucleotides to maintain efficient interactions with their effector proteins—e.g., RNA-induced Silencing Complex (RISC) and RNaseH (5). Although the widely-used PS modification contributes to metabolic stability and bio-distribution properties of siRNAs and ASOs *in vivo*, it can cause toxicity dependent on sequence, position and combination of 2′-substituents, potentially limiting the oligonucleotide therapeutic index (5,11–15). To reduce the chance of PS-related toxicity one of options could be reducing the numbers of PS where PS is converted to canonical phosphodiester backbone compromising metabolic stability. Thus, developing alternative, metabolically-stable phosphodiester bond analogues with bio-compatible configurations would be expected to offer therapeutic advances to the oligonucleotides.

One chemical option for a close-to-natural backbone modification is vinylphosphonate, or (*E*)-VP, a phosphate mimic that replaces the oxygen-phosphorus bond of the phosphodiester linkage with a carbon-phosphorus bond (16). While (*E*)-VP is not likely to alter the charge or molecular size of the backbone, this modification does restrict the torsion angle to 180^o^, which may have an impact on protein recognition to provide the desired metabolic impact (17–20). Modifications that induce structural constraint exerts beneficial effects when applied to the sugars of oligonucleotides. For instance, 2′-fluoro and LNA (locked nucleic acid) sugar modifications can increase nuclease stability and target binding affinity while maintaining compatibility with oligonucleotide effector proteins (7). Synthesis and bio-chemical properties of internucleotide (*E*)-VP backbone (^i^*E*-VP) in a context of single and double stranded DNA oligonucleotides were thoroughly investigated by Hayes, *et al.* where it was found that the backbone structural constraining induces inhibition of DNA helicase activity and DNA-binding nuclease activity (21–25). However, the impact of ^i^*E*-VP backbone on RNA oligonucleotides has not established yet in a context of RNA therapeutics research.

**Figure 1.**
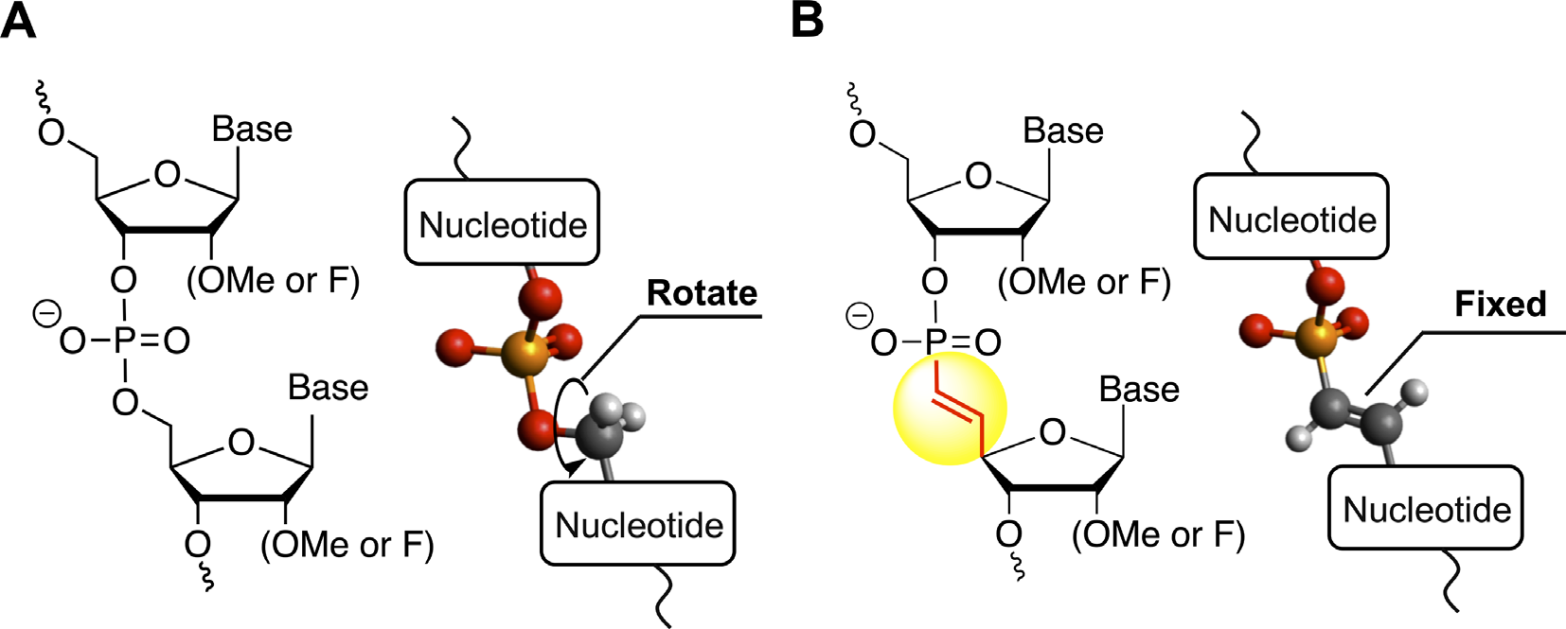
(**A**) Structures of canonical phosphodiester backbone and (**B**) ^i^*E*-VP backbone.

Phosphonate and (*E*)-VP modifications were first explored during the 1980–1990s for the synthesis of different nucleotide-based antiviral agents. Incorporating phosphonate analogues maintained the desired biological interactions, while enhancing metabolic stability (16,26,27). More recently, the impact of a 5′-terminal (*E*)-VP modification was studied in the context of single-stranded and double-stranded RNAs. Introduction of (*E*)-VP on the 5′ end enhanced *in vivo* efficacy of single-stranded, hydrophobically-modified, and GalNAc-conjugated siRNAs (28–35). The enhanced efficacy was attributed to a combination of increased phosphatase resistance and favorable shape-fitting of 5′-**(***E***)**-VP structure in the MID domain of human Ago2 – a protein in RISC (28–35). To date, the biological impact of (*E*)-VP on therapeutic RNA modalities has been evaluated only as a 5′ terminal modification of oligonucleotides or as a monomer, partially because of synthetic limitations. Optimizing synthetic methods to achieve an ‘inter-nucleotide’ (*E*)-VP or (^i^*E*-VP) backbone in the context of therapeutic oligonucleotides will enable investigation into how ^i^*E*-VP-induced constraint of the backbone impacts biological behavior (Figure 1A, B).

Here, we report an optimized synthetic pathway for sufficient production of ^i^*E*-VP-containing dimer phosphoramidites in the context of different sugar modifications. Using dimer phosphoramidite building blocks, we then synthesized panels of ^i^*E*-VP-modified oligonucleotides, and evaluated the impact of ^i^*E*-VP on nuclease and reverse transcriptase activity, and on siRNA efficacy and specificity.

## MATERIALS AND METHODS

### General experimental methods

The NMR spectra were recorded using Brucker 500 MHz spectrometer. ^1^H, ^13^C, ^19^F and ^31^P NMR spectra were recorded at 500 MHz (^1^H-NMR, 500 MHz; ^13^C-NMR, 125 MHz; ^31^P-NMR, 202 MHz). The chemical shifts were measured from tetramethylsilane (0 ppm), CDCl_3_ (7.26 ppm), DMSO-*d*_6_ (2.49 ppm) and CD_3_CN-*d*_3_ (1.93 ppm) for ^1^H-NMR spectra, CDCl_3_ (77.0 ppm), DMSO-*d*_6_ (39.7 ppm) and CD_3_CN-*d*_3_ (1.30 ppm) for ^13^C-NMR spectra, and 85% H_3_PO_4_ for ^31^P-NMR spectra as external standards. High-resolution electrospray ionization mass spectrometry (HR-ESI-MS) analysis for the monomers and dimer nucleos(t)ides were performed on a Thermo Scientific Orbitrap Velos Pro mass spectrometer in the positive ion mode. The mass analysis of oligonucleotides was conducted by LC–MS on an Agilent 6530 accurate-mass Q-TOF LC/MS (Agilent technologies, Santa Clara, CA). Thin-layer chromatography (TLC) analysis was conducted using silica gel-coated aluminium-backed TLC plates (0.20 mm thickness) containing F-254 UV indicator (Silicycle Inc., Canada). Column chromatography was performed with silica column (Flash Column Silica-CS-Agela; 12–330 g; 40–60 μm), using the CombiFlash Rf200 (Teledyne Isco, Inc.) Companion Chromatograph. The synthesis of modified oligonucleotides was performed using MerMaid-12 DNA/RNA synthesizer (Bio automation, USA). Analytical anion-exchange HPLC and reverse phase HPLC were performed on Agilent 1260 Infinity Analytical SFC System combined with an Agilent 1100 series quaternary pump with a degasser. Purified oligonucleotides were desalted by Sephadex G-25 (GE Healthcare).

### Synthesis of ^i^*E*-VP-linked dimer phosphoramidites

#### 3-N-Benzoyl-5′-O-[bis(4-methoxyphenyl)phenylmethyl]-2′-deoxy-2′-fluoro-3′-O-(tert-butyldiphenylsilyl)-uridine (2a)

Anhydrous solution of compound 1a (16.6 g, 20.8 mmol) in pyridine (100 ml) was added anhydrous *N*,*N*-diisopropylethylamine (6.5 ml, 37.4 mmol) and benzoyl chloride (3.6 ml, 31.2 mmol). After the mixture was stirred for 4 h at rt, excess pyridine was evaporated and diluted with CH_2_Cl_2_. The organic solution was washed by sat. aq. NaHCO_3_. The organic layer was collected, dried over MgSO_4_, filtered and evaporated. Obtained crude material was purified by silica gel column chromatography (hexane-ethyl acetate, 4:1 to 1:1) yielding compound 2a as a slightly yellow foam (14.5 g, 78%); ^1^H NMR (500 MHz, CDCl_3_) *δ* 7.88–7.87 (m, 2H), 7.84 (d, 1H, *J* = 8.3 Hz), 7.67–7.58 (m, 5H), 7.48–7.45 (m, 4H), 7.39–7.32 (m, 4H), 7.25–7.23 (m, 3H), 7.18–7.17 (m, 2H), 7.12–7.07 (m, 4H), 6.80–6.75 (m, 4H), 6.08 (dd, 1H, *J*_HH_ = 1.5 Hz, *J*_HF_ = 15.2 Hz), 5.14, (d, 1H, *J*_HH_= 8.3 Hz), 4.59 (ddd, 1H, *J*_HH_ = 3.7, 1.5 Hz, *J*_HF_ = 51.9 Hz), 4.43 (ddd, 1H, *J*_HH_ = 7.4, 4.0 Hz, *J*_HF_ = 19.1 Hz), 4.24–4.23 (m, 1H), 3.79 (s, 6H), 3.62 (dd, 1H, *J*_HH_ = 11.2, 2.0 Hz), 3.35 (dd, 1H, *J*_HH_ = 11.1, 2.0 Hz), 1.00 (s, 9H); ^13^C NMR (126 Hz, CDCl_3_) *δ* 168.4, 161.8, 158.72, 158.66, 148.9, 143.9, 139.4, 135.71. 135.70, 135.1, 134.8, 134.7, 132.3, 132.2, 131.3, 130.4, 130.2, 130.1, 129.1, 128.2, 128.0,127.91, 127.89, 127.2, 113.19, 113.16, 102.2, 92.5 (d, *J*_CF_ = 194.4 Hz), 87.7 (d, *J*_CF_ = 34.5 Hz), 87.2, 82.4, 70.0 (d, *J*_CF_ = 15.4 Hz), 60.7, 60.4, 55.2, 26.6; ^19^F NMR (470 MHz, CDCl_3_) *δ* -201.48 (ddd, *J* = 52.0, 17.3, 17.3 Hz); HRMS (ESI) calcd. for C_53_H_51_FN_2_O_8_SiNa^+^ [M + Na]^+^*m/z* 913.3291, found *m/z* 913.3294.

#### 3-N-Benzoyl-2′-deoxy-2′-fluoro-3′-O-(tert-butyldiphenylsilyl)-uridine (3a)

Compound 2a (14.5 g, 16.3 mmol) was dissolved into 3% trichloroacetic acid/CH_2_Cl_2_ solution (200 ml) containing triethylsilane (8.0 ml, 50.1 mmol) and stirred for 1 h at rt. After the solution was washed by sat. aq. NaHCO_3_ three times, collected organic layer was dried over MgSO_4_, filtered, and evaporated. Obtained crude material was purified by silica gel column chromatography (hexane/ethyl acetate, 4:1 to 3:7) yielding compound 3a as a white foam (8.67 g, 91%); ^1^H NMR (500 MHz, CDCl_3_) *δ* 7.89–7.88 (m, 2H), 7.68–7.64 (6H, m), 7.51–7.45 (m, 4H), 7.42–7.38 (m, 4H), 5.93 (dd, 1H, *J*_HH_= 2.9 Hz, *J*_HF_ = 15.1 Hz), 5.73 (d, 1H, *J*_HH_ = 8.2 Hz), 4.74 (ddd, 1H, *J*_HH_ = 4.1, 3.2 Hz, *J*_HF_ = 52.2 Hz), 4.31 (ddd, 1H, *J*_HH_ = 5.8, 4.7, *J*_HF_ = 15.4 Hz), 4.11–4.09 (m, 1H), 3.82–3.79 (m, 1H), 3.39 (ddd, 1H, *J*_HH_ = 12.1, 5.6, 1.5 Hz), 1.64 (br-s, 1H), 1.11 (s, 9H); ^13^C NMR (126 Hz, CDCl_3_) *δ* 168.3, 161.8, 149.0, 140.5, 135.7, 135.2, 132.8, 132.3, 131.3, 130.5, 130.4, 130.3, 129.2, 128.02, 127.96, 102.4, 91.8 (d, *J*_CF_ = 91.8 Hz), 89.5 (d, *J*_CF_ = 33.6 Hz), 69.5 (d, *J*_CF_ = 69.5 Hz), 60.3, 26.8; ^19^F NMR (470 MHz, CD_3_CN) *δ* -203.20 (ddd, *J =* 52.0, 17.4, 17.4 Hz); HRMS (ESI) calcd. for C_32_H_33_FN_2_O_6_SiNa^+^ [M + Na]^+^*m/z* 611.1984, found *m/z* 611.1988.

#### 6′-Dibromo-(5′-deoxy-5′-methylidene)-3′-O-(tert-butyldiphenylsilyl)-2′-deoxy-2′-fluoro-uridine (5a)

Anhydrous solution of compound 3a (6.5 g, 11.0 mmol) was added IBX (7.7 g, 27.6 mmol) and stirred for 2 h at 85°C. After cooling the mixture in an ice bath, the precipitate in the solution was filtered off through celite. Collected eluent was evaporated, co-evaporated with anhydrous CH_3_CN three times under argon atmosphere, and obtained compound 4a as a white foam was used without further purification. In a separate flask, anhydrous CH_2_Cl_2_ (25 ml) solution containing CBr_4_ (7.3 g, 22.1 mmol) was added PPh_3_ (11.6 g, 44.2 mmol) at 0°C and stirred for 0.5 h at 0°C. To this solution, anhydrous CH_2_Cl_2_ solution (25 ml) of compound 4a was added dropwise (10 min) at 0°C and stirred for 2 h at rt. After diluting with CH_2_Cl_2_, the organic solution was washed by aq. sat. NH_4_Cl, dried over MgSO_4_, filtered, and evaporated. Obtained material was dissolved into minimum amount of diethyl ether and added dropwise to excess diethyl ether solution under vigorously stirring at 0°C. Precipitate in solution was filtered off through celite and eluents was evaporated. Obtained crude material was purified by silica gel column chromatography (hexane/ethyl acetate, 9:1 to 1:1) yielding compound 5a as a white foam (4.3 g, 52% in 2 steps). ^1^H NMR (500 MHz, CDCl_3_) *δ* 7.68–7.84 (m, 2H), 7.70–7.65 (m, 3H), 7.60–7.58 (m, 2H), 7.52–7.49 (m, 2H), 7.42–7.36 (m, 4H), 7.31–7.28 (m, 2H), 7.09 (d, 1H, *J* = 8.2 Hz), 6.25 (d, 1H, *J* = 8.9 Hz), 5.75 (dd, 1H, *J*_HF_ = 8.24 Hz), 5.49 (dd, 1H, *J*_HF_ = 21.4 Hz), 4.77 (t, 1H, *J*_HH_ = 8.5 Hz, *J*_HF_ = 8.5 Hz), 4.38 (dd, 1H, *J*_HH_ = 4.1 Hz, *J*_HF_ = 52.1 Hz), 4.25 (ddd, 1H, *J*_HH_ = 8.1, 4.9 Hz, *J*_HF_ = 19.4 Hz), 1.10 (s, 9H); ^13^C NMR (126 Hz, CDCl_3_) *δ* 167.9, 161.6, 148.3, 141.4, 135.8, 134.7 (d, *J*_C-Br_ = 139.0 Hz), 132.5, 132.2, 131.1, 130.5, 130.3, 130.2, 129.2, 127.9, 102.7, 97.3, 93.3 (d, *J*_CF_ = 39.1 Hz), 91.5 (d, *J*_CF_ = 190.7 Hz), 82.4, 73.9 (d, *J*_CF_ = 16.4 Hz), 26.7; ^19^F NMR (470 MHz, CD_3_CN) *δ* -198.53 (ddd, *J* = 52.0, 20.8, 20.8 Hz); HRMS (ESI) calcd. for C_33_H_32_Br_2_FN_2_O_5_Si^+^ [M + H]^+^*m*/*z* 741.0426, found *m*/*z* 741.0438.

#### (E)/(Z)-6′-Bromo-(5′-deoxy-5′-methylidene)-2′-deoxy-2′-fluoro-3′-O-(tert-butyldiphenylsilyl)-uridine (6a-E and 6a-Z)

Anhydrous solution of compound 5a (4.2 g, 5.66 mmol) in DMF (25 ml) was added dimethyl phosphite (2.09 ml, 22.6 mmol) and triethylamine (1.58 ml, 11.3 mmol) at 0°C, and then stirred overnight at rt. After the solution was diluted with ethyl acetate, the organic solution was washed with aq. sat. NH_4_Cl and brine. Then the organic solution was dried over MgSO_4_, filtered and evaporated. Obtained crude material was purified repeatedly by silica gel column chromatography (hexane/ethyl acetate, 9:1 to 1:1) until all isomeric-pure compounds were collected separately, giving compound 6a-E (1.95 g, 52%); ^1^H NMR (500 MHz, CDCl_3_) *δ* 7.87–7.85 (m, 2H), 7.89–7.85 (m, 3H), 7.61–7.59 (m, 2H), 7.52–7.48 (m, 2H), 7.45–7.32 (m, 6H), 7.08 (d, 1H, *J* = 8.2 Hz), 6.49 (d, 1H, *J* = 13.7 Hz), 5.99 (dd, 1H, *J* = 13.7, 8.1 Hz), 5.75 (d, 1H, *J* = 8.2 Hz), 5.63 (d, 1H, *J*_HF_ = 19.8 Hz), 4.43 (dd, 1H, *J*_HF_ = 52.6 Hz, *J*_HH_ = 4.3 Hz), 4.42 (t, 1H, *J*_HH_ = 8.0 Hz), 4.07 (ddd, *J*_HF_ = 19.5 Hz, *J*_HH_ = 7.8, 4.7 Hz), 1.08 (s, 9H); ^13^C NMR (126 Hz, CDCl_3_) *δ* 148.4, 140.4, 135.8, 135.7, 135.3, 133.3, 132.3, 132.4, 132.1, 131.1, 130.5, 130.4, 130.3, 129.2, 127.95, 127.93, 112.4, 102.7, 91.7 (d, *J*_CF_ = 36.3 Hz), 91.6 (d, *J*_CF_ = 191.6 Hz), 82.8, 73.9 (d, *J*_CF_ = 16.4 Hz), 26.7, 19.1; ^19^F NMR (470 MHz, CD_3_CN) *δ* – 199.04 (ddd, *J* = 52.0, 20.8, 20.8 Hz); HRMS (ESI) calcd. for C_33_H_33_BrFN_2_O_5_Si^+^ [M + H]^+^*m*/*z* 663.1321, found *m*/*z* 663.1329; and 6a-Z (0.58 g, 15%); ^1^H NMR (500 MHz, CDCl_3_) *δ* 7.87–7.85 (m, 2H), 7.68–7.65 (m, 3H), 7.61–7.59 (m, 2H), 7.52–7.48 (m, 2H), 7.42–7.39 (m, 2H), 7.34–7.29 (m, 4H), 7.12 (d, 1H, *J* = 8.2 Hz), 6.51 (d, 1H, *J* = 7.4 Hz), 5.96 (dd, 1H, *J*= 8.4 Hz, 7.4 Hz), 5.75 (d, 1H, *J* = 8.2 Hz), 5.57 (dd, 1H, *J*_HF_ = 20.6 Hz, *J*_HH_ = 1.2 Hz), 5.04 (dd, 1H, *J* = 8.2, 8.2 Hz), 4.48 (*J*_HF_ = 53.1 Hz, *J*_HH_ = 3.5 Hz), 4.24 (ddd, 1H, *J*_HF_ = 18.6 Hz, *J*_HH_ = 7.8, 4.9 Hz), 1.09 (s, 9H); ^13^C NMR (126 Hz, CDCl_3_) *δ* 168.0, 161.7, 148.4, 141.4, 135.9, 135.8, 135.2, 132.6, 132.5, 131.2, 130.6, 130.5, 130.2, 130.1, 129.2, 127.8, 127.7, 114.5, 102.6, 93.0 (d, *J*_CF_ = 37.2 Hz), 91.6 (d, *J*_CF_ = 191.6 Hz), 80.3, 74.3 (d, *J*_CF_ = 16.4 Hz), 26.7, 19.1; HRMS (ESI) calcd. for C_33_H_33_BrFN_2_O_5_Si ^+^ [M + H]^+^*m/z* 663.1321, found *m/z* 663.1331.

#### 5′-O-[Bis(4-methoxyphenyl)phenylmethyl]-2′-O-methyl-uridyl-methylphosphono-[3′(O)→5′(C)]-3-N-benzoyl-5′-deoxy-5′-methylidene-3′-O-(tert-butyldiphenylsilyl)-2′-deoxy-2′-fluoro-uridine (7a)

Anhydrous compound 6a-E (1.95 g, 2.94 mmol) and Pd(OAc)_2_ (125 mg, 0.59 mmol) and [1,1′-Bis(diphenylphosphino)ferrocene]dichloropalladium (II) (0.65 g, 1.18 mmol) were purged with argon, and then dissolved into anhydrous THF (50 ml). After adding propylene oxide (2.06 ml, 29.4 mmol), compound S2a (2.07 g, 3.24 mmol) was added in one portion and stirred at for 4 h at 70°C. After removing solvent under reduced pressure, the crude mixture was purified by silica gel column chromatography (hexane-ethyl acetate, 50:50 to 0:100) and obtained fractions containing compound 7a were further purified by silica gel column chromatography (CH_2_Cl_2_/MeOH, 100:0 to 95:5) yielding compound 7a as a mixture of diastereo-isomers (2.04 g, 57%); ^31^P NMR (202 MHz, CDCl_3_) δ 18.3, 18.2; ^19^F NMR (470 MHz, CDCl_3_) δ –197.45 (ddd, *J* = 52.1, 20.8, 20.8 Hz), –197.69 (ddd, *J* = 52.0, 20.8, 20.8 Hz); HRMS (ESI) calcd. for C_65_H_66_FN_4_O_15_PSiNa ^+^ [M + Na]^+^*m*/*z* 1243.3908, found *m*/*z* 1243.3915; For ^1^H and ^13^C NMR analysis, part of diastereoisomers were purified by preparative TLC. Least polar isomer ^1^H NMR (500 MHz, CDCl_3_) δ 8.98 (br-s, 1H), 7.88–7.84 (m, 3H), 7.69–7.64 (m, 3H), 7.58–7.64 (m, 2H), 7.51–7.48 (m, 2H), 7.40–7.29 (m, 10H), 7.26–7.23 (m, 5H), 7.01 (d, 1H, J = 8.0 Hz), 6.85–6.83 (m, 4H), 6.04 (d, 1H, *J* = 3.5 Hz), 5.89 (ddd, 1H, *J* = 18.8, 17.2, 1.4 Hz), 5.73 (d, 1H, *J* = 8.2 Hz), 5.59 (dd, 1H, *J*_HF_ = 20.4 Hz, *J*_HH_ = 1.3 Hz), 5.24 (d, 1H, *J* = 8.2 Hz), 5.06 (dd, 1H, *J* = 11.7, 6.1 Hz), 4.43–4.38 (m, 1H), 4.32–4.22 (m, 2H), 4.14–4.07 (m, 2H), 3.78 (s, 6H), 3.69 (d, *J*_PH_ = 11.3 Hz), 3.61 (s, 3H), 3.60–3.55 (m, 1H), 3.42–3.41 (m, 1H), 1.08 (s, 9H); ^13^C NMR (125 MHz, CDCl_3_) δ 167.9, 162.8, 161.5, 158.79, 158.75, 150.1, 148.4, 147.4 (d, *J*_CP_ = 6.4 Hz), 144.1, 140.9, 139.6, 134.7, 135.7, 135.3, 134.92, 134.87, 132.3, 132.0, 130.4, 130.34, 130.28, 130.2, 130.1, 129.2, 128.2, 128.11, 128.06, 127.98, 127.95, 127.89, 127.3, 118.0 (d, *J*_CP_ = 190.8 Hz), 113.3, 103.0, 102.4, 92.1 (d, *J*_CF_ = 7.3 Hz), 91.1 (d, *J*_CF_ = 163.5 Hz), 87.3, 86.8, 82.8, 82.4, 81.7, 81.6 (d, *J*_CF_ = 15.4 Hz), 74.3 (d, *J*_CF_ = 17.3 Hz), 71.6 (d, *J*_CP_ = 4.6 Hz), 61.2, 58.6, 58.5, 55.2 52.9, 52.7 (d, *J*_CP_ = 6.3 Hz), 45.8, 26.7, 19.1; most polar isomer ^1^H NMR (500 MHz, CDCl_3_) δ 8.47 (br-s, 1H), 7.87–7.84 (m, 3H), 7.65–7.64 (m, 3H), 7.59–7.56 (m, 2H), 7.51–7.48 (m, 2H), 7.42–7.23 (m, 15H), 7.90 (d, 1H, *J* = 8.2 Hz), 6.87–6.85 (m, 4H), 6.73 (ddd, 1H, *J* = 22.7, 17.1, 5.8 Hz), 6.02 (ddd, 1H, *J* = 18.6, 7.0, 1.1 Hz), 6.00 (d, 1H, *J* = 3.3 Hz), 5.78 (d, 1H, *J* = 8.2 Hz), 5.73 (dd, 1H, *J*_HF_ = 19.3 Hz, *J*_HH_ = 1.4 Hz), 5.23 (d, 1H, *J* = 8.2 Hz), 5.14–5.10 (m, 1H), 4.59–4.56 (m, 1H), 4.37 (ddd, *J*_HF_ = 52.3 Hz, *J*_HH_ = 3.4, 1.4 Hz), 4.27–4.26 (m, 1H), 4.10–4.07 (m, 1H), 3.98 (dd, 1H, *J* = 4.7, 3.5 Hz), 3.80 (s, 6H), 3.64 (dd, 1H, *J* = 11.1, 2.3 Hz), 3.52–3.47 (m, 4H), 3.38 (s, 3H), 1.08 (s, 9H); ^13^C NMR (125 MHz, CDCl_3_) δ 167.9, 162.6, 161.6, 158.9, 158.8, 149.9, 148.5, 146.6 (d, *J*_CP_ = 6.4 Hz), 143.9, 140.2, 139.6, 135.73, 135.69, 134.9, 134.7, 132.11, 132.09, 131.1, 130.4, 130.4, 130.3, 130.24, 130.20, 130.1, 129.2, 128.3, 128.13, 128.07, 127.97, 127.91, 127.4, 120.0 (d, *J*_CP_ = 192.5 Hz), 113.3, 103.0, 102.4, 91.4 (d, *J*_CF_ = 192.5 Hz), 91.2 (d, *J*_CF_ = 33.6 Hz), 87.4, 86.9, 82.5, 82.2, 81.8 (d, *J*_CP_ = 7.3 Hz), 74.1 (d, *J*_CF_ = 15.5 Hz), 72.0 (d, *J*_CP_ = 5.4 Hz), 61.1, 58.7, 58.5, 55.3, 52.2 (d, *J*_CP_ = 5.5 Hz), 26.7, 19.2.

#### 5′-O-[Bis(4-methoxyphenyl)phenylmethyl]-2′-O-methyl-uridyl-methylphosphono-[3′(O)→5′(C)]-3-N-benzoyl-2′,5′-dideoxy-2′-fluoro-5′-methylidene-uridine(8a)

Compound 7a (2.0 g, 1.64 mmol) in anhydrous THF (22.5 ml) was added 1.0 M TBAF-THF (2.5 ml, 2.5 mmol) and stirred at ambient temperature for 30 min. After diluting with CH_2_Cl_2_ (120 ml), the organic layer was washed with brine, dried over MgSO_4_, filtered, and then evaporated. Obtained crude material was purified by silica gel column chromatography (1%TEA-CH_2_Cl_2_/MeOH, 100:0 to 94:6) yielding compound 8a (1.52 g, 94%); ^31^P NMR (202 MHz, CDCl_3_) δ 19.0, 18.7; HRMS (ESI) calcd. for C_49_H_48_FN_4_O_15_PNa^+^ [M + Na]^+^*m*/*z* 1005.2730, found *m*/*z* 1005.2717; for^1^H and ^13^C NMR analysis, part of diastereoisomers were purified by prep-TLC. Least polar isomer ^1^H NMR (500 MHz, CD_3_CN) δ 9.08 (br-s, 1H), 7.99–7.97, 7.76–7.73, 7.66 (d, 1H, *J* = 8.2 Hz), 7.58–7.56 (m, 2H), 7.52 (d, 1H, *J* = 8.2 Hz), 7.45–7.44 (m, 2H), 7.34–7.31 (m, 7H), 6.92–6.83 (m, 5H), 6.11 (ddd, 1H, *J* = 20.1, 17.2, 1.4 Hz), 5.88–5.83 (m, 3H), 5.31 (dd, 1H, *J* = 8.3, 2.2 Hz), 5.16 (dd, 1H, *J*_HF_ = 52.5 Hz, *J*_HH_= 4.0 Hz), 5.02 (ddd, 1H, *J* = 8.0, 5.3, 5.3 Hz), 4.48–4.44 (m, 1H), 4.24–4.22 (m, 1H), 4.21–4.18 (m, 1H), 4.01 (dd, 1H, J = 4.7, 4.7 Hz), 3.94 (d, 1H, *J* = 7.7 Hz), 3.77 (s, 6H), 3.61 (d, *J*_PH_= 11.3 Hz), 3.43–3.42 (m, 2H), 3.41 (s, 3H); ^13^C NMR (125 MHz, CD_3_CN) δ 170.2, 163.7, 163.2, 159.9, 151.4, 150.2, 148.6 (d, *J*_CP_= 5.5 Hz), 145.6, 142.8, 141.0, 136.6, 136.4, 136.3, 132.3, 131.2, 131.1, 130.5, 129.1, 129.0, 128.7, 128.1, 120.9 (d, *J*_CP_ = 218.9 Hz), 114.21, 114.20, 103.0, 94.2 (d, *J*_CF_ = 185.3 Hz), 91.9 (d, *J*_CF_ = 37.2 Hz), 88.1, 87.9, 82.9, 82.8, 82.6 (d, *J*_CF_ = 3.6 Hz), 82.5, 82.3,73.7 (d, *J*_CF_ = 16.3 Hz), 73.1, 73.0, 62.7, 59.1, 59.0, 56.0, 53.2 (d, *J*_CP_ = 5.4 Hz); ^19^F NMR (470 MHz, CD_3_CN) δ –200.1 (ddd, *J* = 47.1, 21.2, 21.2 Hz); ^31^P NMR (202 MHz, CD_3_CN) δ 18.2; most polar isomer ^1^H NMR (500 MHz, CD_3_CN) δ 9.22 (br-s, 1H), 7.98–7.96 (m, 2H), 7.76–7.74 (m, 1H), 7.68–7.66 (m, 1H), 7.57–7.54 (m, 2H), 7.48 (d, 1H, J = 8.2 Hz), 7.44–7.42 (m, 2H), 7.34–7.30 (m, 7H), 6.90–6.87 (m, 5H), 6.07 (ddd, 1H, *J* = 20.1, 17.3, 1.6 Hz), 5.88–5.87 (m, 1H), 5.83–5.79 (m, 1H), 5.33 (dd, 1H, *J* = 8.2, 2.1 Hz), 5.15 (dd, 1H, *J*_HF_ = 53.0 Hz, *J*_HH_= 4.6 Hz), 5.00–4.96 (m, 1H), 4.36–4.32 (m, 1H), 4.20–4.09 (m, 2H), 4.05–4.03 (m, 1H), 3.76 (s, 6H), 3.69 (d, 3H, *J*_PH_ = 11.2 Hz), 3.49 (s, 3H), 3.43–3.41 (m, 2H), 3.34 (dd, 1H, *J* = 11.3, 3.5 Hz); ^13^C NMR (125 MHz, CD_3_CN) δ 169.7, 163.4, 162.7, 159.40, 159.38, 150.9, 149.7, 148.6 (d, *J*_CP_ = 6.3 Hz), 145.2, 142.5, 140.6, 136.2, 136.02, 135.96, 131.89, 130.9, 130.72, 130.67, 130.1, 128.62, 128.59, 127.7, 119.0 (d, *J*_CP_ = 176.1 Hz), 113.80, 113.78, 102.59, 102.55, 93.6 (d, *J*_CF_ = 185.3 Hz), 91.5 (d, *J*_CF_ = 36.3 Hz), 87.8, 87.4, 82.4, 82.2 (d, *J*_CF_ = 6.4 Hz), 81.9, 81.8, 73.2 (d, *J*_CF_ = 16.4 Hz), 72.32, 72.27, 62.2, 58.6, 55.5, 53.1 (d, *J*_CP_ = 6.4 Hz); ^19^F NMR (470 MHz, CD_3_CN) δ –200.2 (ddd, *J* = 52.1, 20.8, 20.8 Hz); ^31^P NMR (202 MHz, CD_3_CN) δ 18.1.

#### 5′-O-[Bis(4-methoxyphenyl)phenylmethyl]-2′-O-methyl-uridyl-methylphosphono-[3′(O)→5′(C)]-3-N-benzoyl-2′,5′-dideoxy-2′-fluoro-methylidene-uridine, 3′-[2-cyanoethyl-N,N-bis(1-methylethyl)- phosphoramidite] (9a)

Compound 8a (589.7 mg, 0.6 mmol) was rendered anhydrous by repeated co-evaporation with anhydrous CH_3_CN and then dissolved into anhydrous CH_2_Cl_2_ (6.0 ml). To this solution *N*,*N*-diisopropylethylamine (0.31 ml, 1.8 mmol) and 2-cyanoethyl *N*,*N*-diisopropylchlorophosphoramidite (0.16 ml, 0.72 mmol) were added at 0°C. After stirring for 30 min at 0°C, the reaction mixture was diluted with excess CH_2_Cl_2_. The organic layer was repeatedly washed with aq. sat. NaHCO_3_, dried over MgSO_4_, filtered, and evaporated. The obtained crude material was purified by silica gel column chromatography (1%TEA-CH_2_Cl_2_/MeOH, from 100:0 to 96:4) yielding compound 9a as a white foam (0.57 g, 80%); ^31^P NMR (202 MHz, CDCl_3_) δ 150.3, 151.2, 151.1, 151.0, 18.72, 18.65, 18.55, 18.3; HRMS (ESI) calcd. for C_58_H_65_FN_6_NaO_16_P_2_^+^ [M + Na]^+^*m*/*z* 1205.3809, found *m*/*z* 1205.3799.

#### 3-N-Benzoyl-2′-O-methyl-3′-O-(tert-butyldiphenylsilyl)-uridine (3b)

Anhydrous solution of compound 1b (1.35 g, 2.0 mmol) in pyridine (10 ml) was added *N*,*N*-diisopropylethylamine (0.63 ml, 3.6 mmol) and benzoyl chloride (0.35 ml, 3.0 mmol), and stirred for 3 h at rt. After diluting with excess CH_2_Cl_2_, the organic solution was washed with sat. NaHCO_3_ aq. and brine. After drying over MgSO_4_, filtered and evaporating, obtained crude material containing compound 2b was used for the next reaction without further purification. Obtained crude material containing compound 2b was added 3% trichloroacetic acid in CH_2_Cl_2_ (25 ml) and triethylsilane (1 ml, 6.26 mmol), and stirred for 1 h at rt. After the reaction mixture was diluted with CH_2_Cl_2_, the solution was washed with sat. NaHCO_3_ aq. three times, dried over MgSO_4_, filtered, then evaporated. Obtained crude material was purified by silica gel column chromatography (hexane/ethyl acetate, 4:1 to 1:4) yielding pure compound 3b (0.60 g, 63% in 2 steps); ^1^H NMR (500 MHz, DMSO-*d*_6_) *δ* 8.13 (d, 1H, *J* = 8.2 Hz), 7.95 (d, 2H, *J* = 7.3 Hz), 7.81 (t, 1H, *J* = 7.5 Hz), 7.69–7.68 (m, 2H), 7.64–7.59 (m, 4H), 7.49–7.42 (m, 6H), 5.93 (d, 1H, *J* = 4.6 Hz), 5.26 (t, 1H, *J* = 4.6 Hz), 4.36 (dd, 1H, *J* = 4.6, 4.6 Hz), 4.02–4.00 (m, 1H), 3.65–3.61 (m, 1H), 3.54 (dd, 1H, *J* = 4.6, 4.6 Hz), 3.09 (s, 3H), 1.03 (s, 9H); ^13^C NMR (126 Hz, DMSO-*d*_6_) *δ* 169.8, 162.1, 149.5, 141.3, 136.1, 135.9, 135.8, 133.4, 133.2, 131.5, 130.7, 130.52, 130.48, 130.0, 128.4, 128.3, 102.1, 86.7, 85.6, 82.8, 79.7, 70.8, 60.2, 57.8, 27.2, 19.4; HRMS (ESI) *m*/*z* calcd for C_33_H_37_N_2_O_7_Si^+^ [M + H]^+^*m*/*z* 601.2365, found *m*/*z* 601.2372.

#### 6′-Dibromo-(5′-deoxy-5′-methylidene)-2′-O-methyl-3′-O-(tert-butyldiphenylsilyl)-uridine (5b)

Anhydrous solution of compound 3b (0.3 g, 0.5 mmol) in CH_3_CN (5 ml) was added IBX (0.35 g, 1.3 mmol) and stirred for 2 h at 85°C. After cooling the solution at 0°C, the precipitate was filtered off by celite-filtration. Obtained eluent containing compound 4b was evaporated, rendered anhydrous by repeated co-evaporation with anhydrous CH_3_CN, and used for the next reaction without further purification. Separately prepared anhydrous solution of CBr_4_ (0.33 g, 1.0 mmol) in CH_2_Cl_2_ (5.0 ml) was added triphenylphosphine (524.6 mg, 2.0 mmol) at 0°C in one portion and stirred at 0°C for 30 min. To this solution, compound 4b in anhydrous CH_2_Cl_2_ (1.5 ml) was added dropwise (10 min) at 0°C and stirred for 2 h at rt. The solution was then diluted with CH_2_Cl_2_ and washed with sat. NaHCO_3_ aq. and brine. After the organic solution was dried over MgSO_4_, filtered and evaporated, obtained crude material was purified by silica gel column chromatography (hexane/ethyl acetate, 9:1 to 4:6) yielding compound 5b (0.21 g, 56% in 2 steps); ^1^H NMR (500 MHz, CDCl_3_) *δ* 7.88 (d, 2H, *J* = 7.3 Hz), 7.70–7.62 (5H, m), 7.51–7.38 (m, 9H), 7.08 (d, 1H, *J* = 8.2 Hz), 6.26 (d, 1H, *J* = 8.6 Hz), 5.75 (d, 1H, *J* = 8.2 Hz), 5.68 (d, 1H, *J* = 0.8 Hz), 4.84 (dd, 1H, *J* = 8.6, 8.6 Hz), 3.86 (dd, 1H, *J* = 7.5, 5.0 Hz), 3.30 (s, 3H), 3.18 (br-s, 1H), 1.11 (s, 9H); ^13^C NMR (126 MHz, CDCl_3_) *δ* 168.3, 161.7, 148.6, 138.9, 135.9, 135.8, 134.3, 132.6, 132.4, 131.2, 130.5, 130.4, 130.3, 129.2, 128.0, 127.9, 102.4, 97.5, 90.0, 82.44, 82.39, 74.4, 58.2, 26.7, 19.1; HRMS (ESI) *m*/*z* calcd for C_34_H_35_Br_2_N_2_O_6_Si^+^ [M + H]^+^*m*/*z* 753.0626 [M + H] ^+^, found *m*/*z* 753.0637.

#### (E)/(Z)-6′-Bromo-(5′-deoxy-5′-methylidene)-2′-O-methyl-3′-O-(tert-butyldiphenylsilyl)-uridine (6b-E and 6b-Z)

Anhydrous solution of compound 5b (6.11 g, 8.1 mmol) in DMF (35 ml) was added dimethyl phosphite (2.97 ml, 34.0 mmol) and triethylamine (2.26 ml, 17.0 mmol) at 0°C, and then stirred overnight at rt. After the solution was diluted with ethyl acetate, the organic solution was washed with sat. NH_4_Cl aq. and brine. Then the organic solution was dried over MgSO_4_, filtered and evaporated, and obtained crude material was purified repeatedly by silica gel column chromatography (hexane/ethyl acetate, 9:1 to 1:1) until all pure isomeric compound were collected separately, giving compound 6b-E (3.0 g, 55%); ^1^H NMR (500 MHz, CDCl_3_) *δ* 7.89–7.87 (m, 2H), 7.70–7.62 (m, 5H), 7.51–7.39 (m, 8H), 7.10 (d, 1H, *J* = 8.3 Hz), 6.47 (dd, 1H, *J* = 13.6, 0.8 Hz), 6.01 (dd, 1H, *J* = 13.6, 7.9 Hz), 5.76–5.74 (m, 2H), 4.51 (dd, 1H, *J* = 7.8, 7.8 Hz), 7.36 (dd, 1H, = 7.8, 4.9 Hz), 3.34 (s, 3H), 3.17 (dd, 1H, *J*= 4.7, 1.2 Hz), 1.09 (s, 9H); ^13^C NMR (126 MHz, CDCl_3_) *δ* 168.3, 161.7, 148.7, 138.4, 135.9, 135.8, 135.3, 133.8, 132.6, 132.4, 131.2, 130.5, 130.4, 130.3, 129.2, 128.0, 127.9, 112.1, 102.3, 88.9, 82.8, 82.6, 77.2, 74.2, 58.1, 26.8, 19.1; HRMS (ESI) *m*/*z* calcd for C_34_H_36_BrN_2_O_6_Si^+^ [M + H] ^+^*m*/*z* 675.1521 [M + H] ^+^, found *m*/*z* 675.1526 and 6b-Z (1.23 g, 22%); ^1^H NMR (500 MHz, CDCl_3_) *δ* 7.89–7.87 (m, 2H), 7.72–7.70 (m, 2H), 7.68–7.63 (m, 3H), 7.51–7.44 (m, 4H), 7.41–7.37 (m, 4H), 7.16 (d, 1H, *J* = 8.2 Hz), 6.53 (dd, 1H, *J*= 7.4, 0.6 Hz), 6.03 (dd, 1H, *J* = 8.5, 7.4 Hz), 5.75–5.73 (m, 2H), 5.12 (t, 1H, *J* = 8.1 Hz), 3.93 (dd, 1H, *J* = 6.9, 5.0 Hz), 3.32 (br-s, 1H), 3.26 (s, 3H), 1.10 (s, 9H); ^13^C NMR (126 MHz, CDCl_3_) *δ* 168.3, 161.8, 148.7, 139.3, 135.91, 135.85, 135.22, 132.74, 132.71, 131.2, 130.8, 130.5, 130.23, 130.16, 129.2, 127.78, 127.75, 114.6, 102.2, 90.1, 82.4, 80.6, 77.2, 74.8, 58.1, 26.8, 19.2; C_34_H_36_BrN_2_O_6_Si^+^ [M + H] ^+^*m*/*z* 675.1521 [M + H] ^+^, found *m*/*z* 675.1529.

#### 5′-O-[Bis(4-methoxyphenyl)phenylmethyl]-2′-deoxy-2′-fluoro-uridyl methylphosphono-[3′(O)→5′(C)] -3-N-benzoyl-5′-deoxy-5′-methylidene-3′-O-(tert-butyldiphenylsilyl)-2′-O-methyl-uridine (7b)

Anhydrous compound 6b-E (2.84 g, 4.20 mmol) and Pd(OAc)_2_ (188.6 mg, 0.84 mmol) and [1,1′-Bis(diphenylphosphino)ferrocene]dichloropalladium (II) (931.4 mg, 1.68 mmol) were purged with argon, and then dissolved into anhydrous THF (50 ml). After adding propylene oxide (2.94 ml, 42.0 mmol), compound S2b (3.16 g, 5.04 mmol) was added in one portion and stirred at for 4 h at 70°C. After removing solvent under reduced pressure, the crude mixture was purified by silica gel column chromatography (hexane/ethyl acetate, 50:50 to 0:100) and obtained fractions containing compound 7b were further purified by silica gel column chromatography (1%TEA-CH_2_Cl_2_/MeOH, 100:0 to 95:5) yielding pure compound 7b as a mixture of diastereoisomers (3.3 g, 64%); HRMS (ESI) calcd. for C_65_H_66_FN_4_NaO_15_PSi^+^ [M + Na]^+^*m*/*z* 1243.3908 [M + Na]^+^, found *m*/*z* 1243.3912. For ^1^H, ^13^C, ^19^F, and ^31^P NMR analysis, part of diastereoisomers were purified by preparative TLC. Least polar isomer ^1^H NMR (500 MHz, CDCl_3_) δ 9.12 (br-s, 1H), 7.89–7.85 (m, 3H), 7.77–7.59 (m, 5H), 7.49–7.24 (m, 17H), 7.15 (d, 1H, *J* = 8.2 Hz), 6.87 (d, 4H, *J* = 7.8 Hz), 6.84–6.77 (m, 1H), 6.04 (dd, 1H, *J*_HF_ = 15.7 Hz, *J*_HH_ = 0.9 Hz), 5.98 (ddd, 1H, *J* = 20.2, 19.1, 1.1 Hz), 5.94 (d, 1H, *J* = 3.1 Hz), 5.77 (d, 1H, *J* = 8.2 Hz), 5.25 (d, 1H, *J* = 8.1 Hz), 5.22–5.07 (m, 2H), 4.67–4.64 (m, 1H), 4.29 (dd, 1H, *J* = 7.7, 1.8 Hz), 3.91 (dd, 1H, *J* = 6.4, 2.5 Hz), 3.80 (s, 6H), 3.73 (dd, 1H, *J* = 11.3, 1.8 Hz), 3.52–3.49 (m, 4H), 3.22 (s, 3H), 3.11 (dd, 1H, *J* = 3.1, 3.1 Hz), 1.08 (s, 9H); ^13^C NMR (126 MHz, CDCl_3_) δ 168.3, 162.6, 161.7, 158.85, 158.83, 149.8, 148.9, 148.8, 143.7, 139.4, 138.5, 135.8, 135.7, 135.2, 134.7, 132.5, 132.4, 131.3, 130.5, 130.4, 130.30, 130.28, 130.24, 129.2, 129.1, 128.3, 128.2, 128.1, 128.0, 127.9, 127.4, 125.3, 117.9 (d, *J*_CP_= 192.5 Hz), 113.4, 102.74, 102.69, 91.8 (d, *J*_CF_ = 91.8 Hz), 88.2, 87.7 (d, *J*_CF_ = 33.6 Hz), 87.4, 82.5 (d, *J*_CP_ = 22.7 Hz), 82.1, 80.7 (d, *J*_CF_ = 8.1 Hz), 74.0, 70.8 (dd, *J*_CP_ = 4.5 Hz, *J*_CF_ = 15.4 Hz), 60.3, 58.0, 55.3, 52.4 (d, *J*_CP_= 5.5 Hz), 26.8, 19.2; ^19^F NMR (470 MHz, CD_3_CN) δ –199.53 (ddd, *J* = 52.0, 20.8, 20.8 Hz);^31^P NMR (202 MHz, CDCl_3_) δ 19.3; most polar isomer ^1^H NMR (500 MHz, CDCl_3_) δ 8.9 (br-s, 1H), 7.89–7.87 (m, 2H), 7.83 (d, 1H, *J* = 8.1 Hz), 7.69–7.59 (m, 5H), 7.49–7.23 (m, 17H), 7.04 (d, 1H, *J* = 8.3 Hz), 6.85 (d, 2H, *J* = 3.2 Hz), 6.83 (d, 2H, *J* = 3.2 Hz), 6.82 (ddd, 1H, *J* = 23.1, 17.2, 4.3 Hz), 6.13 (dd, 1H, *J*_HF_= 15.7 Hz, *J*_HH_ = 2.2 Hz), 5.89 (ddd, 1H, *J* = 20.0, 17.2, 1.7 Hz), 5.83 (d, 1H, *J* = 2.6 Hz), 5.73 (d, 1H, *J* = 8.3 Hz), 5.29–5.28 (m, 2H), 5.19–5.11 (m, 1H), 4.57–4.54 (m, 1H), 4.27–4.25 (m, 1H), 3.87 (dd, 1H, *J* = 6.9, 5.1 Hz), 3.78 (s, 3H), 3.77 (s, 3H), 3.73 (d, 3H, *J*_PH_ = 11.4 Hz), 3.61 (dd, 2H, *J* = 11.4, 2.1 Hz), 3.43 (dd, 1H, *J* = 11.3, 2.1 Hz), 3.21 (s, 3H), 3.17–3.15 (m, 1H), 1.08 (s, 9H); ^13^C NMR (125 MHz, CDCl_3_) δ 168.2, 162.6, 161.5, 158.81, 158.78, 149.9, 148.7 (d, *J*_CP_ = 3.7 Hz), 144.0, 139.5, 138.9, 135.8, 135.7, 135.3, 134.81, 134.76, 132.45, 132.3, 131.2, 130.4, 130.3, 130.2, 130.1, 129.2, 128.08, 128.06, 128.02, 127.9, 127.3, 117.3 (d, *J*_CP_ = 192.5 Hz), 113.4, 102.71, 102.68, 92.7, 91.9 (d, *J*_CF_ = 195.3 Hz), 89.2, 87.43 (d, *J*_CF_ = 33.6 Hz), 87.37, 82.2 (d, *J*_CP_ = 21.8 Hz), 82.1, 80.9 (d, *J*_CF_ = 8.1 Hz), 74.3, 71.0 (dd, *J*_CF_ = 15.5 Hz, *J*_CP_ = 4.5 Hz), 60.6, 57.9, 55.22, 55.21, 52.8 (d, *J*_CP_= 5.6 Hz), 26.8, 19.2; ^19^F NMR (470 MHz, CD_3_CN) δ -199.43 (ddd, *J* = 52.1, 20.8, 20.8 Hz); ^31^P NMR (202 MHz, CDCl_3_) δ 18.7.

#### 5′-O-[Bis(4-methoxyphenyl)phenylmethyl]-2′-deoxy-2′-fluoro-uridyl methylphosphono-[3′(O)→5′(C)]-3-N-benzoyl-5′-deoxy-5′-methylidene-2′-O-methyl-uridine (8b)

Compound 7b (3.3 g, 2.70 mmol) in anhydrous THF (36.5 ml) was added 1.0 M TBAF-THF (4.05 ml, 4.05 mmol) and stirred at ambient temperature for 30 min. After diluting with CH_2_Cl_2_ (150 ml), the organic layer was washed with brine, dried over MgSO_4_, filtered, and then evaporated. Obtained crude material was purified by silica gel column chromatography (1%TEA-CH_2_Cl_2_/MeOH, 100:0 to 92:8) yielding compound 8b (1.25 g, 47%); HRMS (ESI) calcd. for C_49_H_48_FN_4_NaO_15_P^+^ [M + Na]^+^*m*/*z* 1005.2730, found *m*/*z* 1005.2728; For ^1^H, ^13^C, ^19^F, ^31^P NMR analysis, part of diastereoisomers were purified by preparative TLC. Least polar isomer ^1^H NMR (500 MHz, CDCl_3_) δ 9.42 (br-s, 1H), 7.96–7.94 (m, 2H), 7.87 (d, 1H, *J* = 8.3 Hz), 7.67–7.64 (m, 1H), 7.50 (t, 2H, *J* = 7.9 Hz), 7.38–7.24 (m, 10H), 7.09 (ddd, 1H, *J* = 23.8, 17.3, 3.7 Hz), 6.86 (d, 4 H, *J* = 8.8 Hz), 6.12 (ddd, 1H, *J* = 21.8, 17.3, 1.7 Hz), 6.06 (dd, 1H, *J*_HF_ = 16.3 Hz, *J*_HH_ = 1.0 Hz), 5.96 (s, 1H), 5.90 (d, 1H, *J* = 8.3 Hz), 5.32–5.17 (m, 3H), 4.50–4.48 (m, 1H), 4.34–4.32 (m, 1H), 3.95 (ddd, 1H, *J* = 14.1, 8.7, 5.5 Hz), 3.82–3.78 (m, 7H), 3.71 (dd, 1H, *J* = 11.4, 2.0), 3.58 (d, 3H, *J*_PH_ = 11.5 Hz), 3.55 (s, 3H), 3.51 (dd, 1H, *J* = 11.4, 2.5 Hz), 3.27 (d, 1H, *J* = 9.0 Hz); ^13^C NMR (125 MHz, CDCl_3_) δ 168.5, 162.8, 161.9, 158.8, 150.3, 149.0 (d, *J*_CP_ = 7.3 Hz), 143.8, 139.4, 138.7, 135.3, 134.9, 134.7, 131.2, 130.6, 130.2, 129.3, 128.3, 128.1, 127.4, 116.8, (d, *J*_CP_ = 192.6 Hz), 113.4, 102.9, 102.8, 91.7 (d, *J*_CF_ = 191.7 Hz), 88.7, 87.9 (d, *J*_CF_= 33.6 Hz), 87.4, 83.1, 82.4 (d, *J*_CP_ = 22.7 Hz), 80.9 (d, *J*_CF_ = 8.2 Hz), 72.8, 71.0 (dd, *J*_CF_ = 14.5, *J*_CP_ = 3.6 Hz), 60.4, 58.9, 55.3, 52.5 (d, *J*_CP_ = 6.4 Hz); ^19^F NMR (470 MHz, CD_3_CN) δ -199.53 (ddd, *J* = 52.0, 20.8, 20.8 Hz); ^31^P NMR (202 MHz, CDCl_3_) δ 19.7; most polar isomer ^1^H NMR (500 MHz, CDCl_3_) δ 8.63 (br-s, 1H), 7.95–7.93 (m, 2H), 7.81 (d, 1H, *J* = 8.3 Hz), 7.69–7.66 (m, 1H), 7.53–7.49 (m, 2H), 7.37–7.24 (m, 10H), 6.97 (ddd, 1H, *J* = 23.4, 17.4, 4.0 Hz), 6.86–6.83 (m, 4H), 6.07 (dd, 1H, *J*_HF_ = 16.5 Hz, *J*_HH_ = 1.1 Hz), 6.00 (ddd, 1H, *J* = 19.6, 17.4, 1.7 Hz), 5.86–5.85 (m, 2H), 5.32 (d, 1H, *J* = 8.3 Hz), 5.28 Hz (ddd, 1H, *J*_HF_ = 51.5 Hz, *J*_HH_ = 4.2, 1.5 Hz), 5.15–5.08 (m, 1H), 4.31–4.29 (m, 1H), 4.20–4.17 (m, 1H), 3.91–3.87 (m, 1H), 3.84–3.83 (m, 1H), 3.80–3.77 (m, 9H), 3.65 (dd, 1H, *J* = 11.5, 2.0 Hz), 3.58 (s, 3H), 3.40 (dd, 1H, *J* = 11.5, 2.6 Hz), 2.90 (d, 1H, *J* = 9.3 Hz); ^13^C NMR (125 MHz, CDCl_3_) δ 168.3, 162.6, 161.6, 158.8, 158.7, 149.8, 148.9, 148.8 (d, *J*_CP_ = 148.8 Hz), 144.1, 139.8, 138.8, 135.4, 135.2, 134.9, 131.2, 130.5, 130.3, 130.1, 129.3, 129.1, 128.1, 127.9, 127.8, 127.3, 117.0 (d, *J*_CP_ = 192.5 Hz), 113.4, 113.2, 102.9, 102.7, 92.0 (d, *J*_CF_ = 92.0 Hz), 89.3, 88.3 (d, *J*_CF_ = 33.6 Hz), 87.3, 82.8, 82.4 (d, *J*_CP_ = 22.7 Hz), 80.7 (d, *J*_CF_ = 8.2 Hz), 72.7, 70.9 (dd, *J*_CF_ = 16.4, *J*_CP_ = 5.5 Hz), 60.7, 58.9, 55.3, 52.8 (d, *J*_CP_ = 52.8 Hz); ^19^F NMR (470 MHz, CD_3_CN) δ -199.43 (ddd, *J* = 52.1, 20.8, 20.8 Hz); ^31^P NMR (202 MHz, CDCl_3_) δ 19.0.

#### 5′-O-[Bis(4-methoxyphenyl)phenylmethyl]-2′-deoxy-2′-fluoro-uridyl methylphosphono-[3′(O)→5′(C)]-3-N-benzoyl-5′-deoxy-5′-methylidene-2′-O-methyl-uridine, 3′-[2-cyanoethyl-N,N-bis(1-methylethyl)- phosphoramidite] (9b)

Compound 8b (393.2 mg, 0.4 mmol) was rendered anhydrous by repeated co-evaporation with anhydrous CH_3_CN and then dissolved into anhydrous CH_2_Cl_2_ (4.0 ml). To this solution *N*,*N*-diisopropylethylamine (0.21 ml, 1.2 mmol) and 2-cyanoethyl *N*,*N*-diisopropylchlorophosphoramidite (0.11 ml, 0.48 mmol) were added at 0°C. After stirring for 30 min at 0°C, the reaction mixture was diluted with excess CH_2_Cl_2_. The organic layer was repeatedly washed with sat. NaHCO_3_ aq., dried over MgSO_4_, filtered, and evaporated. The obtained crude material was purified by silica gel column chromatography (1%TEA-CH_2_Cl_2_/MeOH, from 100:0 to 96:4) yielding compound 9b as a white foam (319.6 mg, 68%); ^31^P NMR (202 MHz, CDCl_3_) δ 150.7, 150.4, 150.3, 19.9, 19.5, 19.4, 18.8. HRMS (ESI) calcd. for C_58_H_65_FN_6_NaO_16_P_2_^+^ [M + Na]^+^*m*/*z* 1205.3809, found *m*/*z* 1205.3799.

#### 5′-O-[Bis(4-methoxyphenyl)phenylmethyl]-2-N-isobutylyl-2′-O-methyl-guanyl methylphosphono-[3′(O)→5′(C)]-5′-deoxy-5′-methylidene-3′-O-(tert-butyldiphenylsilyl)-3-N-benzoyl-2′-deoxy-2′-fluoro-uridine (7c)

Anhydrous compound 6a-E (2.19 g, 3.31 mmol) and Pd(OAc)_2_ (148.2 mg, 0.66 mmol) and [1,1′-Bis(diphenylphosphino)ferrocene]dichloropalladium (II) (731.8 mg, 1.32 mmol) were purged with argon, and then dissolved into anhydrous THF (23.0 ml). After adding propylene oxide (2.32 ml. 33.1 mmol), compound S2c (2.72 g, 3.64 mmol) in anhydrous THF (10.0 ml) was added and stirred at 70°C overnight. After removing solvent under reduced pressure, the crude mixture was purified by silica gel column chromatography (ethyl acetate/MeOH, 100:0 to 96:4) yielding pure compound 7c (1.33g, 30%); HRMS (ESI) calcd. for C_70_H_74_FN_7_O_15_PSi^+^ [M + H]^+^*m*/*z* 1330.4728, found *m*/*z* 1330.4744; For ^1^H, ^13^C, ^31^P, ^19^F NMR analysis, part of diastereoisomers were separated by preparative TLC. Least polar isomer ^1^H NMR (500 MHz, CD_3_CN) δ 11.9 (br-s, 1H), 10.2 (br-s, 1H), 7.94–7.90 (m, 3H), 7.76–7.73 (m, 1H), 7.67–7.54 (m, 6H), 7.45–7.20 (m, 16H), 6.85–6.76 (m, 4H), 6.17 (ddd, 1H, *J* = 20.2, 17.3, 1.1 Hz), 5.94 (d, 1H, *J* = 3.6 Hz), 5.87–5.84 (m, 1H), 5.74 (d, 1H, *J* = 8.3 Hz), 5.72 (d, *J*_HF_ = 22.0 Hz), 4.75 (dd, 1H, *J*_HF_ = 52.6 Hz, *J*_HH_ = 4.6 Hz), 4.66–4.63 (m, 2H), 4.40–4.33 (m, 2H), 3.77 (s, 3H), 3.76 (s, 3H), 3.54 (d, 3H, *J*_PH_ = 11.3 Hz), 3.40 (dd, 1H, *J* = 11.1, 2.4 Hz), 3.25 (dd, 1H, *J* = 8.1, 4.1 Hz), 3.22 (s, 3H), 2.63–2.57 (m, 1H), 1.13 (d, 3H, *J* = 2.6 Hz), 1.12 (d, 3H, *J* = 2.6 Hz), 1.07 (s, 9H); ^13^C NMR (125 MHz, CD_3_CN) δ 180.4, 169.5, 162.7, 159.23, 159.21, 155.9, 149.4, 148.6, 148.2 (d, *J*_CP_ = 5.4 Hz), 145.3, 143.5, 139.1, 136.3, 136.24, 136.22, 136.19, 136.1, 133.0, 132.8, 131.8, 130.9, 130.8, 130.5, 130.4, 130.0, 129.5, 128.8, 128.54, 128.46, 128.42, 128.3, 127.5, 125.8, 120.1 (d, *J*_CP_ = 189.8 Hz), 113.5, 102.4, 93.3 (d, *J*_CF_ = 37.2 Hz), 92.6 (d, *J*_CF_ = 188.0 Hz), 86.6, 86.3, 82.7, 82.5, 81.9 (d, *J*_CF_ = 5.4 Hz), 81.8 (d, *J*_CP_ = 1.9 Hz), 74.8 (d, *J*_CF_ = 16.3 Hz), 73.2 (d, *J*_CP_ = 4.5 Hz), 62.6, 58.6, 55.5, 53.0 (d, *J*_CP_ = 5.4 Hz), 36.5, 26.7, 19.3, 18.8, 18.7; ^31^P NMR (202 MHz, CD_3_CN) δ 18.6; ^19^F NMR (470 MHz, CD_3_CN) δ -196.54, -196.59, -196.65, -196.68; most polar isomer ^1^H NMR (500 MHz, CD_3_CN) δ 11.9 (br-s, 1H), 10.0 (br-s, 1H), 7.94–8.91 (m, 3H), 7.99–7.76 (m, 1H), 7.68–7.55 (m, 6H), 7.47–7.43 (m, 2H), 7.40–7.33 (m, 8H), 7.24–7.18 (m, 8H), 6.79–6.76 (m, 5H), 6.10–6.13 (m, 1H), 5.97 (d, 1H, *J* = 3.8 Hz), 5.93 (d, 1H, *J* = 3.2 Hz), 5.78–5.73 (m, 2H), 4.76–4.63 (m, 2H), 4.53 (br-s, 1H), 4.31–4.24 (m, 2H), 3.76 (s, 6H), 3.69 (d, *J*_PH_ = 11.5 Hz), 3.51–3.49 (m, 4H), 3.37–3.35 (m, 1H), 3.25–3.21 (m, 1H), 2.61–2.57 (m, 1H), 1.16–1.14 (m, 6H), 1.07–1.06 (m, 9H); ^13^C NMR (125 MHz, CD_3_CN) δ 180.4, 169.6, 162.6, 159.22, 159.21, 155.9, 149.5, 148.5, 147.7 (d, *J*_CP_ = 5.4 Hz), 145.4, 143.1, 139.2, 136.4, 136.3, 136.2, 136.14, 136.12, 133.0, 132.8, 131.8, 130.93, 130.92, 130.90, 130.8, 130.54, 130.48,130.0, 128.6, 128.5, 128.3, 127.5, 121.0 (d, *J*_CP_ = 215.3 Hz), 113.5, 102.5, 92.6 (d, *J*_CF_ = 189.0 Hz), 91.8, 86.7, 86.5, 82.5, 82.3, 82.1 (d, *J*_CF_ = 5.5 Hz), 81.7 (d, *J*_CP_= 1.2 Hz), 75.0 (d, *J*_CF_ = 14.9 Hz), 73.2 (d, *J*_CP_ = 4.6 Hz), 62.5, 58.6, 55.5, 53.40. 53.35, 36.4, 26.7, 19.3, 18.8, 18.7; ^31^P NMR (202 MHz, CD_3_CN) δ 18.6; ^19^F NMR (470 MHz, CD_3_CN) δ -197.4 (ddd, *J* = 51.7, 20.8, 20.8 Hz).

#### 5′-O-[Bis(4-methoxyphenyl)phenylmethyl]-2-N-isobutylyl-2′-O-methyl-guanyl methylphosphono-[3′(O)→5′(C)]-3-N-benzoyl-2′,5′-dideoxy-5′-methylidene-2′-fluoro-uridine (8c)

Compound 7c (0.8 g, 0.6 mmol) in anhydrous THF (8.1 ml) was added 1.0 M TBAF-THF (0.9 ml, 0.9 mmol) and stirred at ambient temperature for 30 min. After diluting with CH_2_Cl_2_ (150 ml), the organic layer was washed with brine, dried over MgSO_4_, filtered, and then evaporated. Obtained crude material was purified by silica gel column chromatography (1%TEA-CH_2_Cl_2_/MeOH, 100:0 to 92:8) yielding compound 8c (0.56 g, 85%); HRMS (ESI) calcd. for C_54_H_56_FN_7_O_15_P^+^ [M + H]^+^*m*/*z* 1092.3551, found *m*/*z* 1092.3558; ^31^P NMR (202 MHz, CD_3_CN) δ 19.1, 19.0. For ^1^H, ^13^C and ^19^F NMR analysis, part of diastereoisomers were purified by preparative TLC, and only least polar isomer was collected as an isomer pure fraction. Least polar isomer ^1^H NMR (500 MHz, CD_3_CN) δ 12.0 (br-s, 1H), 10.2 (br-s, 1H), 7.98–7.96 (m, 2H), 7.86 (s, 1H), 7.75–7.72 (m, 1H), 7.56–7.51 (m, 3H), 7.34–7.33 (m, 2H), 7.23–7.18 (m, 7H), 6.15 (ddd, 1H, *J* = 20.2, 17.2, 1.4 Hz), 5.93 (d, 1H, *J* = 3.9 Hz), 5.86–5.81 (m, 2H), 5.67 (ddd, 1H, *J* = 8.4, 5.3, 5.3 Hz), 5.18 (dd, 1H, *J*_HF_ = 52.8 Hz, *J*_HH_ = 4.7 Hz), 4.66 (dd, 1H, *J* = 4.5 Hz), 4.50–4.48 (m, 1H), 4.32–4.28 (m, 2H), 4.24–4.15 (m, 1H), 3.74 (s, 6H), 3.57 (3H, d, *J*_PH_ = 11.5 Hz), 3.39–3.36 (m, 4H), 3.30 (dd, 1H, *J* = 11.0, 4.4 Hz), 2.59 (sep, 1H, *J* = 6.9 Hz), 1.14 (d, 3H, *J* = 6.8 Hz), 1.10 (d, 3H, *J* = 6.8 Hz); ^13^C NMR (125 MHz, CD_3_CN) δ 181.1, 170.2, 163.3, 159.8, 159.7, 156.5, 150.1, 149.4, 149.13, 149.06 (d, *J*_CP_ = 6.3 Hz), 145.8, 143.2, 139.7, 136.8, 136.7, 132.3, 131.4, 131.1, 131.0, 130.5, 129.1, 129.0, 128.0, 122.2, 120.1, 114.05, 114.04, 103.0, 94.2 (d, *J*_CF_ = 185.3 Hz), 92.4 (d, *J*_CF_ = 36.3 Hz), 87.2, 82.8 (d, *J*_CF_ = 6.4 Hz), 82.5, 82.3, (dd, *J*_CP_ = 2.7, 2.7 Hz), 74.0 (d, *J*_CP_ = 4.6 Hz), 73.7 (d, J_CF_ = 16.7 Hz), 63.4, 59.2, 55.98, 55.96, 53.5 (d, *J*_CP_ = 6.3 Hz), 47.3, 36.9, 19.3, 19.2; ^19^F NMR (470 MHz, CD_3_CN) δ –199.9 (ddd, *J* = 53.9, 20.9, 20.9 Hz).

#### 5′-O-[Bis(4-methoxyphenyl)phenylmethyl]-2-N-isobutyryl-2′-O-methyl-guanyl-methylphosphono-[3′(O)→5′(C)]-3-N-benzoyl-2′,5′-dideoxy-2′-fluoro-5′-methylidene-uridine, 3′-[2-cyanoethyl N,N-bis(1-methylethyl)- phosphoramidite] (9c)

Compound 8c (557 mg, 0.51 mmol) was rendered anhydrous by repeated co-evaporation with anhydrous CH_3_CN and then dissolved into anhydrous CH_2_Cl_2_ (5.1 ml). To this solution *N*,*N*-diisopropylethylamine (0.34 ml, 1.94 mmol) and 2-cyanoethyl *N*,*N*-diisopropylchlorophosphoramidite (0.17 ml, 0.77 mmol) were added at 0°C. After stirring for 30 min at 0°C, the reaction mixture was diluted with excess CH_2_Cl_2_. The organic layer was repeatedly washed with sat. NaHCO_3_ aq., dried over MgSO_4_, filtered, and evaporated. The obtained crude material was purified by silica gel column chromatography (1% TEA-CH_2_Cl_2_/Acetone, from 100:0 to 70:30). Obtained compound 9c with a slight amount of reagent residues was dissolved in Et_2_O-ethyl acetate (1:1, v/v), repeatedly washed by sat. NaHCO_3_ aq., and then washed by brine. Organic layer was dried over MgSO_4_, filtered, and evaporated, yielding compound 9c as a white foam (367 mg, 57%); ^31^P NMR (202 MHz, CDCl_3_) δ 151.6, 151.5, 151.10, 151.05, 19.5, 19.2, 18.4, 18.3. HRMS (ESI) calcd. for C_63_H_73_FN_9_O_16_P_2_^+^ [M + H]^+^*m*/*z* 1292.4629, found *m*/*z* 1292.4643.

### Synthesis of inter-nucleotide (*E*)-vinylphosphonate modified RNA oligonucleotides

Synthesis of all RNA oligonucleotides with or without ^i^*E*-VP linkages were performed with a MerMade 12 automated RNA synthesizer (BioAutomation) on a 1 μmol UnyLinker support (ChemGenes, co.). All 2*′*-modified (2*′*-OMe and 2*′*-F) phosphoramidites (ChemGenes) and ^i^*E*-VP-linked dimer phosphoramidites were prepared at a concentration of 0.1 M in anhydrous acetonitrile. Synthesis was conducted on a standard 1.0 μmol scale RNA phosphoramidite synthesis cycle, which consists of (i) detritylation, (ii) coupling, (iii) capping, and (iv) iodine oxidation to phosphate by 0.02 M I_2_ in THF-pyridine-H_2_O (7:2:1, v/v/v), or sulfurization by 0.1 M DDTT in pyridine:CH_3_CN (9:1, v/v). Coupling of phosphoramidites was conducted with a standard protocol for 2-cyanoethyl phosphoramidite using BTT as an activator. For 5′-terminal phosphorylation, bis(2-cyanoethyl)-*N*,*N*-diisopropyl phosphoramidite (ChemGenes) was used. For the 5′-termianl (*E*)-VP modification, 5′-vinyl tetraphosphonate-(pivaloyloxymethyl)-2′-*O*-methyl-uridine 3′-CE phosphoramidite (ChemGenes) was used. For 3*′*-cholesterol modified RNA oligonucleotide synthesis, cholesterol 3*′*-lcaa CPG 500Å (ChemGenes) was used. For 3*′*-GalNAc conjugated passenger strand synthesis, GalNAc-TEG CPG 500Å (Primetech, ALC.) was used. In the case of ^i^*E*-VP modified RNA, RNA on solid support was first treated with TMSBr-pyridine-CH_2_Cl_2_ (3:1:18, v/v/v) for 1 h at ambient temperature in RNA synthesis column. Solid support was washed by water (1 ml × 3), CH_3_CN (1 ml × 3) and CH_2_Cl_2_ (1 ml × 3) by flowing solutions through the synthesis column, and then dried under vacuum. After transferring the solid support to screw-capped sample tube, base treatment by NH_4_OH-EtOH (3:1, v/v) for 48 h at 26°C was conducted. For non-^i^*E*-VP modified oligonucleotides, base treatment was conducted after chain elongation of oligonucleotides. For 5′-termianl (*E*)-VP modified guide strands, base treatment for the deprotection was conducted by NH_4_OH (3% diethylamine) at 35°C for 20 h. Crude oligonucleotides without cholesterol conjugates were purified by standard anion exchange HPLC, whereas oligonucleotides with cholesterol-conjugate were purified by reversed-phase HPLC. All obtained purified oligonucleotides were desalted by Sephadex G-25 (GE Healthcare) and characterized by electrospray ionization mass spectrometry (ESI-MS) analysis.

### General procedures for nuclease digestion tests

Oligonucleotides were incubated in buffers with or without Terminator^TM^ 5′-P-dependent 5′-exonuclease, 5′-P-independent 5′-exonuclease of phosphodiesterase II from bovine spleen (BSP), or 3′-exonuclease of snake venom phosphodiesterase I (SVPD). An aliquot of the reaction mixtures was taken at each time point and quenched by adding 95% formamide containing 20 mM EDTA, subsequently frozen by liquid nitrogen, and then kept at –80°C. Samples were heated at 95°C for 1 min, iced, and then immediately applied to the 20% formamide 20% polyacrylamide gel containing 7 M urea. After gel electrophoresis at 500 V for 2 h, gels were stained with SYBR^®^ Gold Nucleic Acid Gel Stain (Thermo Fisher Scientific), and visualized by Typhoon FLA 9000 (GE Healthcare).

#### Terminator 5′-P dependent 5′-exonuclease stability test

2.5 μM oligonucleotide (50 pmol) were incubated in RNase-free water, or with 0.17 U or 3.3U of Terminator™ (EpiCentre) exonuclease at 37°C in buffer A (EpiCentre, provided with Terminator™ enzyme).

#### BSP stability test

10 μM oligonucleotide was incubated at 37°C in RNase-free water or 30 mM NaOAc (pH 6.0) buffer containing 0.25 U/ml BSP.

#### SVPD stability test

17.5 μM oligonucleotide (50 pmol) was incubated in RNase-free water or in a 10 mM Tris–HCl buffer (pH 8.0) containing 2.0 mM MgCl_2_ and 4 mU/ml (or 10mU/ml) of SVPD (Sigma-Aldrich).

### Primer extension assays

36-nt DNA adapter with a 5′ phosphate (Integrated DNA Technologies, Inc.) was adenylated using the 5′ DNA Adenylation Kit (NEB Cat# E2610) according to manufacturer's protocol. This adenylated DNA was then ligated to sequence-matched oligonucleotides with or without ^i^*E*-VP modification (^VP^G6 for ON5 and ^Ctrl^G4 for ON7, or ^VP^G10 for ON6 and ^Ctrl^G1 for ON8, respectively) using RNA Ligase 2, truncated K227Q (NEB Cat# M0351) according to manufacturer's protocol. Ligation products were run on a 15% Polyacrylamide gel containing 7 M urea, gel extracted into 300 mM NaCl, and ethanol precipitated. The marker was prepared in the same way. The ligated marker was then hydrolyzed in 50 mM NaHCO_3_, pH 9 at 90°C for 20 min. The reaction was then put on ice and neutralized by adding an equal volume of 50 mM Tris–HCl pH 7. Reverse transcription reactions were performed with AMV Reverse Transcriptase (NEB Cat# M0277) and SuperScript II^TM^ (ThermoFisher Cat# 11904018) according to the manufacturer's protocol, using 10 pmol of a 5′-FAM labeled primer (Integrated DNA Technologies, Inc.), 5′-FAM-d-[CCTTGGCACCCGAGAATTCCA]-3′, complementary to the DNA adapter. These reactions were ethanol precipitated, resuspended in a 47.5% Formamide 10 mM EDTA loading dye, heated to 95°C for 2 min and put on ice. All reactions were then run on a 15% polyacrylamide gel containing 7 M urea and fluorescence was imaged on a Typhoon FLA 7000 (GE Healthcare).

### Thermo stability assay

1 μM guide strand and 1 μM complementary sense strand were annealed in a 10 mM sodium phosphate buffer (pH 7.2) containing 100 mM NaCl and 0.1 mM EDTA by heating at 95°C for 1 min and cooled down gradually to room temperature. *T*_m_ measurement was performed with temperature controller. Both the heating and cooling curves were measured over a temperature range from 20 to 95°C at 1.0°C/min for three times.

### In vitro screen

#### siRNA passive delivery

Cells were plated in Dulbecco's modified Eagle's medium containing 6% FBS at 8,000 cells per well in 96-well cell culture plates. siRNAs were diluted to twice the final concentration in OptiMEM (Carlsbad, CA; 31985–088), and 50 μl diluted siRNAs were added to 50 μl of cells, resulting in 3% FBS final. Cells were incubated for 72 h at 37°C and 5% CO_2_. The maximal dose in the *in vitro* dose response assays was 1.5 μM compound.

#### Quantitative analysis of target mRNA

mRNA was quantified from cells using the QuantiGene 2.0 assay kit (Affymetrix, QS0011). Cells were lysed in 250 μl diluted lysis mixture composed of one part lysis mixture (Affymetrix, 13228), two parts H_2_O and 0.167 μg/μl proteinase K (Affymetrix, QS0103) for 30 min at 55°C. Cell lysates were mixed thoroughly, and 40 μl of each lysate was added per well of a capture plate with 40 μl diluted lysis mixture without proteinase K and 20 μl diluted probe set. Probe sets for human *HTT* and Hypoxanthine Phosphoribosyltransferase (*HPRT*) (Affymetrix; *#*SA-50339, SA-10030) were diluted and used according to the manufacturer's recommended protocol. Datasets were normalized to *HPRT*.

#### Creating bar graph

Data were analyzed using GraphPad Prism 7 software (GraphPad Software, Inc., San Diego, CA). Concentration-dependent IC_50_ curves were fitted using a log (inhibitor) versus response -variable slope (four parameters). For each cell treatment plate, the level of knockdown at each dose was normalized to the mean of the control (untreated) group. The lower limit of the curve was set to less than 5, and the upper limit of the curve was set to greater than 95. To create the bar graph, the percent difference was calculated by subtracting the IC_50_ value for each compound from the IC_50_ value for each corresponding control compound, dividing by the IC_50_ value for the control compound, and multiplying by 100. If the percent difference was less than -500%, the percent difference was artificially set to -500%. The lower limit of the graph was cut at -300%.

#### Reporter plasmid preparation

Two plasmids were designed containing a 40mer sequence flanking the SNP rs362273 of the human huntingtin (*htt*) gene, in a psiCheck-2 vector (Promega cat.# C8029). Each plasmid is identical except for the base at the SNP site. PsiCheck-2 construct made via restriction cloning with XHO1 and NotI sites. Constructs were transformed into OneShot Top10 chemically competent cells. Successfully transformed cells were identified by ampicillin selection. Plasmids subsequently isolated from cells by Qiagen HiSpeed Plasmid Maxi Kit (ref. #12663).

#### Cell treatment and reporter assay

HeLa cells grown and maintained in Gibco DMEM (ref.# 11965-092) with 1% p/s and 10% heat inactivated FBS. Three days prior to treatment, two 10 cm^2^ dishes were plated with 2 × 10^6^ HeLa cells. The following day, DMEM is replaced with Gibco OptiMEM (ref. #31985–070) and 6 μg of reporter plasmid is added to cells using Invitrogen Lipofectamine 3000 (ref. #L3000-015), following the manufacturer's protocol. Cells are left in OptiMEM/lipofectamine overnight to allow for maximum reporter plasmid transfection.

The following day, siRNA was diluted in Opti-MEM and added to 96-well white wall clear bottom tissue culture plate, in triplicate, for each reporter plasmid for passive uptake. For a dose response curve, an 11 point 1:1 dilution series was performed with final siRNA treatment concentrations of 1.5, 0.7500, 0.375, 0.1875, 0.0938, 0.0469, 0.0234, 0.0117, 0.00585, 0.002925 and 0.0014625 μM. HeLa cells transfected with reporter plasmids the prior night were resuspended in DMEM with 6% heat inactivated FBS (no p/s) at 0.15 × 10^6^ cells/ml and added to plate containing siRNA.

For lipid-mediated uptake dose response curves, a seven point 1:2 dilution series was performed for final siRNA treatment concentrations of 5, 1.67, 0.55, 0.185, 0.0617, 0.0206 and 0.0069 nM. Hela cells transfected with reporter plasmids the prior night were resuspended in DMEM with 10% FBS (no p/s) at 0.0833 × 10^6^ cells/ml and 90 μl of the cell suspension was added to 96-well plates containing 10 μl siRNA formulated with RNAiMax in OptiMEM per well.

Cells were lysed after 72 h of treatment (100% confluency) with 1x Passive Lysis Buffer from Dual-Luciferase Assay System Pack (Promega ref. #E1960). Following lysis, luminescence was read after addition of 50 μl Luciferase Assay Reagent II (Promega ref. #E1960), then read a second time after addition of 50 μl/well of Stop and Glow reagent (Promega ref. #E1960). Absorbances normalized to untreated controls and graphed on a log scale.

### Evaluation of compounds efficacy and discriminative power in vivo in BAC-HD mice

BAC HD heterozygous female mice were purchased from The Jackson Laboratory (Tg(HTT*97Q)LXwy/JChdi). All animals were maintained and used according to the Institutional Animal Care and Use Committee guidelines of University of Massachusetts Medical School (docket #20210018). Mice were housed and maintained with a maximum of five to a cage until they aged to necessary time point. Mice were injected subcutaneously with 10 mg/kg of ^Ctrl^D18 and ^VP^D24 at ∼12 weeks of age. The BAC HD heterozygous mice were euthanized two weeks after subcutaneous GalNAc conjugated compounds injections. At this point mice were deeply anesthetized with tribromoethanol, and liver, kidneys, and spleens were collected fresh. Half of the tissues were flash frozen on dry ice and stored at -80°C for protein analysis and the others were placed in RNAlater at 4°C for mRNA analysis.

### Automatic western detection of mutant and wt huntingtin

For analysis of HTT protein expression in mouse livers WES by ProteinSimple was used. 2 mm-size tissue punches were homogenized at 75 μl of buffer (10 mM HEPES pH 7.2, 250 mM sucrose, 1 mM EDTA buffer with protease and phosphatase inhibitors) and stored at −80°C. Protein amount was determined using a Bradford Assay. Samples were diluted in 0.1 × sample buffer (ProteinSimple) to ∼0.2 μg/μl. Anti-HTT antibody (36) was diluted 1:50 in antibody diluent (ProteinSimple). This antibody detects both mutant and wt huntingtin protein, which are separated by weight due to the expansion of the CAG repeat in mutant (∼103 copies) versus wt (∼7 copies) proteins. The anti-vinculin (Sigma, V9131) antibody was diluted 1:3000 in diluent and used as a normalization control. Assay was performed as described by the manufacture instructions using the 66–440 kDa plate (SM-W008).

## RESULTS

### Synthesis of ^i^*E*-VP-dimer phosphoramidites

The three features defining the utility of synthetic oligonucleotide modifications are stabilization, efficacy, and specificity. Sugar and backbone modifications need to be used in combination to achieve these optimal properties. Therefore, we set out to optimize the synthetic pathways enabling oligonucleotide backbone ^i^*E*-VP incorporation in the context of the two most commonly-used sugar modifications: 2′-OMe and 2′-F.

**Scheme 1.**
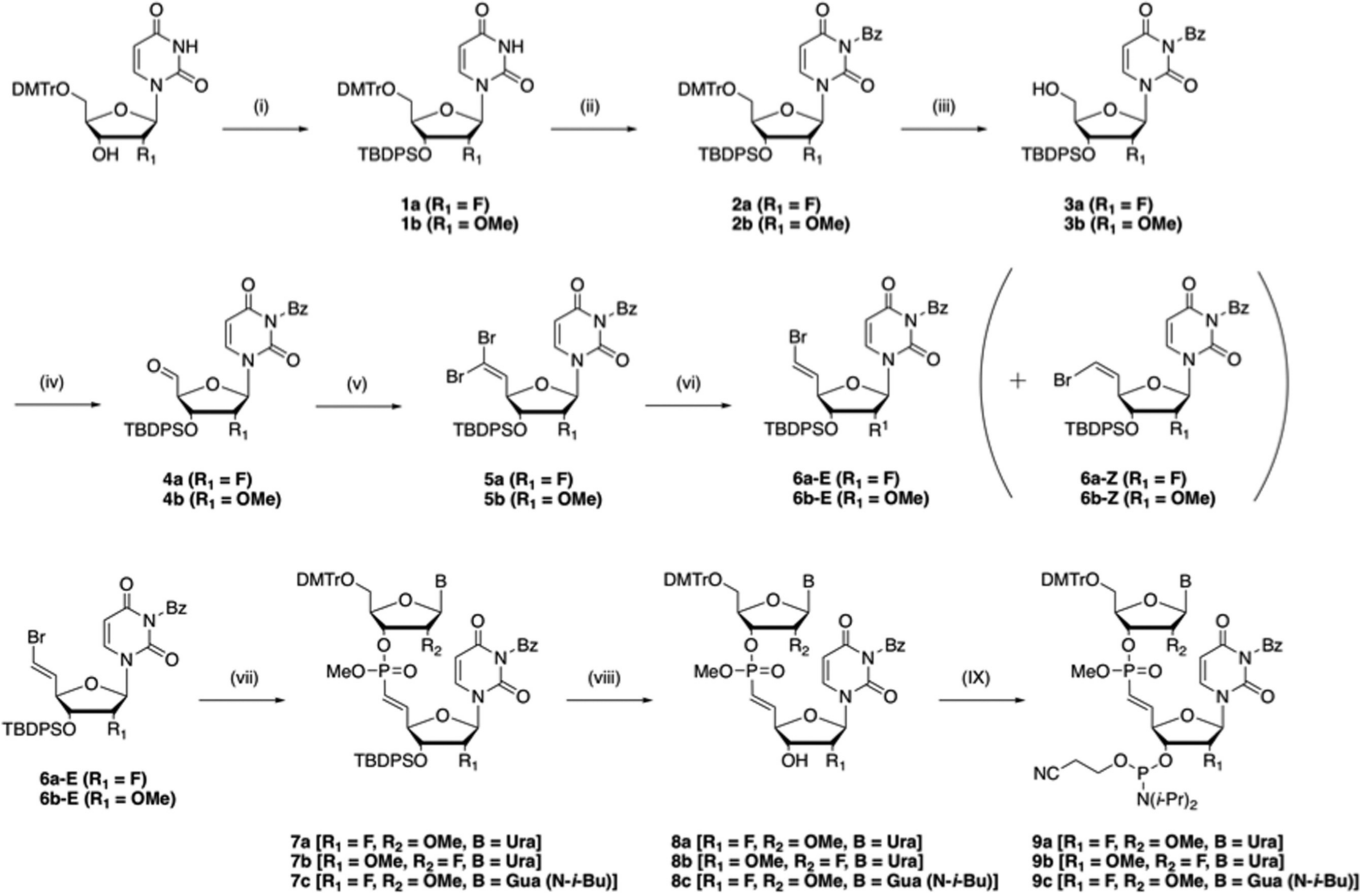
Synthesis of ^i^*E*-VP-linked dimer phosphoramidites (**9a**, **9b**, and **9c**). *Reagents and conditions*: (i) TBDPSCl, Imidazole/DMF, rt, overnight, **1a**: 99%, **1b**: 95%; (ii) BzCl, DIPEA/CH_2_Cl_2_, rt, 4 h, **2a**: 78%, **2b**: N.D.^a^; (iii) 3% trichloroacetic acid, triethylsilane/CH_2_Cl_2,_ rt, 1 h, **3a**: 91%, **3b**; 63% (2 steps); (iv) IBX/CH_3_CN, 85°C, 2 h; (v) CBr_4_, PPh_3_/CH_2_Cl_2_, 0°C then rt, 2 h, **5a**: 52% in 2 steps, **5b**: 56% in 2 steps; (vi) dimethylphosphite, triethylamine/DMF, rt, overnight, **6a-E**: 52%, **6b-E**: 55%, **6a-Z**: 15%, **6b-Z**: 22%; (vii) 5′-*O*-DMTr-uridine-3′-*H*-phosphonate methyl ester (2′-OMe: **S2a** or 2′-F: **S2b**) or 5′-*O*-DMTr-2-*N*-(*iso*-butyryl)- 2′-OMe-guanosine-3′-*H*-phosphonate methyl ester (**S2c)**, Pd(OAc)_2_, dppf, propylene oxide/THF, 70°C or 75°C, 4 h-overnight, **7a**: 57%, **7b**: 64%, **7c**: 30%; (viii) 0.1 M TBAF/THF, rt, 0.5 h, **8a**: 94%, **8b**: 47%, **8c**: 85%; (IX) 2-cyanoethyl *N*,*N*-diisopropylchlorophosphoramidite, DIPEA/CH_2_Cl_2_, 0°C then rt, 0.5 h, **9a**: 80%, **9b**: 68%, **9c**: 57%; ^a^Not determined (see materials and methods).

Dimer nucleotide phosphoramidite synthesis of 2′-OMe-Ur-(^i^*E*-VP)-2′-F-Ur (**9a**), 2′-F-Ur-(^i^*E*-VP)-2′-OMe-Ur (**9b**), and 2′-OMe-G-(^i^*E*-VP)-2′-F-Ur (**9c**) was carried out using the following synthetic approach (Scheme 1). Synthesis began with commercially-available 5′-*O*-DMTr protected nucleosides (purchased from Hongene Biotechnology Co., Ltd.), followed by protection of 3′-OH with TBDPS and 3-N with benzoyl (Bz) groups, and eventual 5′-O-DMTr group deprotection. After oxidation of 5′-OH by 2-iodoxybenzoic acid (IBX) converting to aldehyde (**4a** and **4b**), Wittig olefination was carried out to obtain dibromo olefin compounds (**5a** and **5b**) (38). Use of a 3-N-Bz protecting group was essential for the efficiency of this Wittig olefination. Without this protective group, the reaction mixture yielded a highly polar compound that could not be converted to target material even after prolonged reaction time and heating (data not shown). Our result differs from a previous report demonstrating successful synthesis of a DNA variant, ^i^*E*-VP-containing dimer (dT-^i^*E*-VP-dT), without nucleobase protection (38). This difference is likely due to our use of 2′-OMe and 2′-F sugar modifications, both of which are essential for generating functionally-active siRNAs (39,40). Use of published protocols (no nucleobase protection) for the synthesis of 2′-OMe- and 2′-F-containing ^i^*E*-VP dimers resulted in extremely low yields, likely due to the C3′-endo sugar conformation of 2′-OMe and 2′-F nucleosides, of which the 5′-aldehyde residue may orient proximal to an unprotected uracil base, inducing nucleobase-involving side reactions.

Hirao reduction of the dibromo compound produced (*E*)-bromo olefin nucleoside (**6a-E** and **6b-E**) as a major product and (*Z*)-vinyl olefin nucleoside (**6a-Z** and **6b-Z**) as a minor product (38,41). Purification of both stereo-isomers was carried out by repeated silica gel column chromatography (Supplementary Figures S1, S2). The palladium-mediated cross-coupling condensation between (*E*)-bromo olefin nucleoside (**6a-E** and **6b-E)** and nucleoside-*H*-phosphonate methyl ester (**S2a**, **S2b**, or **S2c)** yielded a P-OMe-protected ^i^*E*-VP-linked nucleotide dimer (38). After 3′-O-deprotection and phosphitylation, ^i^*E*-VP linked dimer phosphoramidites (**9a**, **9b**, and **9c**) were synthesized without deprotection of the 3-N-Bz group, which was eventually removed in the final step: base treatment of oligonucleotides. (See supplementary data for details about oligonucleotide synthesis).

#### Oligonucleotide synthesis

All oligonucleotides used were synthesized on MerMade 12 (BioAutomation) synthesizer in 1 μmol scale using universal UNYLinker support or cholesterol 3′-lcaa CPG (ChemGenes). All commercially available 2′-modified nucleoside phosphoramidites and ^i^*E*-VP-dimer phosphoramidites were coupled under standard conditions on solid support using 5-(benzylthio)-1-*H*-tetrazole (BTT) as an activator (see supporting information for details). The efficiencies, equivalent use, and time of reaction for dimer phosphoramidites were similar to monomeric assembly, indicating that this strategy can be used to synthesize ^i^*E*-VP-modified oligonucleotides with similar yields and efficiency (Supplementary Figures S3, S4). The obtained ^i^*E*-VP-modified siRNAs were first treated with TMSBr to deprotect the methyl group on ^i^*E*-VP, followed by standard base deprotection to release oligonucleotides from the solid support (34). Control siRNAs with identical sequence and chemical modification pattern, but without ^i^*E*-VP modification, were prepared to enable direct comparison of ^i^*E*-VP incorporation on efficacy independent of sequence and modification pattern (Supplementary Tables S1–S3).

#### ^i^ *E*-VP incorporation can positively or negatively impact oligonucleotide-enzyme interactions

**Figure 2.**
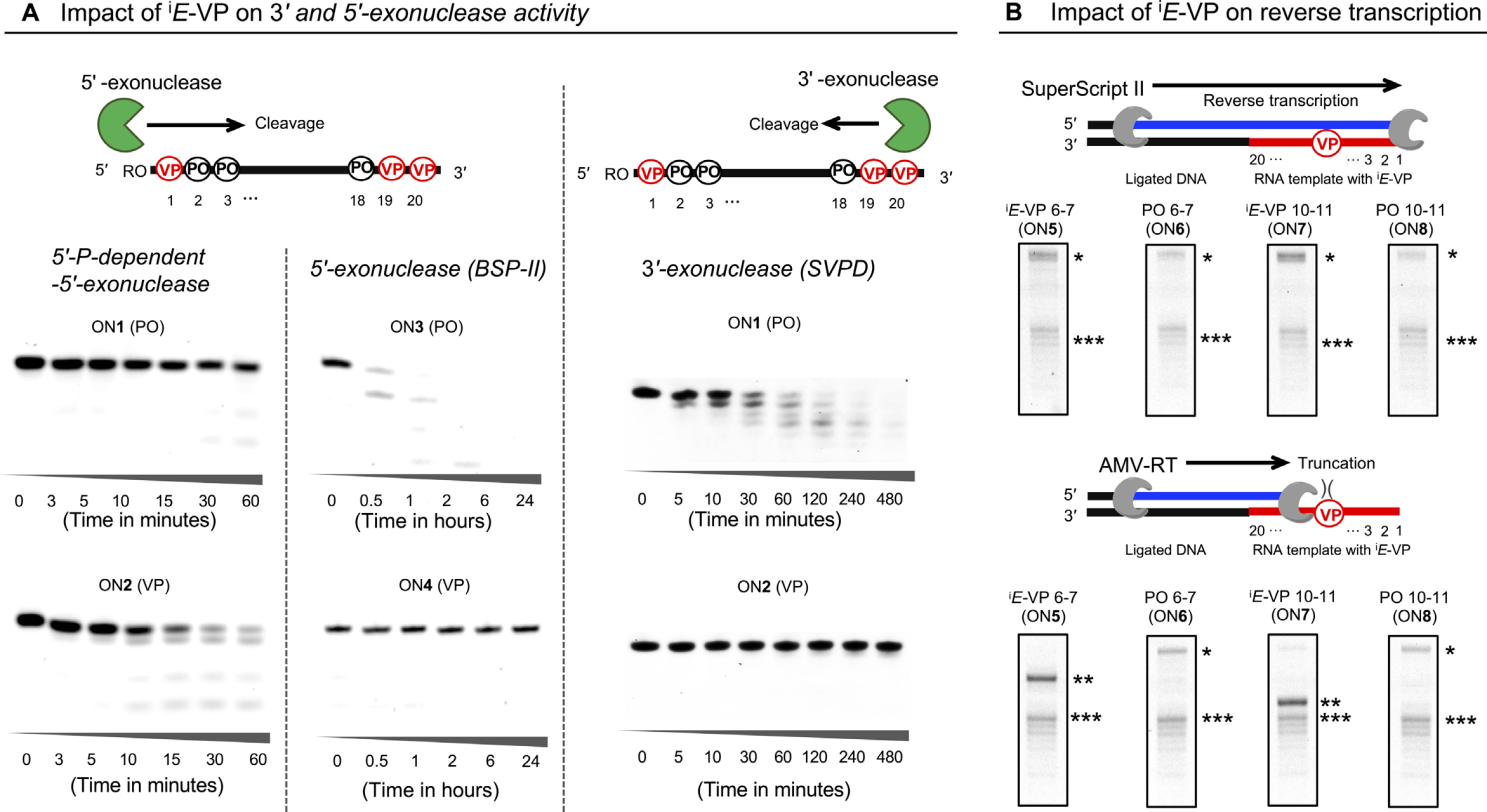
Impact of incorporating ^i^*E*-VP into oligonucleotide backbone on nuclease and reverse transcriptase activity. (**A**) Nuclease digestion test using P-O3′ bond-cleaving 5′-phosphate (P)-dependent 5′-exonuclease (Terminator™, 8.5 mU/μl), P-O5′-cleaving 5′-exonuclease (BSP II, 0.25 U/ml), and P-O3′-bond cleaving snake venom phosphodiesterase I (SVPD, 4 mU/ml). R = PO_3_^–^ for **ON1** and **2**, R = H for **ON3** and **4**. (**B**) Reverse transcription by Superscript II and AMV RT using ^i^*E*-VP-modified RNA template. *Full-length, extended product. **Truncated product stopped at position of ^i^*E*-VP (6–7 or 10–11) on templates. ***Minor truncated products stopped at phosphorothioate modification on templates.

^i^ *E*-VP modification at 3′-terminal backbone linkages provided significant stabilization against the P-O3′-cleaving 3′-exonuclease, snake venom phosphodiesterase I (SVPD). At conditions tested, >90% of the control oligonucleotide (**ON1**) was degraded within an hour, while ^i^*E*-VP-modified compound (**ON2**) showed no detectable degradation, even after 8 h, suggesting the ^i^*E*-VP structure is highly incompatible with the SVPD active site (Figure 2A).

To evaluate the impact of ^i^*E*-VP incorporation on oligonucleotide interactions with nucleases, we synthesized oligonucleotide variants with or without ^i^*E*-VP incorporated at 5′ and 3′-terminal backbone linkages (**ON1**-**4**, Supplementary Table S3). ON1 and 2 where chemically phosphorylated to meet substrate requirements for 5′-phosphate-dependent 5′-exonuclease. We also sought to evaluate how stereo constraint of the backbone impacts the ability of reverse transcriptases (RTs) to synthesize complementary DNA because oligonucleotides can serve as a template for polymerase reactions. To achieve this, we synthesized template oligonucleotide variants with or without ^i^*E*-VP incorporated between nucleotide positions 6–7 (**ON5** and **6**, respectively) or 10–11 (**ON7** and **8**, respectively), which positions located at middle positions in ^i^*E*-VP modified template oligonucleotide (Supplementary Table S3). For synthesis of all oligonucleotides, an automated RNA synthesizer was used to assemble phosphoramidites on a solid support (see Materials and Methods). After incubating with a variety of exonucleases, full-length oligonucleotide and degradation products were resolved on denaturing gels and visualized by SYBR-Gold. Interestingly, ^i^*E*-VP-containing **ON2** behaved as a better substrate for 5′-P-dependent 5′-exonuclease compared to an oligonucleotide of identical sequence and sugar modification pattern, but with a canonical (non-modified) phosphodiester linkage (**ON1**) (Figure 2A). At the conditions tested, >70% of ^i^*E*-VP-containing **ON2** was degraded at 60 min, while the majority (>50%) of **ON1** remained intact. This result is likely due to the locked torsion angle of ^i^*E*-VP inducing a better fit in the 5′-P-dependent 5′-exonuclease active site (50). By contrast, ^i^*E*-VP incorporation at the 5′-terminal backbone linkage provided full stabilization against the 5′-exonuclease BSP (bovine spleen phosphodiesterase II). This is because BSP cleaves at P-O5′ of the phosphodiester linkage, which is replaced with the uncleavable P-C(sp^2^) bond in the ^i^*E*-VP structure (**ON4**, Figure 1B and 2A).

As a positive control for all nuclease stability experiments, we synthesized PS-containing oligonucleotides. As expected, PS linkages exhibited high resistance against all exonucleases tested (Supplementary Figures S7–S9). However, iE-VP’s protection against SVPD was relatively less than that observed with PS. This became more evident when stabilization effects were evaluated at exaggerated SVPD concentrations and assayed using ^i^*E*-VP/PS mixed sequences (Supplementary Figures S7 and S8). Altogether, our results from the exonuclease digestion assays demonstrate that backbone stereo-constraining can either disturb or enhance activity of exonucleases in enzyme-specific manner.

To investigate the impact of the ^i^*E*-VP modification on primer extension by RTs, ^i^*E*-VP-containing oligonucleotides (**^VP^G6** and **^VP^G10** for **ON5** and **6**, respectively) and control oligonucleotides (**^Ctrl^G4** and **^Ctrl^G1**, for **ON7** and **8**, respectively) were ligated to a DNA adapter and hybridized to a 5′-FAM-labeled primer (Figure 2B). When Avian Myeloblastosis Virus reverse transcriptase (AMV-RT) was used, primer extension was quantitatively terminated upon encountering the ^i^*E*-VP-modified inter-nucleotide position. Truncated band position was shifted between **ON5** (^i^*E*-VP modification between nucleotides 6–7) and **ON7** (^i^*E*-VP between nucleotides 10–11) by four nucleotides, confirming specificity of the observed phenomena. Our results suggest that AMV-RT cannot accommodate the stereo-constrained backbone for reverse transcription. In contrast, Superscript II (a genetically-engineered Moloney Murine Leukemia Virus (MMLV) RT variant with better processivity and tolerance for modifications) efficiently utilized ^i^*E*-VP-containing templates with no termination products observed at the site of ^i^*E*-VP incorporation (Figure 2B) (51). Collectively, these findings demonstrate that, though it is a minimal structural change, structure constraint of the ^i^*E*-VP backbone induces huge impact on enzymatic polymerization reaction in enzyme specific manner.

#### Design and synthesis of chemically-modified siRNAs

To evaluate the impact of ^i^*E*-VP incorporation on siRNA RISC forming proteins, we designed and synthesized a panel of modified siRNA guide strands, in which individual positions of the backbone were modified with ^i^*E*-VP (**^VP^G1**-**20**), as follows:

#### siRNA conjugate and chemical modification pattern design

**Figure 3.**
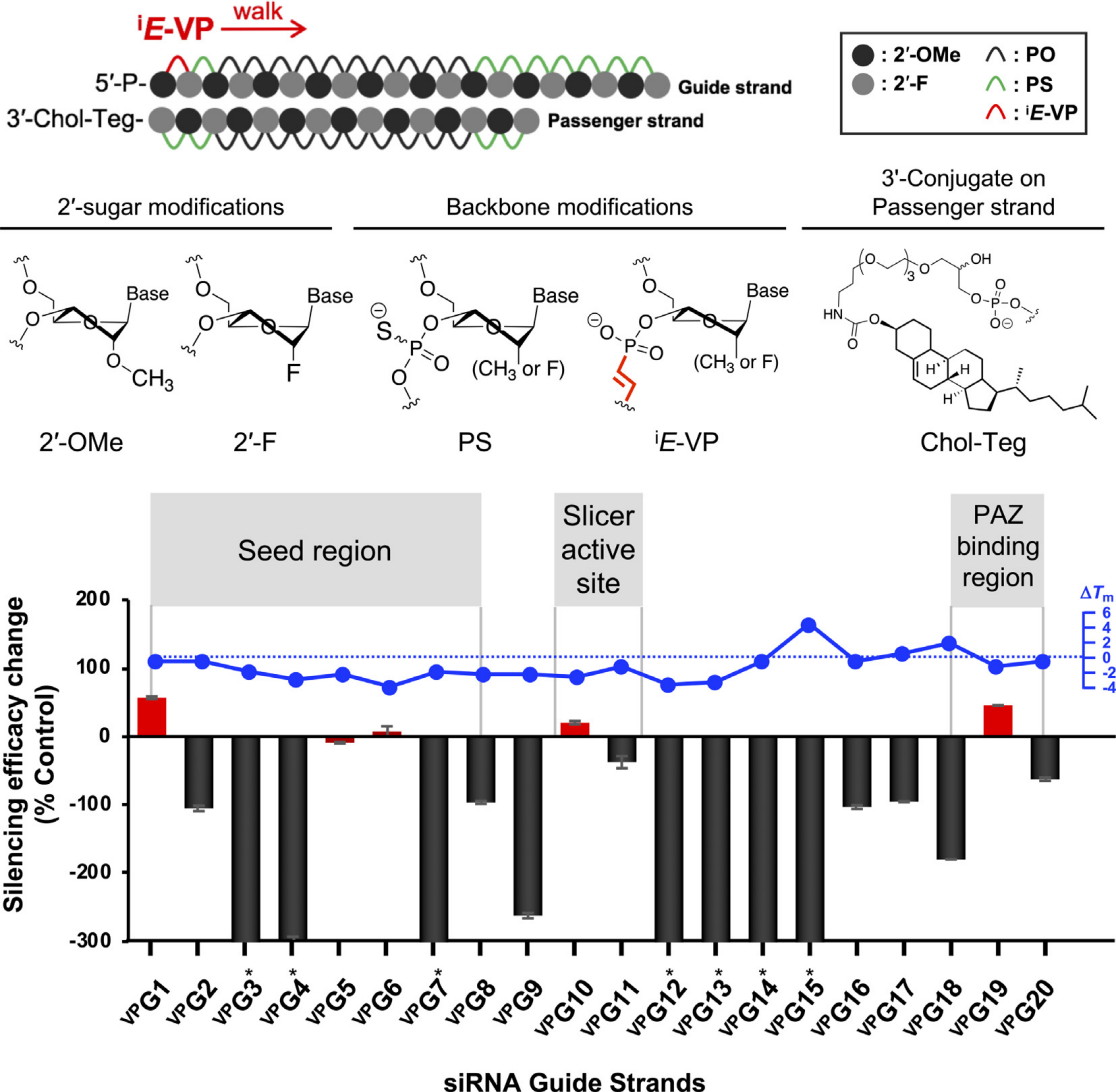
Position-dependent impact of ^i^*E*-VP backbone on siRNA efficacy and impact of ^i^*E*-VP backbone on thermal stability of siRNA duplex. Silencing efficacy change was defined by eq 1. Red bar graphs indicate siRNAs having neutral or enhanced silencing efficacy compared to control siRNAs. Black bar graphs indicate ^i^*E*-VP-modified siRNAs showed lower efficacy than that of control siRNAs. See Table 1 and supporting information for sequences, and Supplementary Table S7 for IC_50_ values used for the calculation of efficacy changes. *Efficacy change that exceed –300% was set as an artificial difference to -300% in the graph. Comparative *T*_m_ of siRNA duplex containing ^i^*E*-VP and corresponding control siRNA duplex was measured in 10 mM Sodium phosphate buffer (pH 7.0) containing 100 mM NaCl and 0.1 mM EDTA. Δ*T*_m_ = *T*_m_^VP^ – *T*_m_^Ctrl^. *T*_m_ values were determined in triplicate experiments.

Because conjugate-mediated delivery is the dominant strategy for fully chemically modified siRNA delivery in the clinic (8–10,37), we elected to use cholesterol-conjugated fully chemically modified asymmetric siRNA in our studies (Figure 3) (48). The chemical modification pattern consisted of alternating 2′-OMe and 2′-F sugar modifications with terminal PS stabilization for both guide and passenger strands, a configuration that is widely utilized for conjugated siRNAs (1,8,40,42). These compounds are readily internalized by many cell types without requiring formulation, making functional *in vitro* evaluation straightforward (30). When a similar chemical modification pattern was applied to siRNAs conjugated to GalNAc or hydrophobic moieties, robust *in vivo* efficacy was achieved (29,37, 43–47).

#### siRNA sequence design

We selected previously-validated, highly-potent sequences targeting the huntingtin (HTT) gene (48). The panel of these siRNAs contain a uridine-uridine dimeric sequence at each nucleotide position on the guide strand (Table 1).

**Table 1.** Sequence of ^i^*E*-VP modified guide strands and control guide strands

^VP^Guide strands	Sequence (5′ → 3′)^a^	Calcd. mass [M-H]^–^	Observed mass	Correspond Passenger strands^b^	^VP^siRNA #	Correspond ^Ctrl^siRNA^c^ #
^VP^G1	P-U**vp**U#AAUCUCUUUAC#U#G#A#U#A#U#A	6599.4	6599.7	P1	^VP^D1	^Ctrl^D1
^VP^G2	P-U#U**vp**UUUAAAUCCUG#A#G#A#A#G#A#A	6724.5	6724.8	P2	^VP^D2	^Ctrl^D2
^VP^G3	P-U#U#U**vp**UUAAAUCCUG#A#G#A#A#G#A#A	6739.6	6740.7	P2	^VP^D3	^Ctrl^D2
^VP^G4	P-U#U#UU**vp**UAAAUCCUG#A#G#A#A#G#A#A	6740.6	6740.7	P2	^VP^D4	^Ctrl^D2
^VP^G5	P-U#C#UCU**vp**UUACUGAU#A#U#A#A#U#U#A	6614.4	6615.7	P3	^VP^D5	^Ctrl^D3
^VP^G6	P-U#A#UGUU**vp**UUCACAU#A#U#U#G#U#C#A	6631.4	6631.6	P4	^VP^D6	^Ctrl^D4
^VP^G7	P-U#G#AAUGU**vp**UCACGC#A#G#U#G#G#G#C	6802.6	6802.8	P5	^VP^D7	^Ctrl^D5
^VP^G8	P-U#A#UCAGCU**vp**UUUCC#A#G#G#G#U#C#G	6700.5	6700.7	P6	^VP^D8	^Ctrl^D6
^VP^G9	P-U#U#AAUCUCU**vp**UUAC#U#G#A#U#A#U#A	6615.4	6615.7	P1	^VP^D9	^Ctrl^D1
^VP^G10	P-U#U#AAUCUCUU**vp**UAC#U#G#A#U#A#U#A	6615.4	6615.7	P1	^VP^D10	^Ctrl^D1
^VP^G11	P-U#U#AACGUCAGU**vp**UC#A#U#A#A#A#C#C	6675.5	6675.7	P7	^VP^D11	^Ctrl^D7
^VP^G12	P-U#C#CACUAUGUUU**vp**U#C#A#C#A#U#A#U	6590.4	6590.7	P8	^VP^D12	^Ctrl^D8
^VP^G13	P-U#C#CAAAUACUGGU**vp**U#G#U#C#G#G#U	6708.5	6708.7	P9	^VP^D13	^Ctrl^D9
^VP^G14	P-U#C#CGGUCACAACA#U**vp**U#G#U#G#G#U	6707.5	6707.7	P10	^VP^D14	^Ctrl^D10
^VP^G15	P-U#A#UGUUUUCACAU#A#U**vp**U#G#U#C#A	6615.4	6615.7	P14	^VP^D15	^Ctrl^D4
^VP^G16	P-U#U#UGGUAGCUGAA#A#G#U**vp**U#C#U#U	6710.4	6710.7	P11	^VP^D16	^Ctrl^D11
^VP^G17	P-U#U#AAUCUCUUUAC#U#G#A#U**vp**U#U#A	6576.3	6576.6	P1	^VP^D17	^Ctrl^D12
^VP^G18	P-U#C#UCUUUACUGAU#A#U#A#A#U**vp**U#A	6599.4	6599.7	P3	^VP^D18	^Ctrl^D3
^VP^G19	P-U#U#AAUCUCUUUAC#U#G#A#U#A#U**vp**U	6576.3	6576.7	P1	^VP^D19	^Ctrl^D13
^VP^G20	P-U**vp**U#AAUCUCUUUAC#U#G#A#U**vp**U#U**vp**U	6513.2	6513.7	P1	^VP^D20	^Ctrl^D14

^a^Uppercase and underlined uppercase represent 2′-OMe and 2′-F, respectively. 5′-end phosphate is represented as ‘P’. Inter-nucleotide phosphorothioate and (*E*)-vinylphosphonate linkages are indicated by ‘#’ and **vp**, respectively.

^b^3′-end tetraethylene glycol (Teg) -linked cholesterol (Chol) conjugated 15 mer passenger strands (**P1**–**P14**) to prepare ^VP^siRNAs (**^VP^D1**–**^VP^D20**) consists with (i) complementary sequence through 5′-end to 15th nucleotide of guide strands and (ii) with fully chemical modification pattern: 5′-N#N#NNNNNNNNNNN#N#N-Teg-Chol-3′ (N and N represent 2′-OMe and 2′-F, respectively. See supporting information for each of passenger strand sequences).

^c^Comparative control siRNA duplexes corresponding to each ^VP^siRNA. Sequences shown in supporting information.

#### Synthesis of siRNA panel

The antisense or ‘guide’ strand of the siRNA duplex is more sensitive to modifications than the sense or ‘passenger’ strand (49). Therefore, we sought to evaluate the impact of incorporating ^i^*E*-VP into the guide strand. We synthesized different guide strands with a single incorporation of ^i^*E*-VP at a specific inter-nucleotide position (**^vp^G1**-**19**) or with multiple terminal ^i^*E*-VP insertions (**^vp^G20**), which were then used to create our screening panel (Table 1). Control guide strands without ^i^*E*-VP consisting with identical chemical modifications were also synthesized (Supplementary Table S1).

#### Position-dependent impact of ^i^*E*-VP incorporation on siRNA efficacy

Using our full panel of ^i^*E*-VP-modified siRNAs (**^VP^D1**–**20**) and matched controls (**^Ctrl^D1**–**14**), we systematically evaluated the position-dependent impact of ^i^*E*-VP in guide strands on siRNA efficacy (Table 1). Specifically, we treated HeLa cells with our siRNA library and measured target *HTT* gene expression by QuantiGene assay after 72 h (see Materials and Methods). We tested compound efficacy at 7 concentrations to calculate IC_50_ values (Supplementary Table S7). IC_50_ of ^i^*E*-VP-modified siRNAs (**^VP^D1**–**20**) and control siRNAs (**^Ctrl^D1**–**14**) of identical sequence were used to define ‘efficacy change’ based on the following equation:

(1) $$\begin{equation*}{\rm{Efficacy\, change}} =100 \times \left( {^{{\rm{Ctrl}}}{\rm{I}}{{\rm{C}}_{{\rm{50}}}}{-^{{\rm{VP}}}}{\rm{I}}{{\rm{C}}_{{\rm{50}}}}} \right)\,{{\rm{/}}^{{\rm{Ctrl}}}}{\rm{I}}{{\rm{C}}_{{\rm{50}}}}\end{equation*}$$

As summarized in Figure 3, the structurally-constraining ^i^*E*-VP backbone either positively or negatively affected RISC efficacy in a position-specific manner. Incorporating ^i^*E*-VP was well tolerated at guide termini positions 1–2 (**^vp^D1**) and 19–20 (**^vp^D19**), and enhanced RISC activity. ^i^*E*-VP was also well tolerated at: (i) ‘seed region’ positions 5–6 (**^vp^D5**) and 6–7 (**^vp^D6**), which are essential for RISC recognition of target mRNA; and (ii) position 10–11 (**^vp^D10**), which is next to the mRNA cleavage site. ^i^*E*-VP incorporation at all other inter-nucleotide positions resulted in decreased RISC activity.

To exclude the possibility that observed differences in efficacy between ^i^*E*-VP-containing and control siRNA were due to a change in siRNA duplex thermal stability (*T*_m_), we measured the impact of ^i^*E*-VP on the *T*_m_ of **^VP^D1**–**20** (Supplementary Figure S5). We observed very limited contribution of *T*_m_ [Δ*T*_m_ = *T*_m_ (^VP^siRNA) – *T*_m_ (^Ctrl^siRNA) = +4.8 to –3.7°C)], and no correlation with functional outcomes (Figure 3 and S6). This suggests that changes in the thermodynamic profile of siRNA duplexes do not significantly contribute to functional differences (Figure 3).

#### Stereo-constraint of the backbone with ^i^*E*-VP enhances SNP-based discrimination

Structural limitation is a major driving force of the specificity in inter-molecular recognition. Our finding that structurally-constraining ^i^*E*-VP is well tolerated at the end of the seed region prompted us to evaluate whether structurally-constraining the backbone could enhance specificity of the RISC-target mRNA complex, particularly in the context of mismatch-based target sequence discrimination. Mismatch-based discrimination by siRNAs is a promising therapeutic strategy for allele-specific silencing (52,53). For instance, in Huntington's disease (HD), the ability to target heterozygous single nucleotide polymorphisms (SNPs) and selectively silence only the mutant allele is clinically preferred (54). We recently developed and optimized SNP-specific siRNA targeting a common HD heterozygous SNP (rs362273) (Conroy, F., Miller, R.A. *et al.* 2021, in revision). Although this compound is potent and shows reasonable SNP discrimination (∼60X selectivity towards single mismatch), there is still detectable off-target activity *in vitro*. The injection of this compound in the CNS supports efficient discrimination in most of the regions of the brain but shows a trend towards reduction of the wild-type allele silencing *in vivo*, particularly in regions with high levels of oligonucleotide accumulation. Both sugar and nucleobase modifications of antisense oligonucleotides were explored as an approach to improve SNP recognition (68), however, the impact of structural constraint of internucleotide backbone of siRNAs on their SNP recognition ability has not been investigated. We hypothesized that a locked ^i^*E*-VP backbone in the context of a mismatched base pair could alter local RISC structure to induce non-functional RNA-Ago2 accommodation and limit off-target recognition and silencing.

**Figure 4.**
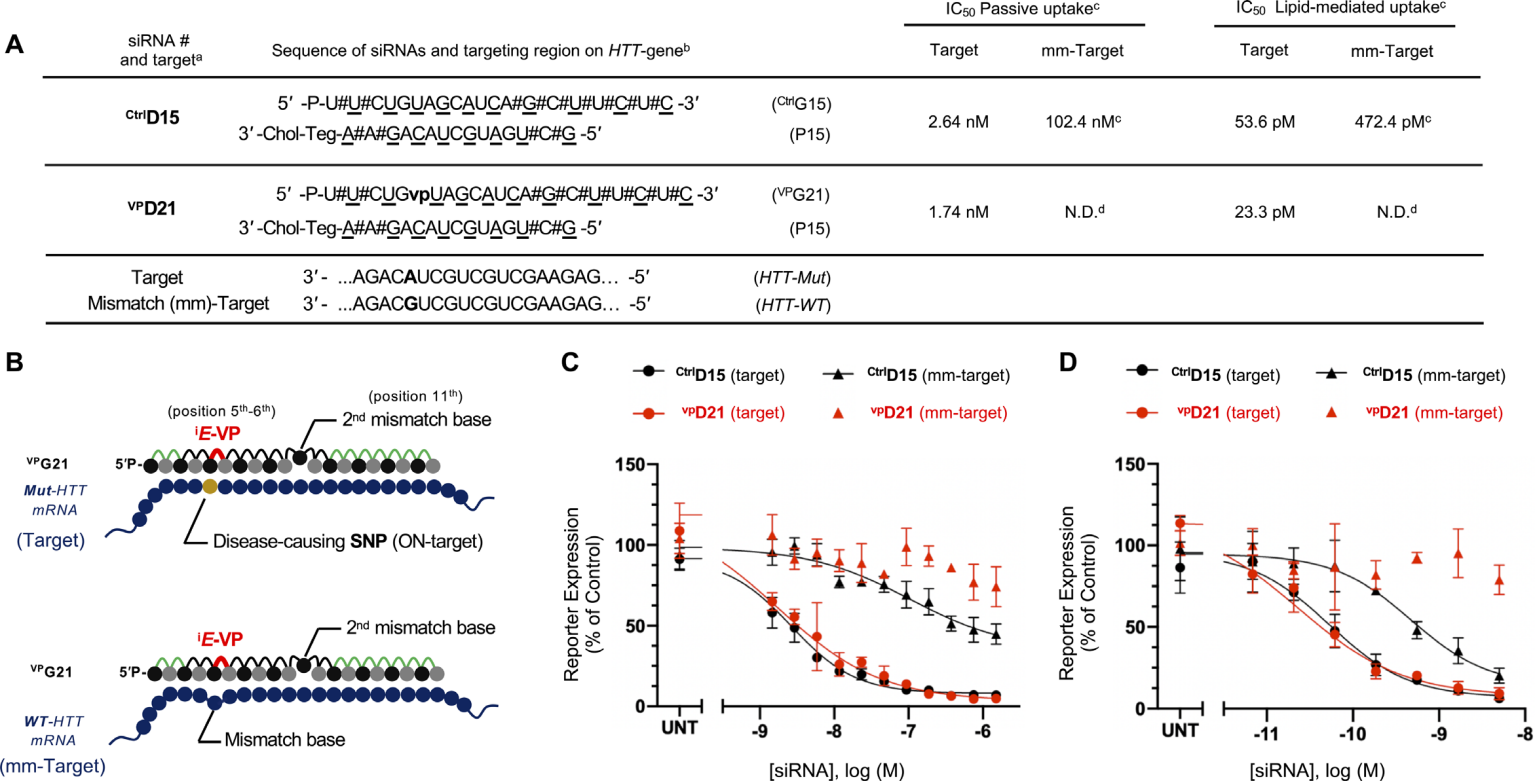
SNPs discrimination properties of ^i^*E*-VP-modified siRNA. (**A**) Sequences of siRNAs (**^Ctrl^D15** and **^VP^D21**), target mRNAs with or without single mismatch (mm) base (target and mm-target, respectively), and IC_50_ values. (**B**) Conceptual scheme of ON and OFF-target silencing of **^VP^D21**. (**C**) Passive uptake of siRNA and dose response of **^VP^D21** and **^Ctrl^D15** in the presence of target or mm-target mRNAs. (**D**) Lipid-mediated uptake and dose response of **^VP^D21** and **^Ctrl^D15** in the presence of target or mm-target mRNAs. ^a^**^Ctrl^D15** and **^VP^D21** consist of **^Ctrl^G15**/**P15** and **^VP^G21**/**P15**, respectively; ^b^Uppercase and underlined uppercase represent 2′-OMe and 2′-F, respectively. 5′-end phosphate is represented as ‘5′-P’. Inter-nucleotide phosphorothioate and (*E*)-vinylphosphonate linkages are indicated by ‘#’ and **vp**, respectively. Passenger strands have 3′-end tetraethylene glycol (Teg)-linked cholesterol (Chol) conjugate. ^c^IC_50_ was calculated based on duplicate experiments. ^d^N.D. = Not determined.

To test this hypothesis, we synthesized a panel of siRNAs targeting HTT SNP rs362273. In these siRNA guide strands, nucleotide position 6 is complementary to the SNP site and position 11 is a secondary mismatch introduced to enhance single mismatch discrimination (Conroy, F., Miller, R.A. *et al.* 2021, in revision). Guide strands were synthesized with stereo-constraining ^i^*E*-VP located 5′-upstream to the primary SNP mismatch (**^VP^G21**-**23**) along with matched controls (**^Ctrl^G15**-**17**) (Supplementary Tables S4, S5). To enable synthesis of these guide strands, we further prepared 2′-OMe-G-(^i^*E*-VP)-2′-F-Ur dimer phosphoramidite (**9c**) using synthetic intermediate **6a-E** (Scheme 1). The sequence and chemical modification patterns of SNP-targeting siRNAs (**^vp^D21**-**23**), corresponding control siRNAs (**^Ctrl^D15**-**17**), and Passenger strands (P15-17) are shown in Supplementary Tables S4-S6. We evaluated siRNA efficacy using a luciferase-based reporter system with a single nucleotide difference in the target region (‘target’ mutant vs ‘mismatch (mm)-target’ wild-type) at seven concentrations to generate IC_50_ values. Incorporating ^i^*E*-VP next to the primary SNP site did not negatively impact ‘on-target’ activity – IC_50_ values of **^Ctrl^D15** and **^VP^D21** were 2.64 nM and 1.74 nM, respectively (Figure 4A). This RISC compatibility of ^i^*E*-VP at position 5–6 was consistent with our efficacy screening results in HeLa cells (Figure 3), despite the different sequence contexts (^i^*E*-VP backbone between 2′-OMe-U/2′-F-U dimer versus 2′-OMe-G/2′-F-U dimer at position 5–6). Importantly, **^VP^D21** completely lost the ability to silence mm-target mRNA at all concentrations tested (Figure 4C for passive uptake and 4D for lipid mediated uptake). Similar results were generated in the context of different secondary mismatches (**^VP^D22** and **^VP^D23**, Supplementary Figure S10), suggesting that stereo-constraining the backbone next to a SNP can be used as a chemical strategy to enhance mismatch discrimination by siRNAs.

### Stereo-constraint of the backbone with ^i^*E*-VP enhances SNP-based discrimination *in vivo* in BAC-HD mice

Incorporation of ^i^*E*-VP next to the mismatch site significantly enhances discrimination between mutant and wt allele, and, most importantly, induces no negative impact on on-target activity. The two-mismatch containing compound shows reasonable discrimination *in vivo* in mice brain (Conroy, F., Miller, R.A. *et al.* 2021, in revision). In CNS delivery, the relative accumulation of compounds in tissues is low. To test the hypothesis that incorporation of ^i^*E*-VP further enhances discrimination *in vivo*, we needed to select the system where high accumulation of oligonucleotides in tissues can be achieved. Trivalent GalNAc conjugation (30,31,40,44,45) enables robust delivery to liver with high degree of oligonucleotides accumulation and represent a great system to push the limits of discrimination *in vivo*. The model compound we use in this study targets HD heterozygous SNP (rs362273). This SNP is present in the BAC-HD 103 human transgene, while the mouse gene carries an opposite allele in this location. The mutant HTT protein contains ∼ 103 CAG repeats in the exon one while mouse wt variant has only ∼ 7 CAGs. Thus, the mutant and wt HTT protein differ in size and can be differentially detected by the western blot.

To evaluate single mismatched SNP discrimination ability of ^i^*E*-VP modified siRNA in liver, trivalent-GalNAc conjugated siRNAs that contains ^i^*E*-VP backbone at position 5 of internucleotide, secondary mismatch base at position 11, and 5′-termianl (*E*)-VP in the guide strand was prepared (**^VP^D24**). For the control GalNAc-siRNA (**^Ctrl^D18**), guide strand without ^i^*E*-VP was also prepared (Supplementary Table S8).

**Figure 5.**
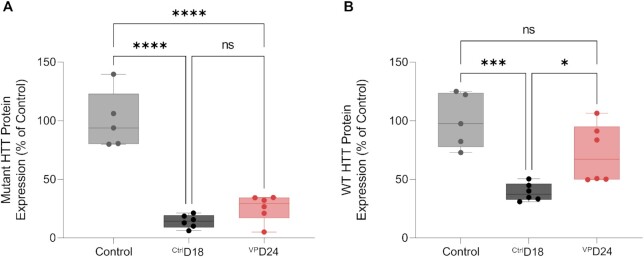
Introduction of ^i^*E*-VP next to the SNP site enhances discrimination based on a single nucleotide difference in the target site. GalNAc conjugated siRNAs with (**^VP^D24**) and without (**^Ctrl^D18)** the introduction of the ^i^*E*-VP next to the SNP site (**^VP^D24** and **^Ctrl^D18**, respectively), were injected at 10 mg/kg dose level SC at ∼12 weeks BAC-HD mice. The level of mutant and WT huntingtin proteins was evaluated using western blot (Protein Simple) in mice livers at two weeks post injection. N-5,6, one-way Anova with Bonferroni correction for multiple comparisons. (**A**) Level of mutant protein expression. Both compounds induce robust silencing. (**B**) Level of WT protein expression. **^Ctrl^D18** induces WT protein silencing which is significantly reduced with **^VP^D24** compound.

BAC-HD mice were treated with **^Ctrl^D18** and **^VP^D24** and level of HTT mutant and wt expression were evaluated at two weeks post injection relative to control. While both **^Ctrl^D18** and **^VP^D24** compounds shows efficient silencing of the mutant protein (>80%, *P* < 0.0001), the level of wt (non-target) expression was affected differently between two variants. The **^Ctrl^D18** induce partial ∼60% silencing of the wt, while the level of silencing induced by **^VP^D24** did not reach a significance threshold (Figure 5). This data indicates that stereo-constraining of the backbone next to the SNP site allows for significant increase in the degree of SNP discrimination *in vivo*, even in the tissue where siRNAs accumulated efficiently.

## DISCUSSION

Oligonucleotide structure is responsible for highly-specific interactions with a wide range of biological partners. The vast majority of nucleic acid-protein interactions involve individual backbone contacts (55), where the structural configuration and the angle of the backbone are significant contributors to the efficiency and specificity of molecular recognition. Here, we describe the synthesis and functional characterization of a novel backbone modification, ^i^*E*-VP, where the bridging oxygen is substituted with a carbon and locked torsion angle at 180^o^. We find that stereo-constraining the backbone can decrease or enhance efficiency of enzymatic recognition. Addition of ^i^*E*-VP to the chemical portfolio of oligonucleotide modifications will enable the use of stereo constraint as a way to modulate efficacy and specificity of a vast range of complex RNP structures, ranging from RISC to aptamer-protein complexes to antiviral inhibitors to CRISPR/Cas genome editing technology.

In the synthesis of ^i^*E*-VP-linked dimer phosphoramidites, nucleobase protection was essential for efficient Wittig olefination to yield dibromo-olefin derivatives (**5a**, **5b**). Using a base-labile acyl group (benzoyl, Bz) for uracil base protection (**3a**, **3b**) is likely applicable to other nucleosides, such as 4-*N*-Ac (or Bz)-cytidine, 6-*N*-Bz-adenosine, and 2-*N*-*i*Bu-guanosine, which are protected with base-labile acyl groups commonly used for phosphoramidite solid-phase oligonucleotide synthesis, thus it would not require any of nucleobase-specific additional deprotection step (38).

Substitution of P-O5′ of the phosphodiester linkage with a non-cleavable P-C (sp^2^) bond resulted in full stabilization against a P-O5′-cleaving 5′-exonuclease. Structural rigidity can both positively and negatively affect the binding affinity of enzymes to oligonucleotides and the formation of intermediate structure in enzymatic cleavage reaction. Partial stabilization against a P-O3′-cleaving 3′-exonuclease was likely due to ^i^*E*-VP-driven structural incompatibility with the enzyme active site. Likewise, the lack of resistance towards P-O3′-cleaving 5′-P-dependent 5′-exonuclease may be due to ^i^*E*-VP-induced structural fitting in the nuclease active site (50). Stability of ^i^*E*-VP backbone at the first internucleotide position in the presence of PS at 3′-downstrem of ^i^*E*-VP (**^VP^G20)** was also tested (Table 1). Rapid cleavage of **^VP^G20** was also observed, whereas **^Ctrl^G14** containing PS at first internucleotide position showed significant resistance against this nuclease (Supplementary Figure S9). Thus, iE-VP can be used to stabilize the 5′ and 3′ end of oligonucleotides, except for the first internucleotide position of 5′-P-bearing siRNA guide strands. Currently, PS is the dominant modification used in the clinic for terminal oligonucleotide protection (5–11). Current clinically-advanced lead oligonucleotides use partially-modified PS bonds to avoid toxicity (56). In these compounds, however, decreasing PS content and increasing PO content reduces metabolic stability and negatively affects duration of effect and efficacy. Combining ^i^*E*-VP with PS might generate compounds with lower toxicity while maintaining stability and potency. As for any new modification, the impact of ^i^*E*-VP incorporation on oligonucleotide toxicity must be systematically evaluated *in vivo*. Enzyme specific impact of ^i^*E*-VP was also observed with reverse transcriptases. These results imply that ^i^*E*-VP is useful biology tool to modulate interaction with various enzymes.

Backbone variants that change RNA duplex shape or charge recognition cannot be applied to siRNAs because those modifications disrupt siRNA-Ago2 interactions (57). This trend is especially true for the siRNA guide strand, which is selectively loaded into RISC and is much more sensitive to modifications than the passenger strand (1,49). Therefore, we evaluated the impact of incorporating ^i^*E*-VP into the guide strand specifically. Published crystal structures of Ago2 in complex with a fully modified guide strand (similar modification pattern used in this study) with or without cleaved substrate show that backbone torsion angles (P-O5′-C5′-C4′) vary from 140° to 177° in the majority of inter-nucleotide backbones in positions 1–14 (58,62,63). This range suggests that the incorporation of ^i^*E*-VP, expected to restrict the torsion angle to 180°, should not cause major disturbances in structure at most positions. Experimentally, this assumption was not the case. ^i^*E*-VP was poorly tolerated at many internal regions, except for positions between 5–7 and 10–12. There was no correlation between tolerance of ^i^*E*-VP, RISC function, and backbone torsion angles from currently-available crystal structures, suggesting that the observed negative effects are related to structural interference during dynamic changes in RISC formation, or in the target-induced fitting required for mRNA cleavage. This finding is consistent with the current understanding that RISC undergoes a significant conformational change to catalyse mRNA cleavage (59).

The tolerance of stereo-angle fixation at the end of the seed region (positions between 5–7) and near a mRNA cleaving site (positions 10–11) suggests that these regions do not undergo a significant conformational change or are weakly/flexibly recognized by Ago2. Indeed, tolerance at positions 5–7 is consistent with structural flexibilities previously observed in RISC crystal structure (62), and in single molecule kinetics analysis of RISC (61). In both analyses, chemical modification and mismatches in this region had minimal impact on siRNA potency and binding affinity/kinetics of the seed with target mRNA (60,61). Definitive conclusions regarding tolerance of ^i^*E-*VP in the cleavage site are still up for debate, but our findings are consistent with the current understanding that backbone positions 10 and 11 form a kinked structure that can accommodate dynamic conformational changes during target mRNA binding (61). This implies that molecular positioning of the backbone in this region is relatively unrestricted and structurally-constrained ^i^*E*-VP was tolerated in this region.

Some enhancement in activity was observed with ^i^*E*-VP incorporation at the guide strand termini (positions 1 and 2). According to crystal structures, the defined torsion angle of the phosphodiester backbone in this region is 158^o^ (58). Thus, the ^i^*E*-VP-induced shift to 180° should require some conformational adaptation. It is possible that structural fixation of the relatively flexible duplex terminus may support stable formation of RISC or RISC-substrate complex; and thus, enable sustained RISC function. This conclusion is supported by previous reports showing that incorporation of glycol nucleic acid (GNA) or unlocked-nucleic-acid (UNA) – nucleoside derivatives that induce structural flexibility – in positions 1 and/or 2 significantly decreases RISC activity (64,65). We also observed enhanced RISC activity with ^i^*E*-VP incorporation at terminal position 19–20 (PAZ binding region). Analysis of the PAZ domain in RISC crystal structure indicates a reasonable fit of ^i^*E*-VP in this region (66). Fixation of an optimal fit in PAZ may slightly enhance RISC activity (IC_50_ for **^VP^D19** and **^Ctrl^D13**: 45.5 and 82.8 nM, respectively, Supplementary Table S7). Collectively, our findings from experiments incorporating position-specific ^i^*E*-VP into the siRNA guide strand not only inform strategies for using ^i^*E*-VP to manipulate RISC efficacy, but also provide structural insight into our current understanding of RISC dynamics.

In this study, we also demonstrate an ^i^*E*-VP-driven enhancement of siRNA sensitivity towards a G to A SNP. This finding has important implications for mismatch-based discrimination strategies to achieve allele-specific silencing in disease treatment (53). While some siRNAs and ASOs have been developed to exhibit mismatch sensitivity, the level a single mismatch discrimination is usually in a range of an order of magnitude of difference in IC_50_ (44,58,59), and is not sufficient to translate into robust gene discrimination *in vivo*. Extensive chemical engineering has been employed to continue enhancing ASO mismatch-based recognition (67,68). For siRNAs, incorporation of a GNA modification at position 7 in the seed has been explored to limit potential microRNA-like target recognition (which does not require perfect complementarity) by siRNAs *in vivo* and enhance their overall specificity (69). In our study, stereo-constraining the backbone next to the primary SNP significantly enhanced siRNA ability to discriminate between mRNA alleles based on a single mismatch. This discrimination enabled productive silencing of target *Mut-HTT*, without any silencing of the *wild type*-*HTT* allele, generating a compound with complete selectivity *in vitro* based on a single mismatch.

The observed enhanced mismatch sensitivity is likely driven by the structural restriction of the functional accommodation. Normally, the structure of the RISC-substrate complex can accommodate mismatches. This accommodation requires an induced fit at the SNP-proximal backbones, which is fully blocked by the introduction of structurally-constrained ^i^*E*-VP. Future studies should confirm that similar enhancement can be observed with mismatches of different nucleotide composition.

Enhancement in SNP-based discrimination essential for translation of the SNP-selective compounds towards clinical evaluation, specifically for indications where preservation of wt allele expression is essential. Oligonucleotides do not distribute uniformly in many tissues, specifically CNS, resulting in highly significant differences in degree of accumulation across brain regions (47,56). This bias complicates ability to achieve allele specific silencing in tissue with highly different accumulation levels. Here we further demonstrate, that introduction of ^i^*E*-VP next to the mismatch allows significant enhancement of selectivity at conditions where delivery level are saturated. We intentionally treated animals with high dose, 10 mg/kg, which results in saturating level of huntingtin targeting compounds accumulation in liver. While the control compounds, containing two mismatches shows reasonable discrimination in the brain, it silencing both mutant and wild type variant in liver *in vivo*. Incorporation of the ^i^*E*-VP significantly reduced the level of wt HTT protein silencing without compromising efficacy toward mutant protein. Enhancing siRNA selectivity and abolishing single mismatch silencing by ^i^*E*-VP modification could pave the path towards developing fully allele-specific siRNAs for devastating CNS disorders, including HD.

In summary, we describe a modified high-yielding procedure enabling incorporation of ^i^*E*-VP backbone modifications into 2′-OMe and 2′-F modified oligonucleotides. The modified synthetic protocol for ^i^*E*-VP includes nucleobase protection with base labile benzoyl group, which was essential for successful adaptation of this chemistry for 2′-OMe and F modified nucleosides. Using our newly-synthesized ^i^*E*-VP-modified oligonucleotides, we determined that stereo constraint of the oligonucleotide backbone can inhibit or enhance protein recognition, depending on the enzyme in question and the position of the ^i^*E*-VP within the oligonucleotide. ^i^*E*-VP provided significant 3′ terminal stabilization. Our systematic evaluation of ^i^*E*-VP incorporation on *in vitro* siRNA efficacy demonstrates that this modification enhances activity when incorporated at termini and is tolerated in certain internal positions of the guide strand. The ability to internally modify siRNA with ^i^*E*-VP will expand the tool box of backbone modifications available for modulating clinical siRNA properties. Incorporation of structurally-constraining ^i^*E*-VP next to a SNP-mismatch base pair located in the seed region results in significant enhancement of SNP-mismatch discrimination. This novel strategy could pave the way towards the development of highly-discriminatory allele-specific siRNAs, which could potentially treat neurodegenerative disorders in the future. While ^i^*E*-VP fixes the torsion angle at 180^o^, it has minimal impact on backbone size, charge, and geometry, making it a useful modification available for, not just siRNAs, but also other oligonucleotides that require extensive protein interactions, such as RNase-H dependent ASOs and CRISPR-RNAs.

## DATA AVAILABILITY

The data that support this study are available from the corresponding authors upon reasonable request.

## Supplementary Material

gkab1126_Supplemental_File

## Notes

Present address: Anastasia Khvorova, RNA Therapeutics Institute, University of Massachusetts Medical School, Worcester, MA 01605, USA.
