# Supplementary material for: Structurally constrained phosphonate internucleotide linkage impacts oligonucleotide-enzyme interaction, and modulates siRNA activity and allele specificity
Source: Nucleic Acids Res. 2021 Nov 25;49(21):12069–88. doi: 10.1093/nar/gkab1126 (PMC8643693; doi:10.1093/nar/gkab1126)

Supporting Information

Structurally Constrained Phosphonate Internucleotide Linkage Impacts Oligonucleotide-Enzyme Interaction, and Modulates siRNA Activity and Allele Specificity

Ken Yamada[1], Samuel Hildebrand[1], Sarah M. Davis[1], Rachael Miller[1, 3], Faith Conroy[1, 3], Ellen Sapp[4], Jillian Caiazzi[1], Julia F. Alterman[1], Loic Roux[1], Dimas Echeverria[1], Matthew R. Hassler[1], Edith L. Pfister[3], Marian DiFiglia[4], Neil Aronin[1, 3], Anastasia Khvorova[1,2]*

[1]RNA Therapeutics Institute, University of Massachusetts Medical School, 368 Plantation Street, Worcester, Massachusetts 01605, United States; [2]Program in Molecular Medicine, University of Massachusetts Medical School; [3]Department of Medicine, University of Massachusetts Medical School; [4] Department of Neurology, Harvard Medical School and MassGeneral Institute for Neurodegenerative Disease, Charlestown, Massachusetts, United States.

*Corresponding author: Anastasia.Khvorova@umassmed.edu and Ken.Yamada@umassmed.edu

Contents

General remarks ---------- S3
Scheme S1 ---------- S4
Synthesis of compound **S2a** ---------- S4
Synthesis of compound **S1b** ---------- S4
Synthesis of compound **S2b** ---------- S5
Synthesis of compound **S1c** and **S2c** ---------- S6
Table S1 ---------- S7
Table S2 ---------- S8
Table S3 ---------- S9
Table S4, S5, and S6 ---------- S10
Table S7 and **Table S8** ---------- S11
Figure S1 and **Figure S2** ---------- S12
Figure S3 and **Figure S4** ---------- S13
Figure S5 and **Figure S6** ---------- S14
Figure S7 and **Figure S8** ---------- S15
Figure S9 ---------- S16
Figure S10 ---------- S17
^{1}H-NMR and ^{13}C-NMR of **2a** ---------- S18
^{19}F-NMR of **2a** and ^{1}H-NMR of **3a** ---------- S19
^{13}C-NMR and ^{19}F-NMR of **3a** ---------- S20

^{1}H-NMR and ^{13}C-NMR of **5a**--- S21

^{19}F-NMR of **5a** and ^{1}H-NMR of **6a-E**-- S22

^{13}C-NMR and ^{19}F-NMR of **6a-E**-- S23

^{1}H-NMR and ^{13}C-NMR of **6a-Z**--- S24

^{19}F-NMR of **6a-Z** and ^{1}H-NMR of **7a** (least polar isomer) -- S25

^{13}C-NMR and ^{19}F-NMR of **7a** (least polar isomer) -- S26

^{31}P-NMR of **7a** (least polar isomer) and ^{1}H-NMR of **7a** (most polar isomer) -- S27

^{13}C-NMR and ^{19}F-NMR of **7a** (most polar isomer) --- S28

^{31}P-NMR of **7a** (most polar isomer) and ^{1}H-NMR of **8a** (least polar isomer) --- S29

^{13}C-NMR and ^{19}F-NMR of **8a** (least polar isomer) -- S30

^{31}P-NMR of **8a** (least polar isomer) and ^{1}H-NMR of **8a** (most polar isomer) -- S31

^{13}C-NMR and ^{19}F-NMR of **8a** (most polar isomer) --- S32

^{31}P-NMR of **8a** (most polar isomer) and **9a** --- S33

^{1}H-NMR and ^{13}C-NMR of **3b**--- S34

^{1}H-NMR and ^{13}C-NMR of **5b**--- S35

^{1}H-NMR and ^{13}C-NMR of **6b-E** -- S36

^{1}H-NMR and ^{13}C-NMR of **6b-Z** -- S37

^{1}H-NMR and ^{13}C-NMR of **7b** (least polar isomer) --- S38

^{31}P-NMR of **7b** (least polar isomer) and ^{1}H-NMR of **7b** (most polar isomer) -- S39

^{13}C-NMR and ^{31}P-NMR of **7b** (most polar isomer) --- S40

^{1}H-NMR and ^{13}C-NMR of **8b** (least polar isomer) --- S41

^{19}F-NMR and ^{31}P-NMR of **8b** (least polar isomer) -- S42

^{1}H-NMR and ^{13}C-NMR of **8b** (most polar isomer) -- S43

^{19}F-NMR and ^{31}P-NMR of **8b** (most polar isomer) --- S44

^{31}P-NMR of **9b** and ^{1}H-NMR of **7c** (least polar isomer)--- S45

^{13}C-NMR and ^{19}F-NMR of **7c** (least polar isomer) -- S46

^{31}P-NMR of **7c** (least polar isomer) and ^{1}H-NMR of **7c** (most polar isomer)--- S47

^{13}C-NMR and ^{19}F-NMR of **7c** (most polar isomer) --- S48

^{31}P-NMR of **7c** (most polar isomer) and ^{1}H-NMR of **8c** (least polar isomer) --- S49

^{13}C-NMR and ^{19}F-NMR of **8c** (least polar isomer) -- S50

^{31}P-NMR of **8c** (least polar isomer) and ^{1}H-NMR of **8c** (most polar isomer) -- S51

^{13}C-NMR and ^{19}F-NMR of **8c** (most polar isomer) --- S52

^{31}P-NMR of **8c** (most polar isomer) and **9c** --- S53

^{1}H-NMR and ^{13}C-NMR of **S2a** --- S54

^{31}P-NMR of **S2a** and ^{1}H-NMR of **S1b** -- S55

^{13}C-NMR and ^{19}F-NMR of **S1b** -- S56

^{31}P-NMR of **S1b** and ^{1}H-NMR of **S2b** -- S57
^{13}C-NMR and ^{19}F-NMR of **S2b** --- S58
^{31}P-NMR of **S2b** ^{1}H-NMR of **S1c** -- S59
^{13}C-NMR and ^{31}P-NMR of **S1c** -- S60
^{1}H-NMR and ^{13}C-NMR of **S2c** -- S61
^{31}P-NMR of **S2c** --- S62

■ General remarks

The NMR spectra were recorded using Brucker 500 MHz spectrometer. ^{1}H, ^{13}C, ^{19}Fand ^{31}P NMR spectra were recorded at 500 MHz (^{1}H NMR, 500 MHz; ^{13}C-NMR, 125 MHz; ^{31}P-NMR, 202 MHz). The chemical shifts were measured from tetramethylsilane (0 ppm), $CDCl_3$ (7.26 ppm), DMSO-d_6 (2.49 ppm) and CD_3CN-d_3 (1.93 ppm) for ^{1}H-NMR spectra, $CDCl_3$ (77.0 ppm), DMSO-d_6 (39.7 ppm) and CD_3CN-d_3 (1.30 ppm) for ^{13}C-NMR spectra, and 85% H_3PO_4 for ^{31}P-NMR spectra as external standards. High-resolution electrospray ionization mass spectrometry (HR-ESI-MS) analysis for the monomers and dimer nucleos(t)ides were performed on a Thermo Scientific Orbitrap Velos Pro mass spectrometer in the positive ion mode. The mass analysis of oligonucleotides was conducted by LC-MS on an Agilent 6530 accurate-mass Q-TOF LC/MS (Agilent technologies, Santa Clara, CA). Thin-layer chromatography (TLC) analysis was conducted using silica gel-coated aluminium-backed TLC plates (0.20 mm thickness) containing F-254 UV indicator (Silicycle Inc., Canada). Column chromatography was performed with silica column (Flash Column Silica-CS-Agela; 12-330 g; 40-60 µm), using the CombiFlash Rf200 (Teledyne Isco, Inc.) Companion Chromatograph. The synthesis of modified oligonucleotides was performed using MerMaid-12 DNA/RNA synthesizer (Bio automation, USA). Analytical anion-exchange HPLC and reverse phase HPLC were performed on Agilent 1260 Infinity Analytical SFC System combined with an Agilent 1100 series quaternary pump with a degasser. Purified oligonucleotides were desalted by Sephadex G-25 (GE Healthcare).

Scheme S1. Synthesis of *H*-phosphonate methyl esters (**S2a**, **S2b**, and **S2c**)[a]

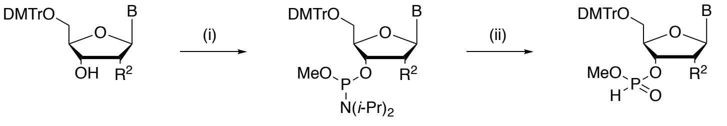

S1a (R^2 = OMe, B = Ura)
S1b (R^2 = F, B = Ura)
S1c (R^2 = OMe, B = N-*i*bu-Gua)

S2a (R^2 = OMe, B = Ura)
S2b (R^2 = F, B = Ura)
S2c (R^2 = OMe, B = N-*i*bu-Gua)

[a]Reagents and conditions: (i) *N,N*-diisopropylmethylphosphonamidic chloride, *N,N*-diisopropylethylamine/CH_2Cl_2, 0°C, 1 h, 76% (**S1a**), 74% (**S1b**), 59% (**S1c**); (ii) 1*H*-tetrazole/CH_3CN, rt, 30 min, then H_2O, rt, 10 min, quant (**S2a**), 91% (**S2b**), 97% (**S2c**).

*5′-O-DMTr-2′-O-methyl-uridine-3′-H-phosphonate methyl ester (**S2a**).* 5′-*O*-DMTr-2′-*O*-methyl-uridine (2.0 g, 3.57 mmol) was rendered anhydrous by repeated co-evaporation with anhydrous CH_3CN and then dissolved into anhydrous CH_2Cl_2 (35 mL). To this solution *N,N*-diisopropylethylamine (1.99 mL, 11.4 mmol) and *N,N*-diisopropylmethylphosphonamidic chloride (1.19 mL, 5.35 mmol) were added at 0 °C. After stirring for 1 h at 0 °C, the reaction mixture was diluted with excess CH_2Cl_2. The organic layer was repeatedly washed with sat. $NaHCO_3$ aq., dried over $MgSO_4$, filtered, and evaporated. The obtained crude material was purified by silica gel column chromatography (hexane/ethyl acetate, 80:20 to 40:60, containing 1% TEA). Obtained compound S2a with a slight amount of reagent residues was dissolved in Et_2O, repeatedly washed by sat. $NaHCO_3$ aq., and then washed by brine. Organic layer was dried over $MgSO_4$, filtered, and evaporated, yielding compound 9c as a white foam (1.95 g, 76%). To this compound S2a (1.95 g, 2.70 mmol) was added 0.45 M 1*H*-tetrazole/CH_3CN solution. After stirring for 30 min at rt., water (1.8 mL) was added and further stirred for 10 min at rt. The solution was diluted with ethyl acetate and washed repeatedly by brine 6 times. The organic layer was dried over $MgSO_4$, filtered, and evaporated, yielding sufficiently pure compound S2a as a white foam (1.71 g, quant); ^{1}H NMR (500 MHz, $CDCl_3$) δ 9.18 (br-s, 0.5H), 9.16 (br-s, 0.5 H), 7.88 (d, 0.5H, J = 8.0 Hz), 7.81 (d, 0.5H, J = 8.1 Hz), 7.37-7.24 (m, 9H), 6.99 (d, 0.5H, J_{PH} = 731.9 Hz), 6.86 (d, 4H, J = 8.3 Hz), 6.80 (d, 0.5H, J_{PH} = 718.4 Hz), 6.09 (d, 0.5H, J = 4.2 Hz), 6.04 (d, 0.5H, J = 3.3 Hz), 5.32 (dd, 0.5H, J = 8.1, 1.9 Hz), 5.27 (dd, 0.5H, J = 9.2, 2.0 Hz), 5.18-5.14 (m, 0.5H), 5.09-5.05 (m, 0.5H), 4.32-4.28 (m, 1H), 4.14 (dd, 0.5H, J = 4.6, 4.6 Hz), 4.05 (dd, 0.5H, J = 4.7, 3.5 Hz), 3.80 (d, 1.5H, J_{PH} = 12.2 Hz), 3.80 (s, 6H), 3.70 (d, 1.5H, J_{PH} = 12.2 Hz), 3.65-3.58 (m, 4H), 3.50-3.45 (m, 1H); ^{13}C NMR (125 MHz, $CDCl_3$) δ 163.0, 162.9, 158.84, 158.80, 158.78, 150.22, 150.16, 143.91, 143.86, 139.6, 134.8, 134.7, 134.6, 130.2, 130.13, 130.11, 128.2, 128.13, 128.06, 127.4, 127.3, 113.3, 102.6, 102.5, 87.5, 87.4, 86.9, 86.5, 82.8, 82.7, 82.02, 81.95, 81.53, 81.47, 73.7, 73.6, 72.1, 72.0, 61.5, 61.0, 59.0, 58.7, 55.3, 53.4, 52.1 (d, J_{CP} = 5.5 Hz), 51.6 (d, J_{CP} = 6.3 Hz); ^{31}P NMR (202 MHz, $CDCl_3$) δ 8.77, 7.93; HRMS (ESI) calcd. for $C_{32}H_{35}N_2NaO_{10}P^+$ [M + H]$^+$ *m/z* 661.1922, found *m/z* 661.1927.

*5′-O-DMTr-2′-deoxy-2′-fluoro-uridine, 3′-[methyl-N,N-bis(1-methylethyl)- phosphoramidite] (**S1b**).* 5′-*O*-DMTr-2′-deoxy-2′-fluoro-uridine (0.5 g, 0.91 mmol) was rendered anhydrous by repeated co-evaporation with anhydrous CH_3CN and then dissolved into anhydrous CH_2Cl_2 (5.0 mL). To this solution *N,N*-diisopropylethylamine (0.48 mL, 2.73 mmol) and *N,N*-diisopropylmethylphosphonamidic chloride (0.2 mL, 1.09 mmol) were added at 0 °C. After stirring for 1 h at rt, the reaction

mixture was diluted with excess CH_2Cl_2. The organic layer was repeatedly washed with sat. $NaHCO_3$ aq., dried over $MgSO_4$, filtered, and evaporated. The obtained crude material was purified by silica gel column chromatography (hexane/ethyl acetate, 80:20 to 40:60, containing 1% TEA). Obtained compound **S1b** with a slight amount of reagent residues was dissolved in Et_2O, repeatedly washed by sat. $NaHCO_3$ aq., and then washed by brine. Organic layer was dried over $MgSO_4$, filtered, and evaporated, yielding compound **S1b** as a white foam (0.48 g, 74%); ^{1}H NMR (500 MHz, CD_3CN) δ 9.13 (br-s, 1H), 7.77 (d, 0.5H, J = 8.1 Hz), 7.71 (d, 0.5H, J = 8.3 Hz), 7.47-7.35 (m, 2H), 7.35-7.23 (m, 7H), 6.88-6.85 (m, 4H), 5.90 (d, 0.5H, J_{HF} = 18.6 Hz), 5.89 (d, 0.5H, J_{HF} = 18.3 Hz), 5.27 (d, 0.5H, J = 8.2 Hz), 5.23 (d, 0.5H, J = 8.2 Hz), 5.11 (dd, 0.5H, J_{HF} = 52.8 Hz, J_{HH} = 4.4 Hz), 5.08 (dd, 0.5H, J_{HF} = 47.9 Hz, J_{HH}= 4.4 Hz), 4.71-4.63 (m, 0.5H), 4.56-4.47 (m, 0.5H), 4.15-4.13 (m, 1H), 3.77 (s, 3H), 3.76 (s, 3H), 3.62-3.48 (m, 3H), 3.42-3.36 (m, 2.5H), 3.25 (d, 1.5H, J_{PH} = 13.1 Hz), 1.17-1.13 (m, 9H), 1.03-1.01 (m, 3H); ^{13}C NMR (125 MHz, CD_3CN) δ 164.02, 164.00, 159.88, 159.86, 151.2, 145.9, 141.8, 141.6, 136.58, 136.55, 136.4, 131.29, 131.26, 131.24, 131.22, 129.2, 129.1, 128.99, 128.1, 114.20, 114.17, 102.7, 102.6, 94.3 (d, J_{CF} = 188.0 Hz), 93.8 (d, J_{CF} = 190.8 Hz), 90.5 (d, J_{CF} = 36.3 Hz), 90.2 (d, J_{CF} = 35.4 Hz), 87.61, 87.60, 82.3 (d, J_{CF} = 3.7 Hz), 82.2 (d, J_{CF} = 6.3 Hz), 70.7 (dd, J = 15.5, 15.5 Hz), 70.1 (dd, J = 16.3, 16.3 Hz), 62.5, 61.8, 56.01, 55.99, 51.3 (d, J_{CP} = 17.2 Hz), 51.0 (d, J_{CP} = 17.3 Hz), 44.02, 44.00, 43.92, 43.90, 25.2, 25.11, 25.08, 25.06, 25.03, 24.96, 24.90; ^{19}F NMR (470 MHz, CD_3CN) δ -199.36, -199.38, -199.40, -199.42, -199.44, -199.47, -199.49, -199.51, -199.53, -199.56, -199.57, -199.97, -199.90, -200.01, -200.02, -200.03, -200.06, -200.07, -200.08, -200.10, -200.12, -200.14, -200.15, -200.17, -200.19; ^{31}P NMR (202 MHz, CD_3CN) δ 151.00 (d, J_{PF} = 9.9 Hz), 150.99 (d, J_{PF} = 10.0 Hz); HRMS (ESI) calcd. for $C_{37}H_{46}FN_3O_8P^+$ $[M + H]^+$ *m/z* 710.3001, found *m/z* 710.2999.

5'-O-DMTr-2'-deoxy-2'-fluoro-uridine-3'-H-phosphonate methyl ester ***(S2b)***

Anhydrous **S1b** (2.85 g, 4.02 mmol) was dissolved in 0.45 M 1*H*-tetrazole/CH_3CN solution (18.0mL, 8.04 mmol) and stirred for 30 min at rt. To this solution, H_2O (2.4 mL) was added and stirred for 30 min at rt. After diluting with ethyl acetate, the organic solution was washed with brine six times, dried over $MgSO_4$, filtered and then evaporated. Obtained compound **S2b** was sufficiently pure and obtained as a white solid (2.29 g, 91%); ^{1}H NMR (500 MHz, $CDCl_3$) δ 9.06 (br-s, 1H), 7.82 (d, 0.5H, J = 8.2 Hz), 7.77 (d, 0.5H, J = 8.1 Hz), 7.38-7.24 (m, 9H), 6.93 (dd, 0.5H, J_{PH} = 725.8 Hz, J_{HF} = 1.8 Hz), 6.86-6.84 (m, 4H), 6.78 (d, 0.5H, J_{PH} = 723.5 Hz), 6.15 (dd, 0.5H, J_{HF} = 15.7 Hz, J_{HH} = 2.8 Hz), 6.11 (dd, 0.5H, J_{HF} = 16.2 Hz, J_{HH} = 2.0 Hz), 5.37 (dd, 0.5H, J = 8.1, 2.0 Hz), 5.33-5.12 (m, 3H), 4.33-4.30 (m, 1H), 3.81-3.77 (m, 8.5H), 3.70-3.65 (m, 2.5H), 3.50 (dd, 0.5H, J = 11.3, 2.4 Hz), 3.46 (dd, 0.5H, J = 11.4, 2.4 H); ^{13}C NMR (125 MHz, $CDCl_3$) δ 162.8, 162.7, 158.88, 158.86, 158.84, 158.83, 150.0, 149.9, 143.9, 143.8, 139.63, 139.60, 134.81, 134.80, 134.75, 134.65, 130.23, 130.20, 130.18, 130.13, 128.2, 128.1, 128.0, 127.4, 127.3, 113.4, 113.3, 102.91, 102.85, 92.7, 92.3, 91.2, 90.8, 878.0, 87.7, 87.6, 87.51, 87.45, 87.28, 81.2, 81.1, 80.8, 80.7, 71.6 (d, J_{CF} = 5.5 Hz), 71.4 (d, J_{CF} = 5.5 Hz), 71.0 (d, J_{CF} = 5.5 Hz), 70.9 (d, J_{CF} = 5.4 Hz), 60.8, 60.4, 55.29, 55.25, 52.3 (d, J_{CP} = 6.3 Hz), 52.1 (d, J_{CF} = 6.4 Hz); ^{19}F NMR (470 MHz, $CDCl_3$) δ -199.6 (ddd, J = 52.0, 19.1, 19.1); ^{31}P NMR (202 MHz, $CDCl_3$) δ 8.64, 8.25; HRMS (ESI) calcd. for $C_{31}H_{32}FN_2NaO_9P^+$ $[M + Na]^+$ *m/z* 649.1722, found *m/z* 649.1720.

5′-O-DMTr-2′-O-methyl-2-N-isobutyryl-guanosine, 3′-[methyl-N,N-bis(1-methylethyl)- phosphoramidite] ***(S1c)***. 5′-*O*-DMTr-2′-*O*-methyl-2-*N*-isobutyryl-guanosine (3.35 g, 5.00 mmol) was rendered anhydrous by repeated co-evaporation with anhydrous CH_3CN and then dissolved into anhydrous CH_2Cl_2 (50 mL). To this solution *N*,*N*-diisopropylethylamine (2.61 mL, 15.0 mmol) and *N,N*-diisopropylmethylphosphonamidic chloride (1.60 mL, 8.24 mmol) were added at 0 °C. After stirring for 1 h at rt, the reaction mixture was diluted with excess CH_2Cl_2. The organic layer was repeatedly washed with sat. $NaHCO_3$ aq., dried over $MgSO_4$, filtered, and evaporated. The obtained crude material was purified by silica gel column chromatography (CH_2Cl_2/acetone, 100:0 to 70:30, containing 1% TEA). Obtained compound **S1c** with a slight amount of reagent residues was dissolved in Et_2O, repeatedly washed by sat. $NaHCO_3$ aq., and then washed by brine. Organic layer was dried over $MgSO_4$, filtered, and evaporated, yielding compound **S1c** as a white foam (2.45 g, 59%); ^{1}H NMR (500 MHz, $CDCl_3$) δ 11.9 (1H, br-s), 7.82-7.81 (m, 1H), 7.58-7.55 (m, 2H), 744-7.40 (m ,4H), 7.32-7.26 (m, 3H), 6.83-6.78 (m, 4H), 5.91-5.83 (m, 1H), 4.79-4.72 (m, 1H), 4.64-4.58 (m, 1H), 4.32-4.26 (m, 1H), 3.77 (s, 3H), 3.76 (s, 3H), 3.60-3.50 (m, 3H), 3.46-3.42 (m, 5H), 3.18 (d, 1H, J = 13.5 Hz), 3.13-3.09 (m, 1H), 1.65-1.58 (m, 1H), 1.32-1.14 (m, 10H), 0.98 (d, 3H, J = 6.8 Hz), 0.91-0.88 (m, 3H), 0.72-0.70 (m, 3H); ^{13}C NMR (125 MHz, $CDCl_3$) δ 178.3, 158.78, 158.76, 158.74, 155.6, 148.23, 148.20, 147.1, 145.1, 144.9, 138.9, 136.2, 136.0, 135.8, 135.7, 130.0, 128.12, 128.08, 128.07, 128.04, 127.2, 127.1, 122.55, 122.53, 113.29, 113.26, 86.7, 86.6, 86.4, 86.2, 84.8, 84.25, 84.22, 81.76, 81.74, 81.21, 81.16, 70.6, 70.5, 70.1, 70.0, 63.4, 63.0, 58.73, 58.70, 58.26, 58.23, 55.3, 53.5, 51.1, 51.0, 50.1, 49.9, 43.14, 43.05, 42.8, 42.7, 36.1, 24.71, 24.65, 24.60, 18.6, 18.5, 18.4; ^{31}P NMR (202 MHz, $CDCl_3$) δ 151.3, 150.8. HRMS (ESI) calcd. for $C_{43}H_{56}N_6O_9P^+$ $[M + H]^+$ *m/z* 831.3841, found *m/z* 831.3841.

5′-O-DMTr-2′-O-methyl-2-N-isobutyryl-uridine-3′-H-phosphonate methyl ester (S2c). Anhydrous **S1c** (2.45 g, 2.95 mmol) was dissolved in 0.45 M 1*H*-tetrazole/CH_3CN solution (13.1mL, 5.90 mmol) and stirred for 30 min at rt. To this solution, H_2O (1.78 mL) was added and stirred for 30 min at rt. After diluting with ethyl acetate, the organic solution was washed with brine six times, dried over $MgSO_4$, filtered and then evaporated. Obtained compound **S2c** was sufficiently pure and obtained as a white solid (2.13 g, 97%); ^{1}H NMR (500 MHz, $CDCl_3$) δ 11.97 (br-s, 0.5H), 11.95 (br-s, 0.5H), 9.18 (br-s, 0.5H), 8.52 (br-s, 0.5H), 7.79 (d, 1H, J = 4.7 Hz), 7.44-7.43 (m, 1H), 7.35-7.33 (m, 1H), 7.31-7.29 (m, 2H), 7.24-7.18 (m, 5H), 7.04 (d, 0.5H, J_{PH} = 729.2 Hz), 6.79-6.74 (m, 4H), 6.75 (d, 0.5H, J_{PH} = 719.2 Hz), 6.16-6.12 (m, 0.5H), 5.85-5.84 (m, 1H), 5.73-5.70 (m, 0.5H), 5.04-5.02 (m, 0.5H), 4.86-4.84 (m, 0.5H), 4.33-4.28 (m, 1H), 3.81 (d, 0.5H, J_{PH} = 12.5 Hz), 3.77-3.78 (m, 6H), 3.67 (d, 0.5H, J_{PH} = 12.0 Hz), 3.53 (s, 1.5H), 3.53 (s, 1.5H), 3.17-3.09 (m, 1H), 2.19-2.15 (m, 0.5H), 1.91-1.85 (m, 0.5H), 1.08 (d, 1.5H, J = 6.9 Hz), 1.01 (d, 1.5H, J = 6.9 Hz), 0.95 (d, 1.5H, J = 6.9 Hz), 0.84 (d, 1.5H, J = 6.9 Hz); ^{13}C NMR (125 MHz, $CDCl_3$) δ178.8, 178.6, 158.74, 158.72, 158.6, 155.5, 155.4, 147.9, 147.6, 147.5, 147.4, 144.6, 144.4, 139.1, 139.0, 135.67, 135.66, 135.31, 135.27, 130.0, 129.9, 128.0, 127.95, 127.93, 127.8, 127.1, 127.0, 122.3, 122.1, 113.2, 113.1, 86.5, 86.3, 85.9, 82.0, 81.9, 81.6, 81.3, 80.6, 80.5, 74.3, 74.2, 72.9, 72.8, 62.5, 61.1, 59.3, 58.8, 55.2, 55.23, 55.21, 53.4, 52.2 (d, J_{CP} = 5.4 Hz), 51.7 (d, J_{CP} = 5.5 Hz), 36.2, 36.1, 18.58, 18.55, 18.54, 18.51; ^{31}P NMR (202 MHz, $CDCl_3$) δ 9.09, 8.79; HRMS (ESI) calcd. for $C_{37}H_{43}N_5O_{10}P^+$ $[M + H]^+$ *m/z* 748.2742, found *m/z* 748.2748.

Table S1. Sequence of control guide strands (^{Ctrl}**G1**-**14**)

^{Ctrl}Guide strands	Sequence (5′ -> 3′)^{a}	Calcd mass [M-H]$^{-}$	Found mass	Correspond Sense strands^{b}	^{Ctrl}siRNA #
^{Ctrl}**G1**	P-U#U#AAUCUCUUUAC#U#G#A#U#A#U#A	6619.4	6619.7	P1	^{Ctrl}D1
^{Ctrl}**G2**	P-U#U#UUUAAAUCCUG#A#G#A#A#G#A#A	6744.6	6744.7	P2	^{Ctrl}D2
^{Ctrl}**G3**	P-U#C#UCUUUACUGAU#A#U#A#A#U#U#A	6619.4	6619.7	P3	^{Ctrl}D3
^{Ctrl}**G4**	P-U#A#UGUUUCACAU#A#U#U#G#U#C#A	6635.4	6635.6	P4	^{Ctrl}D4
^{Ctrl}**G5**	P-U#G#AAUGUUCACGC#A#G#U#G#G#G#C	6806.6	6806.8	P5	^{Ctrl}D5
^{Ctrl}**G6**	P-U#A#UCAGCUUUCC#A#G#G#G#U#C#G	6704.5	6704.7	P6	^{Ctrl}D6
^{Ctrl}**G7**	P-U#U#AACGUCAGUUC#A#U#A#A#A#C#C	6679.5	6679.7	P7	^{Ctrl}D7
^{Ctrl}**G8**	P-U#C#CACUAUGUUUU#C#A#C#A#U#A#U	6594.4	6594.7	P8	^{Ctrl}D8
^{Ctrl}**G9**	P-U#C#CAAAUACUGGU#U#G#U#C#G#G#U	6728.5	6728.7	P9	^{Ctrl}D9
^{Ctrl}**G10**	P-U#C#CGGUCACAACA#U#U#G#U#G#G#U	6727.5	6727.7	P10	^{Ctrl}D10
^{Ctrl}**G11**	P-U#U#UGGUAGCUGAA#A#G#U#U#C#U#U	6730.5	6730.7	P11	^{Ctrl}D11
^{Ctrl}**G12**	P-U#U#AAUCUCUUUAC#U#G#A#U#U#U#A	6596.4	6596.6	P12	^{Ctrl}D12
^{Ctrl}**G13**	P-U#U#AAUCUCUUUAC#U#G#A#U#A#U#U	6596.4	6596.6	P13	^{Ctrl}D13
^{Ctrl}**G14**	P-U#U#AAUCUCUUUAC#U#G#A#U#U#U#U	6573.3	6573.6	P14	^{Ctrl}D14

^{a} Uppercases and underlined uppercases represents 2'-OMe and 2'-F, respectively. 5'-end phosphate is represented as "5'P". Inter-nucleotide phosphorothioate is indicated by "#" symbol. ^{b}3'-end tetraethylene glycol (Teg) -linked cholesterol (Chol) conjugated 15 mer sense strands (**P1**-**14**) to prepare ^{Ctrl}siRNAs (^{Ctrl}**D1**-**14**) consists with (i) complementary sequence through 5'-end to 15th nucleotide of guide strands and (ii) with fully chemical modification pattern: 5'-N#N#NNNNNNNNNNN#N#N-Teg-Chol-3' (N and N represent 2'-OMe and 2'-F, respectively. See **Table S2** for each of sense strand sequences).

Table S2. Sequence of passenger strands (**P1**-**14**) used for in-vitro screening assay

Passenger strands	Sequence (5′ -> 3′)[a]	Calcd mass $[M-H]^-$	Found mass
**P1**	5'-C#A#GUAAAGAGAUU#A#A-TegChol	5765.3	5765.2
**P2**	5'-C#U#CAGGAUUUAAA#A#A-TegChol	5702.3	5702.1
**P3**	5'-A#U#AUCAGUAAAGA#G#A-TegChol	5765.3	5765.2
**P4**	5'-A#U#AUGUGAAAACA#U#A-TegChol	5726.3	5726.2
**P5**	5'-C#U#GCGUGAACAUU#C#A-TegChol	5670.2	5670.1
**P6**	5'-C#U#GGAAAAGCUGA#U#A-TegChol	5757.3	5757.2
**P7**	5'-A#U#GAACUGACGUU#A#A-TegChol	5718.3	5718.4
**P8**	5'-U#G#AAAACAUAGUG#G#A-TegChol	5781.3	5781.2
**P9**	5'-C#A#ACCAGUAUUUG#G#A-TegChol	5694.3	5694.1
**P10**	5'-A#A#UGUUGUGACCG#G#A-TegChol	5750.3	5750.1
**P11**	5'-C#U#UUCAGCUACCA#A#A-TegChol	5614.2	5614.1
**P12**	5'-C#A#GUAAAGAGAUU#A#A-TegChol	5765.3	5765.2
**P13**	5'-C#A#GUAAAGAGAUU#A#A-TegChol	5765.3	5765.2
**P14**	5'-C#A#GUAAAGAGAUU#A#A-TegChol	5765.3	5765.2

[a] Uppercases and underlined uppercases represents 2'-OMe and 2'-F, respectively. 5'-end phosphate is represented as “5'P”. Inter-nucleotide phosphorothioate is indicated by “#” symbol; All passenger strands has 3'-end tetraethylene glycol (Teg) -linked cholesterol (Chol) conjugate.

Table S3. Sequence of oligonucleotides (**ON1−8**, ^{Ctrl}**G14**, and ^{VP}**G20**) used for nuclease stability test

ON #	Sequence (5′ -> 3′)[a]	Calcd mass [M-H]$^{-}$	Found mass
**ON1**	P-UUAAUCUCUUUACUGAUUUU	6428.8	6428.8
**ON2**	P-U**vp**UAAUCUCUUUACUGAU**vp**UU**vp**U	6416.8	6416.8
**ON3**	UUAAUCUCUUUACUGAUUUU	6348.8	6348.8
**ON4**	U**vp**UAAUCUCUUUACUGAU**vp**UU**vp**U	6336.8	6336.8
**ON5**	P-U#C#UCUUUACUGAU#A#U#A#A#U#U#A*NNNGTCNNNTAGNNNTGGA* *ATTCTCGGGTGCCAAGGddC*	*see Table 1 and S1*	
**ON6**	P-U#C#UCU**vp**UUACUGAU#A#U#A#A#U#U#A*NNNGTCNNNTAGNNNTGGA* *ATTCTCGGGTGCCAAGGddC*		
**ON7**	P-U#U#AAUCUCUUUAC#U#G#A#U#A#U#A*NNNGTCNNNTAGNNNTGGA* *ATTCTCGGGTGCCAAGGddC*		
**ON8**	P-U#U#AAUCUCUU**vp**UAC#U#G#A#U#A#U#A*NNNGTCNNNTAGNNNTGGA* *ATTCTCGGGTGCCAAGGddC*		
^{Ctrl}**G14**	5'P-U#U#AAUCUCUUUAC#U#G#A#U#U#U#U		
^{VP}**G20**	5'P-U**vp**U#AAUCUCUUUAC#U#G#A#U**vp**U#U**vp**U		

[a] Uppercases, underlined uppercases, and italic uppercase represents 2'-OMe, 2'-F and 2’-deoxy nucleosides, respectively. ddC represents dideoxy-cytidine. Continuous *N* sequence represents randomized sequence with DNA. 5'-end phosphate is represented as “P”. Inter-nucleotide phosphorothioate and (*E*)-vinylphosphonate linkages are indicated by “#” symbol and **vp**, respectively. 5’-Adenylated 36-nt DNA adapter oligonucleotide for **ON5**-**8** were purchased from Integrated DNA Technologies, Inc. and ligated to 3’-end of ^{Ctrl}**G3**, ^{VP}**G5**, ^{Ctrl}**G1**, and ^{VP}**G10** for **ON5**, **6**, **7**, and **8**, respectively according to the method described in main manuscript.

Table S4. Sequence of ^{i}*E*-VP-modified guide strands (**^{VP}G21-23)** targeting *HTT*-SNP rs362273

^{vp}**Guide strands**	Sequence (5′ -> 3′)[a]	Calcd mass [M-H]$^{-}$	Found mass	Correspond Sense strands[b]	^{VP}siRNA#
**^{VP}G21**	5'P-U#U#CUG**vp**UAGCAUCA#G#C#U#U#C#U#C	6621.4	6621.7	**P15**	**^{VP}D21**
**^{VP}G22**	5'P-U#U#CUG**vp**UAGCAACA#G#C#U#U#C#U#C	6644.5	6644.7	**P16**	**^{VP}D22**
**^{VP}G23**	5'P-U#U#CUG**vp**UAGCACCA#G#C#U#U#C#U#C	6620.4	6620.7	**P17**	**^{VP}D23**

[a] Uppercases and underlined uppercases represents 2'-OMe and 2'-F, respectively. 5'-end phosphate is represented as "5'P". Inter-nucleotide phosphorothioate and (*E*)-vinylphosphonate linkages are indicated by "#" symbol and **vp**, respectively.

Table S5. Sequence of control guide strands (**^{Ctrl}G15-17)** targeting *HTT*-SNP rs362273 and sense strands

^{Ctrl}**Guide strands**	Sequence (5′ -> 3′)[a]	Correspond Sense strands[b]	^{Ctrl}siRNA#
**^{Ctrl}G15**	5'P-U#U#CUGUAGCAGCA#G#C#U#U#C#U#C	**P15**	**^{Ctrl}D15**
**^{Ctrl}G16**	5'P-U#U#CUGUAGCAUCA#G#C#U#U#C#U#C	**P16**	**^{Ctrl}D16**
**^{Ctrl}G17**	5'P-U#U#CUGUAGCAACA#G#C#U#U#C#U#C	**P17**	**^{Ctrl}D17**

[a] Uppercases and underlined uppercases represents 2'-OMe and 2'-F, respectively. 5'-end phosphate is represented as "5'P". Inter-nucleotide phosphorothioate is indicated by "#" symbol. [b]3'-end tetraethylene glycol (Teg)-linked cholesterol (Chol) conjugated 15 mer sense strands (**P15**-**17**) to prepare ^{Ctrl}siRNAs (**^{Ctrl}D15**-**17**, see **Table S6** for each of sense strand sequences).

Table S6. Sequence of sense strands (**P15**-**17**) for **^{vp}G21**-**23** and **^{Ctrl}G15**-**17**

**Passenger strands**	Sequence (5′ -> 3′)[a]
**P15**	G#C#UGAUGCUACAG#A#A-TegChol
**P16**	G#C#UGUUGCUACAG#A#A-TegChol
**P17**	G#C#UGGUGCUACAG#A#A-TegChol

[a] Uppercases and underlined uppercases represents 2'-OMe and 2'-F, respectively. 5'-end phosphate is represented as "5'P". Inter-nucleotide phosphorothioate is indicated by "#" symbol; All passenger strands have 3'-end tetraethylene glycol (Teg) -linked cholesterol (Chol) conjugate.

Table S7. IC_{50} values of ^{VP}siRNAs and ^{Ctrl}siRNAs

^{VP}siRNAs	IC_{50} (nM)[a]	^{VP}siRNAs	IC_{50} (nM)[a]	^{Ctrl}siRNAs	IC_{50} (nM)[a]	^{Ctrl}siRNAs	IC_{50} (nM)[a]
^{VP}D1	20.4	^{VP}D11	338.7	^{Ctrl}D1	47.1	^{Ctrl}D11	50.1
^{VP}D2	177.3	^{VP}D12	N.D.	^{Ctrl}D2	86.4	^{Ctrl}D12	33.0
^{VP}D3	N.D.	^{VP}D13	N.D.	^{Ctrl}D3	618.0	^{Ctrl}D13	82.8
^{VP}D4	342.7	^{VP}D14	954.7	^{Ctrl}D4	271.9	^{Ctrl}D14	81.5
^{VP}D5	674.4	^{VP}D15	6208	^{Ctrl}D5	2306		
^{VP}D6	251.3	^{VP}D16	101.8	^{Ctrl}D6	265.9		
^{VP}D7	N.D.	^{VP}D17	64.5	^{Ctrl}D7	246.3		
^{VP}D8	522.2	^{VP}D18	1729	^{Ctrl}D8	1307		
^{VP}D9	170.4	^{VP}D19	45.5	^{Ctrl}D9	606.8		
^{VP}D10	37.5	^{VP}D20	132.5	^{Ctrl}D10	123.3		

[a]IC_{50} values were reported as the mean values of three independent experiments.

Table S8. Sequence of oligonucleotide used for in vivo SNP-discrimination

**Strands**	Sequence (5′ -> 3′)[a]	Calcd Mass $[M-H]^-$	Found Mass	siRNA#
**^{Ctrl}G18**	5’**vp-**U#U#CUGUAGCAUCAG#C#U#U#C#U#C	6621.44	6621.75	^{Ctrl}D18
**^{VP}G24**	5’**vp-**U#U#CUG**vp**UAGCAUCAG#C#U#U#C#U#C	6616.43	6617.00	^{VP}D24
**P18**	GCUGAUGCUACAGAA-**GalNAc**	6796.20	6796.65	———

[a] Uppercases and underlined uppercases represents 2'-OMe and 2'-F, respectively. 5'-end (E)-vinylphosphonate is represented as “5'**vp**”. Inter-nucleotide phosphorothioate is indicated by “#” symbol; All passenger strands have 3'-end trivalent GalNAc conjugate.

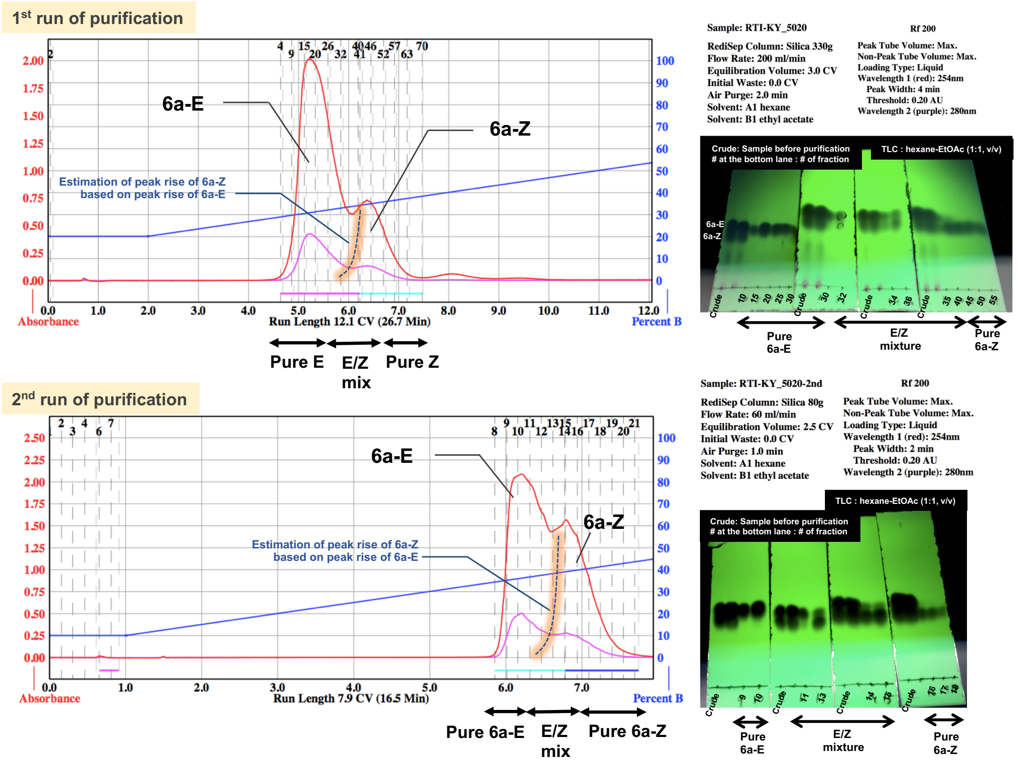

Figure S1. Combiflash chromatographic profile of silica-gel column purification of **6a-E/Z** and TLC analysis of fractions.

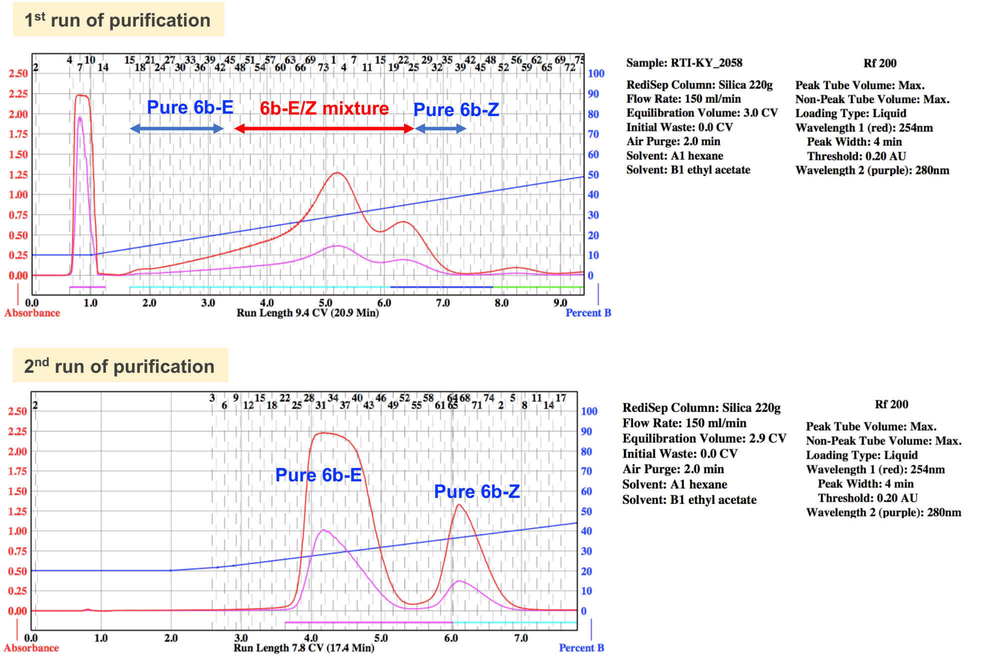

Figure S2. Combiflash chromatographic profile of silica-gel column purification of **6b-E/Z**.

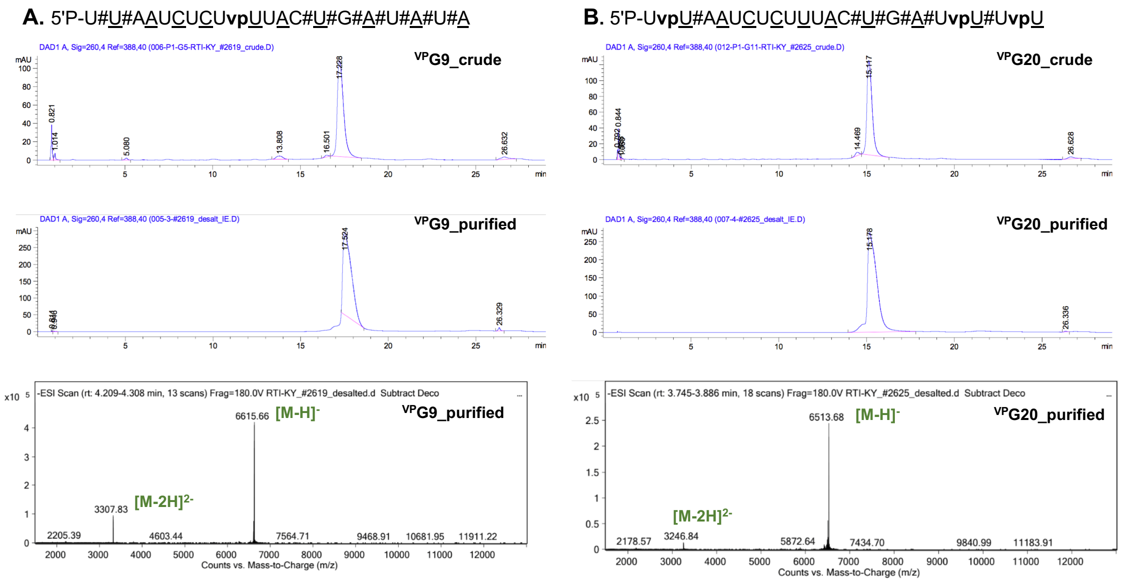

Figure S3. Representative IE-HPLC and Mass profile of oligonucleotides (A) **^{VP}G9** and (B) **^{VP}G20** obtained using phosphoramidite **9a**; IE-HPLC profile of crude sample (top), IE-HPLC profile of purified sample (middle), and Mass profile of purified sample (bottom), respectively. HPLC conditions: PL-SAX ion exchange column (Agilent) (1000 Å pore size; 8 µM particle size; 150 mm length, 4.6 mm diameter), solvent A: 10 mM NaOAc buffer (pH 5.0) containing 20% CH_3CN, B: 1M $NaClO_4$ in water containing 20% CH_3CN, B: 2–35% in 30 min.

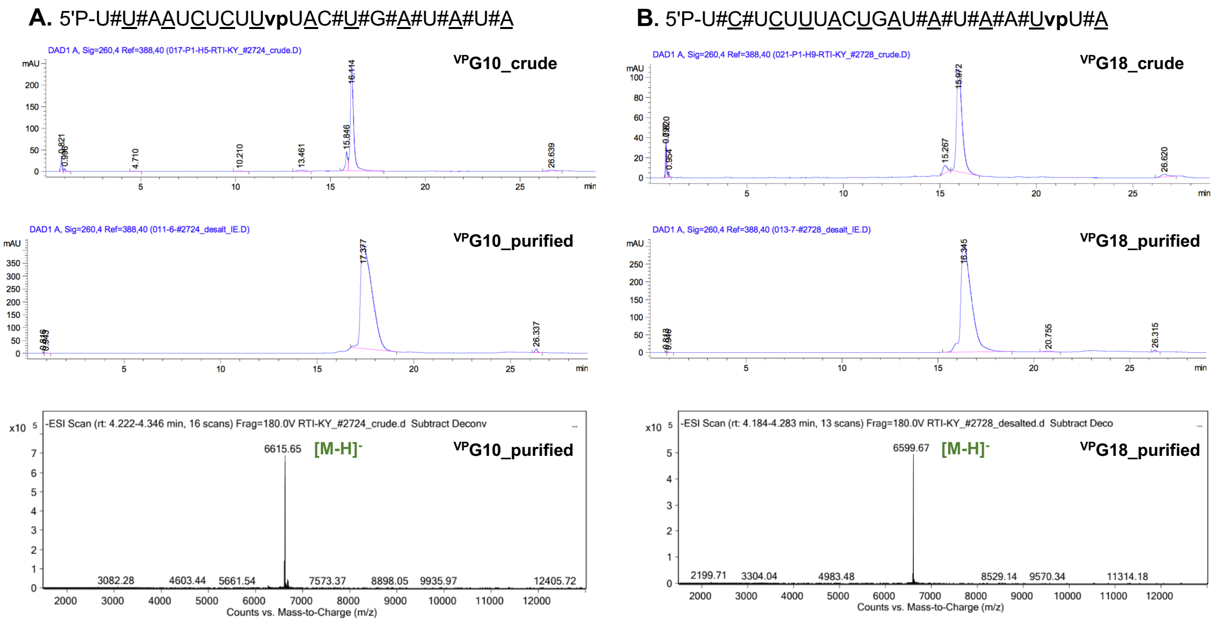

Figure S4. Representative IE-HPLC and Mass profile of oligonucleotides (A) **^{VP}G10** and (B) **^{VP}G18** obtained using phosphoramidite **9b**; IE-HPLC profile of crude sample (top), IE-HPLC profile of purified sample (middle), and Mass profile of purified sample (bottom), respectively. HPLC conditions are same as descried in Figure S1.

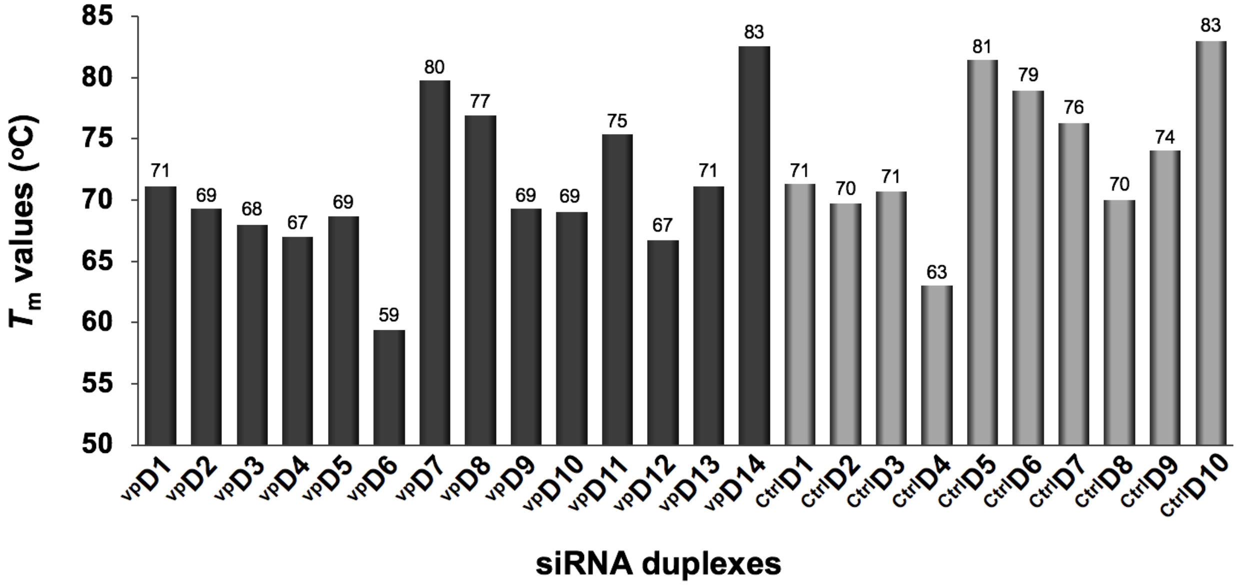

Figure S5. UV melting temperatures of siRNA duplexes (^{VP}**D1-14** and ^{Ctrl}**D1-10**). Melting temperature analysis was conducted in a buffer 10mM Sodium phosphate buffer (pH 7.2) containing 100 mM NaCl, 0.1 mM EDTA and 1 mM guide/sense strand. T_m values were average values determined in triplicate experiments.

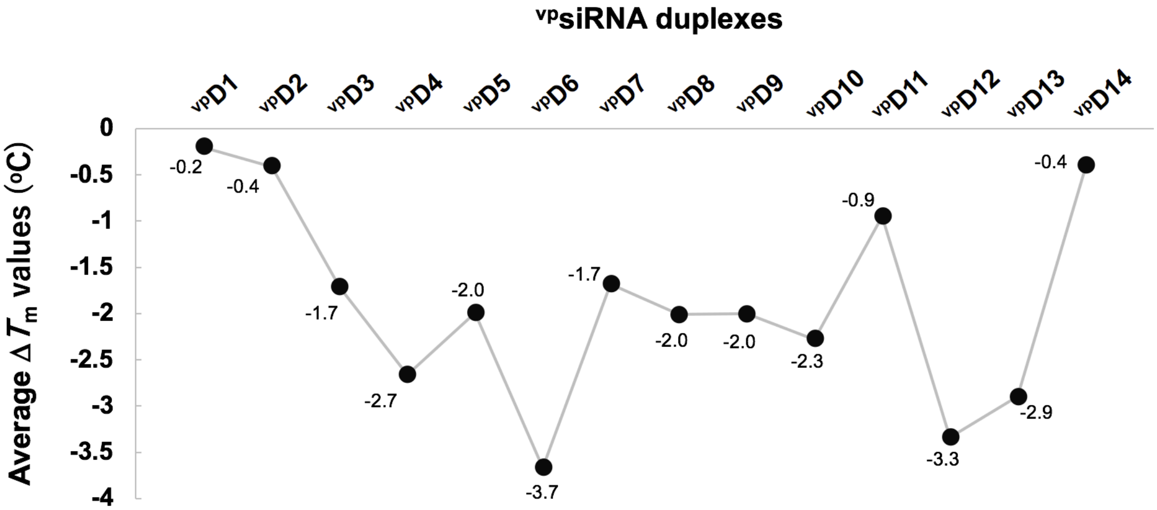

Figure S6. Difference in T_m values (ΔT_m) between ^{VP}siRNA duplexes and corresponding ^{Ctrl}siRNA duplexes. $\Delta T_m = T_m$ (VP-modified) − T_m (VP-unmodified). (see Figure S1 for average T_m values used for calculation)

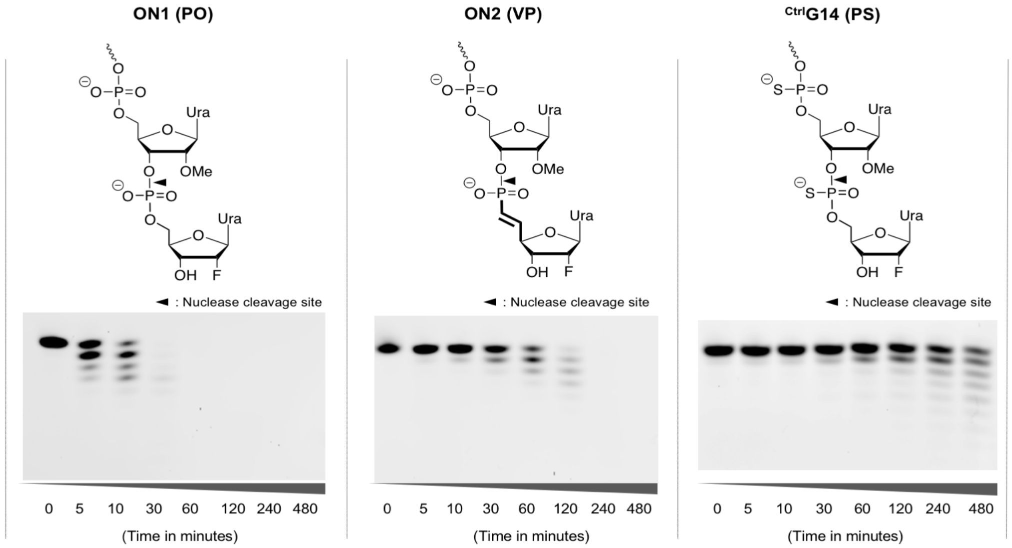

Figure S7. Denature gel of **ORN1**, **ORN2**, and **^{Ctrl}G14** after incubation with exaggerated 3′-exonuclease (SVPD). 17.5 mM each oligonucleotide was incubated in 10 mM Tris-HCl (pH 8), 10 mM $MgCl_2$ buffer containing 10mU/mL SVPD. Aliquot was taken from reaction mixture at each time point.

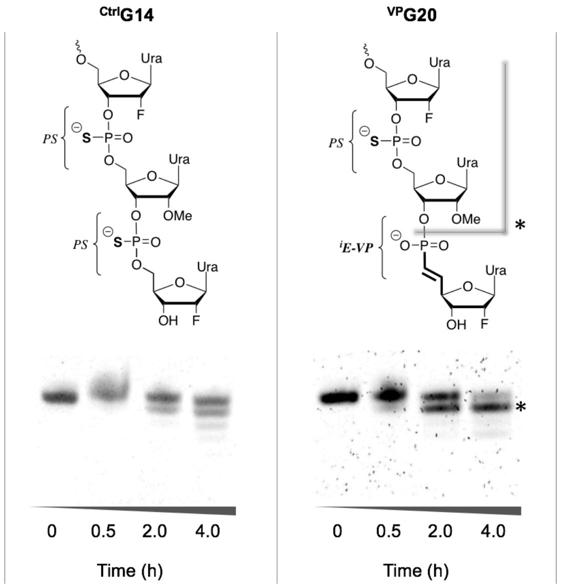

Figure S8. Denature gel of **^{Ctrl}G14** and **^{VP}G20** after incubation with exaggerated 3′-exonuclease (SVPD). 17.5 mM each oligonucleotide was incubated in 10 mM Tris-HCl (pH 8), 10 mM $MgCl_2$ buffer containing 10mU/mL SVPD. Aliquot was taken from reaction mixture at each time point. Denature gel of digestion of **^{VP}G20** (right gel) shows significant intensity of the band of ^{i}*E*-VP cleaved oligonucleotide (indicated by * on the gel) indicating that next PS linkage is a rate limiting step of digestion reaction.

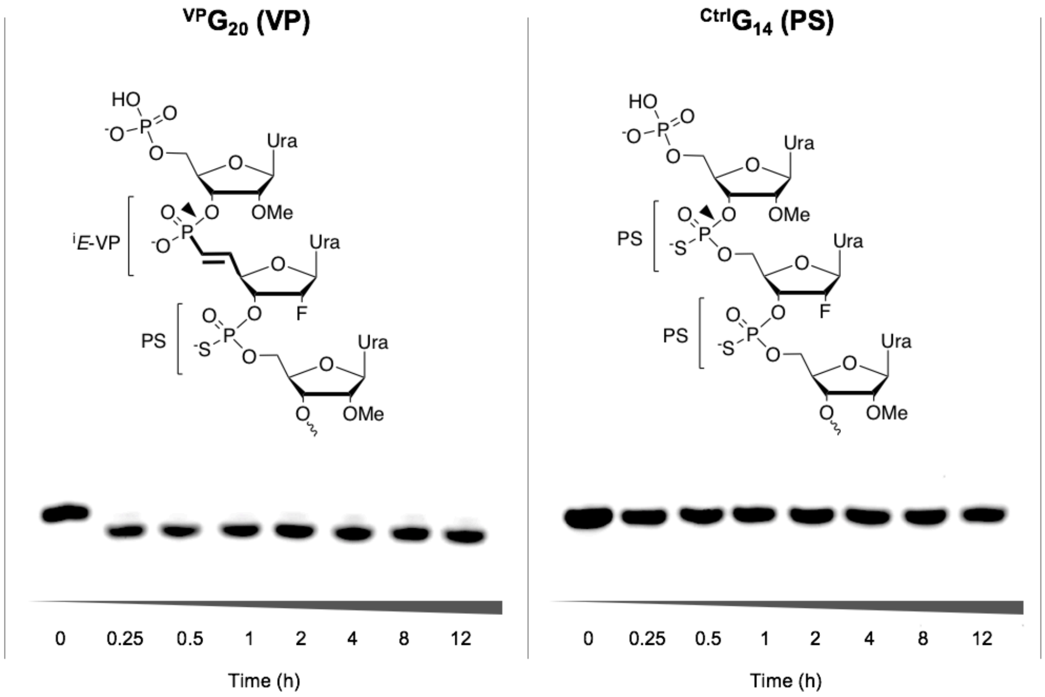

Figure S9. Denature gel of **VPG20 and CtrlG14** after incubation with 5′-P-dependent 5′-exonuclease. 5 μM oligonucleotide (100 pmol) were incubated in RNase-free water, or with Terminator™ (EpiCentre) exonuclease (50 U/mL) at 37°C in buffer A (EpiCentre, provided with Terminator™ enzyme). Aliquot was taken from reaction mixture at each time point. Denature gel of the digestion of **VPG20** (left gel) showed significant intensity of the band of -1 mer oligonucleotide indicating that nuclease cleavage stopped at second inter-nucleotide PS linkage. The gel on the right showed significant stability of PS-linkage.

A.

siRNA # and target[a]	Sequence of siRNAs and targeting region on *HTT*-gene[b]	
**^{VP}D22**	5’- P-U#U#CUG**vp**UAGCAACA#G#C#U#U#C#U#C -3'	(**^{VP}G22**)
	3’- Chol-Teg-A#A#GACAUCGUUG#UC#G -5'	(**P16**)
**^{VP}D23**	5’-P-U#U#CUG**vp**UAGCACCA#G#C#U#U#C#U#C -3’	(**^{VP}G23**)
	3’ -Chol-Teg-A#A#GACAUCGUGGU#C#G -5’	(**P17**)
target	3'- ...AGAC**A**UCGUCGUCGAAGAG… -5'	(HTT-Mut)
mm-target	3'- ...AGAC**G**UCGUCGUCGAAGAG… -5'	(HTT-WT)

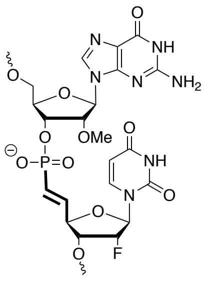

GvpU

B.

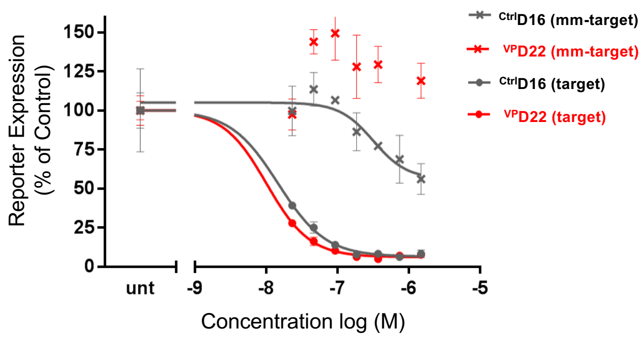

C.

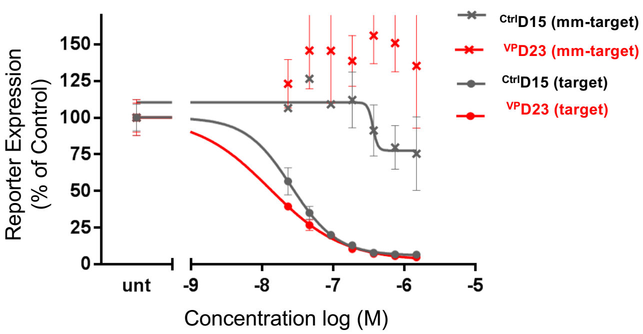

Figure S10. SNPs discrimination properties of ^{i}*E*-VP-modified siRNAs; (**A**) Sequence of siRNAs (**^{VP}D22** and **^{VP}D23**), target mRNAs with or without single mismatch (mm) base (target and mm-target, respectively); (**B**) Does response of **^{VP}D22** and **^{Ctrl}D16** in the presence of target or mm-target mRNAs; (**C**) Does response of **^{vp}D23** and **^{Ctrl}D15** in the presence of target or mm-target mRNAs.[a]**^{VP}D22** and **^{VP}D23** consists with **^{VP}G22/P16** and **^{VP}G23/P17**, respectively; [b]Uppercases and underlined uppercases represents 2'-OMe and 2'-F, respectively. 5'-end phosphate is represented as “5'-P”. Inter-nucleotide phosphorothioate and (*E*)-vinylphosphonate linkages are indicated by “#” symbol and **vp**, respectively. Passenger strands has 3'-end tetraethylene glycol (Teg)-linked cholesterol (Chol) conjugate.

Compound **2a,** ^{1}H-NMR (500 MHz, $CDCl_3$)

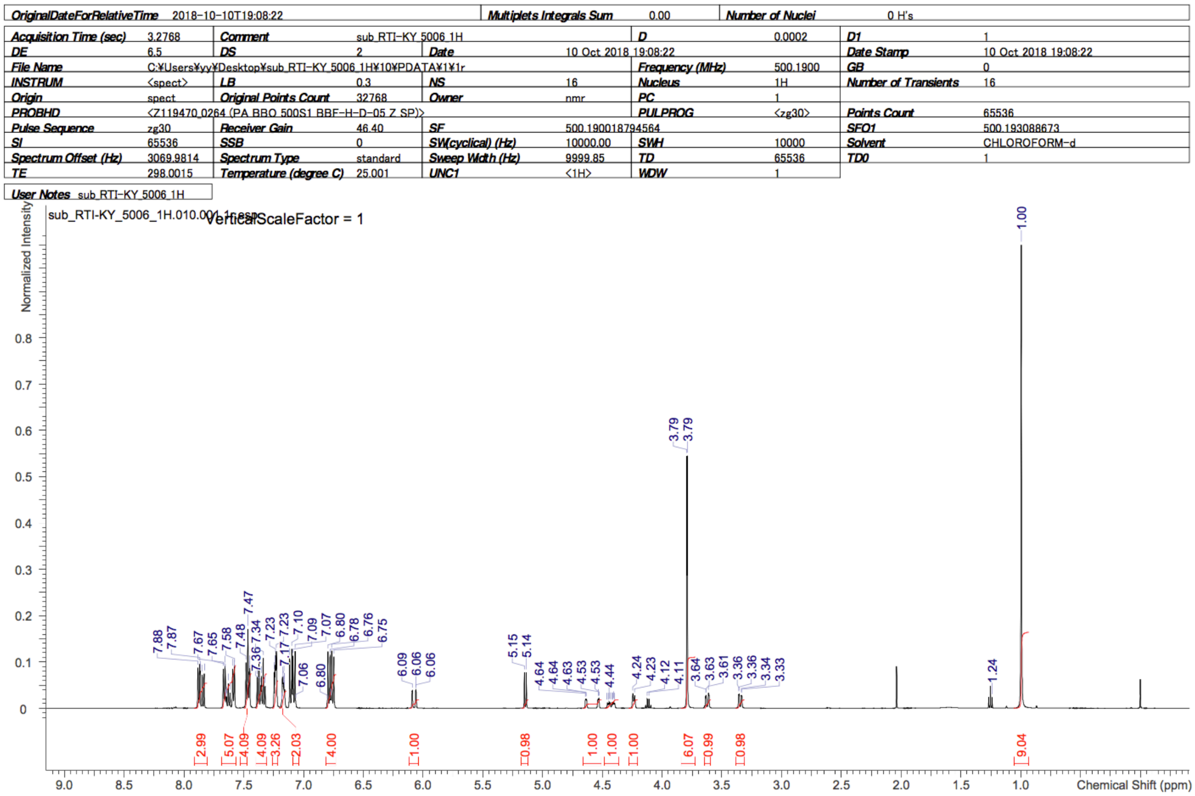

Compound **2a,** ^{13}C-NMR (126 MHz, $CDCl_3$)

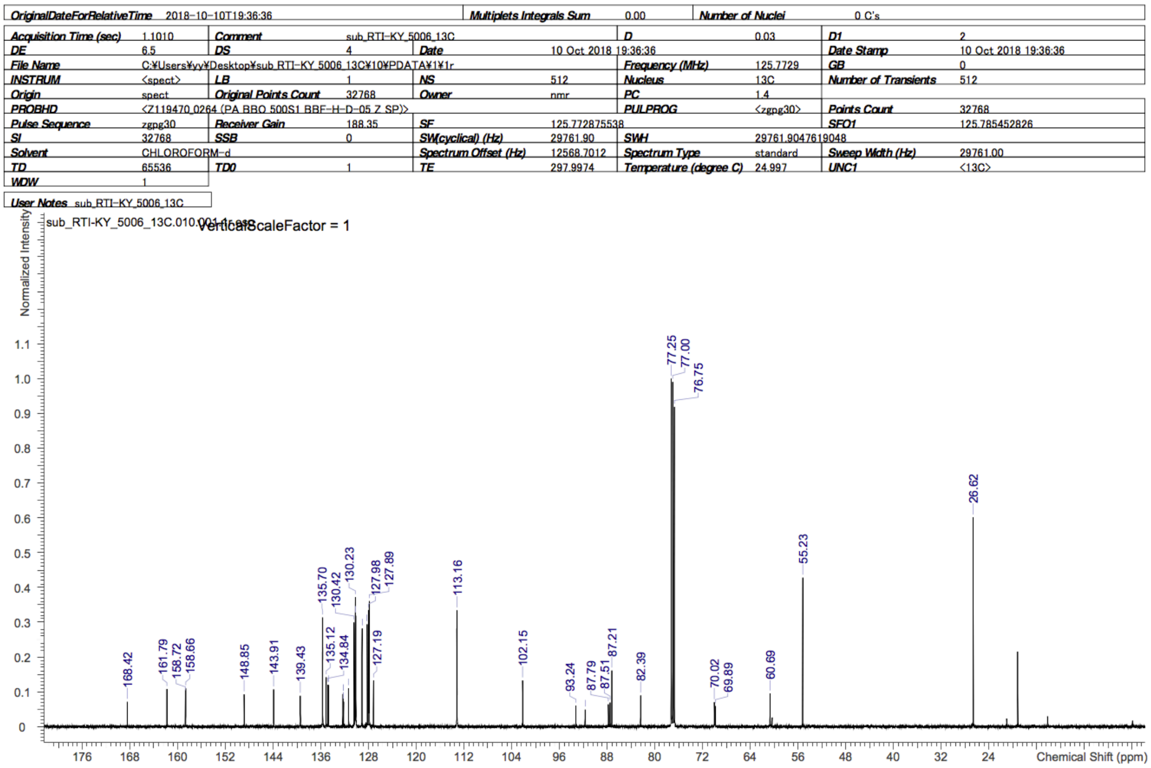

Compound **2a**, ^{19}F-NMR (470 MHz, $CDCl_3$)

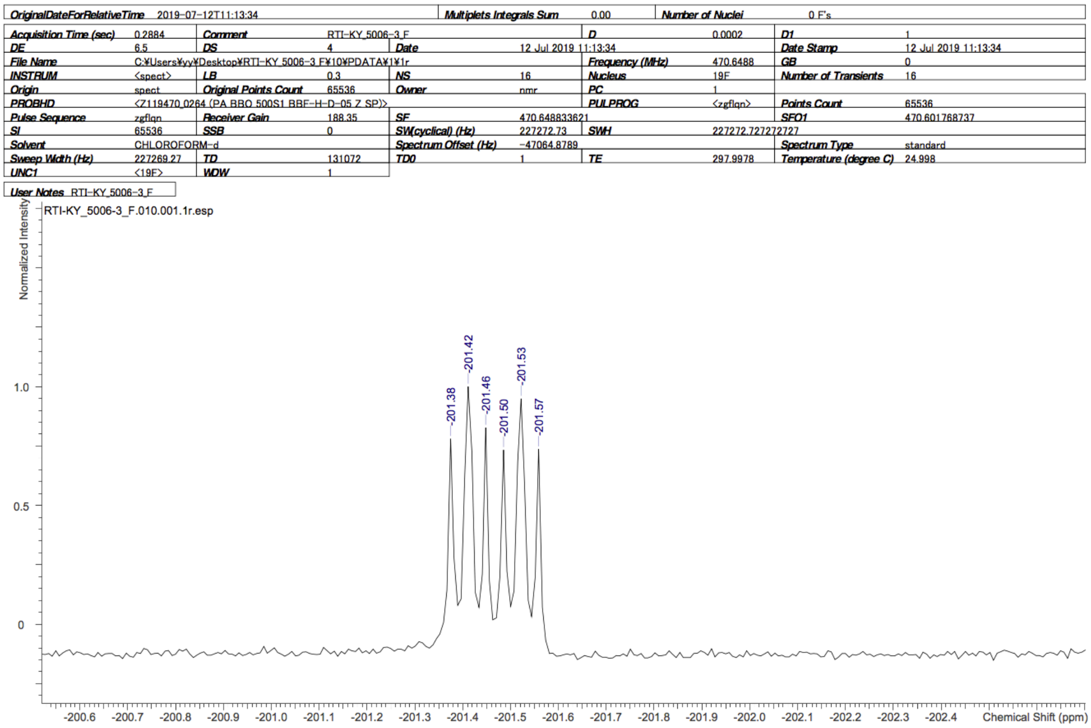

Compound **3a**, ^{1}H-NMR (500 MHz, $CDCl_3$)

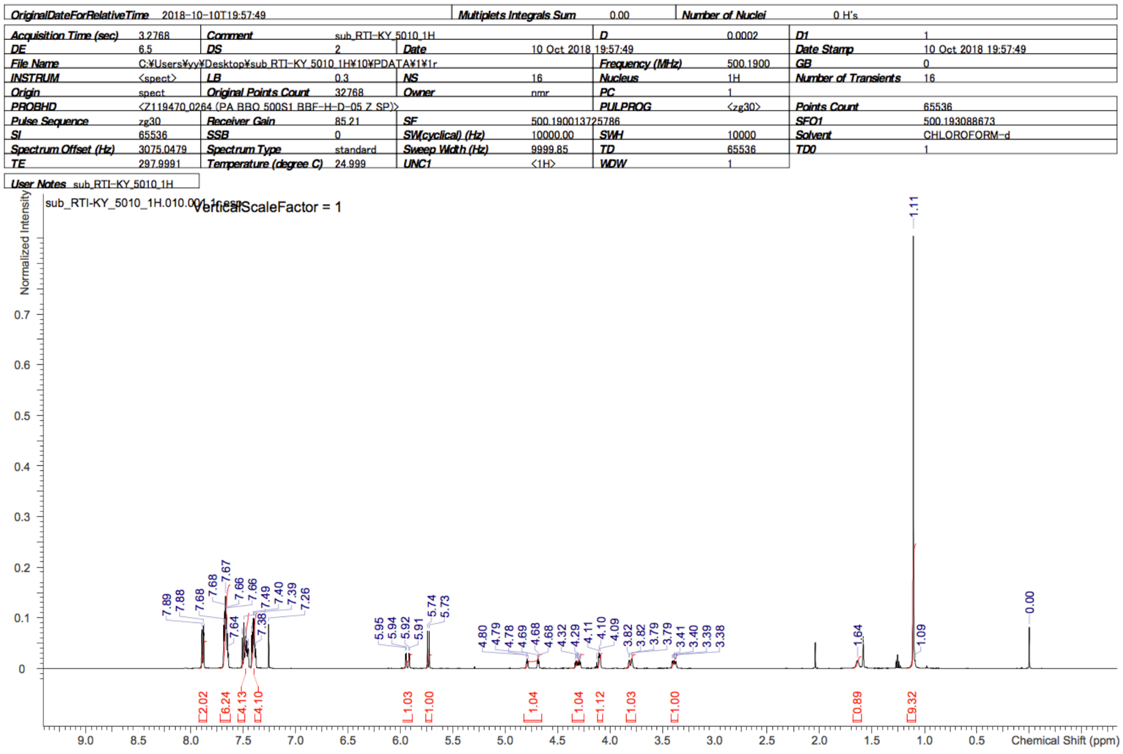

Compound **3a,** ^{13}C-NMR (126 MHz, $CDCl_3$)

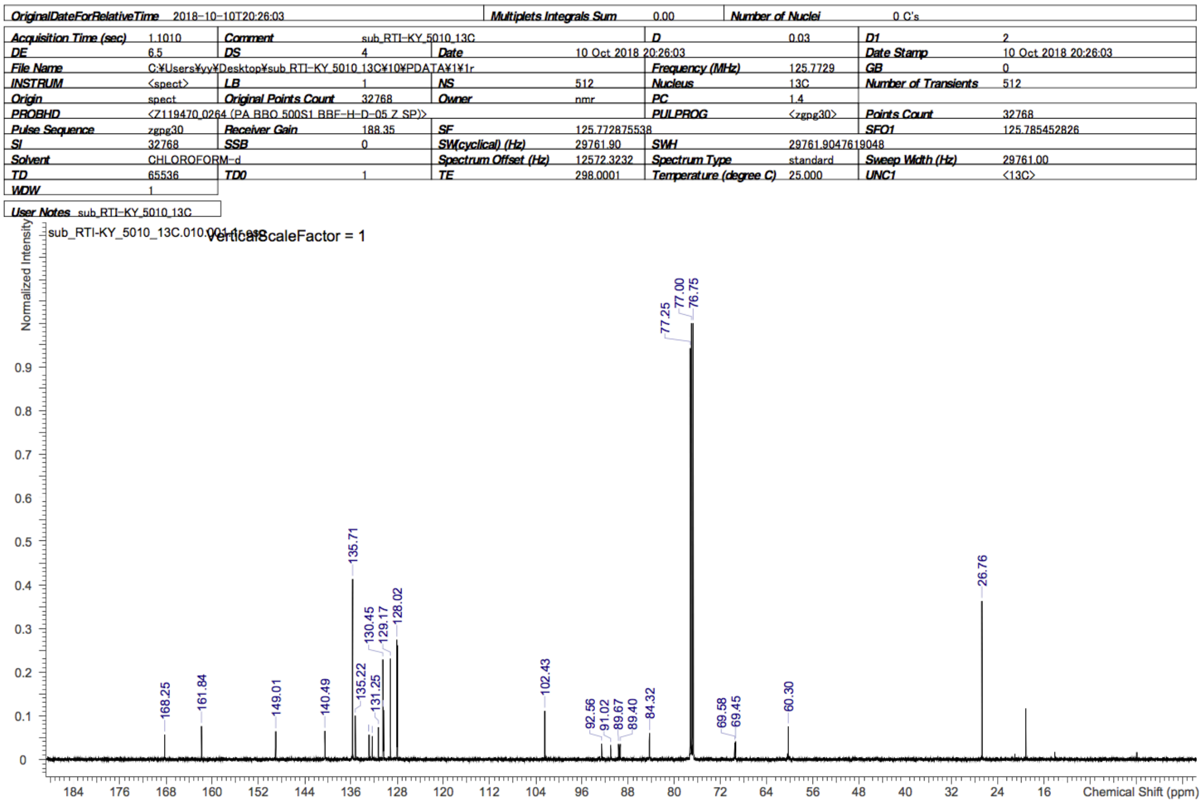

Compound **3a,** ^{19}F-NMR (470 MHz, CD_3CN-*d3*)

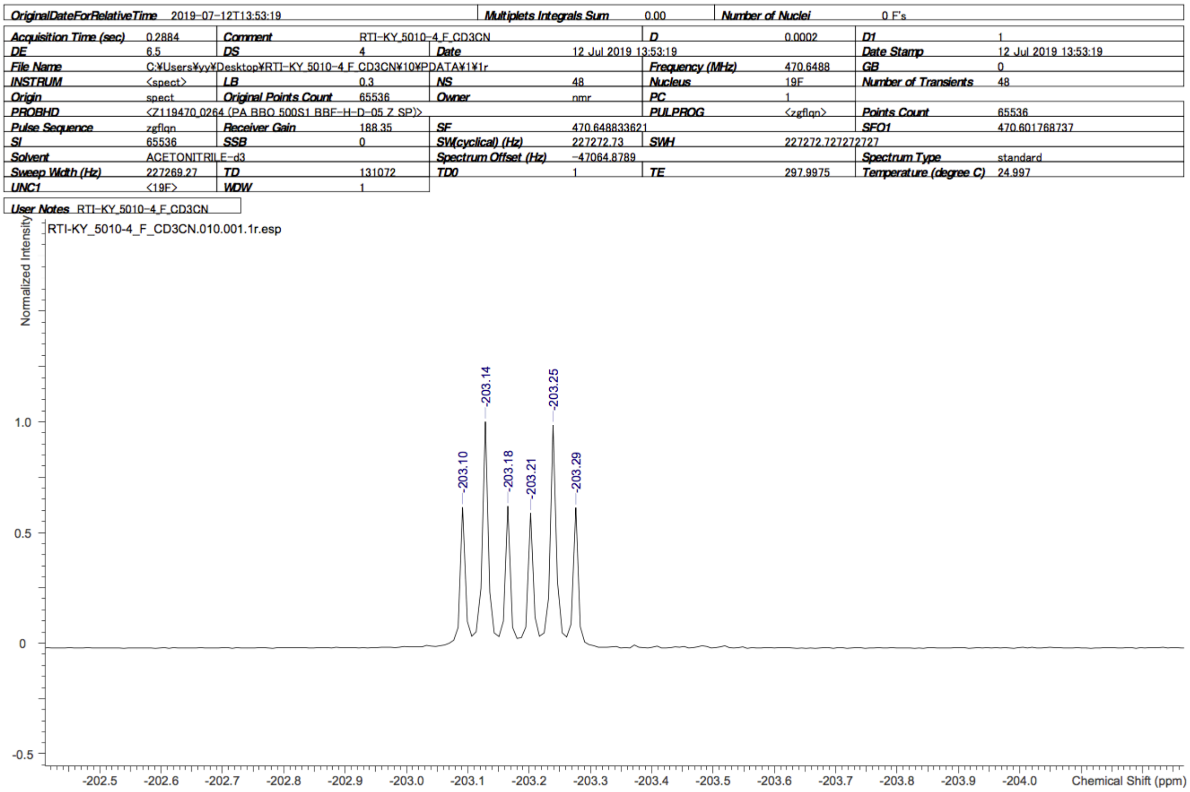

Compound **5a,** ^{1}H-NMR (500 MHz, $CDCl_3$)

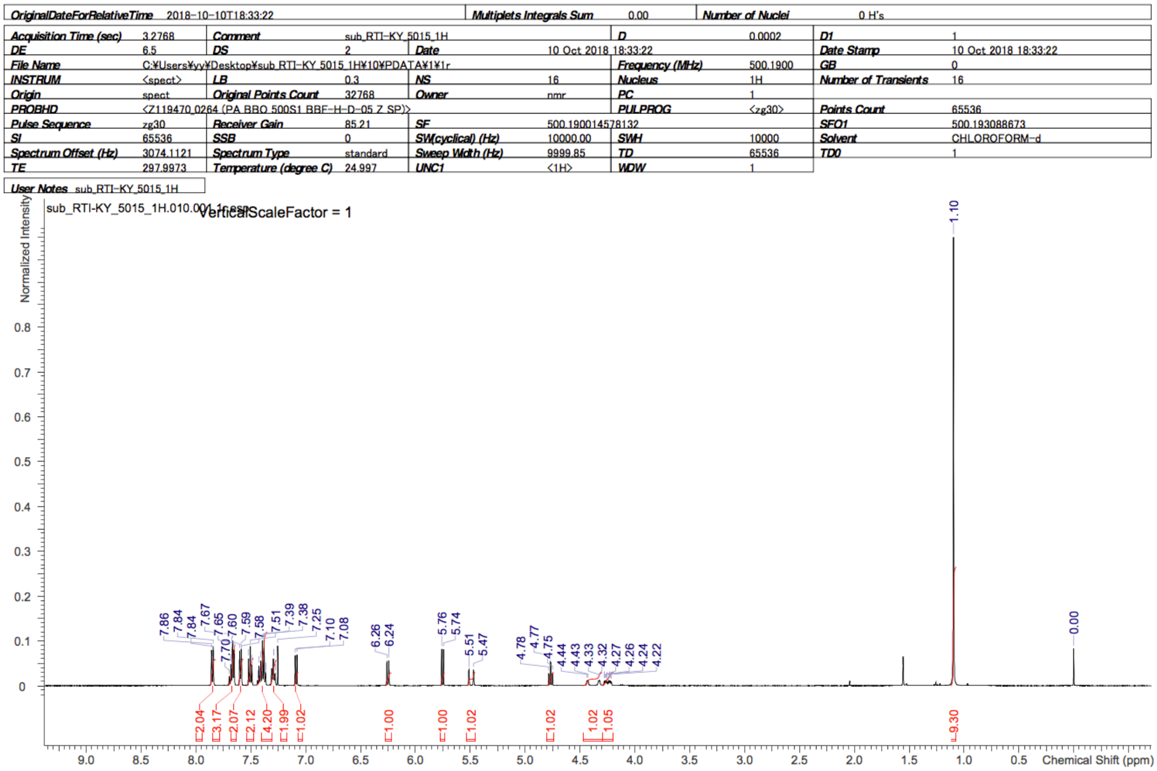

Compound **5a,** ^{13}C-NMR (126 MHz, $CDCl_3$)

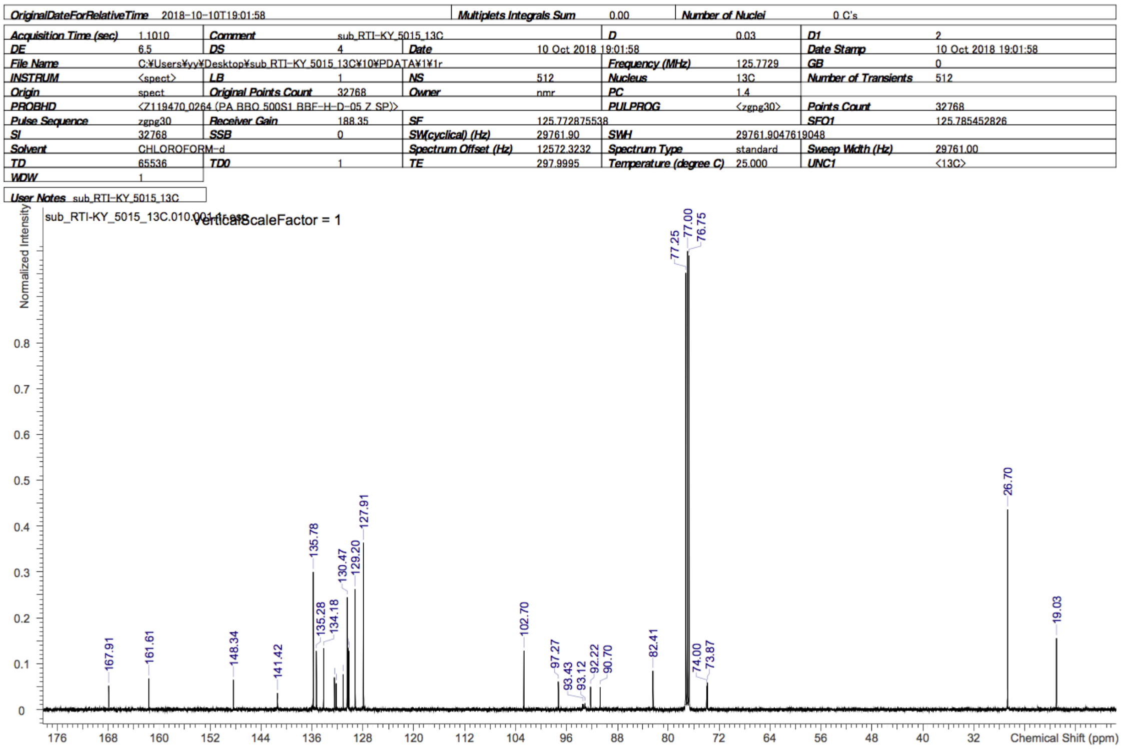

Compound **5a,** ^{19}F-NMR (470 MHz, CD_3CN-*d3*)

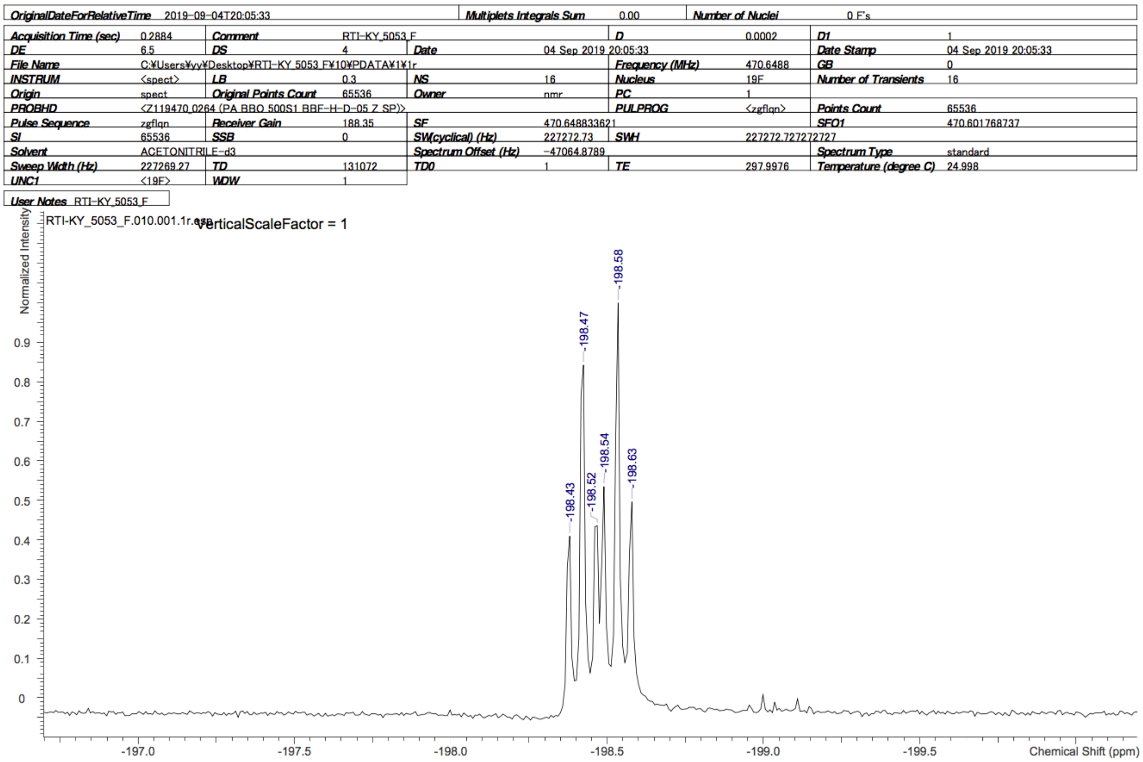

Compound **6a-E,** ^{1}H-NMR (500 MHz, $CDCl_3$)

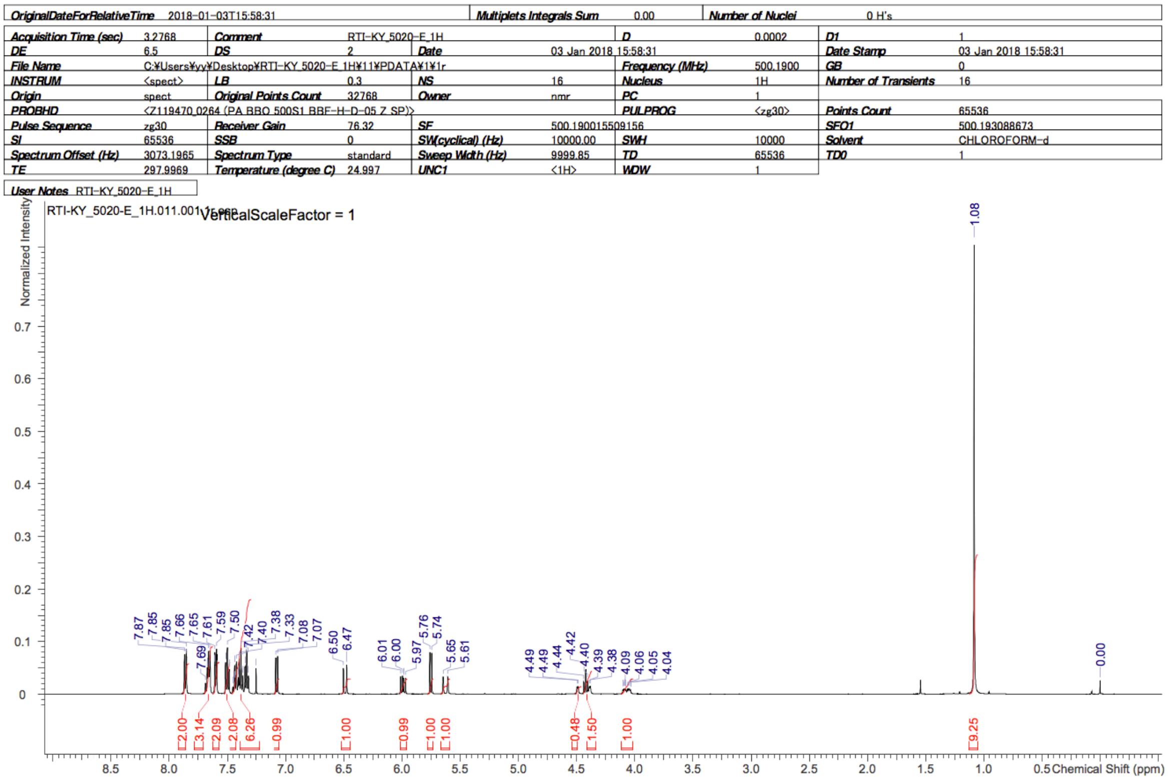

Compound **6a-E,** ^{13}C-NMR (126 MHz, $CDCl_3$)

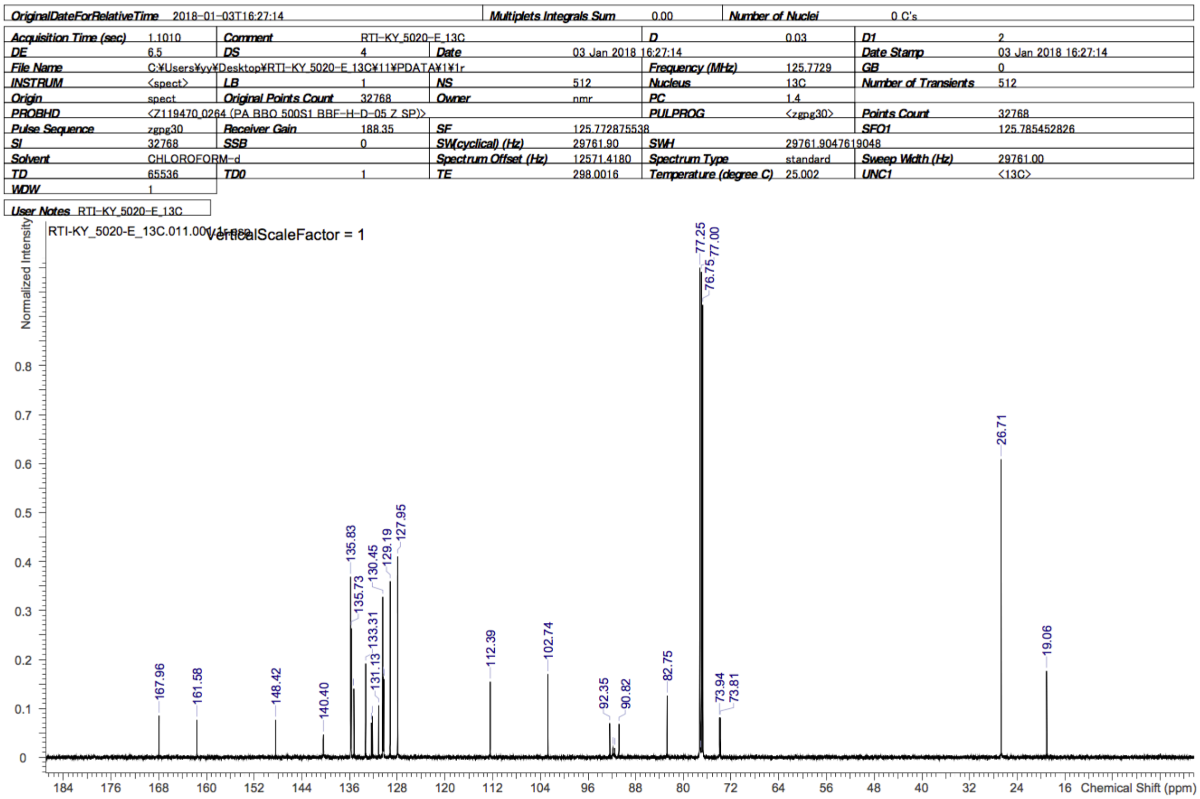

Compound **6a-E**, ^{19}F-NMR (470 MHz, CD_3CN-*d3*)

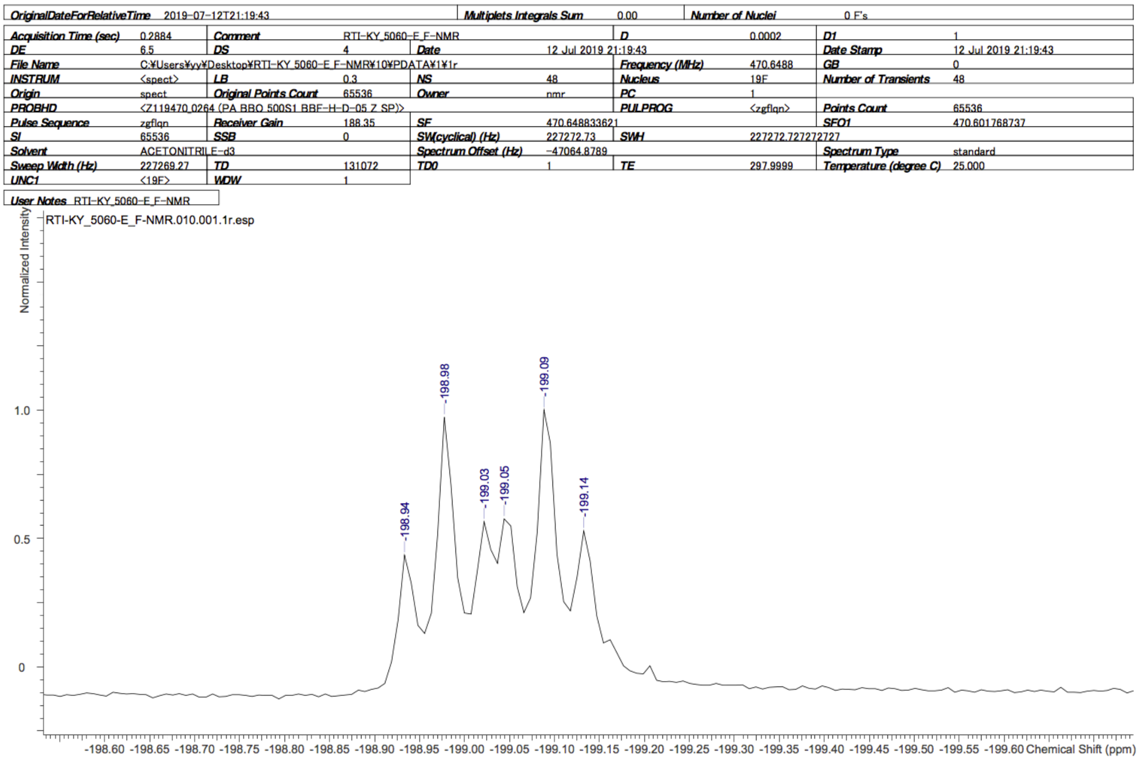

Compound **6a-Z**, ^{1}H-NMR (500 MHz, $CDCl_3$)

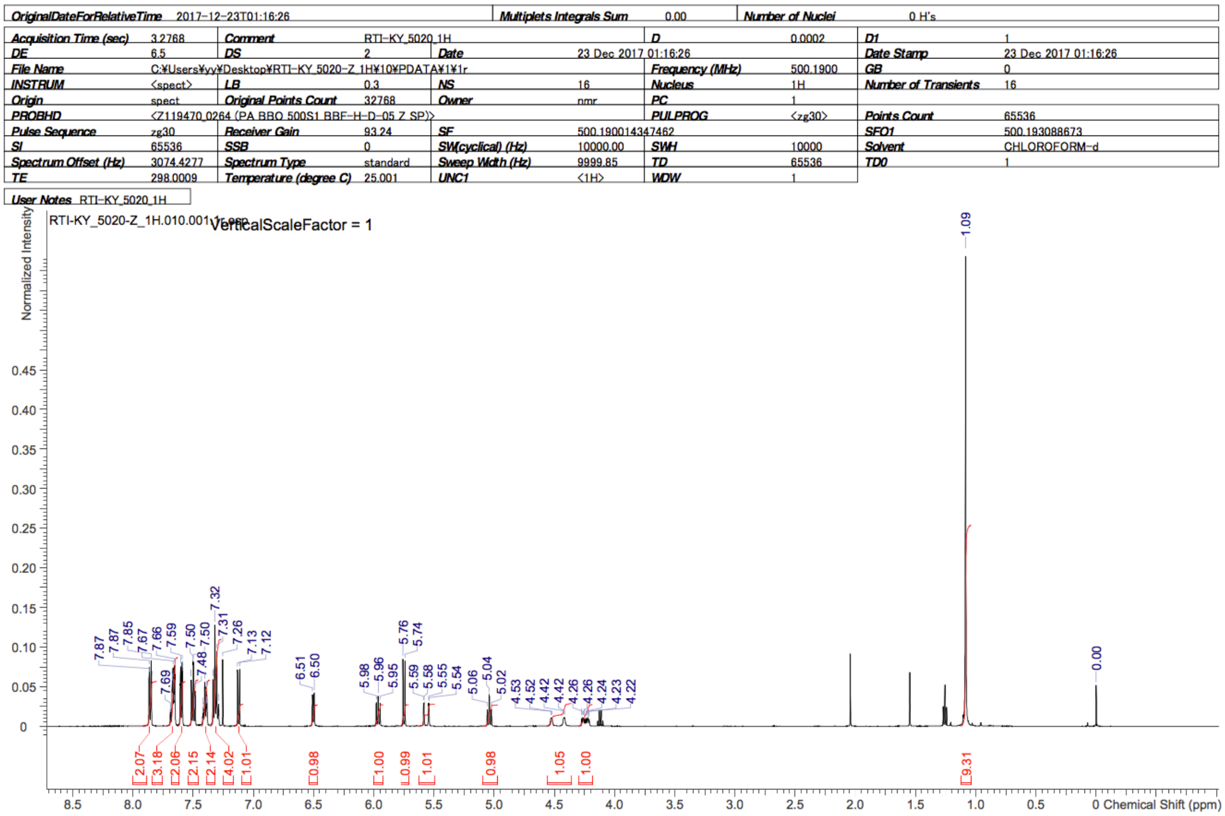

Compound **6a-Z**, ^{13}C-NMR (126 MHz, $CDCl_3$)

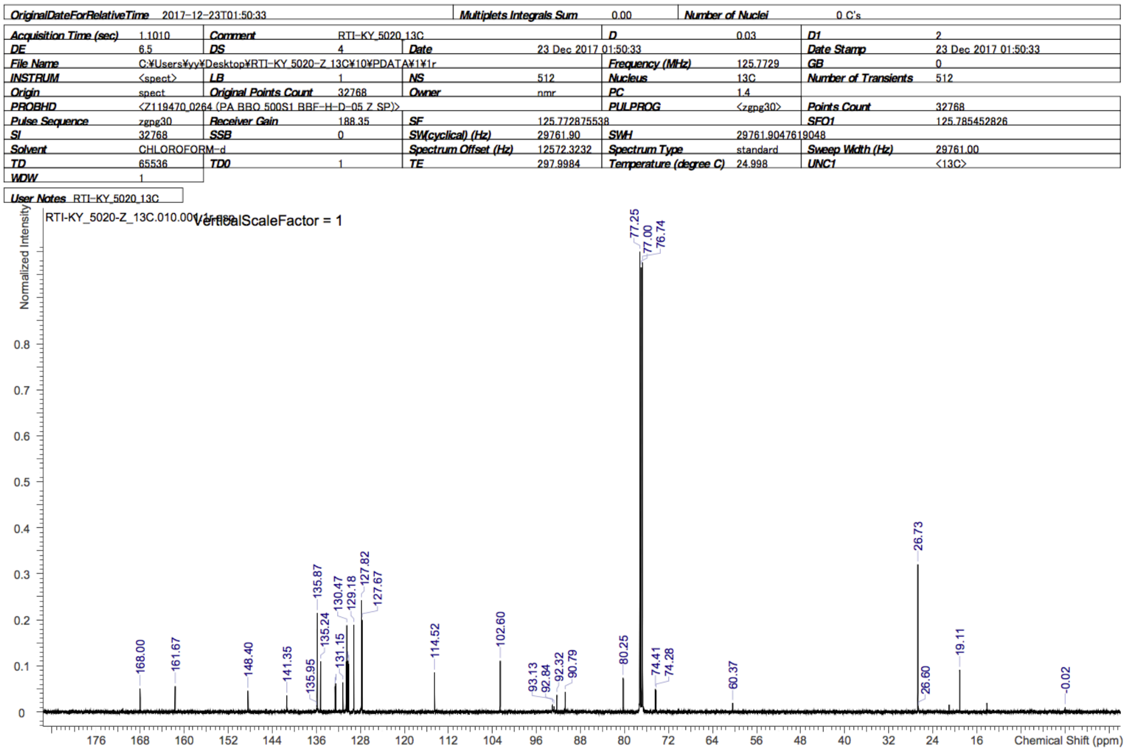

Compound **6a-Z**, ^{19}F-NMR (470 MHz, CD_3CN-*d3*)

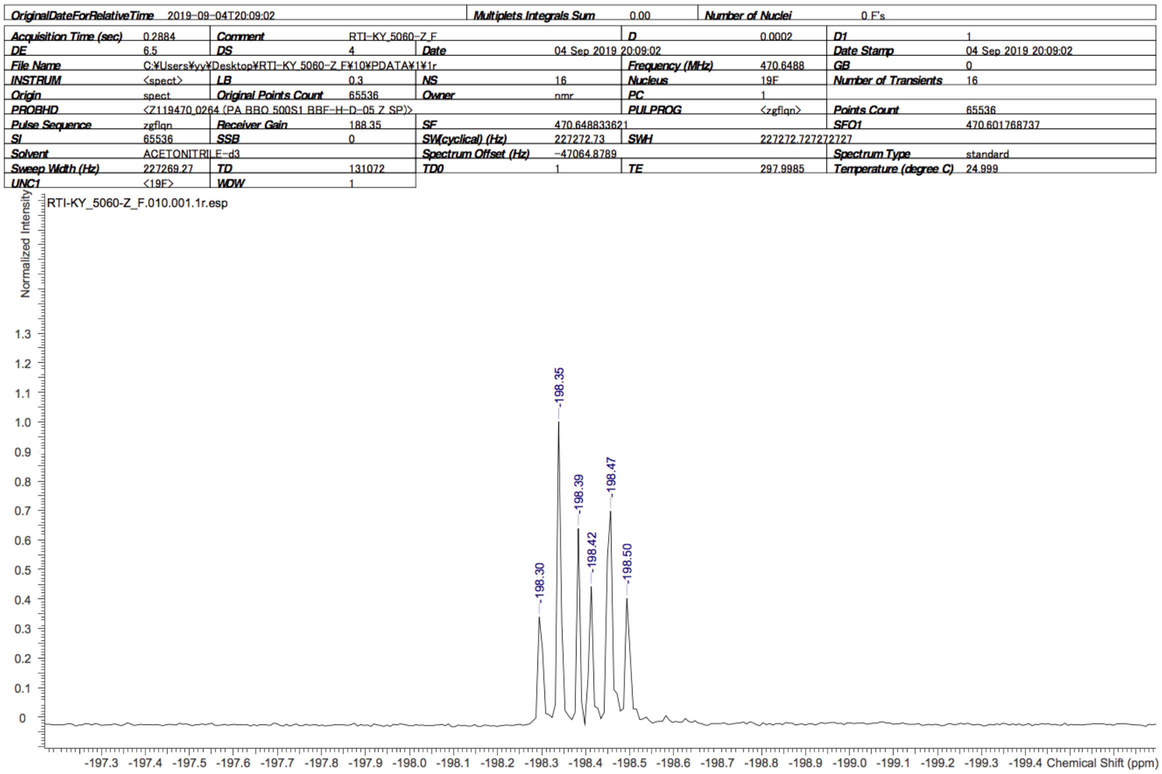

Compound **7a** (least polar isomer), ^{1}H-NMR (500 MHz, $CDCl_3$)

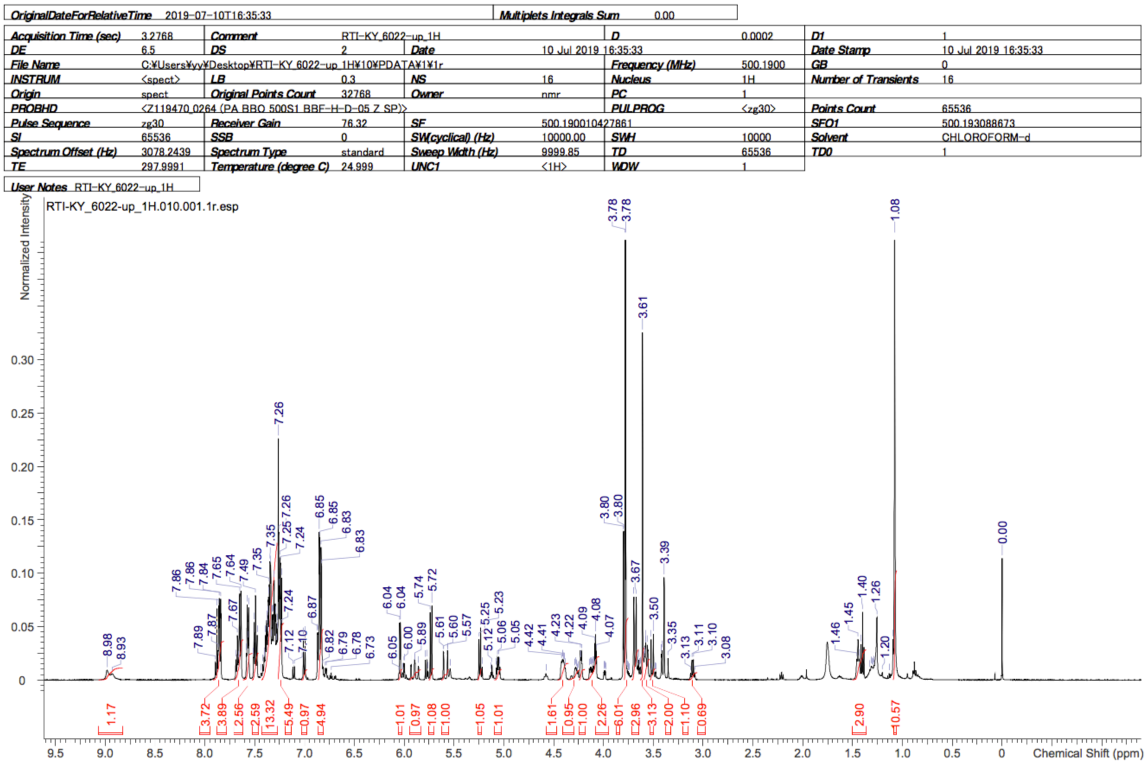

Compound **7a** (least polar isomer), ^{13}C-NMR (126 MHz, $CDCl_3$)

OriginalDateForRelativeTime	2019-07-11T04:49:42			Multiplets Integrals Sum	0.00	Number of Nuclei	0 C's
Acquisition Time (sec)	1.1010	Comment	RTI-KY_6022-up_13C	D	0.03	D1	2
DE	6.5	DS	4	Date	11 Jul 2019 04:49:42	Date Stamp	11 Jul 2019 04:49:42
File Name	C:¥Users¥yy¥Desktop¥RTI-KY_6022-up_13C¥10¥PDATA¥1¥1r			Frequency (MHz)	125.7729	GB	0
INSTRUM	<spect>	LB	1	NS	3072	Nucleus	13C
Number of Transients	3072						
Origin	spect	Original Points Count	32768	Owner	nmr	PC	1.4
PROBHD	<Z119470_0264 (PA BBO 500S1 BBF-H-D-05 Z SP)>			PULPROG	<zgpg30>	Points Count	32768
Pulse Sequence	zgpg30	Receiver Gain	188.35	SF	125.77287696399	SFO1	125.785452826
SI	32768	SSB	0	SW(cyclical) (Hz)	29761.90	SWH	29761.9047619048
Solvent	CHLOROFORM-d			Spectrum Offset (Hz)	12571.4199	Spectrum Type	standard
Sweep Width (Hz)	29761.00						
TD	65536	TD0	1	TE	297.9998	Temperature (degree C)	25.000
UNC1	<13C>						
WDW	1						
User Notes	RTI-KY_6022-up_13C						

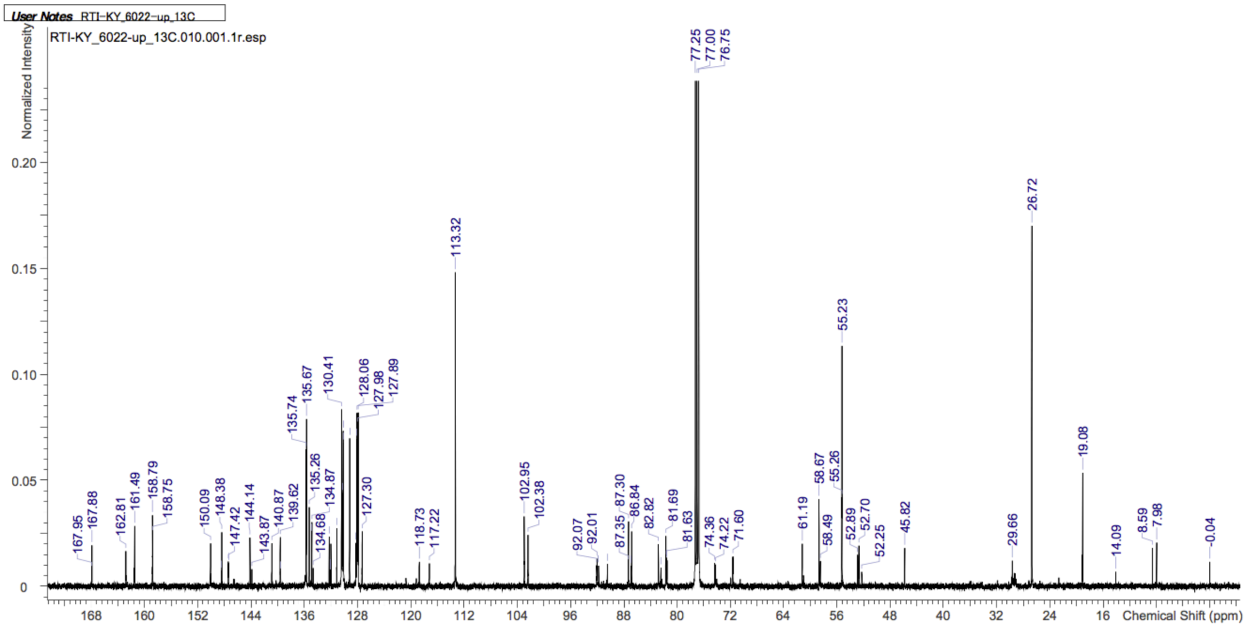

Compound **7a** (least polar isomer), ^{19}F-NMR (470 MHz, CD_3CN-*d3*)

OriginalDateForRelativeTime	2019-07-13T16:43:58			Multiplets Integrals Sum	0.00	Number of Nuclei	0 F's
Acquisition Time (sec)	0.2884	Comment	RTI-KY_6022_up_F_CD3CN	D	0.0002	D1	1
DE	6.5	DS	4	Date	13 Jul 2019 16:43:58	Date Stamp	13 Jul 2019 16:43:58
File Name	C:¥Users¥yy¥Desktop¥RTI-KY_6022_up_F_CD3CN¥11¥PDATA¥1¥1r			Frequency (MHz)	470.6488	GB	0
INSTRUM	<spect>	LB	0.3	NS	48	Nucleus	19F
Number of Transients	48						
Origin	spect	Original Points Count	65536	Owner	nmr	PC	1
PROBHD	<Z119470_0264 (PA BBO 500S1 BBF-H-D-05 Z SP)>	PULPROG	<zgflqn>	Points Count	65536	Pulse Sequence	zgflqn
Receiver Gain	188.35	SF	470.648833621	SFO1	470.601768737		
SI	65536	SSB	0	SW(cyclical) (Hz)	227272.73	SWH	227272.727272727
Solvent	ACETONITRILE-d3	Spectrum Offset (Hz)	-47064.8789	Spectrum Type	standard		
Sweep Width (Hz)	227269.27	TD	131072	TD0	1	TE	298.0001
Temperature (degree C)	25.000						
UNC1	<19F>	WDW	1				
User Notes	RTI-KY_6022_up_F_CD3CN						

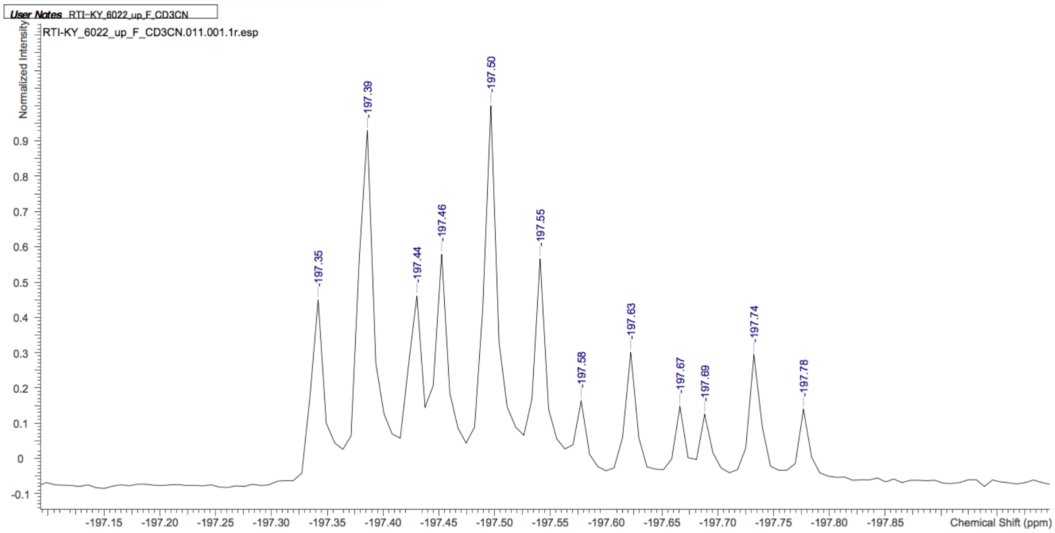

Compound **7a** (least polar isomer), ^{31}P-NMR (202 MHz, $CDCl_3$)

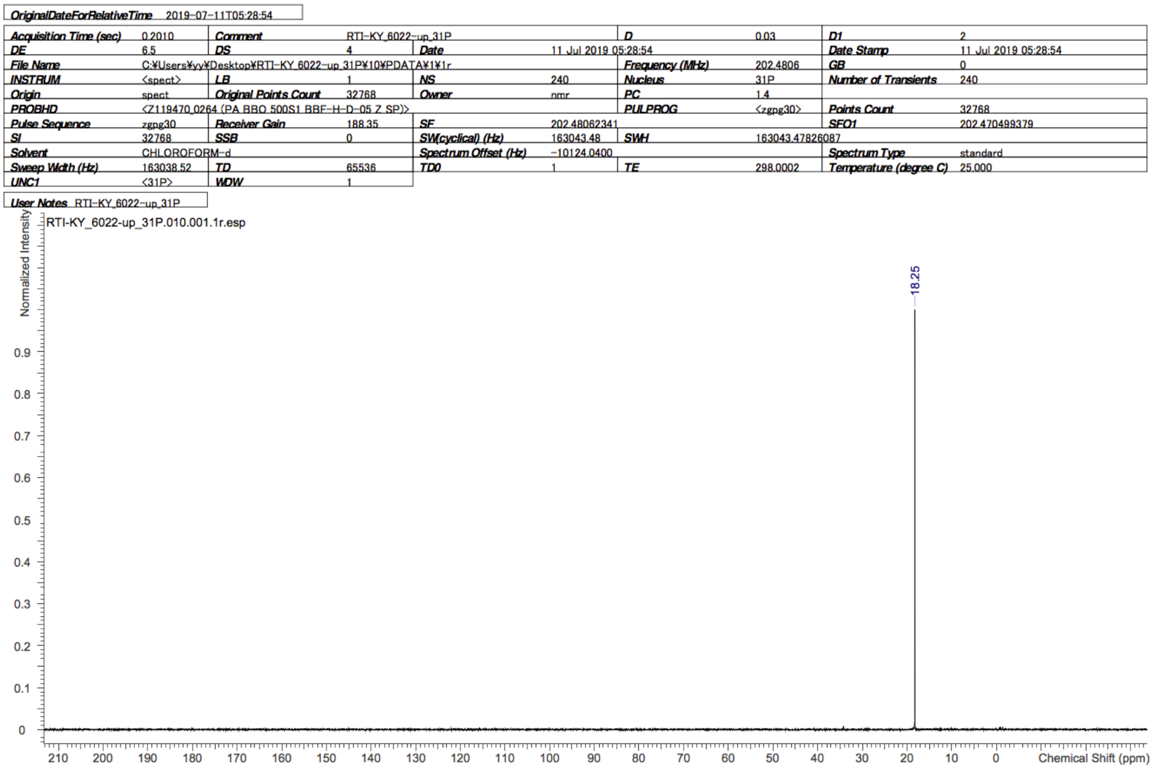

Compound **7a** (most polar isomer), ^{1}H-NMR (500 MHz, $CDCl_3$)

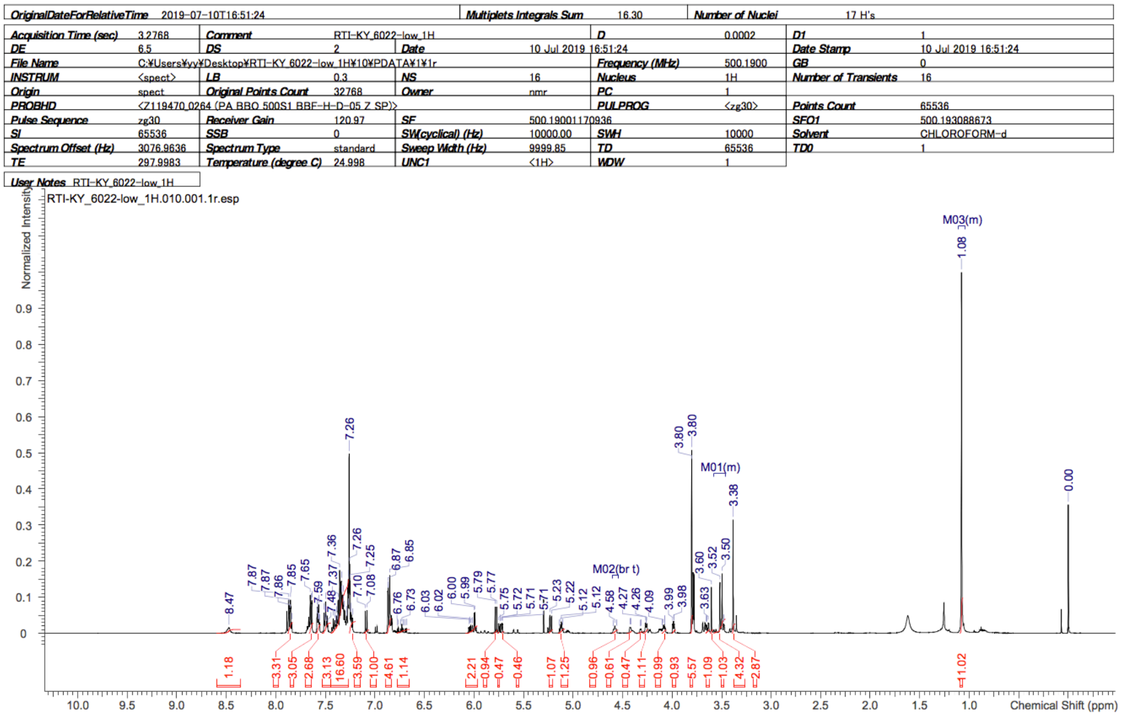

Compound **7a** (most polar isomer), ^{13}C-NMR (126 MHz, $CDCl_3$)

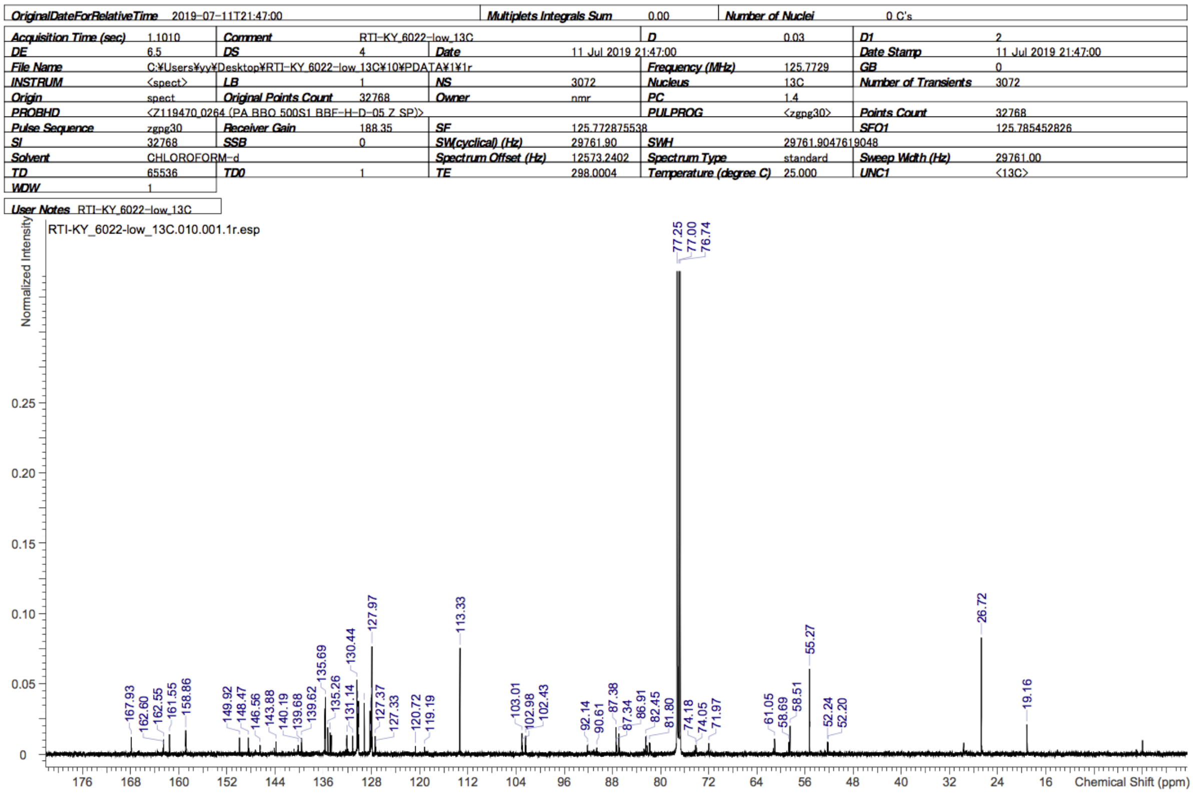

Compound **7a** (most polar isomer), ^{19}F-NMR (470 MHz, CD_3CN-*d3*)

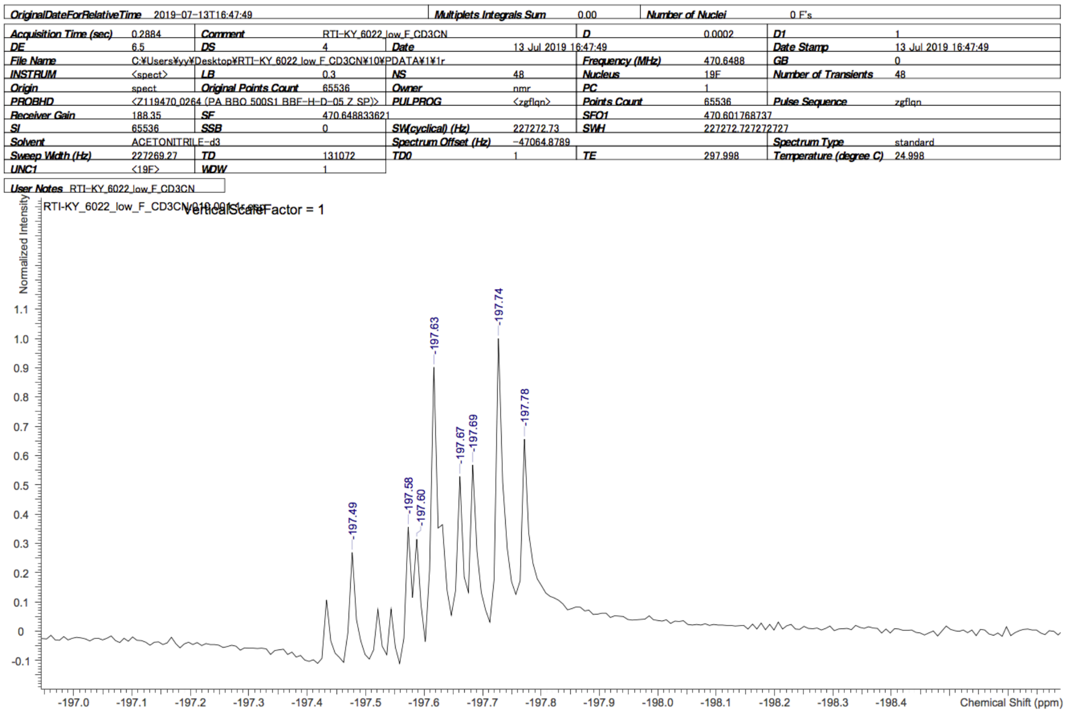

Compound **7a** (most polar isomer), ^{31}P-NMR (202 MHz, $CDCl_3$)

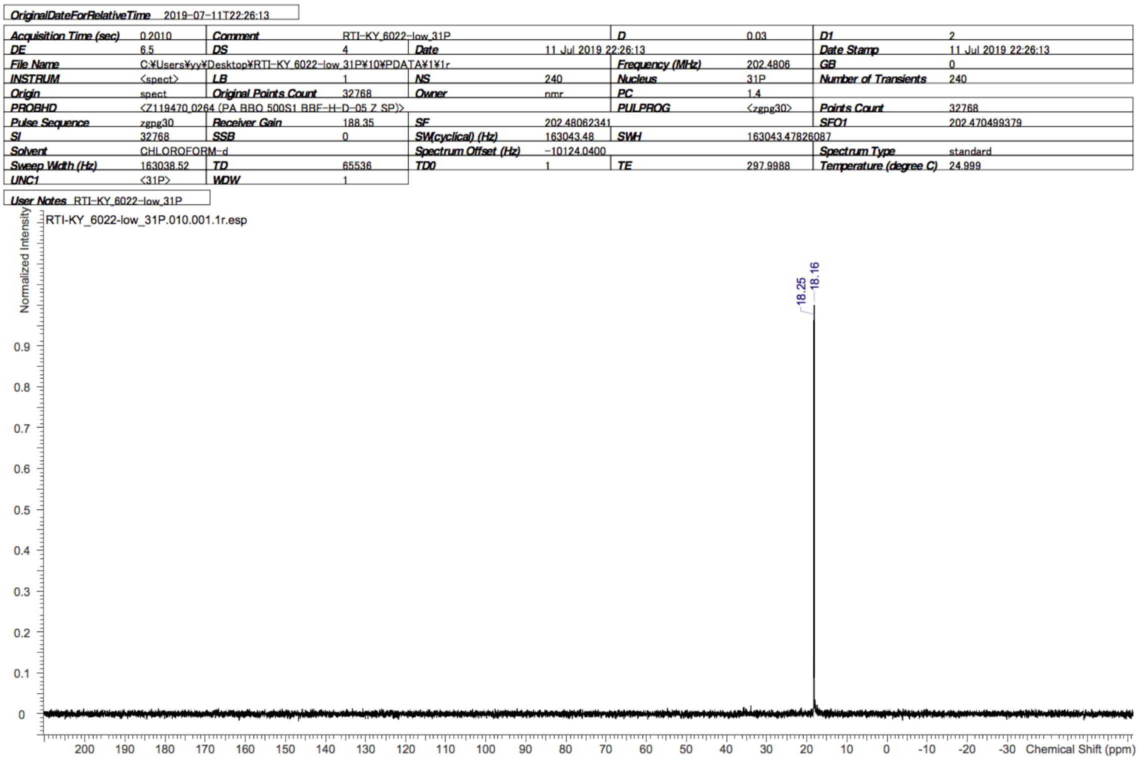

Compound **8a** (least polar isomer), ^{1}H-NMR (500 MHz, CD_3CN-d_3)

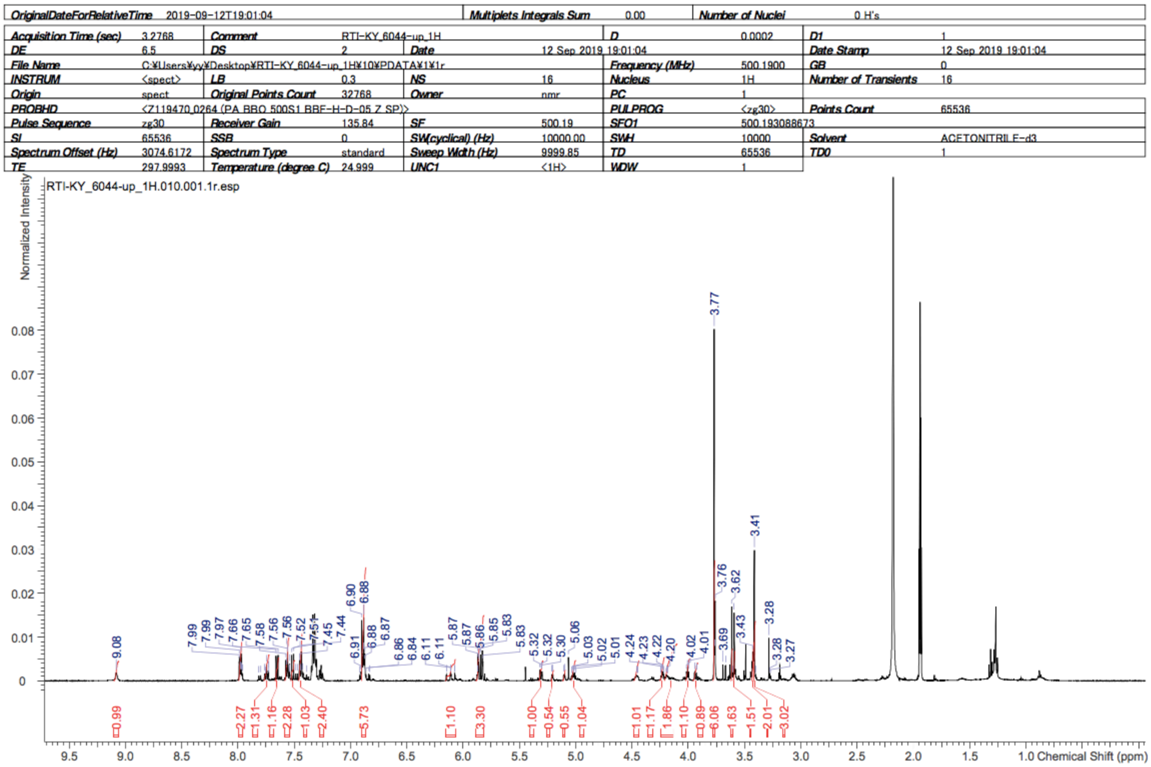

Compound **8a** (least polar isomer), ^{13}C-NMR (126 MHz, CD_3CN-d_3)

OriginalDateForRelativeTime	2019-09-13T04:07:23			*Multiplets Integrals Sum*	0.00	*Number of Nuclei*	0 C's		
Acquisition Time (sec)	1.1010	*Comment*	RTI-KY 6044-up 13C			*D*	0.03	*D1*	2
DE	6.5	*DS*	4	*Date*	13 Sep 2019 04:07:23			*Date Stamp*	13 Sep 2019 04:07:23
File Name	C:¥Users¥yy¥Desktop¥RTI-KY 6044-up 13C¥10¥PDATA¥1¥1r					*Frequency (MHz)*	125.7728	*GB*	0
INSTRUM	<spect>	*LB*	1	*NS*	6144	*Nucleus*	13C	*Number of Transients*	6144
Origin	spect	*Original Points Count*	32768	*Owner*	nmr	*PC*	1.4		
PROBHD	<Z119470_0264 (PA BBO 500S1 BBF-H-D-05 Z SP)>					*PULPROG*	<zgpg30>	*Points Count*	32768
Pulse Sequence	zgpg30	*Receiver Gain*	188.35	*SF*	125.772803746154			*SFO1*	125.785452826
SI	32768	*SSB*	0	*SW(cyclical) (Hz)*	29761.90	*SWH*	29761.9047619048		
Solvent	ACETONITRILE-d3			*Spectrum Offset (Hz)*	12702.2939	*Spectrum Type*	standard	*Sweep Width (Hz)*	29761.00
TD	65536	*TD0*	1	*TE*	298.0002	*Temperature (degree C)*	25.000	*UNC1*	<13C>
WDW	1								

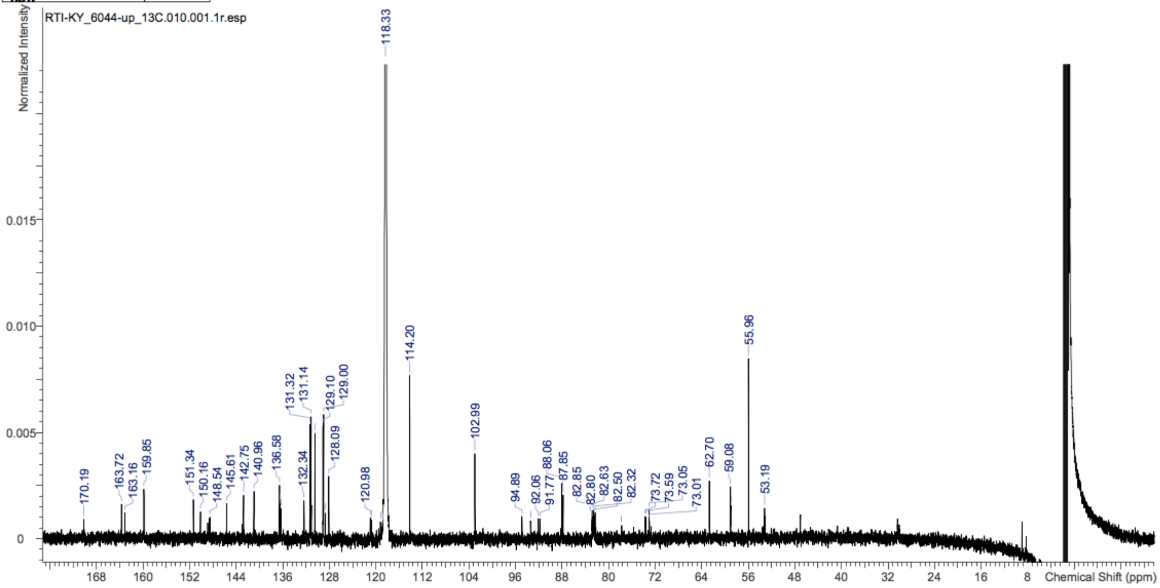

Compound **8a** (least polar isomer), ^{19}F-NMR (470 MHz, CD_3CN-d_3)

OriginalDateForRelativeTime	2019-09-12T22:12:55			*Multiplets Integrals Sum*	0.00	*Number of Nuclei*	0 F's		
Acquisition Time (sec)	0.5767	*Comment*	RTI-KY 6044-up F			*D*	0.0002	*D1*	1
DE	6.5	*DS*	4	*Date*	12 Sep 2019 22:12:55			*Date Stamp*	12 Sep 2019 22:12:55
File Name	C:¥Users¥yy¥Desktop¥RTI-KY 6044-up F¥10¥PDATA¥1¥1r					*Frequency (MHz)*	470.6488	*GB*	0
INSTRUM	<spect>	*LB*	0.3	*NS*	240	*Nucleus*	19F	*Number of Transients*	240
Origin	spect	*Original Points Count*	65536	*Owner*	nmr	*PC*	1		
PROBHD	<Z119470_0264 (PA BBO 500S1 BBF-H-D-05 Z SP)>					*PULPROG*	<zgflqn>	*Points Count*	65536
Pulse Sequence	zgflqn	*Receiver Gain*	188.35	*SF*	470.648833621			*SFO1*	470.601768737
SI	65536	*SSB*	0	*SW(cyclical) (Hz)*	113636.37	*SWH*	113636.363636364		
Solvent	ACETONITRILE-d3			*Spectrum Offset (Hz)*	-47064.8867			*Spectrum Type*	standard
Sweep Width (Hz)	113634.63	*TD*	131072	*TD0*	1	*TE*	297.9994	*Temperature (degree C)*	24.999
UNC1	<19F>	*WDW*	1						

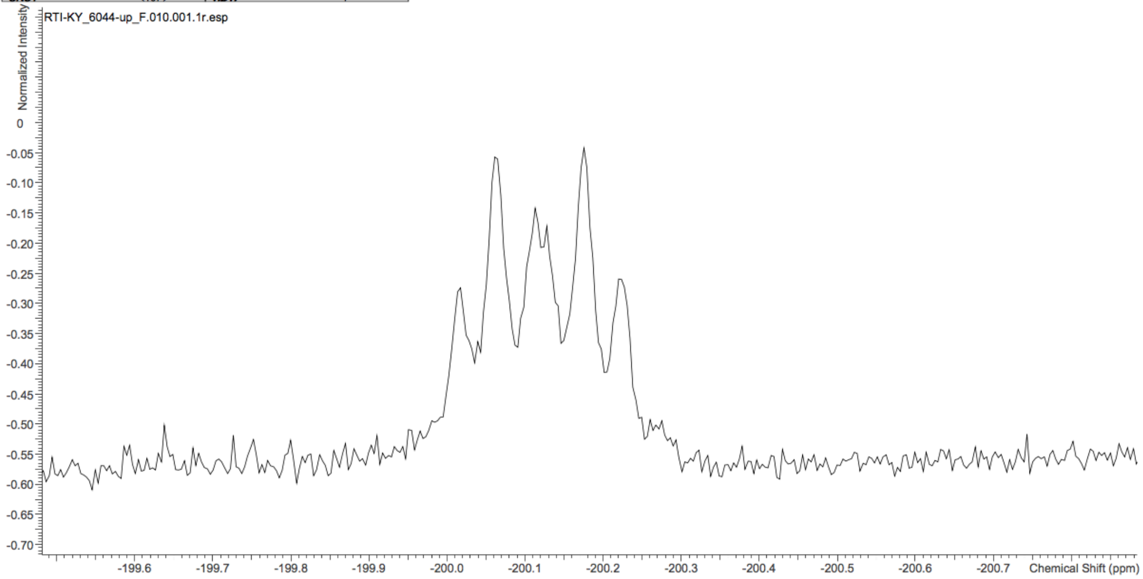

Compound **8a** (least polar isomer), ^{31}P-NMR (202 MHz, CD_3CN-d_3)

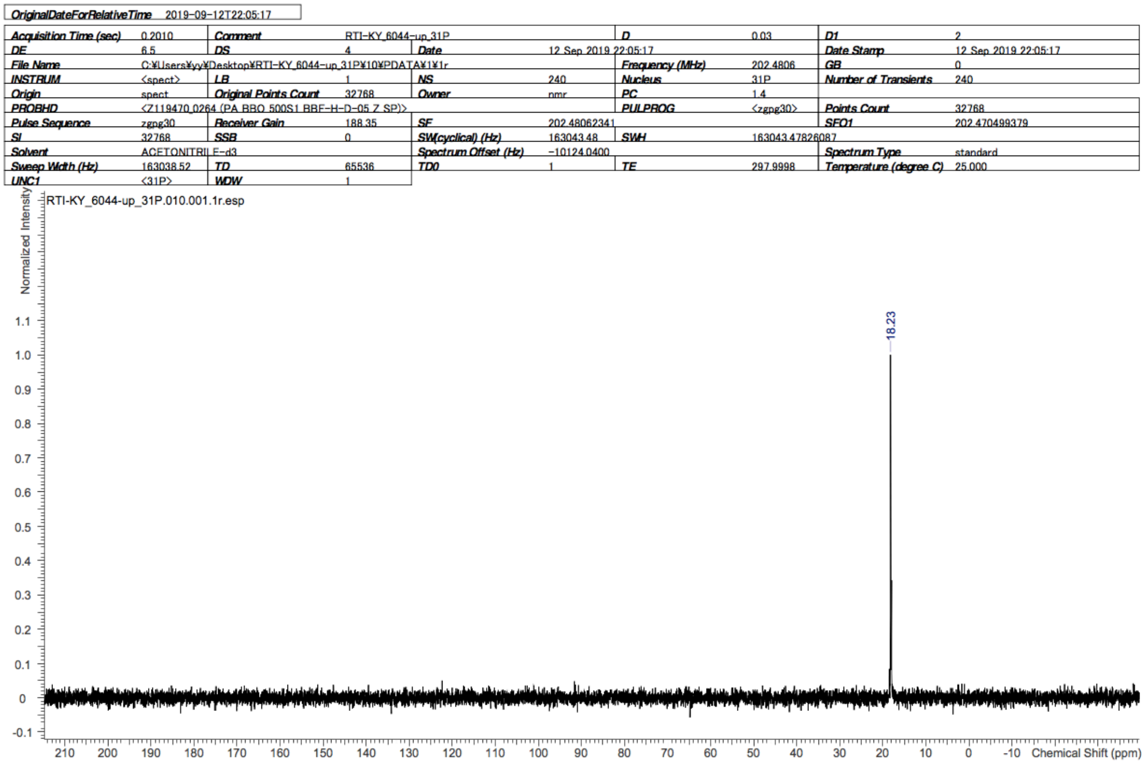

Compound **8a** (most polar isomer), ^{1}H-NMR (500 MHz, CD_3CN-d_3)

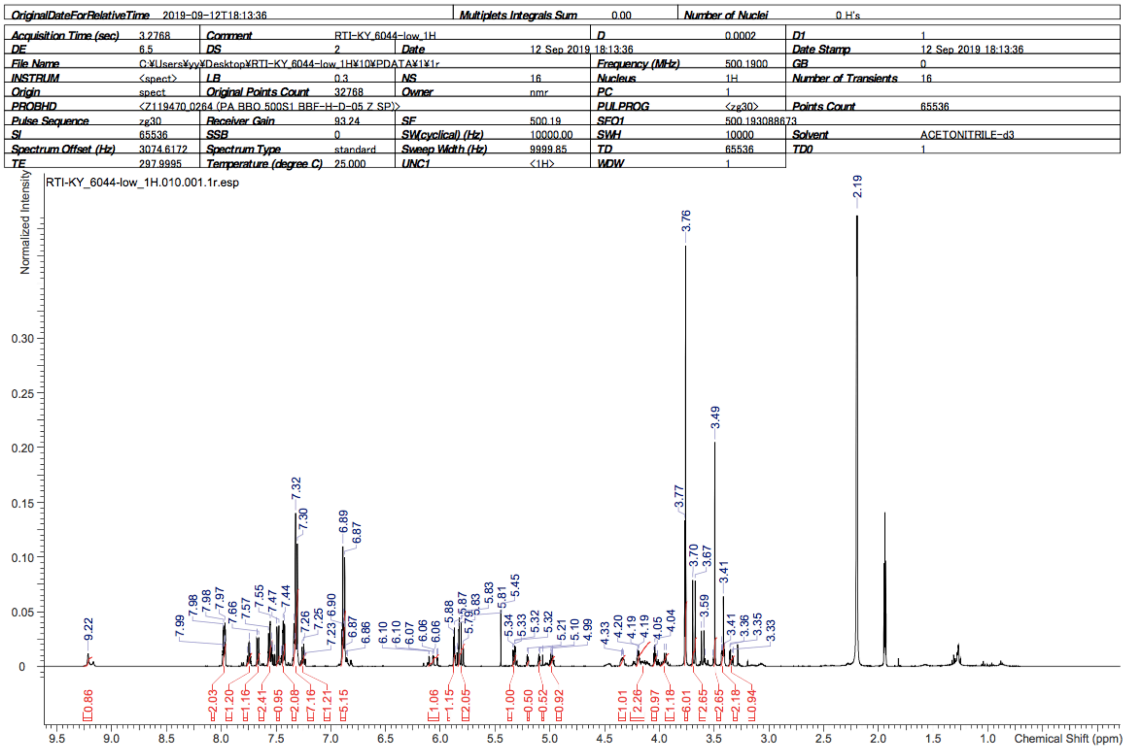

Compound **8a** (most polar isomer), ^{13}C-NMR (126 MHz, CD_3CN-d_3)

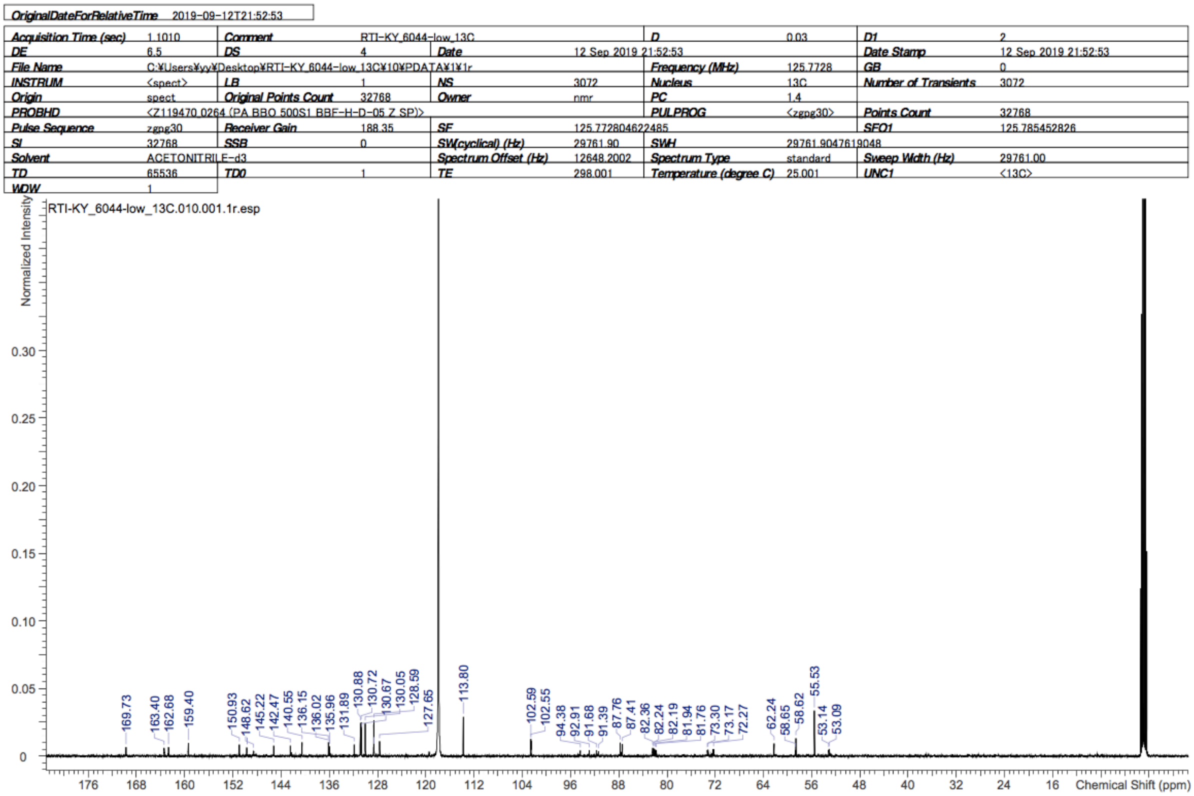

Compound **8a** (most polar isomer), ^{19}F-NMR (470 MHz, CD_3CN-d_3)

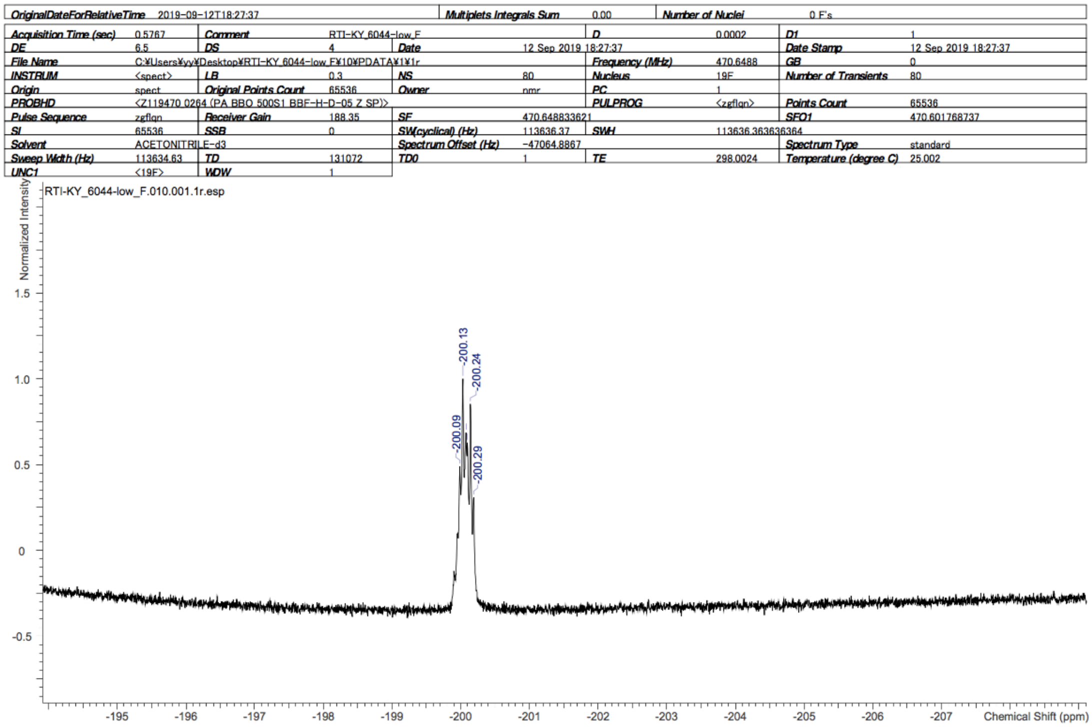

Compound **8a** (most polar isomer), ^{31}P-NMR (202 MHz, CD_3CN-d_3)

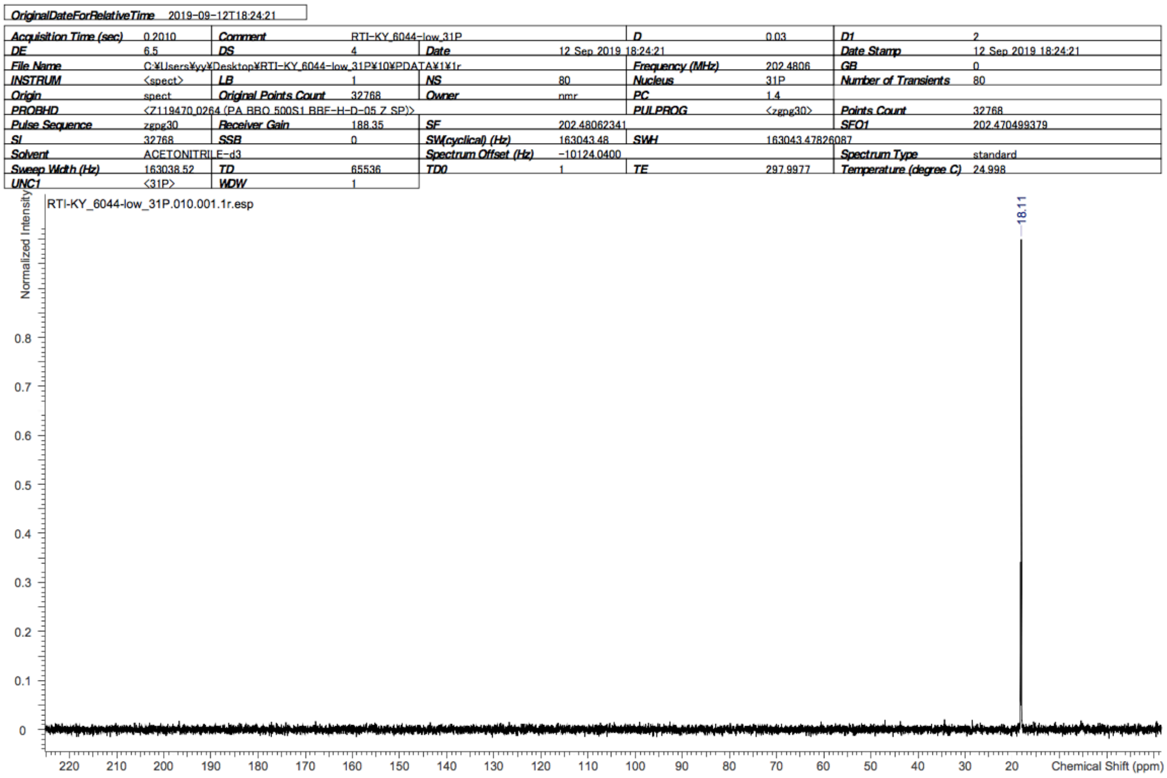

Compound **9a**, ^{31}P-NMR (202 MHz, $CDCl_3$)

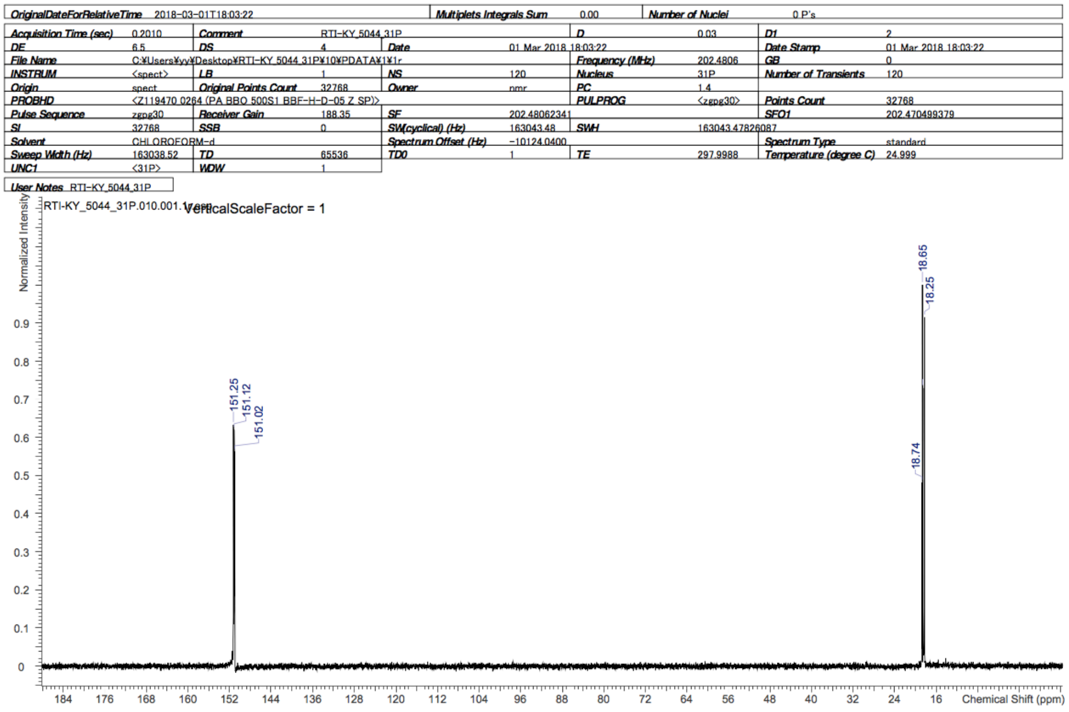

Compound **3b**, ^{1}H-NMR (500 MHz, DMSO-d_6)

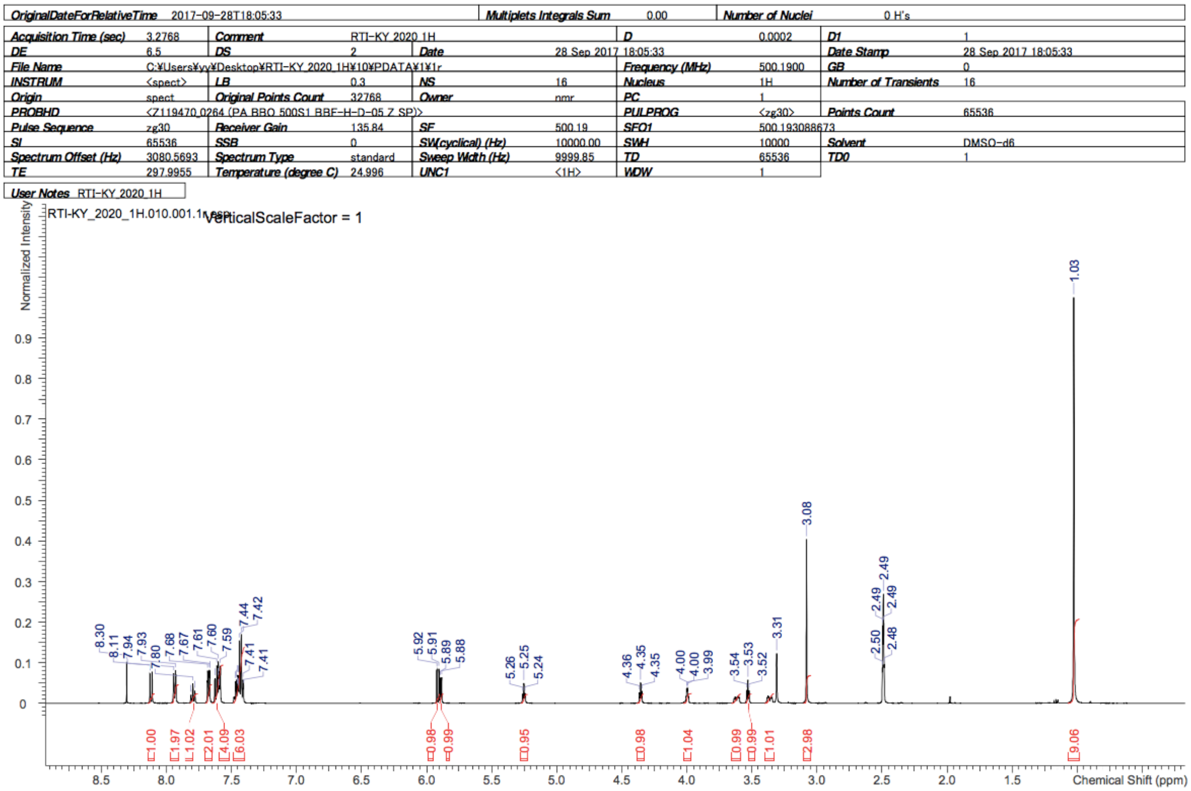

Compound **3b**, ^{13}C-NMR (126 MHz, DMSO-d_6)

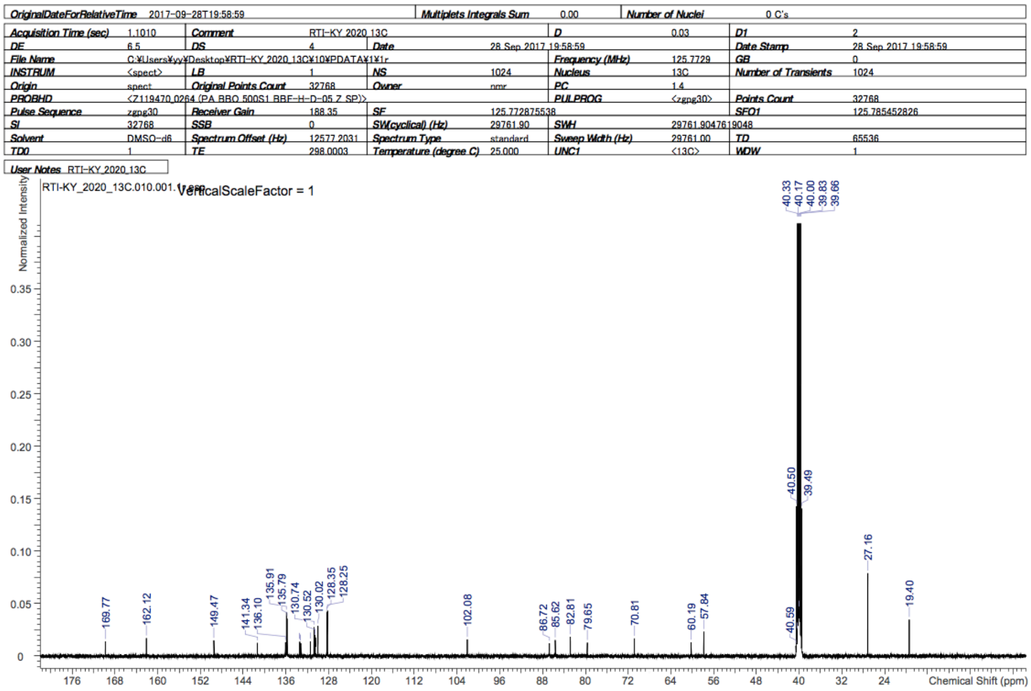

Compound **5b**, ^{1}H-NMR (500 MHz, CDCl3)

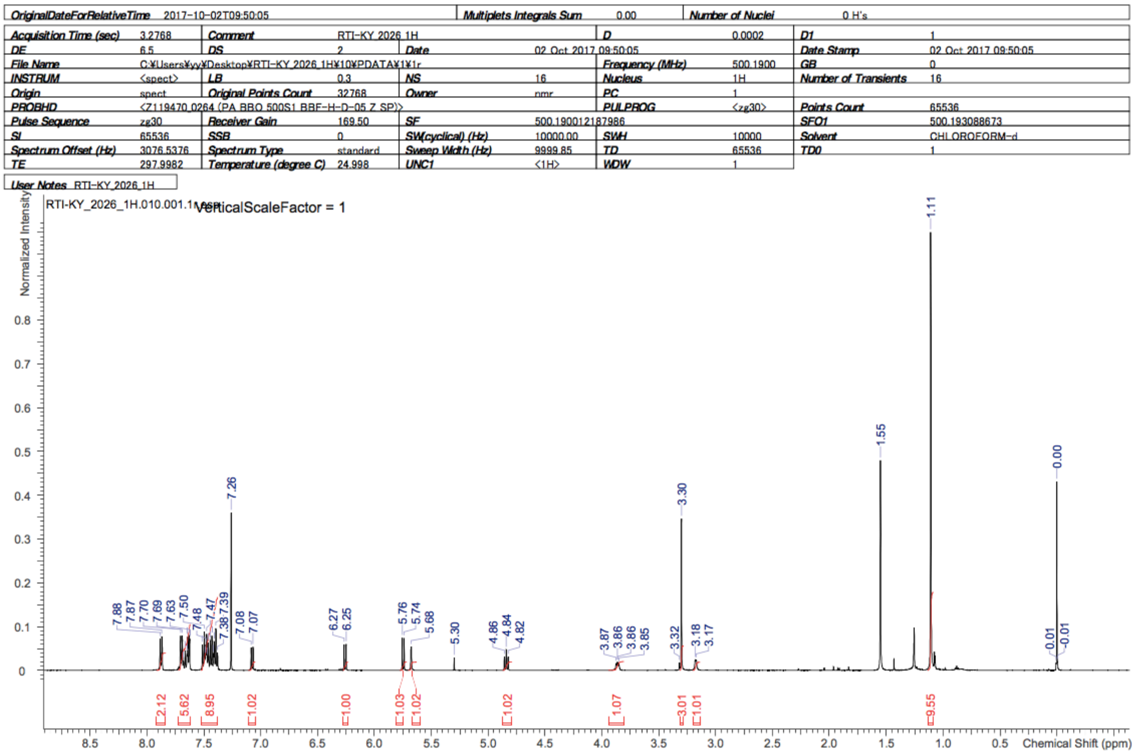

Compound **5b**, ^{13}C-NMR (126 MHz, CDCl3)

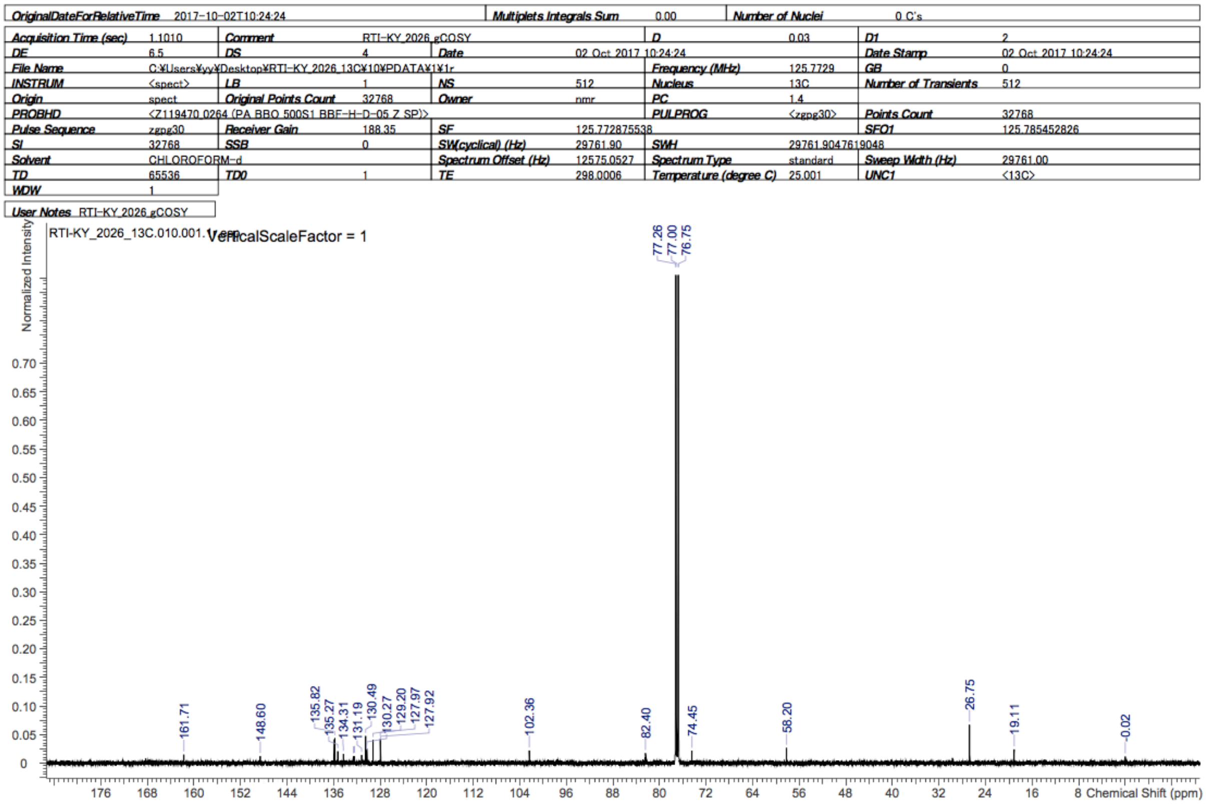

Compound **6b-E**, ^{1}H-NMR (500 MHz, CDCl*3*)

OriginalDateForRelativeTime	2017-10-17T08:34:31	Multiplets Integrals Sum	0.00	Number of Nuclei	0 H's				
Acquisition Time (sec)	3.2768	Comment	RTI-KY_2055-E_1H	D	0.0002	D1	1		
DE	6.5	DS	2	Date	17 Oct 2017 08:34:31	Date Stamp	17 Oct 2017 08:34:31		
File Name	C:¥Users¥yy¥Desktop¥RTI-KY_2058-E_1H¥10¥PDATA¥1¥1r	Frequency (MHz)	500.1900	GB	0				
INSTRUM	<spect>	LB	0.3	NS	16	Nucleus	1H	Number of Transients	16
Origin	spect	Original Points Count	32768	Owner	nmr	PC	1		
PROBHD	<Z119470_0264 (PA BBO 500S1 BBF-H-D-05 Z SP)>	PULPROG	<zg30>	Points Count	65536				
Pulse Sequence	zg30	Receiver Gain	93.24	SF	500.190013386039	SFO1	500.193088673		
SI	65536	SSB	0	SW(cyclical) (Hz)	10000.00	SWH	10000	Solvent	CHLOROFORM-d		
Spectrum Offset (Hz)	3075.3328	Spectrum Type	standard	Sweep Width (Hz)	9999.85	TD	65536	TD0	1
TE	298.0017	Temperature (degree C)	25.002	UNC1	<1H>	WDW	1		
User Notes	RTI-KY_2055-E_1H								

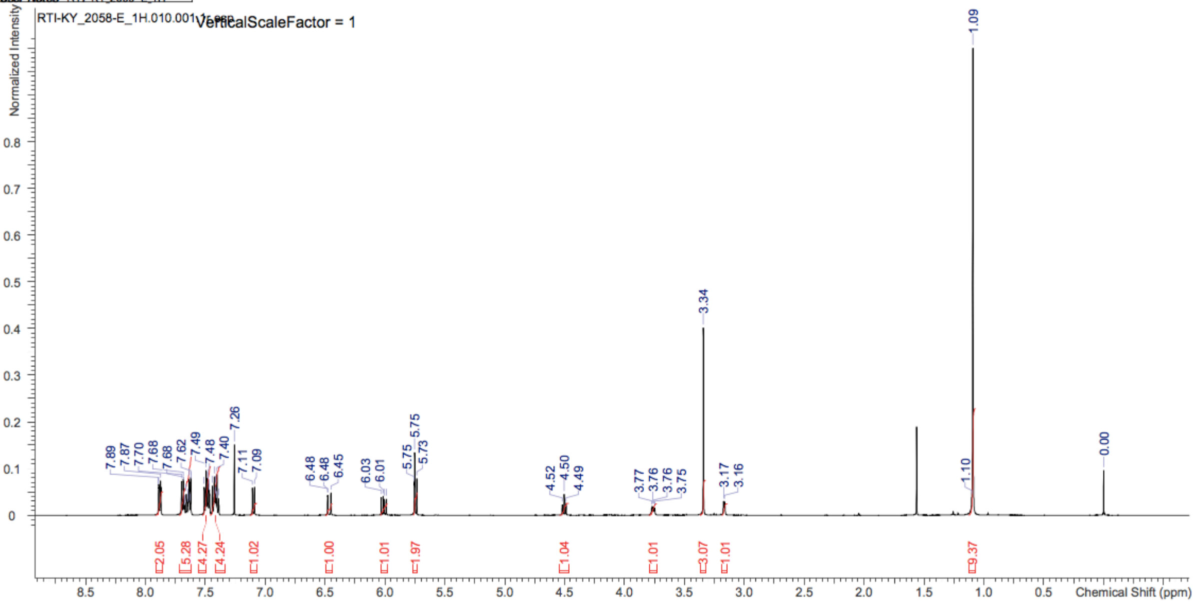

Compound **6b-E**, ^{13}C-NMR (126 MHz, CDCl*3*)

OriginalDateForRelativeTime	2017-10-17T09:02:49	Multiplets Integrals Sum	0.00	Number of Nuclei	0 C's				
Acquisition Time (sec)	1.1010	Comment	RTI-KY_2055-E_13C	D	0.03	D1	2		
DE	6.5	DS	4	Date	17 Oct 2017 09:02:49	Date Stamp	17 Oct 2017 09:02:49		
File Name	C:¥Users¥yy¥Desktop¥RTI-KY_2055-E_13C¥10¥PDATA¥1¥1r	Frequency (MHz)	125.7729	GB	0				
INSTRUM	<spect>	LB	1	NS	512	Nucleus	13C	Number of Transients	512
Origin	spect	Original Points Count	32768	Owner	nmr	PC	1.4		
PROBHD	<Z119470_0264 (PA BBO 500S1 BBF-H-D-05 Z SP)>	PULPROG	<zgpg30>	Points Count	32768				
Pulse Sequence	zgpg30	Receiver Gain	188.35	SF	125.772875538	SFO1	125.785452826		
SI	32768	SSB	0	SW(cyclical) (Hz)	29761.90	SWH	29761.9047619048		
Solvent	CHLOROFORM-d	Spectrum Offset (Hz)	12573.2402	Spectrum Type	standard	Sweep Width (Hz)	29761.00		
TD	65536	TD0	1	TE	298.001	Temperature (degree C)	25.001	UNC1	<13C>
WDW	1								
User Notes	RTI-KY_2055-E_13C								

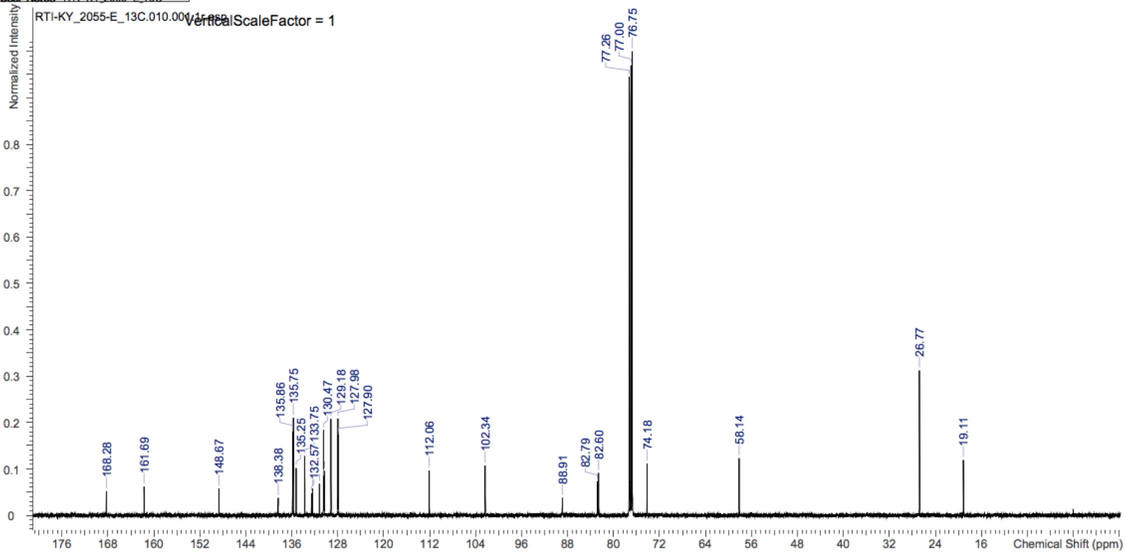

Compound **6b-Z**, ^{1}H-NMR (500 MHz, $CDCl_3$)

OriginalDateForRelativeTime	2017-10-17T09:14:35			Multiplets Integrals Sum	0.00	Number of Nuclei	0 H's
Acquisition Time (sec)	3.2768	Comment	RTI-KY_2055-Z_1H	D	0.0002	D1	1
DE	6.5	DS	2	Date	17 Oct 2017 09:14:35	Date Stamp	17 Oct 2017 09:14:35
File Name	C:¥Users¥yy¥Desktop¥RTI-KY_2058-Z_1H¥10¥PDATA¥1¥1r			Frequency (MHz)	500.1900	GB	0
INSTRUM	<spect>	LB	0.3	NS	16	Nucleus	1H
Number of Transients	16	Origin	spect	Original Points Count	32768	Owner	nmr
PC	1	PROBHD	<Z119470_0264 (PA BBO 500S1 BBF-H-D-05 Z SP)>	PULPROG	<zg30>	Points Count	65536
Pulse Sequence	zg30	Receiver Gain	76.32	SF	500.190014356999	SFO1	500.193088673
SI	65536	SSB	0	SW(cyclical) (Hz)	10000.00	SWH	10000
Solvent	CHLOROFORM-d	Spectrum Offset (Hz)	3074.4172	Spectrum Type	standard	Sweep Width (Hz)	9999.85
TD	65536	TD0	1	TE	298.0001	Temperature (degree C)	25.000
UNC1	<1H>	WDW	1				

User Notes RTI-KY_2055-Z_1H

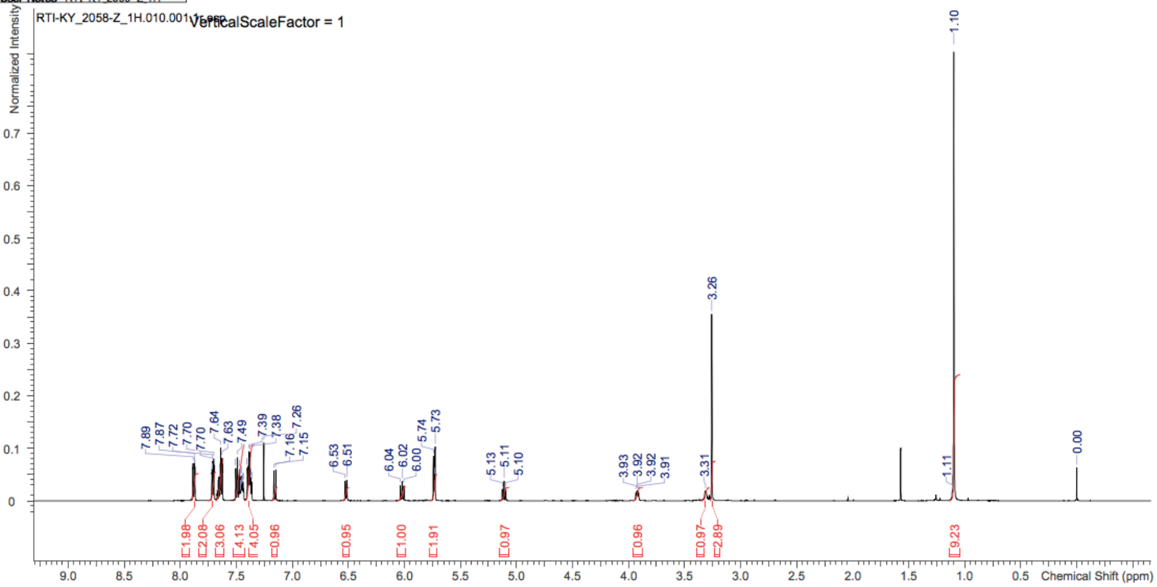

Compound **6b-Z**, ^{13}C-NMR (126 MHz, $CDCl_3$)

OriginalDateForRelativeTime	2017-10-17T09:42:52			Multiplets Integrals Sum	0.00	Number of Nuclei	0 C's
Acquisition Time (sec)	1.1010	Comment	RTI-KY_2055-Z_13C	D	0.03	D1	2
DE	6.5	DS	4	Date	17 Oct 2017 09:42:52	Date Stamp	17 Oct 2017 09:42:52
File Name	C:¥Users¥yy¥Desktop¥RTI-KY_2055-Z_13C¥10¥PDATA¥1¥1r			Frequency (MHz)	125.7729	GB	0
INSTRUM	<spect>	LB	1	NS	512	Nucleus	13C
Number of Transients	512	Origin	spect	Original Points Count	32768	Owner	nmr
PC	1.4	PROBHD	<Z119470_0264 (PA BBO 500S1 BBF-H-D-05 Z SP)>	PULPROG	<zgpg30>	Points Count	32768
Pulse Sequence	zgpg30	Receiver Gain	188.35	SF	125.772875538	SFO1	125.785452826
SI	32768	SSB	0	SW(cyclical) (Hz)	29761.90	SWH	29761.9047619048
Solvent	CHLOROFORM-d	Spectrum Offset (Hz)	12571.4180	Spectrum Type	standard	Sweep Width (Hz)	29761.00
TD	65536	TD0	1	TE	297.9991	Temperature (degree C)	24.999
UNC1	<13C>	WDW	1				

User Notes RTI-KY_2055-Z_13C

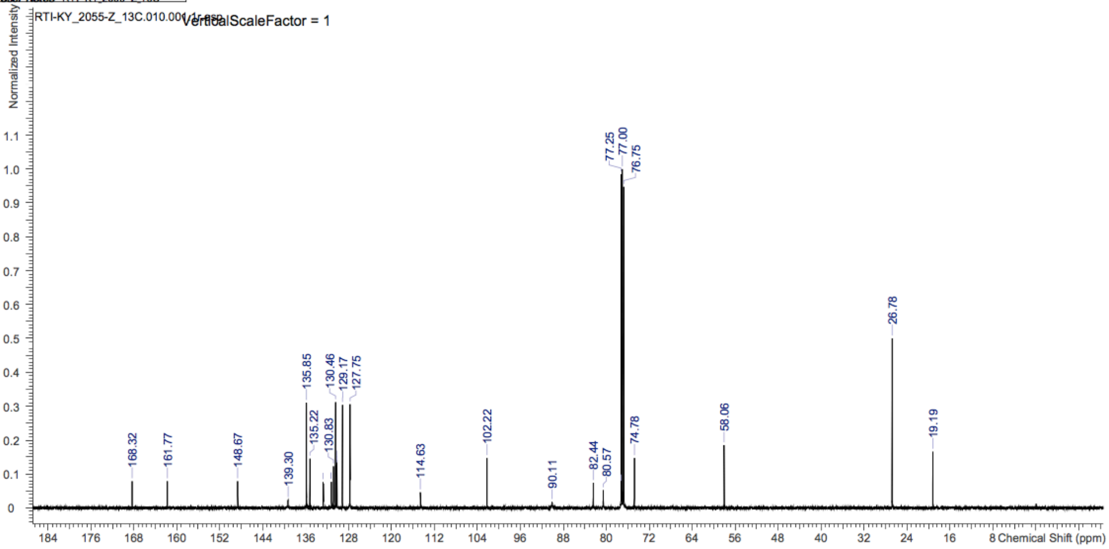

Compound **7b** (least polar isomer), ^{1}H-NMR (500 MHz, $CDCl_3$)

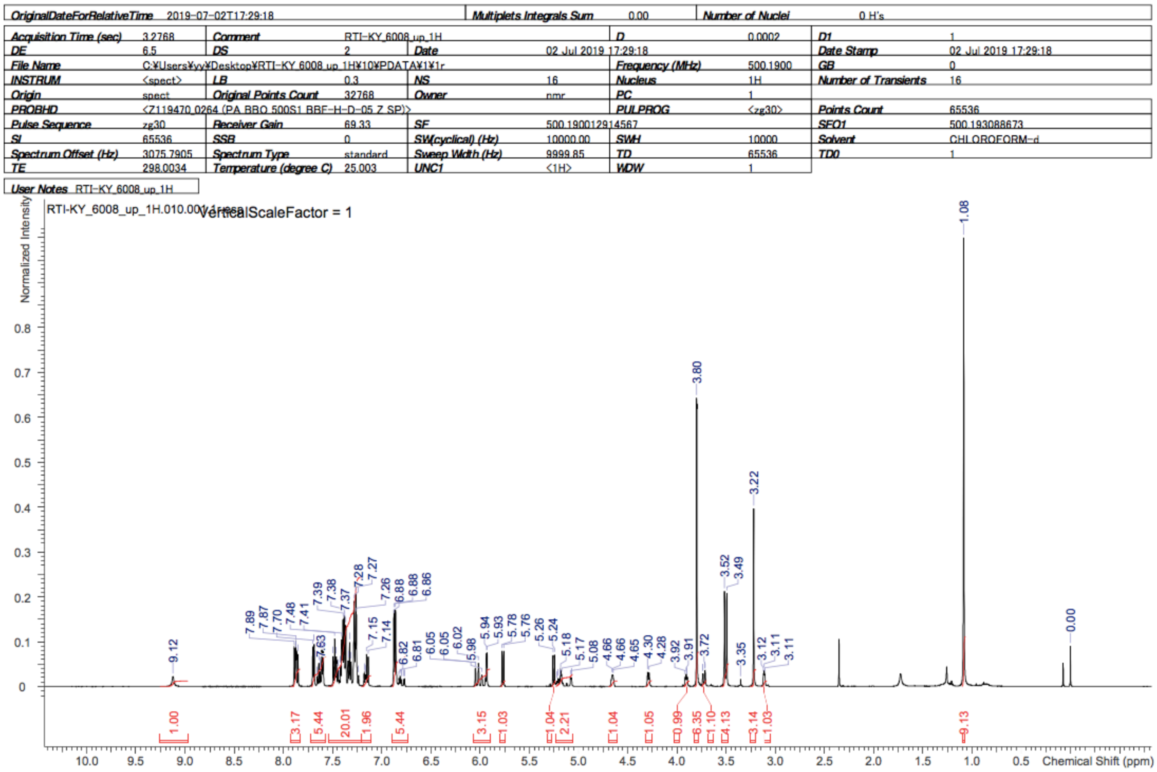

Compound **7b** (least polar isomer), ^{13}C-NMR (126 MHz, $CDCl_3$)

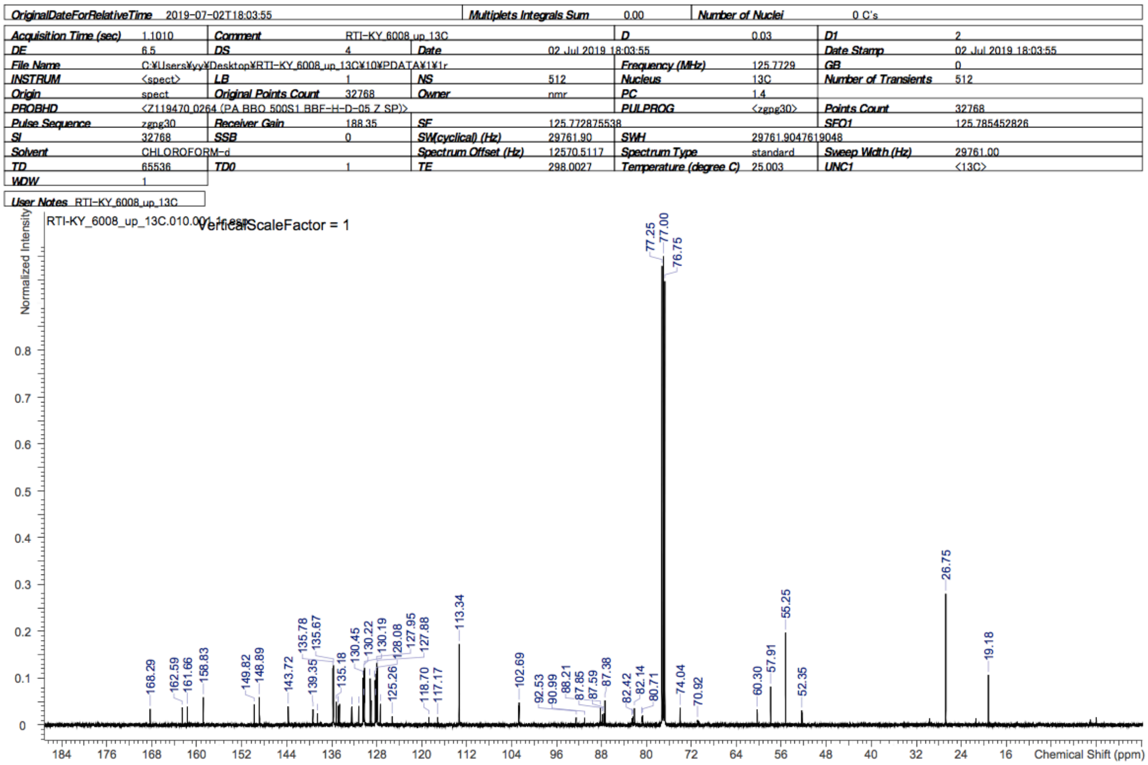

Compound **7b** (least polar isomer), ^{31}P-NMR (202 MHz, $CDCl_3$)

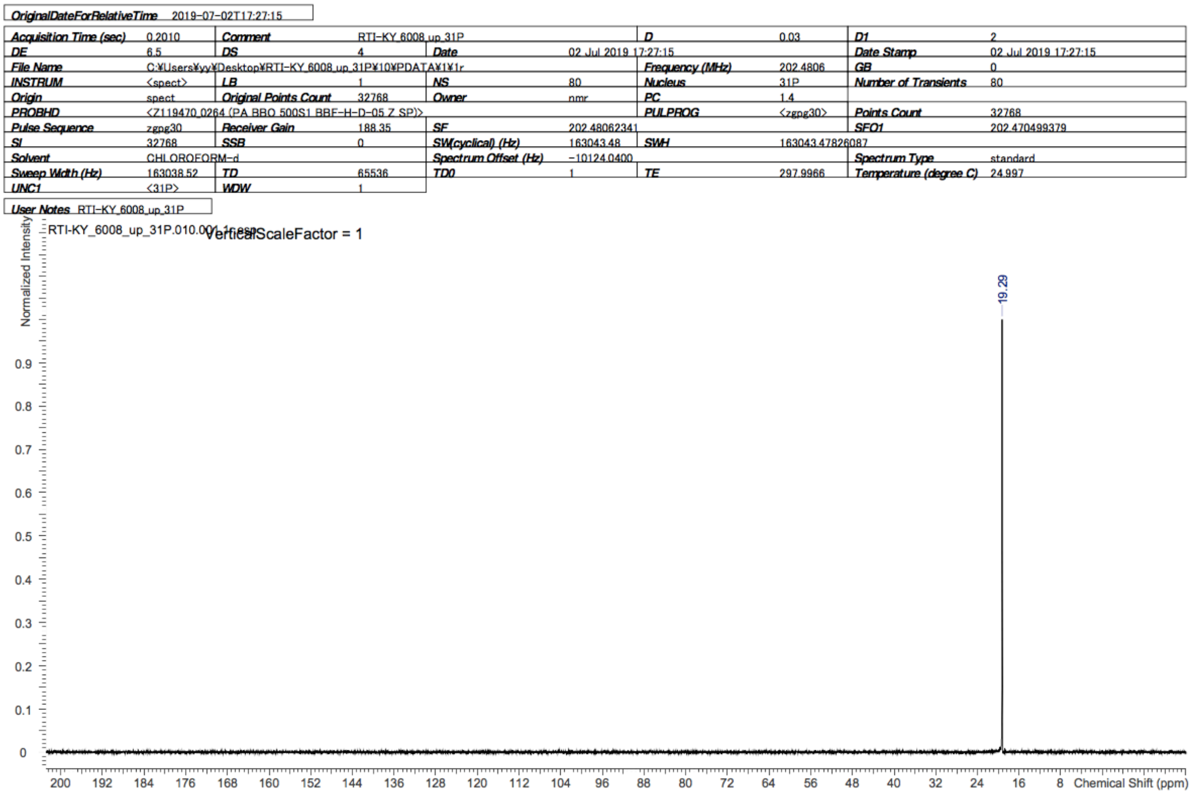

Compound **7b** (most polar isomer), ^{1}H-NMR (500 MHz, $CDCl_3$)

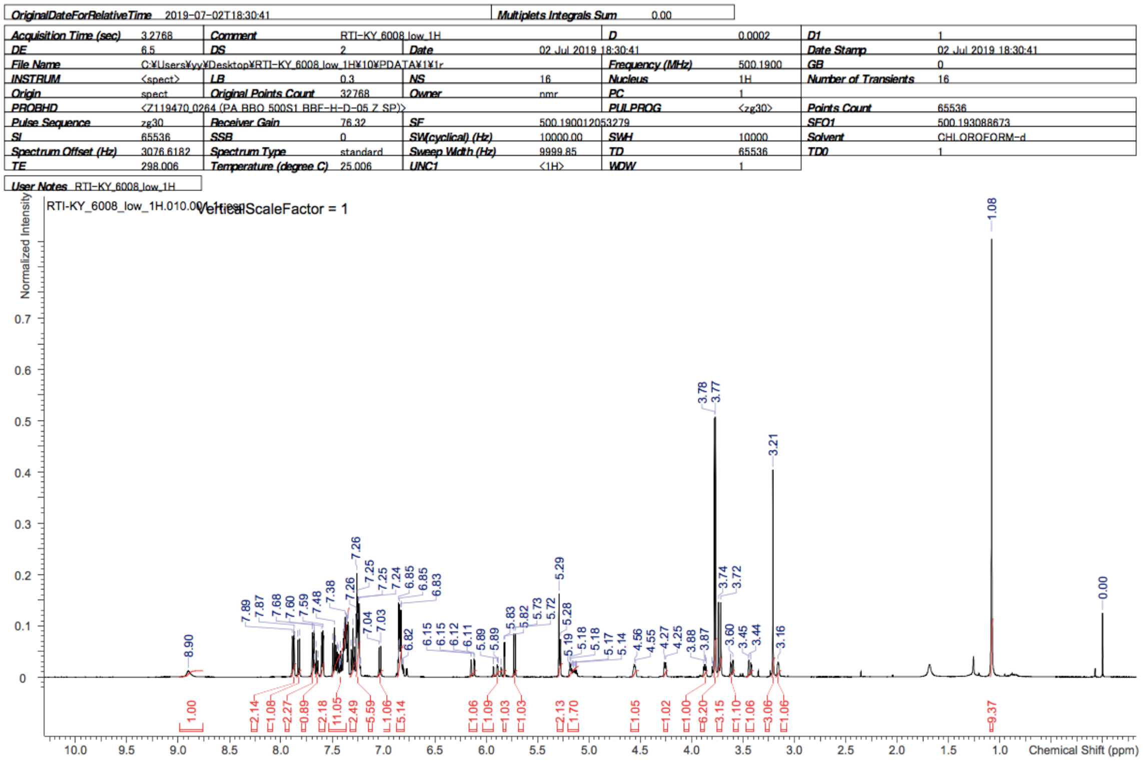

Compound **7b** (most polar isomer), ^{13}C-NMR (126 MHz, $CDCl_3$)

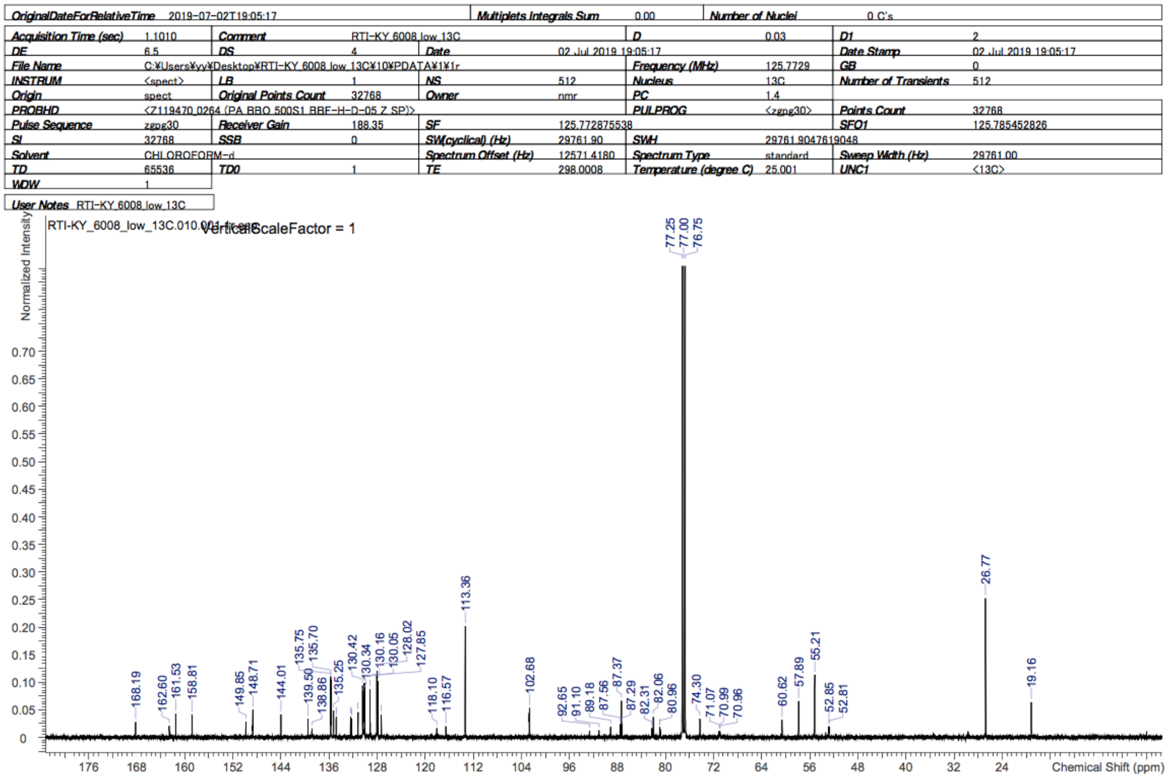

Compound **7b** (most polar isomer), ^{31}P-NMR (202 MHz, $CDCl_3$)

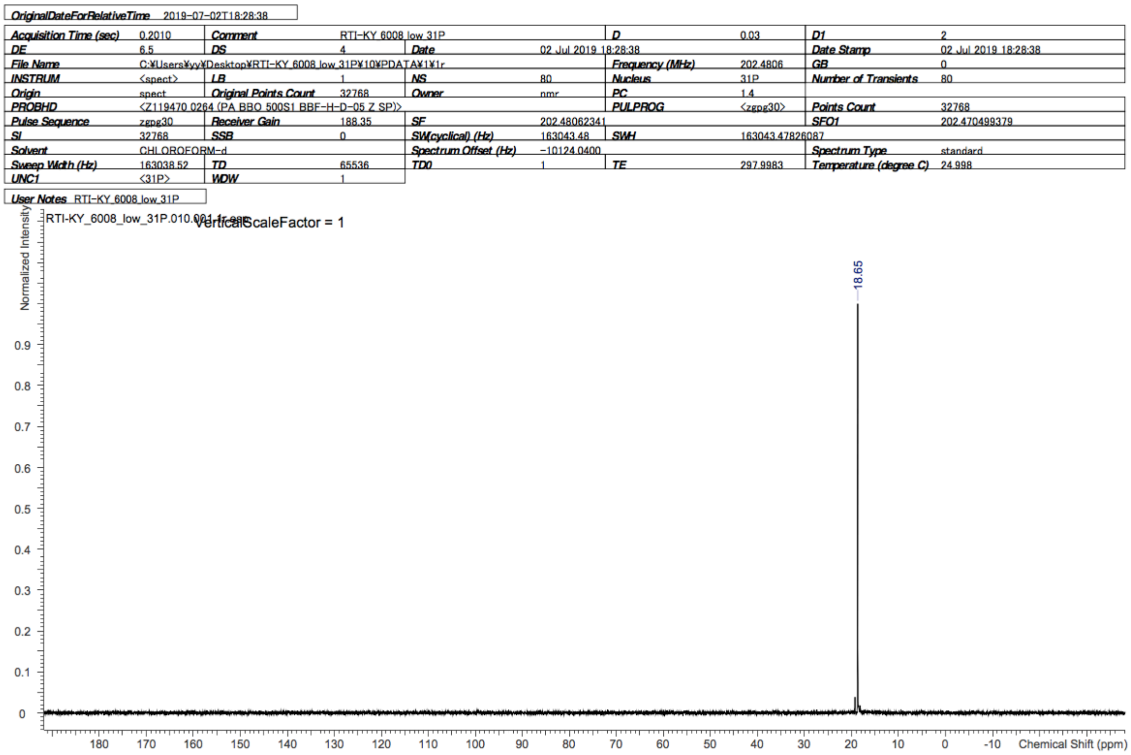

Compound **8b** (least polar isomer), ^{1}H-NMR (500 MHz, $CDCl_3$)

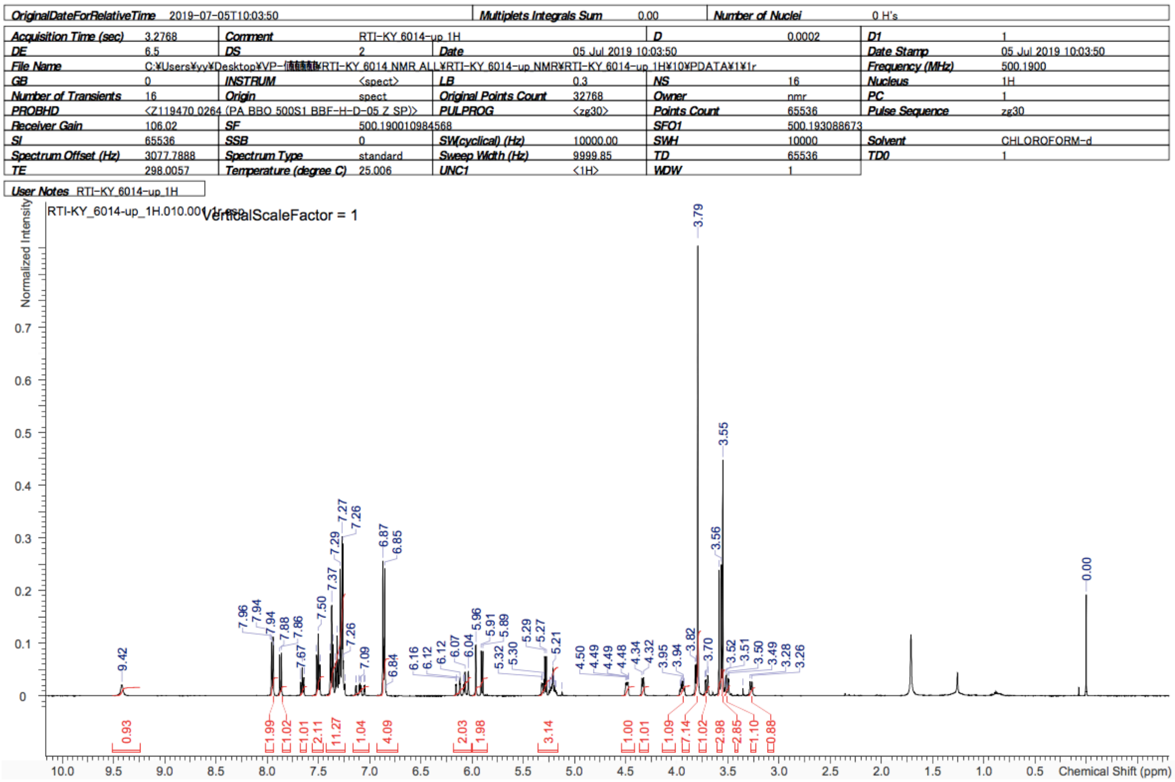

Compound **8b** (least polar isomer), ^{13}C-NMR (126 MHz, $CDCl_3$)

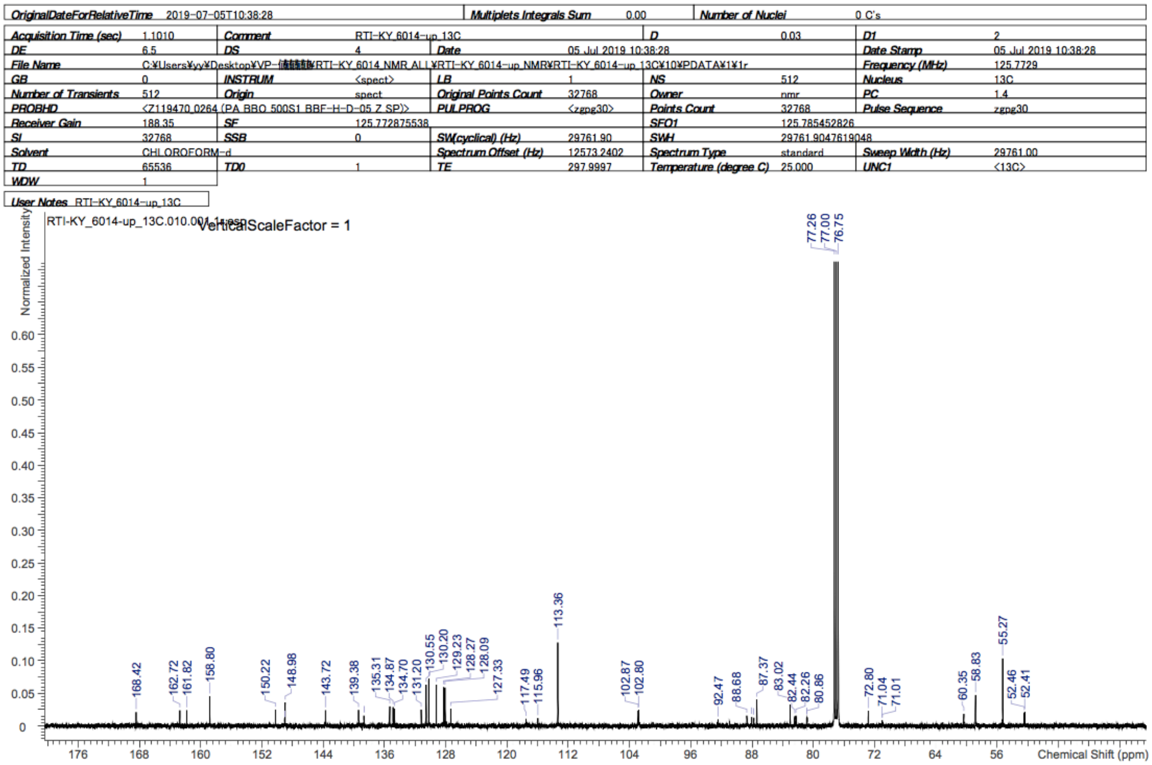

Compound **8b** (least polar isomer), ^{19}F-NMR (470 MHz, CD_3CN-d_3)

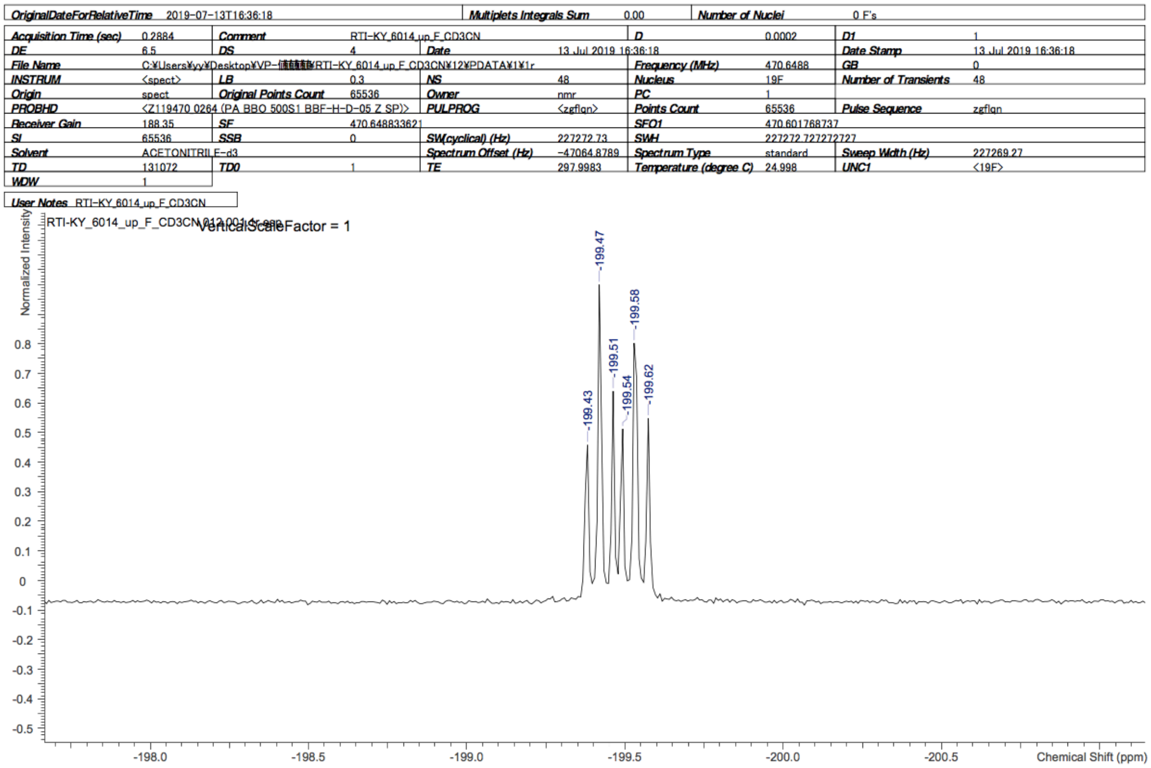

Compound **8b** (least polar isomer), ^{31}P-NMR (202 MHz, $CDCl_3$)

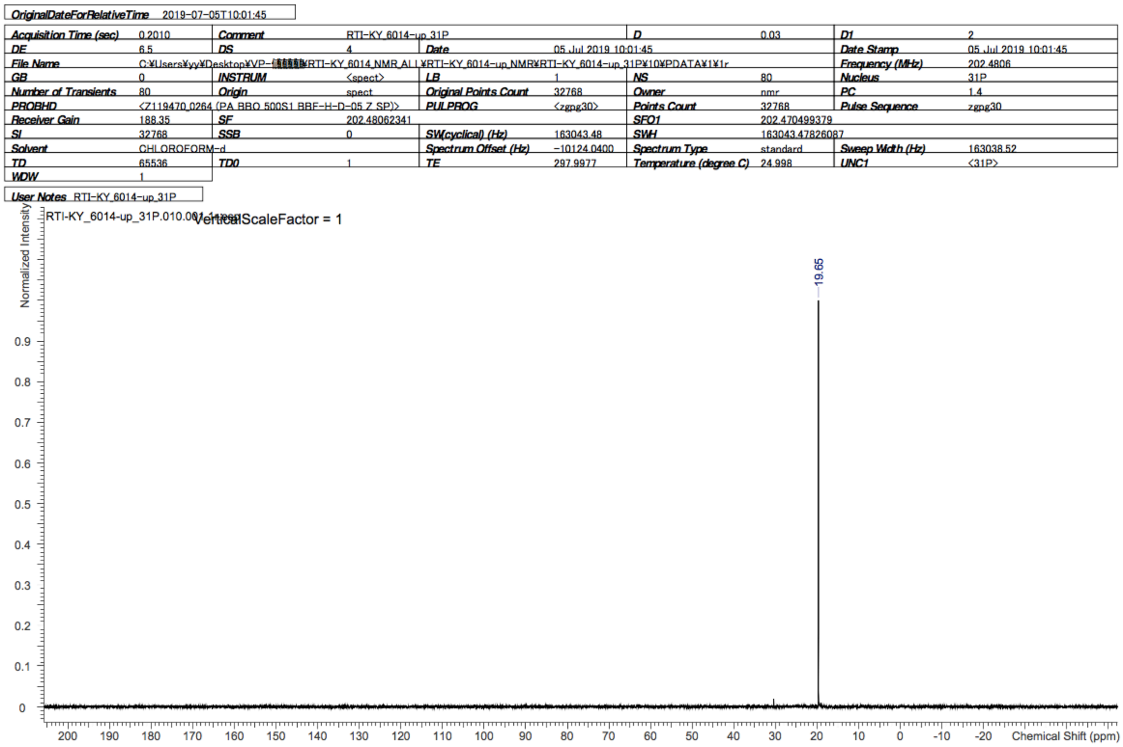

Compound **8b** (most polar isomer), ^{1}H-NMR (500 MHz, CD_3Cl)

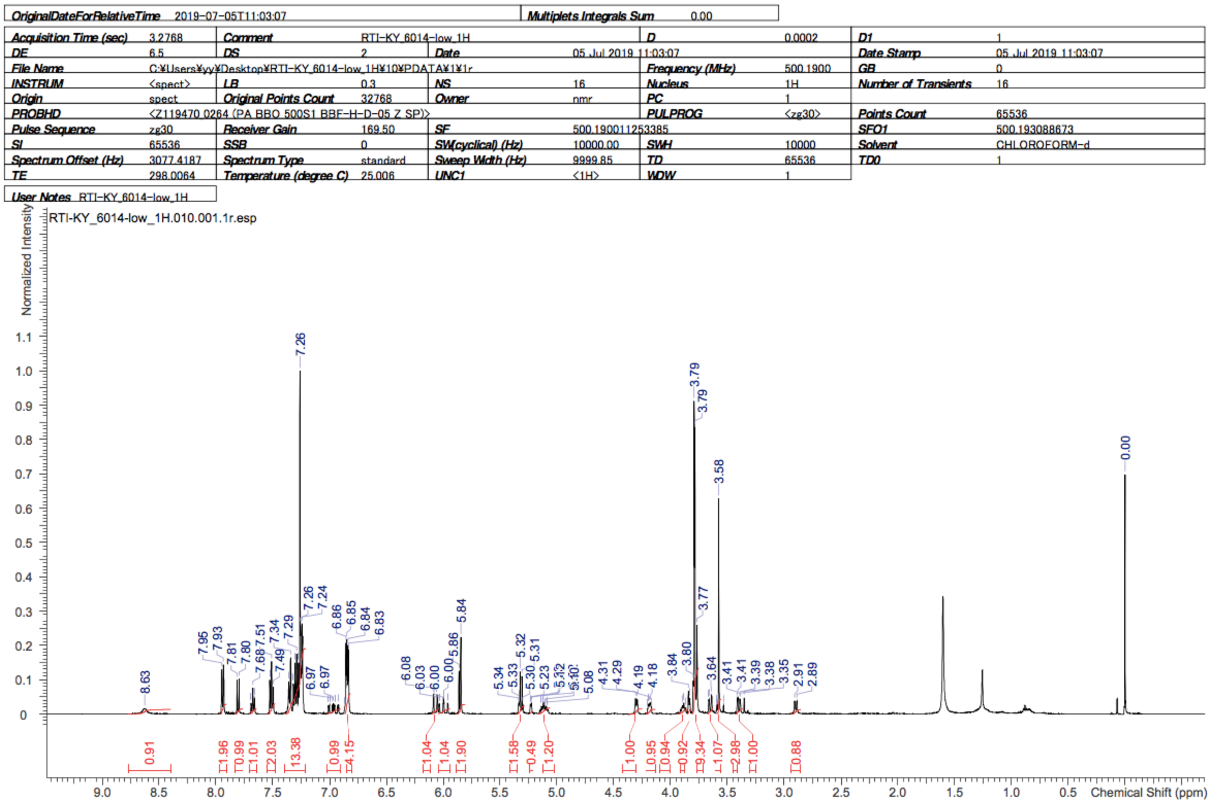

Compound **8b** (most polar isomer), ^{13}C-NMR (126 MHz, CD_3Cl)

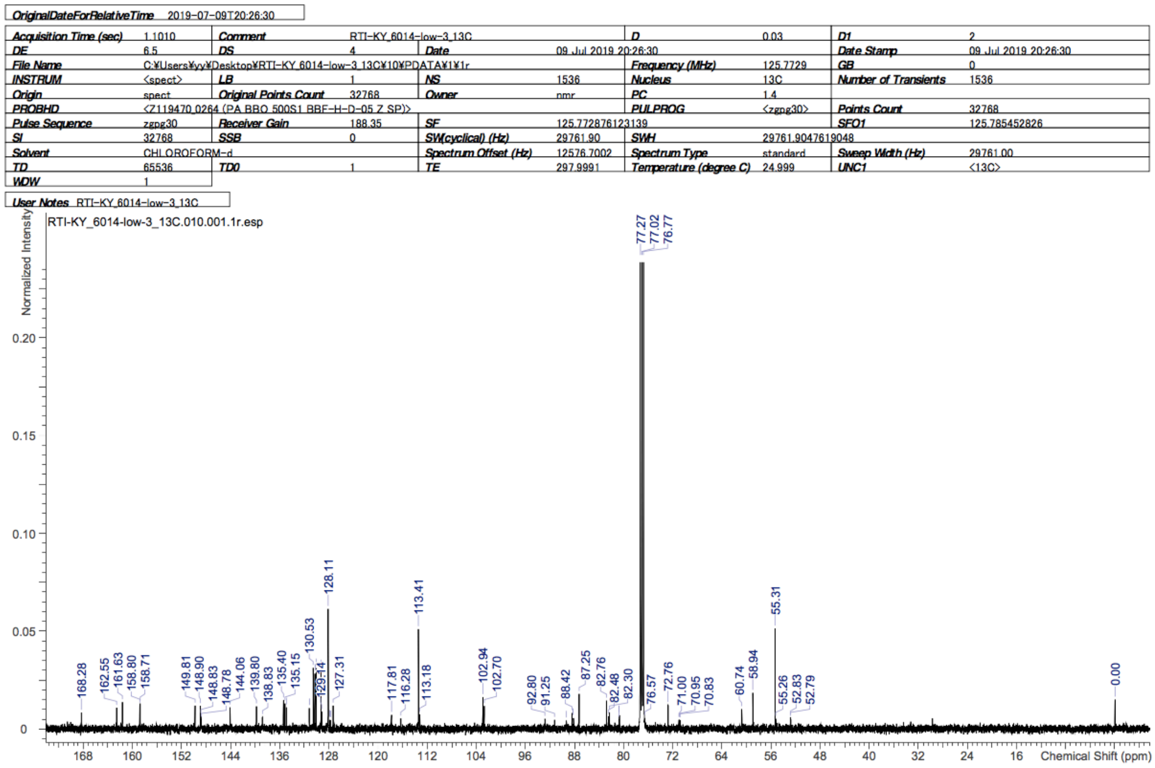

Compound **8b** (most polar isomer), ^{19}F-NMR (470 MHz, CD_3CN-*d3*)

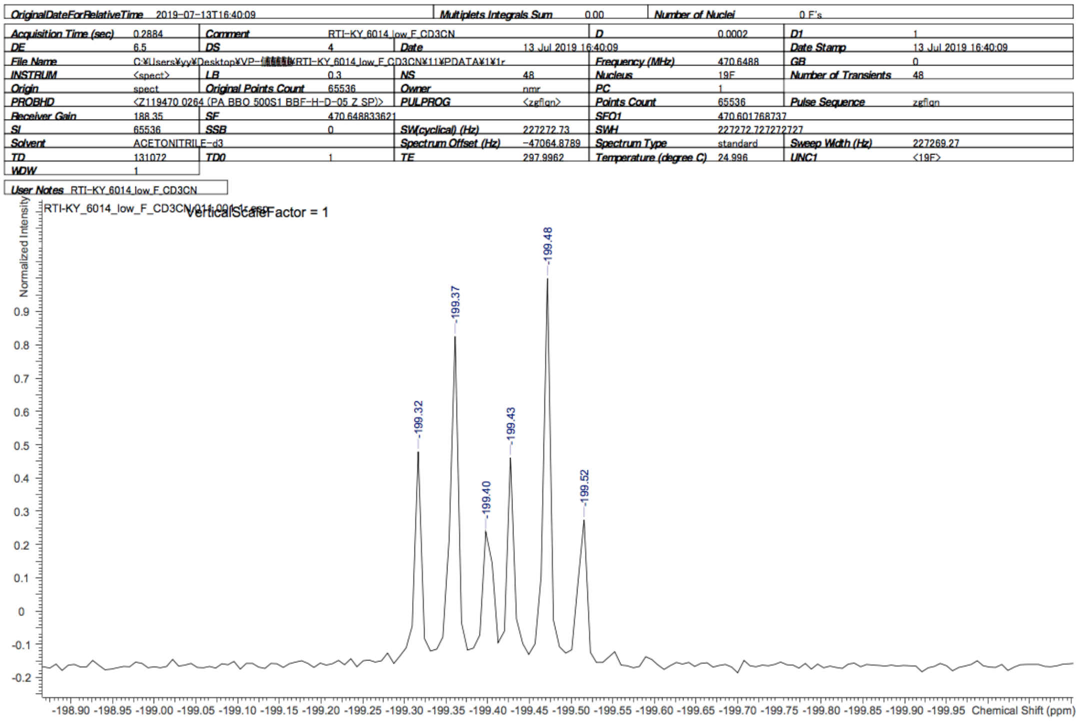

Compound **8b** (most polar isomer), ^{31}P-NMR (202 MHz, CD_3Cl)

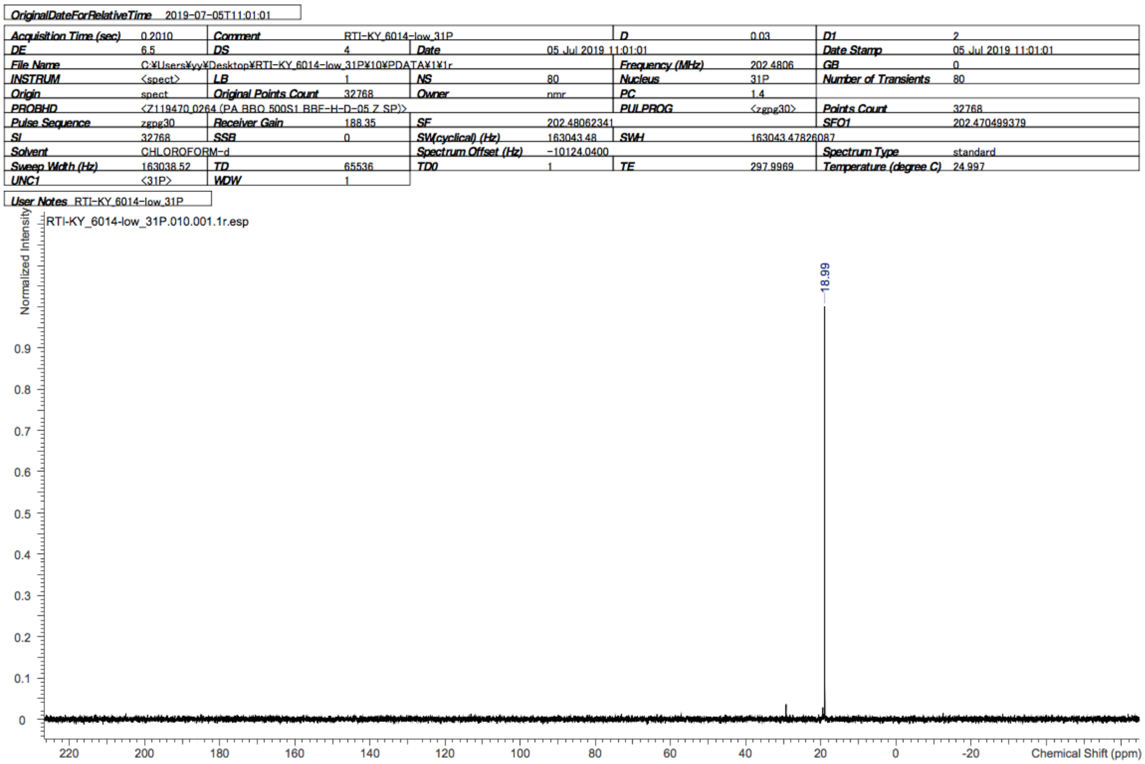

Compound **9b**, ^{31}P-NMR (202 MHz, CD_3Cl)

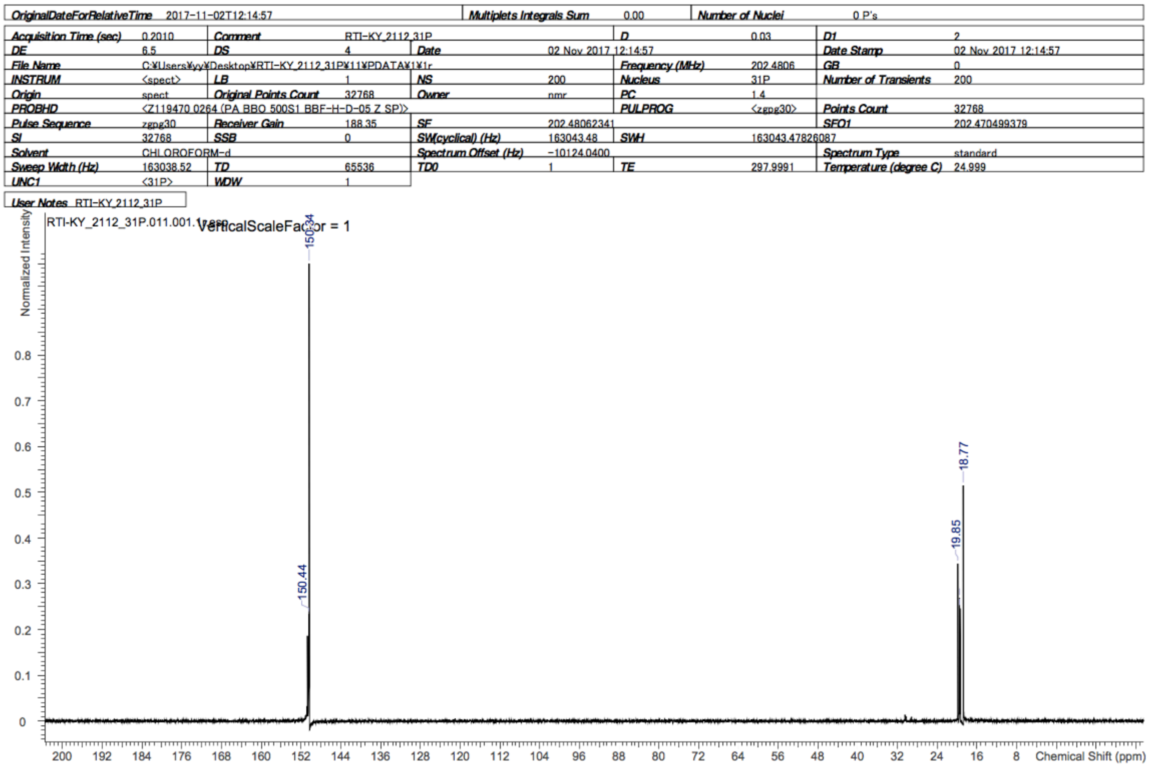

Compound **7c** (least polar isomer), ^{1}H-NMR (500 MHz, CD_3CN-d_3)

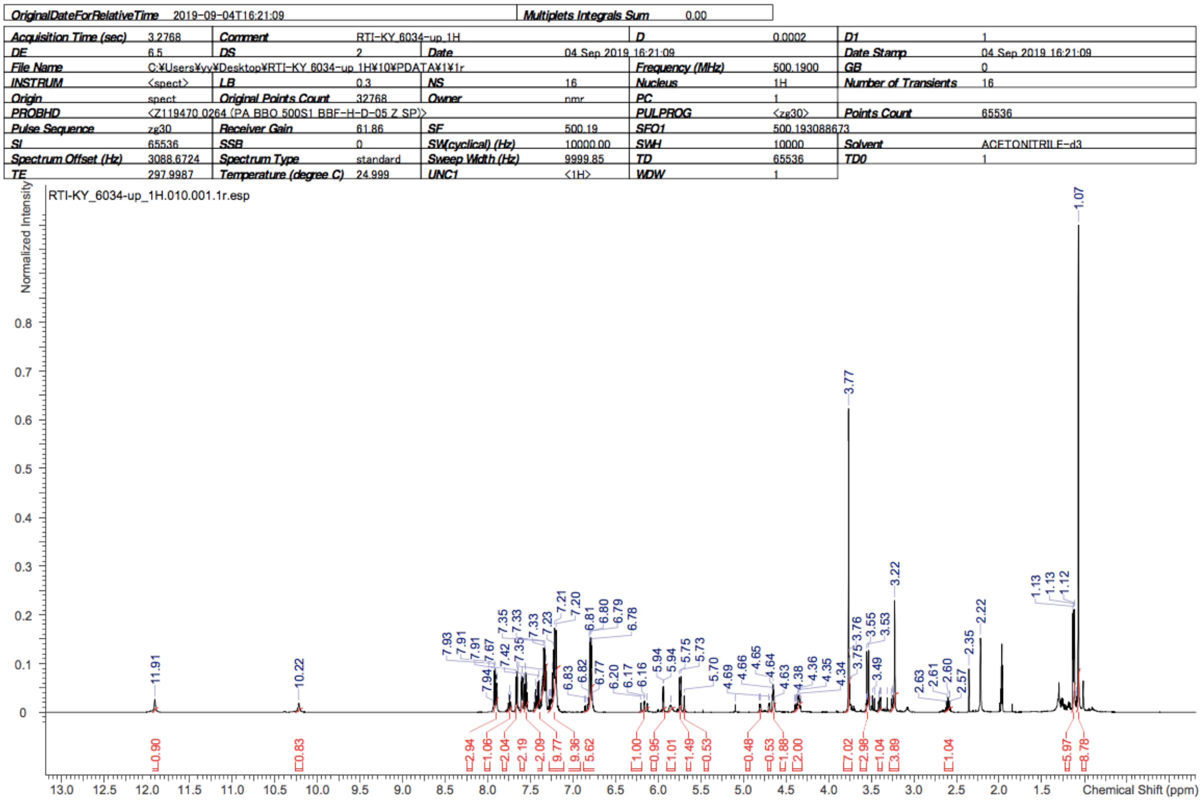

Compound **7c** (least polar isomer), ^{13}C-NMR (126 MHz, CD_3CN-d_3)

OriginalDateForRelativeTime	2019-09-04T16:59:06	*Multiplets Integrals Sum*	0.00	*Number of Nuclei*	0 C's

Acquisition Time (sec)	1.1010	*Comment*	RTI-KY_6034-up_13C			*D*	0.03	*D1*	2
DE	6.5	*DS*	4	*Date*	04 Sep 2019 16:59:06			*Date Stamp*	04 Sep 2019 16:59:06
File Name	C:¥Users¥yy¥Desktop¥RTI-KY_6034-up_13C¥10¥PDATA¥1¥1r					*Frequency (MHz)*	125.7728	*GB*	0
INSTRUM	<spect>	*LB*	1	*NS*	512	*Nucleus*	13C	*Number of Transients*	512
Origin	spect	*Original Points Count*	32768	*Owner*	nmr	*PC*	1.4		
PROBHD	<Z119470_0264 (PA BBO 500S1 BBF-H-D-05 Z SP)>					*PULPROG*	<zgpg30>	*Points Count*	32768
Pulse Sequence	zgpg30	*Receiver Gain*	188.35	*SF*	125.772806224356			*SFO1*	125.785452826
SI	32768	*SSB*	0	*SW(cyclical) (Hz)*	29761.90	*SWH*	29761.9047619048		
Solvent	ACETONITRILE-d3			*Spectrum Offset (Hz)*	12646.6016	*Spectrum Type*	standard	*Sweep Width (Hz)*	29761.00
TD	65536	*TD0*	1	*TE*	297.9996	*Temperature (degree C)*	25.000	*UNC1*	<13C>
WDW	1								

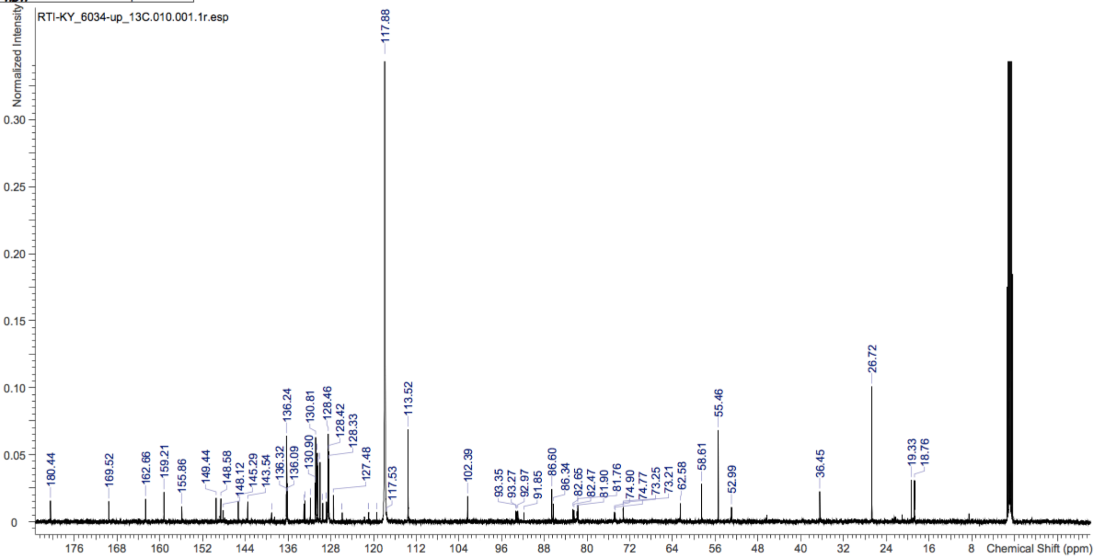

Compound **7c** (least polar isomer), ^{19}F-NMR (470 MHz, CD_3CN-d_3)

OriginalDateForRelativeTime	2019-09-04T16:30:19	*Multiplets Integrals Sum*	0.00	*Number of Nuclei*	0 F's

Acquisition Time (sec)	0.2884	*Comment*	RTI-KY_6034-up_19F			*D*	0.0002	*D1*	1
DE	6.5	*DS*	4	*Date*	04 Sep 2019 16:30:19			*Date Stamp*	04 Sep 2019 16:30:19
File Name	C:¥Users¥yy¥Desktop¥RTI-KY_6034-up_19F¥10¥PDATA¥1¥1r					*Frequency (MHz)*	470.6488	*GB*	0
INSTRUM	<spect>	*LB*	0.3	*NS*	80	*Nucleus*	19F	*Number of Transients*	80
Origin	spect	*Original Points Count*	65536	*Owner*	nmr	*PC*	1		
PROBHD	<Z119470_0264 (PA BBO 500S1 BBF-H-D-05 Z SP)>					*PULPROG*	<zgflqn>	*Points Count*	65536
Pulse Sequence	zgflqn	*Receiver Gain*	188.35	*SF*	470.648833621			*SFO1*	470.601768737
SI	65536	*SSB*	0	*SW(cyclical) (Hz)*	227272.73	*SWH*	227272.727272727		
Solvent	ACETONITRILE-d3			*Spectrum Offset (Hz)*	-47064.8789			*Spectrum Type*	standard
Sweep Width (Hz)	227269.27	*TD*	131072	*TD0*	1	*TE*	298.0009	*Temperature (degree C)*	25.001
UNC1	<19F>	*WDW*	1						

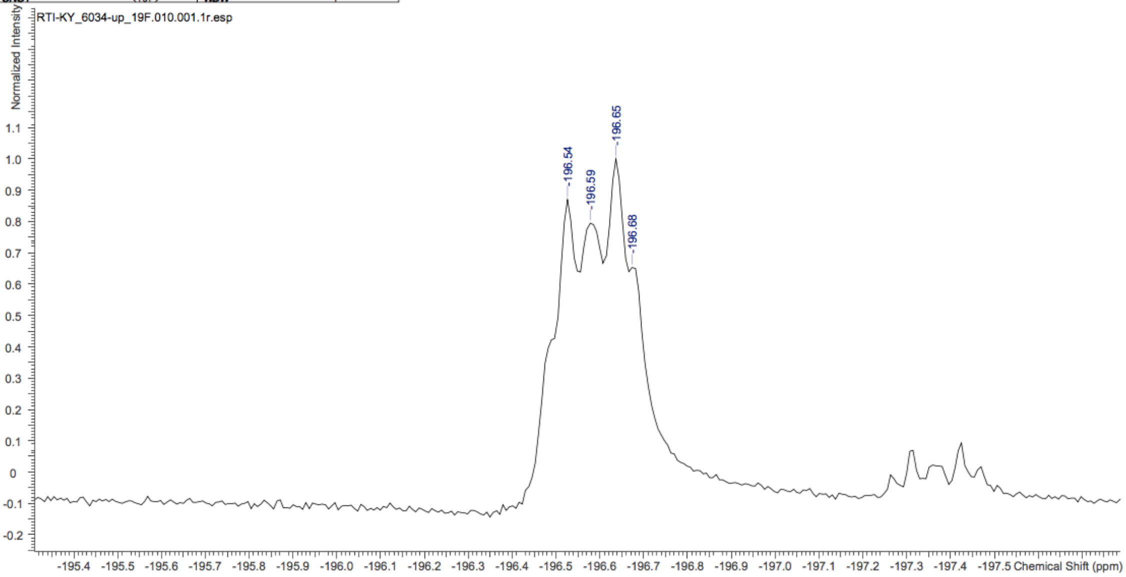

Compound **7c** (least polar isomer), ^{31}P-NMR (202 MHz, CD_3CN-d_3)

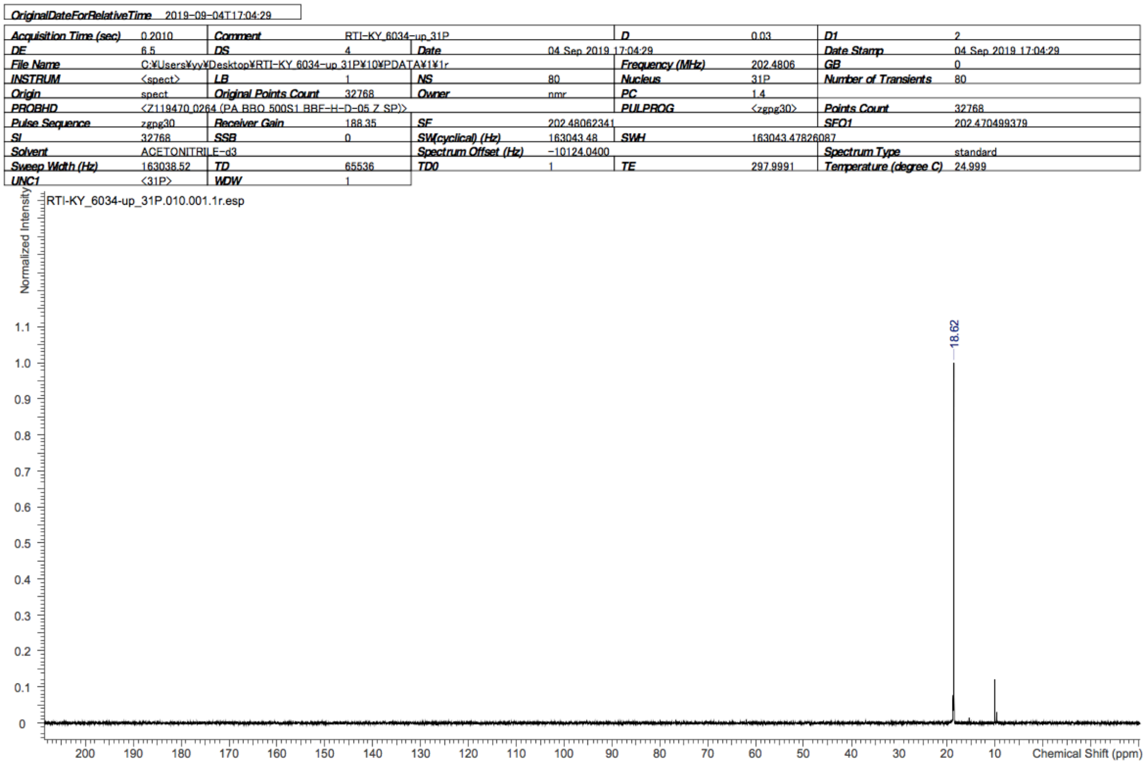

Compound **7c** (most polar isomer), ^{1}H-NMR (126 MHz, CD_3CN-d_3)

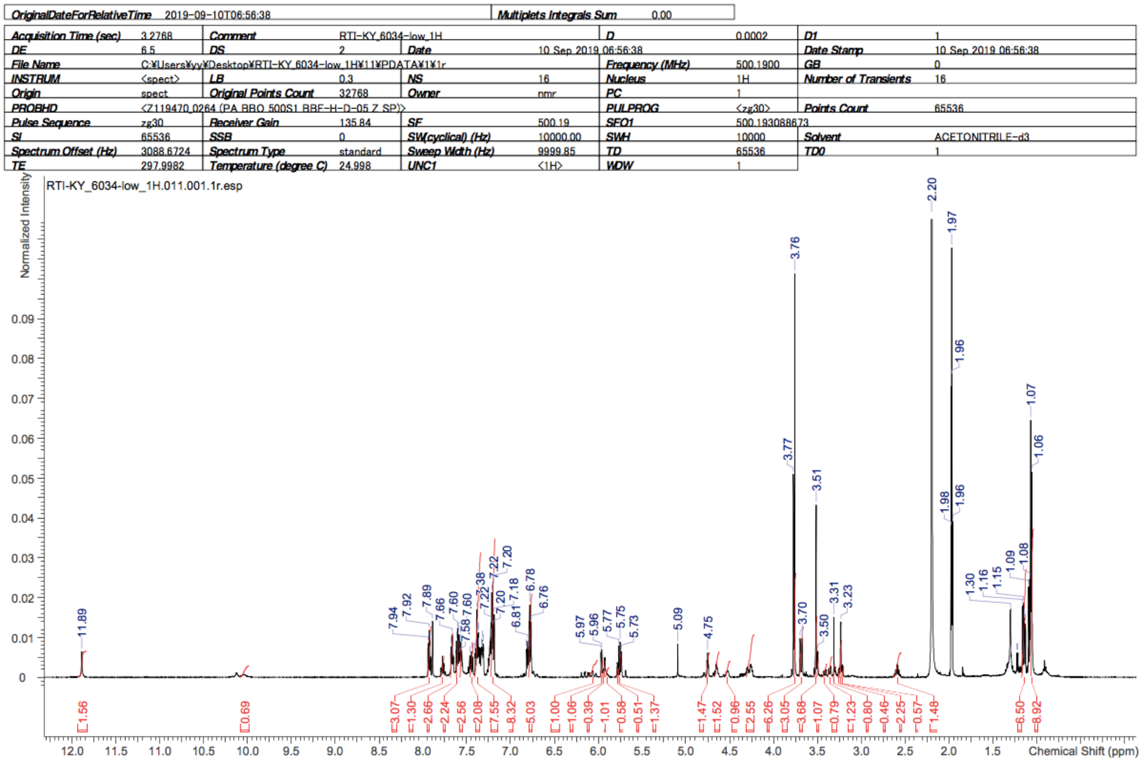

Compound **7c** (most polar isomer), ^{13}C-NMR (126 MHz, CD_3CN-d_3)

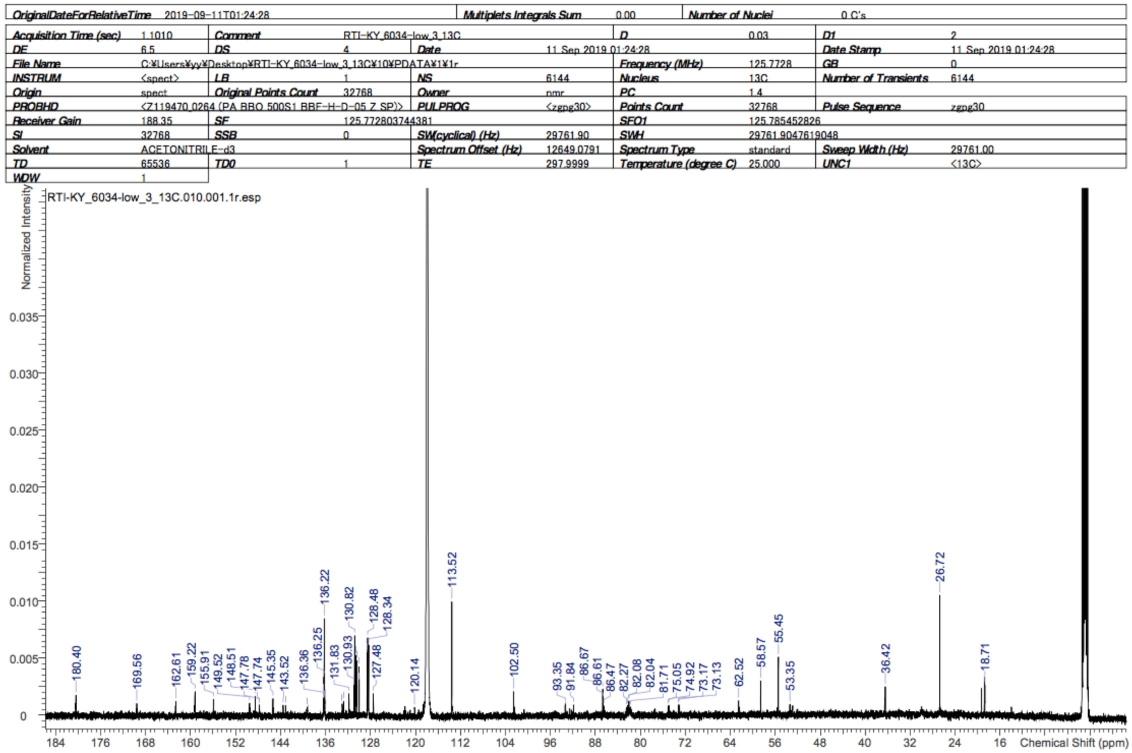

Compound **7c** (most polar isomer), ^{19}F-NMR (202 MHz, CD_3CN-d_3)

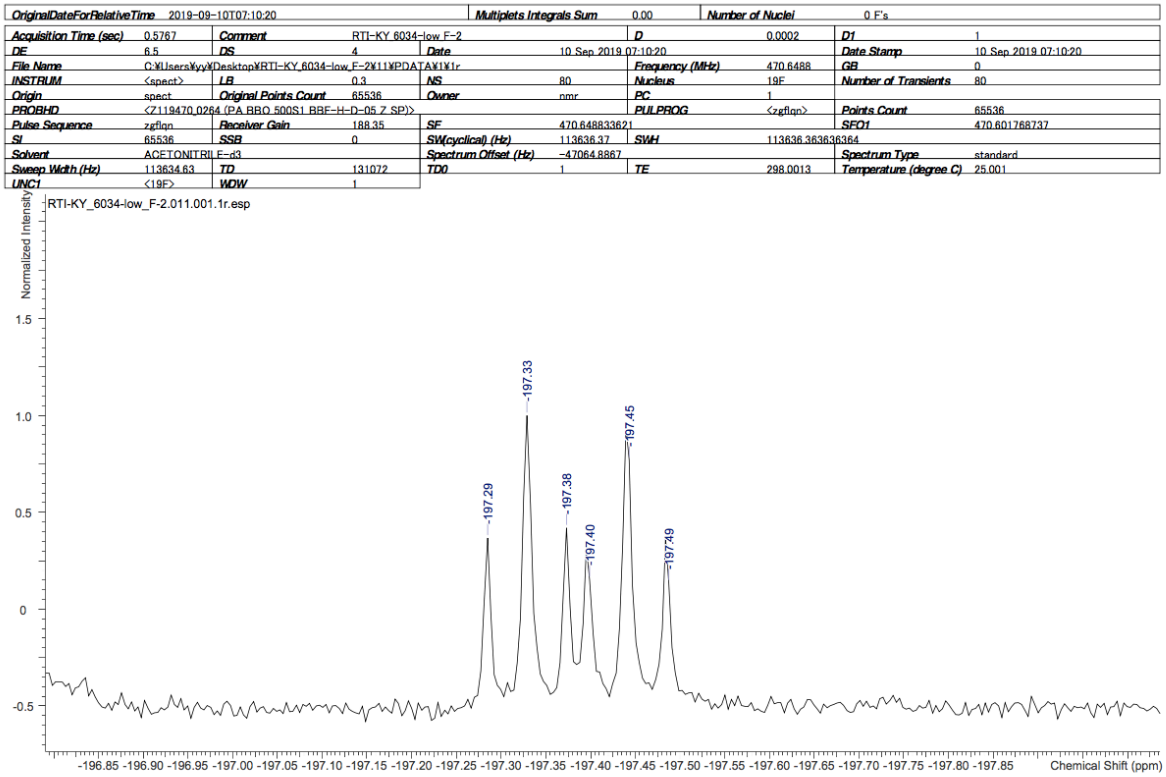

Compound **7c** (most polar isomer), ^{31}P-NMR (202 MHz, CD_3CN-d_3)

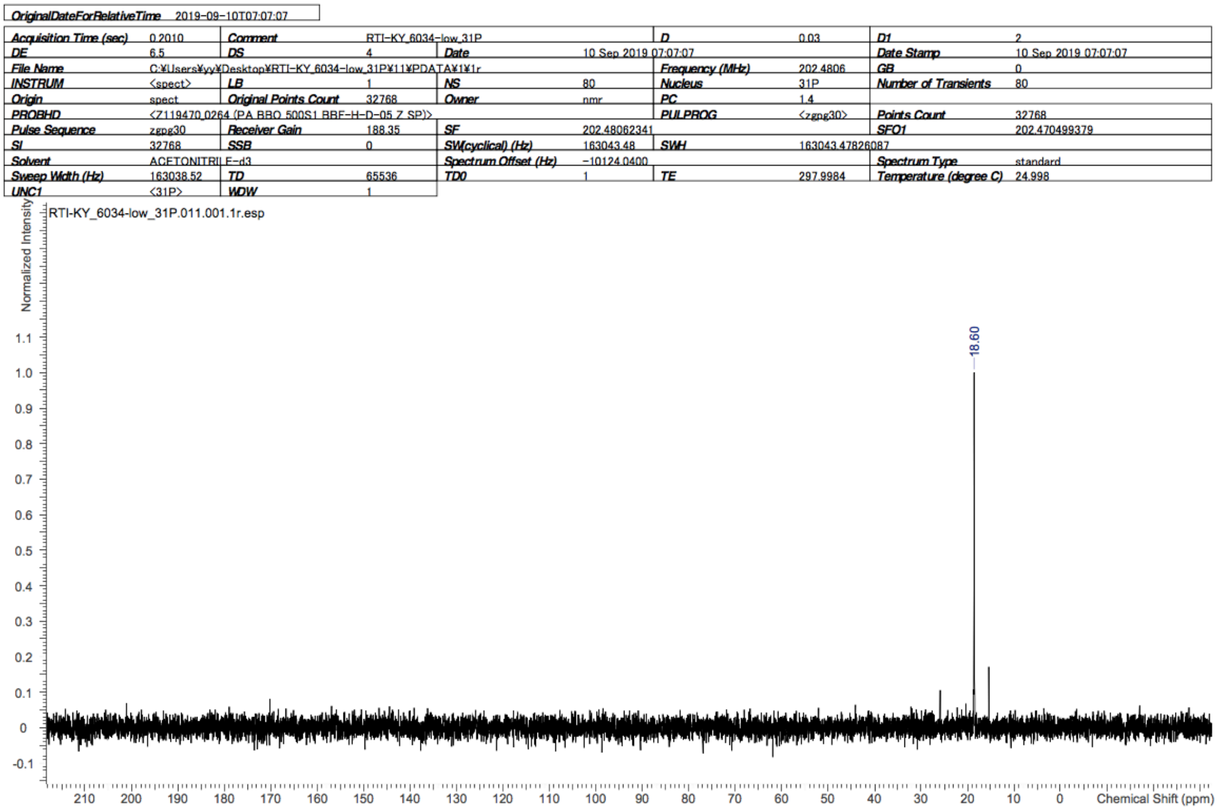

Compound **8c** (least polar isomer), ^{1}H-NMR (500 MHz, CD_3CN-d_3)

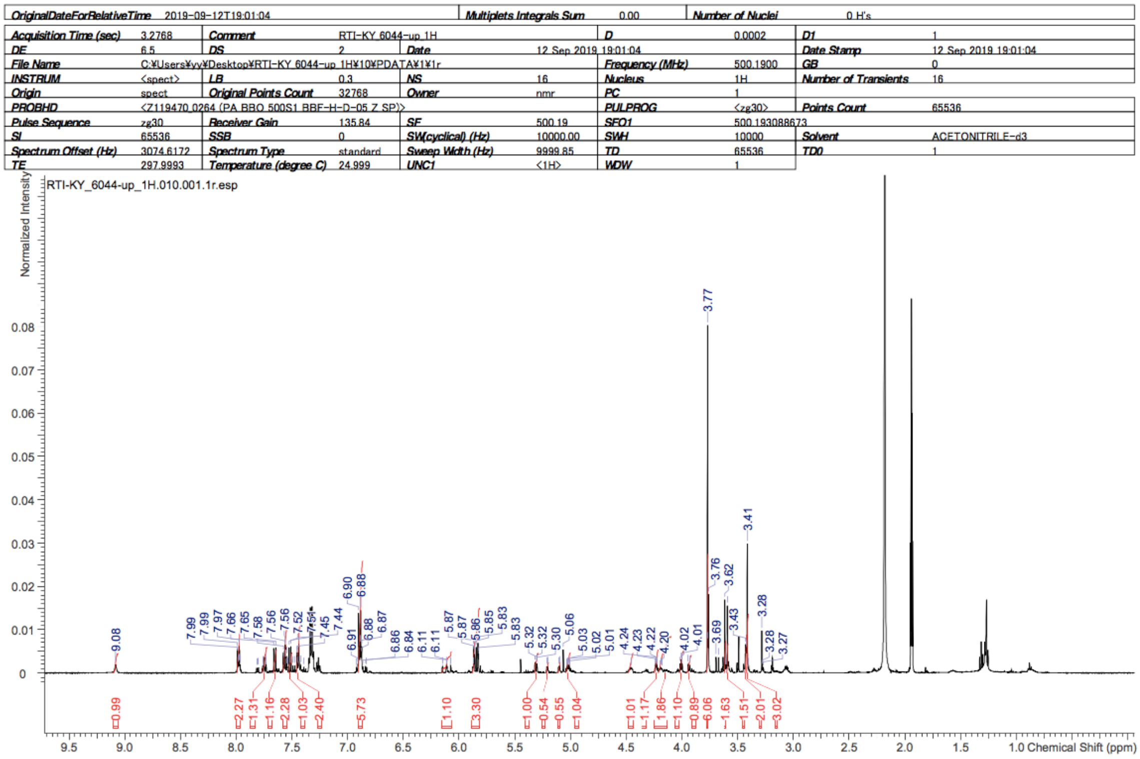

Compound **8c** (least polar isomer), ^{13}C-NMR (126 MHz, CD_3CN-d_3)

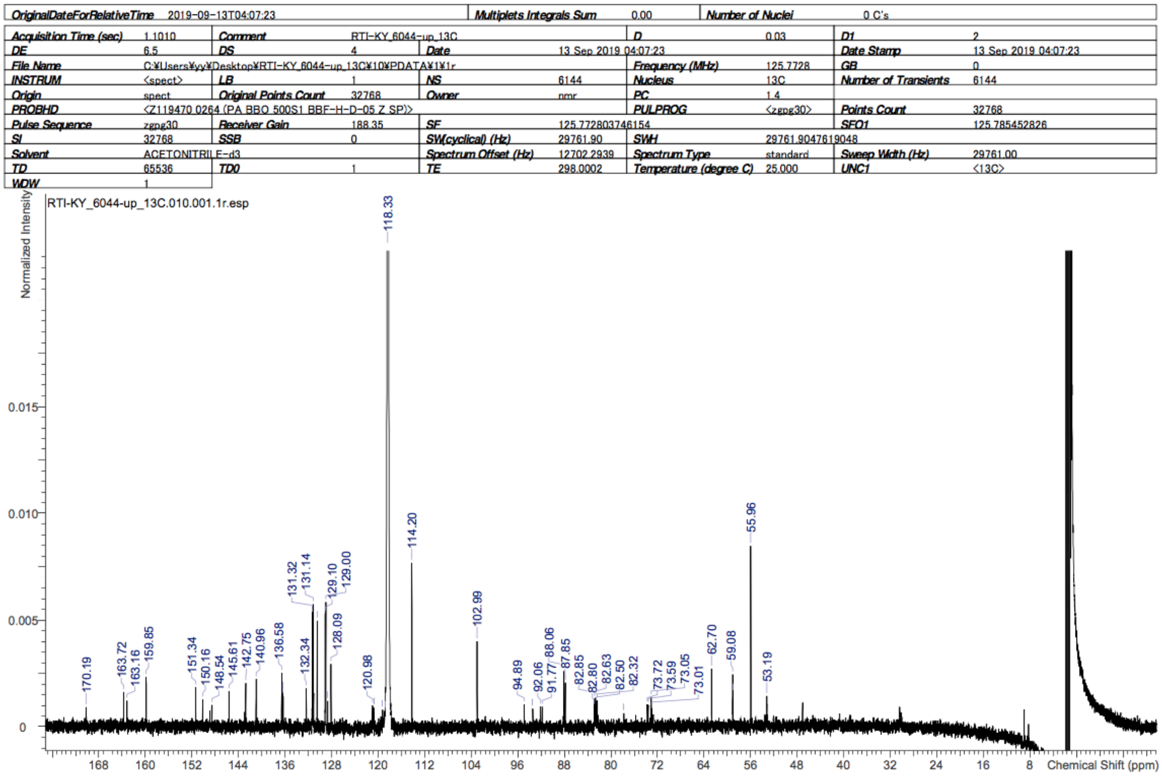

Compound **8c** (least polar isomer), ^{19}F-NMR (470 MHz, CD_3CN-d_3)

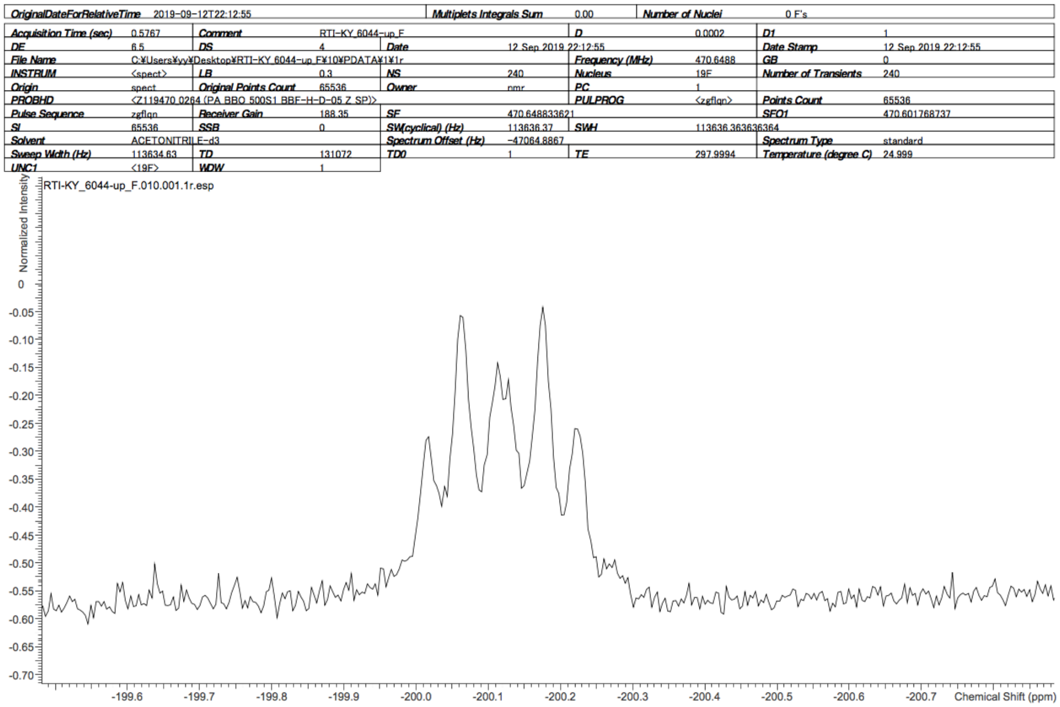

Compound **8c** (least polar isomer), ^{31}P-NMR (202 MHz, CD_3CN-d_3)

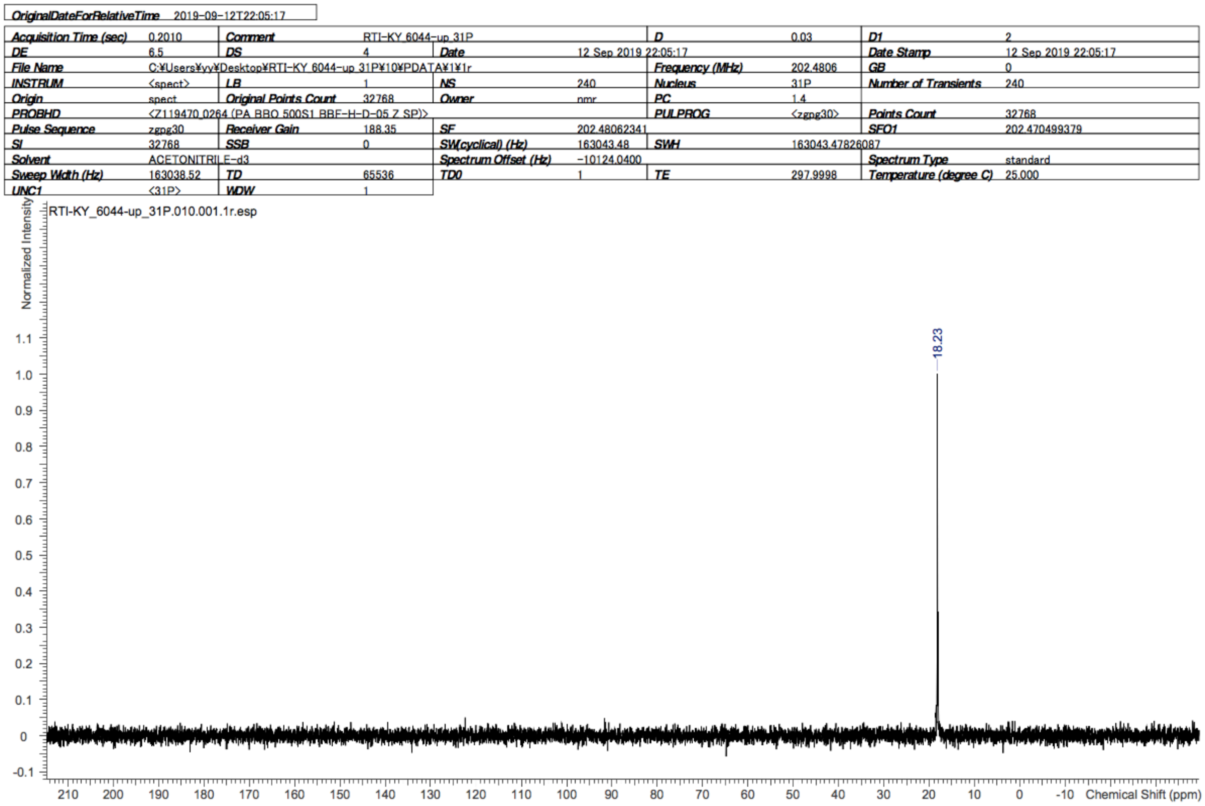

Compound **8c** (most polar isomer), ^{1}H-NMR (500 MHz, CD_3CN-d_3)

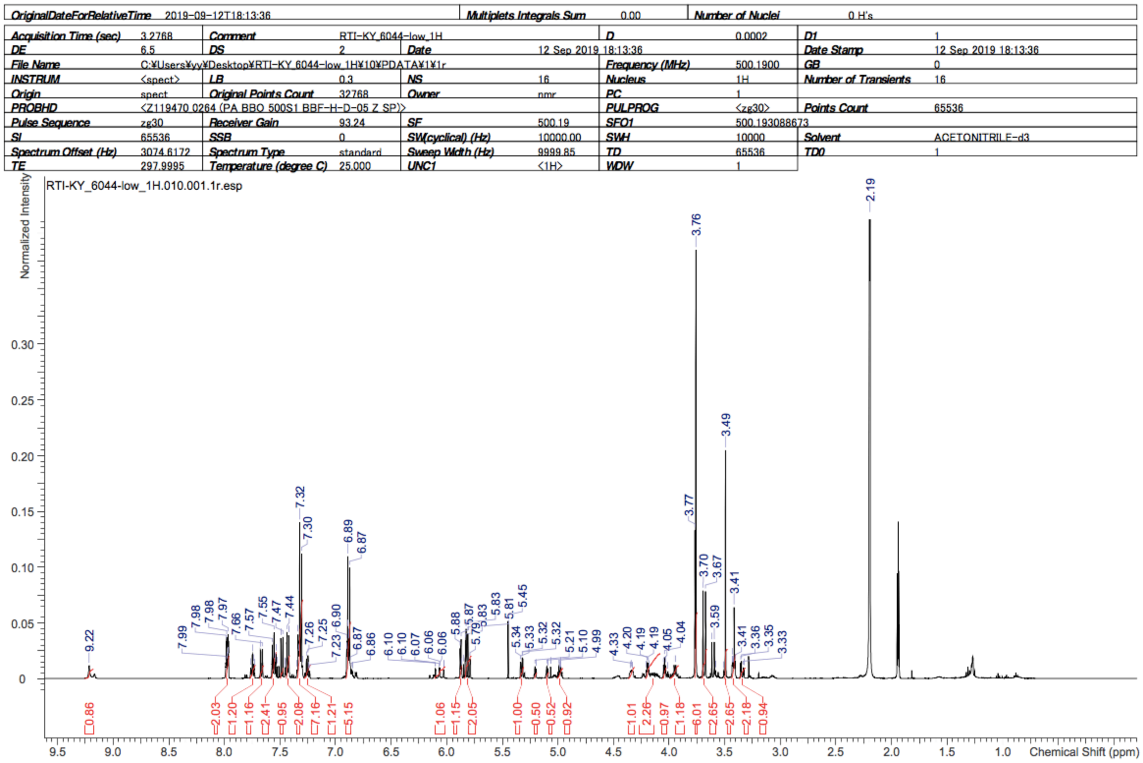

Compound **8c** (most polar isomer), ^{13}C-NMR (126 MHz, CD_3CN-d_3)

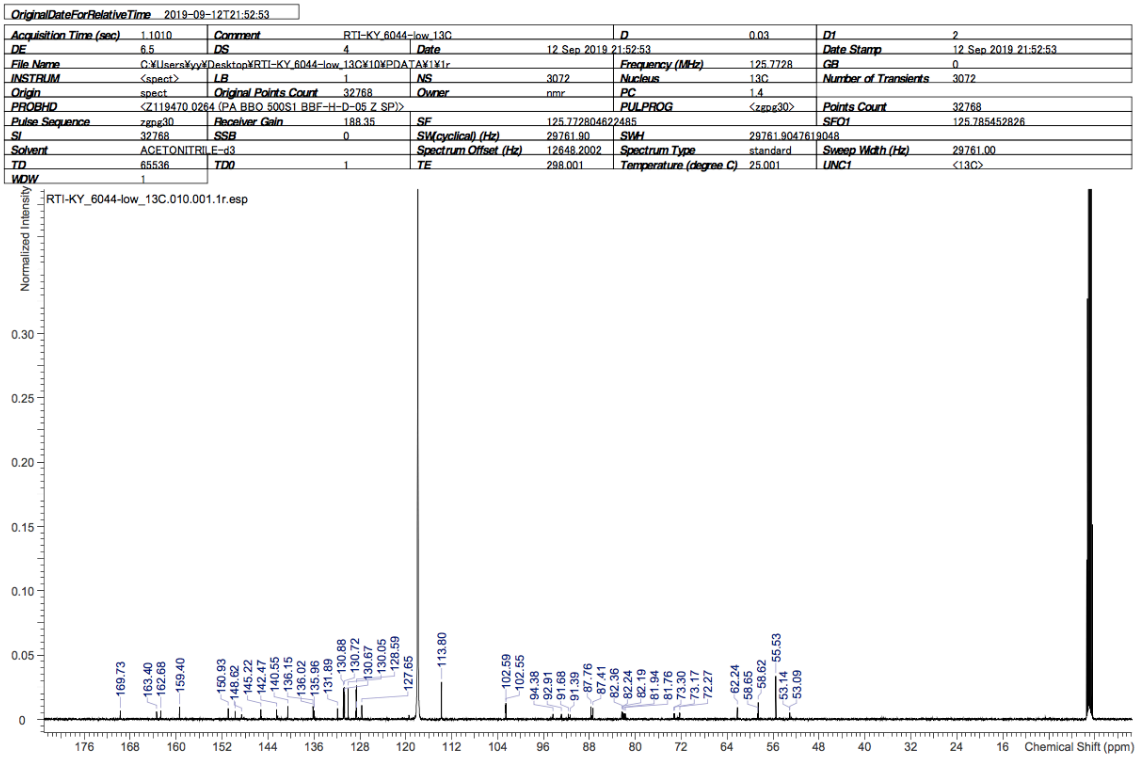

Compound **8c** (most polar isomer), ^{19}F-NMR (470 MHz, CD_3CN-d_3)

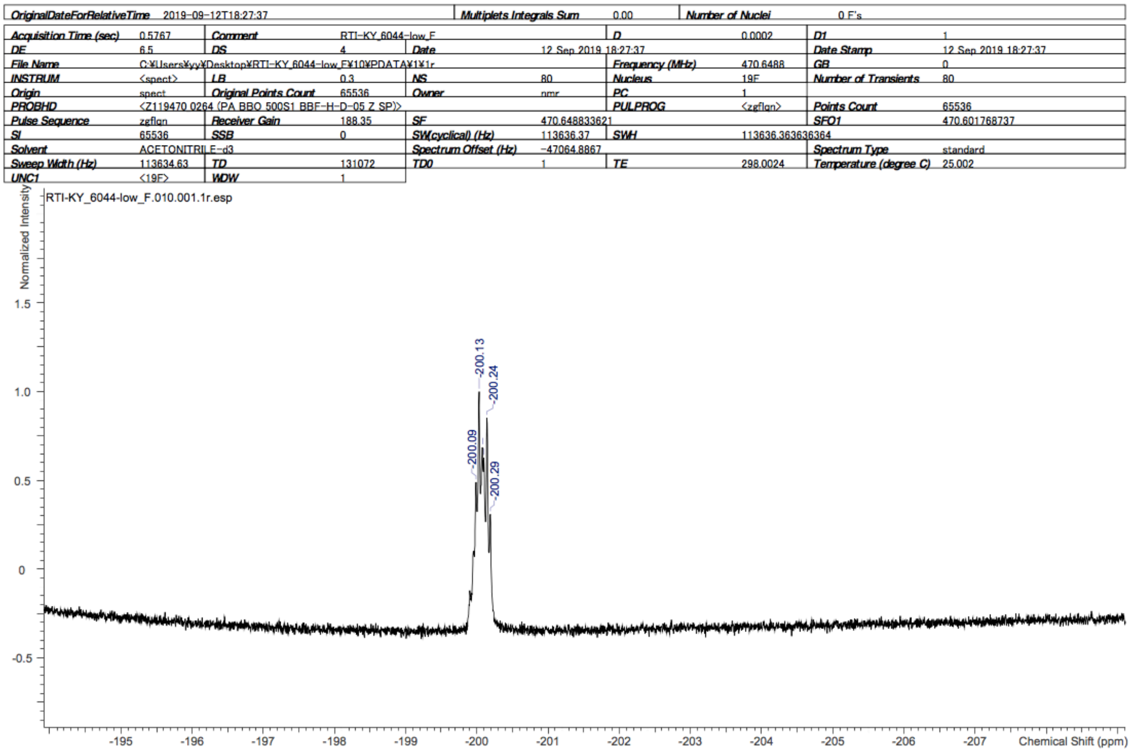

Compound **8c** (most polar isomer), ^{31}P-NMR (202 MHz, CD_3CN-d_3)

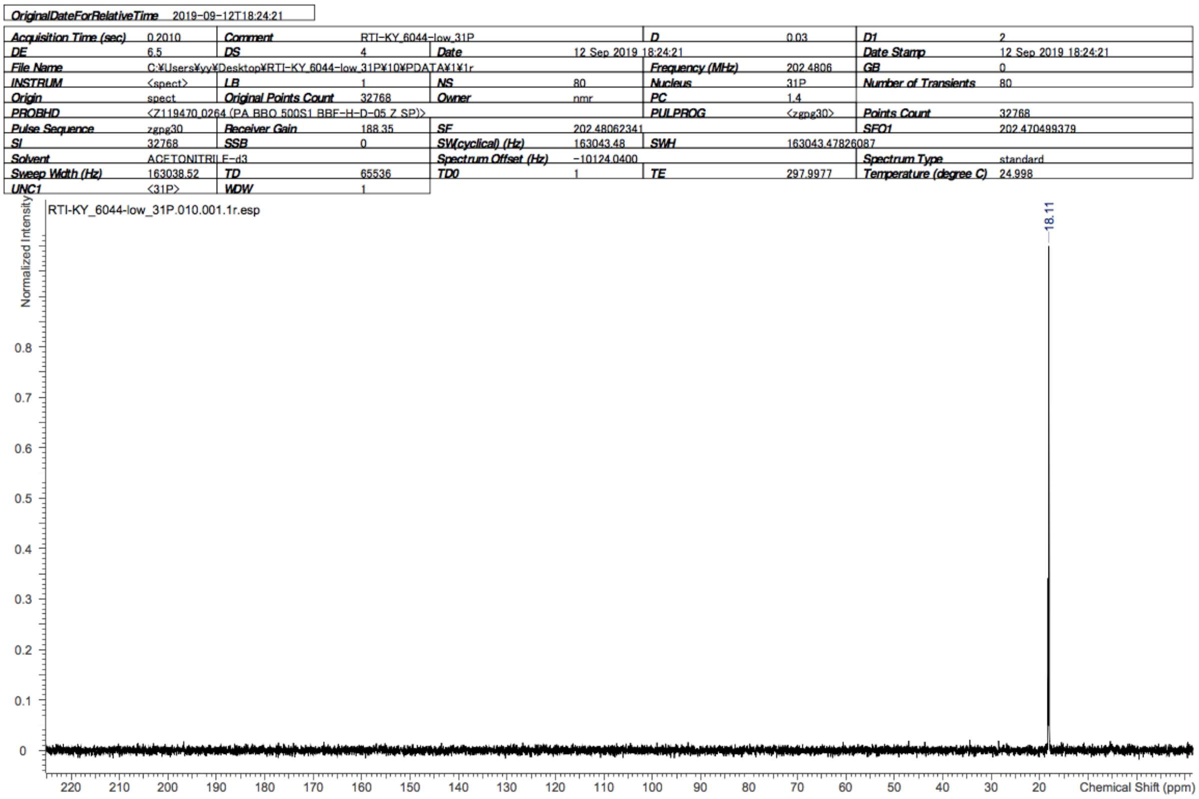

Compound **9c**, ^{31}P-NMR (202 MHz, CD_3CN-d_3)

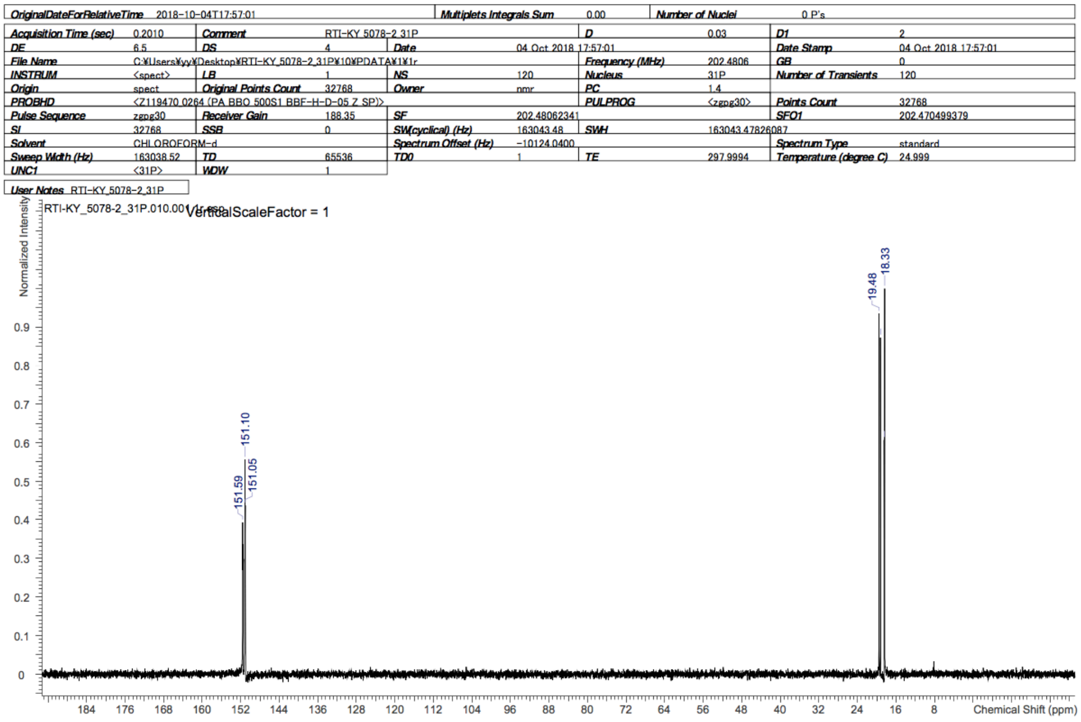

Compound **S2a**, ^{1}H-NMR (500 MHz, $CDCl_3$)

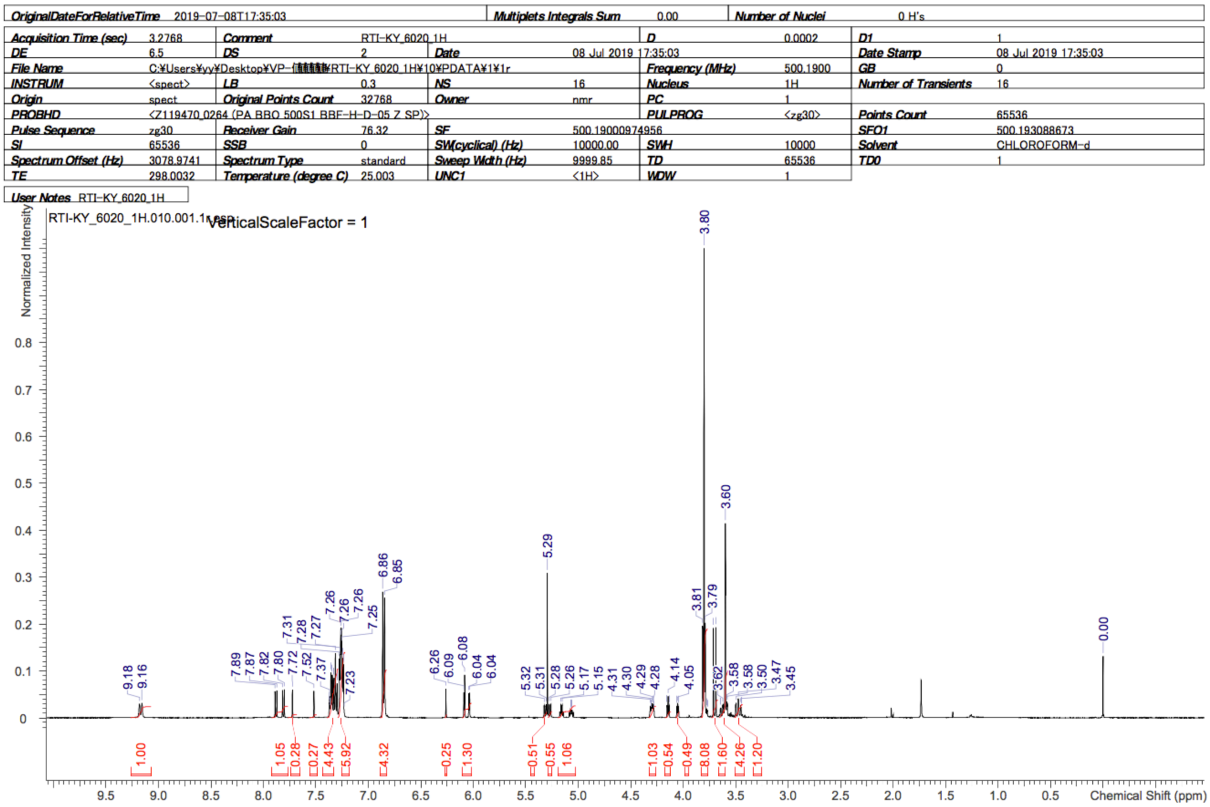

Compound **S2a**, ^{13}C-NMR (126 MHz, $CDCl_3$)

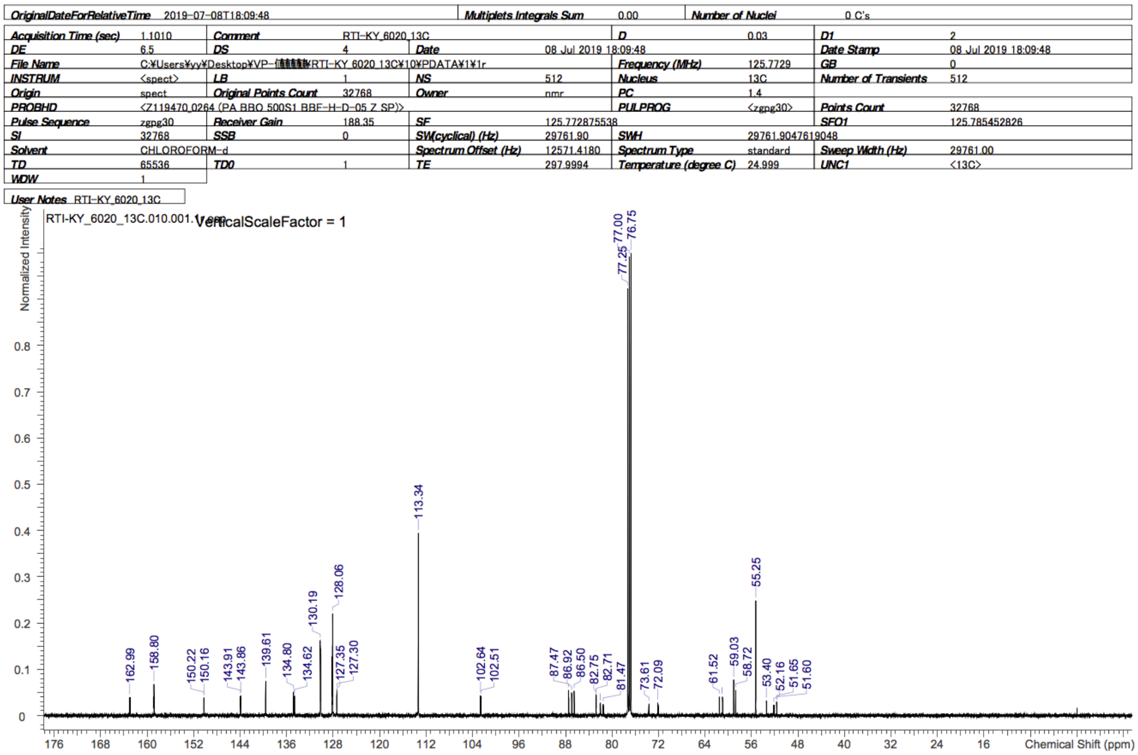

Compound **S2a**, ^{31}P-NMR (200 MHz, $CDCl_3$)

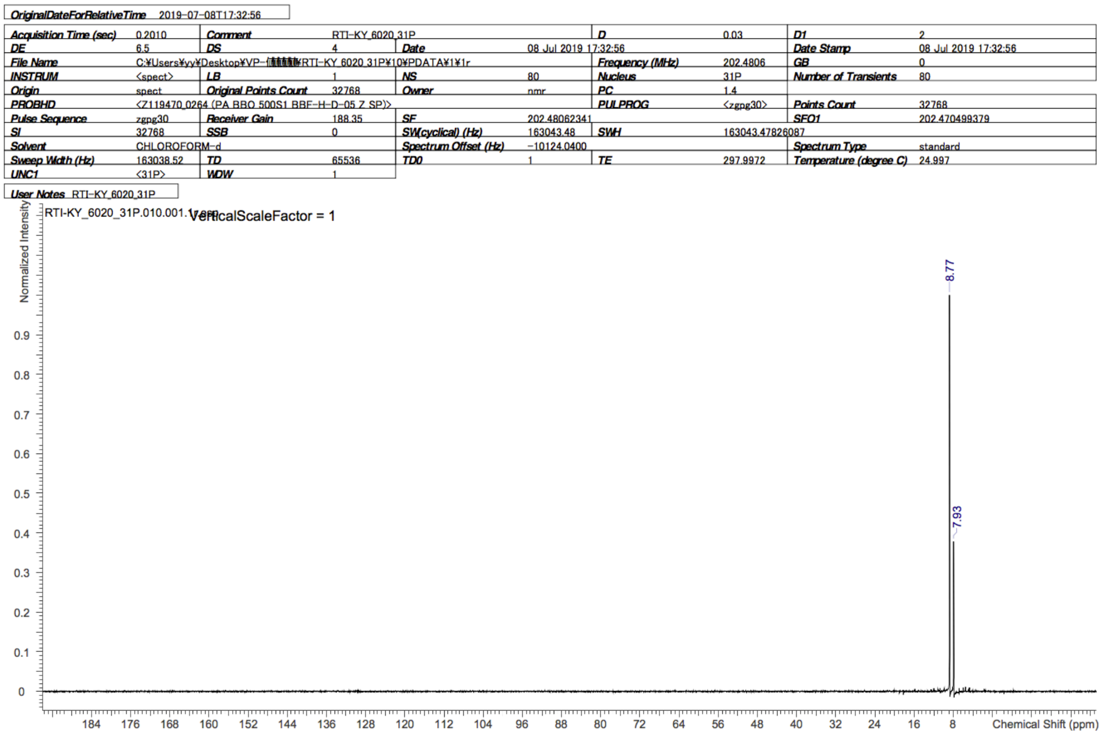

Compound **S1b**, ^{1}H-NMR (500 MHz, CD3CN-d_3)

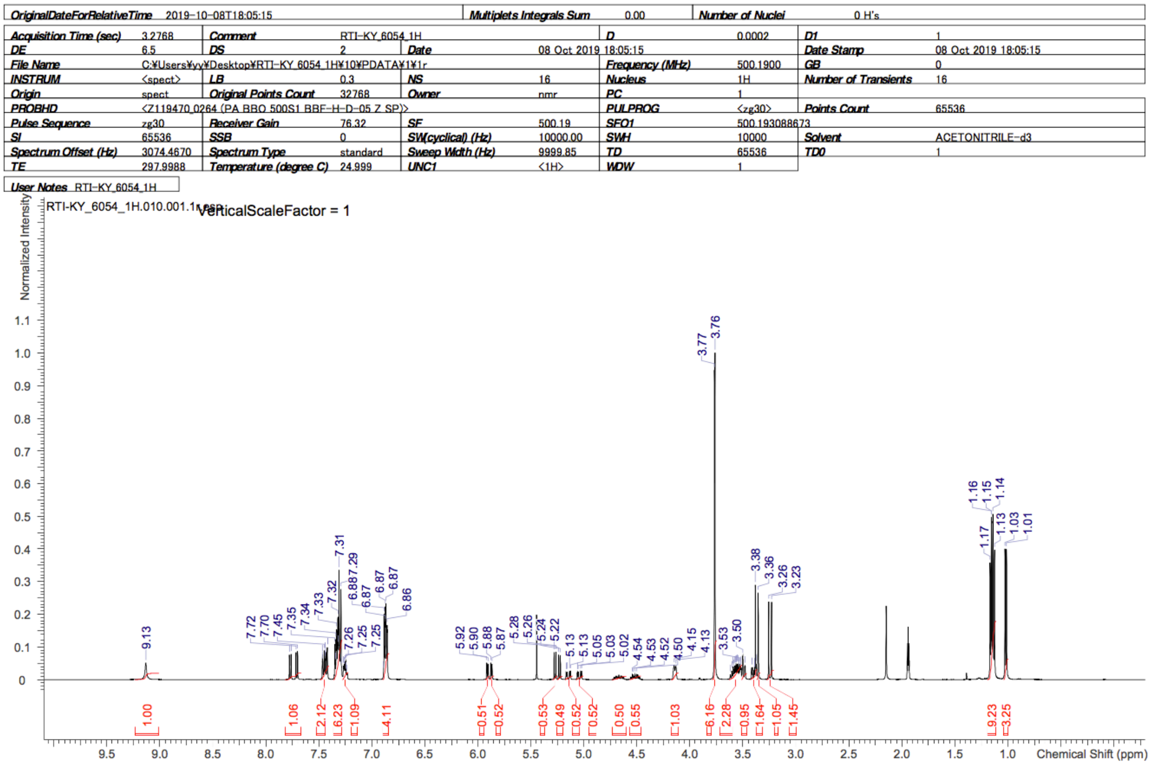

Compound **S1b**, ^{13}C-NMR (126 MHz, CD3CN-d_3)

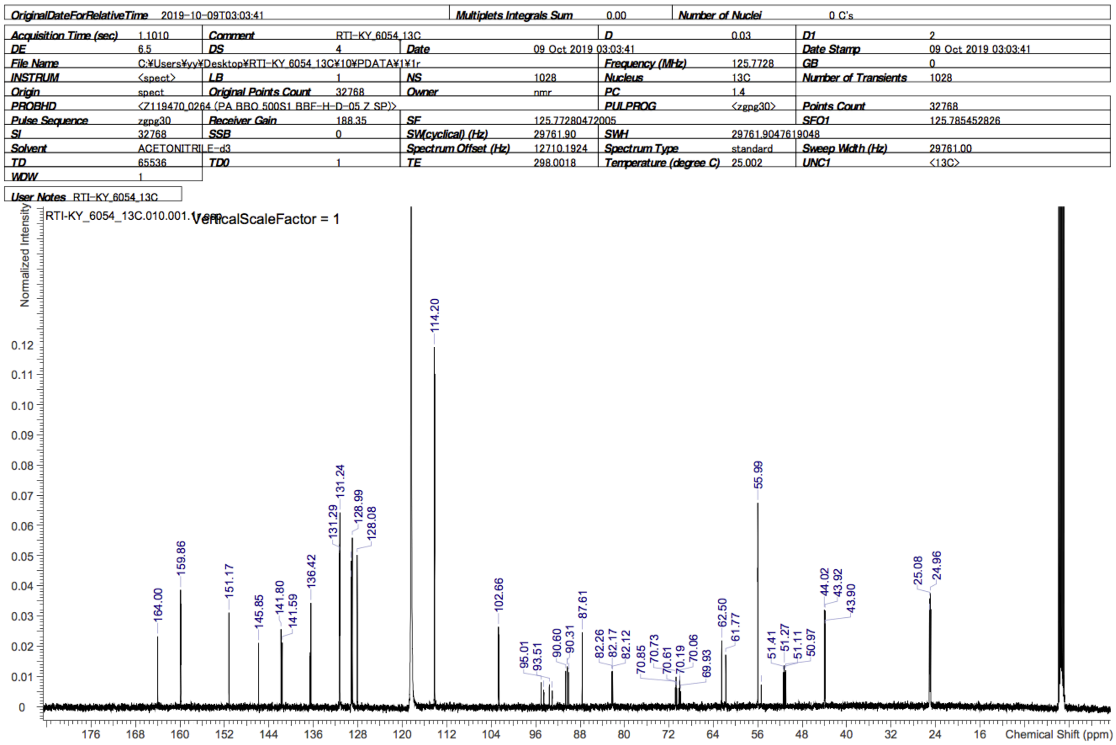

Compound **S1b**, ^{19}F-NMR (470 MHz, CD3CN-d_3)

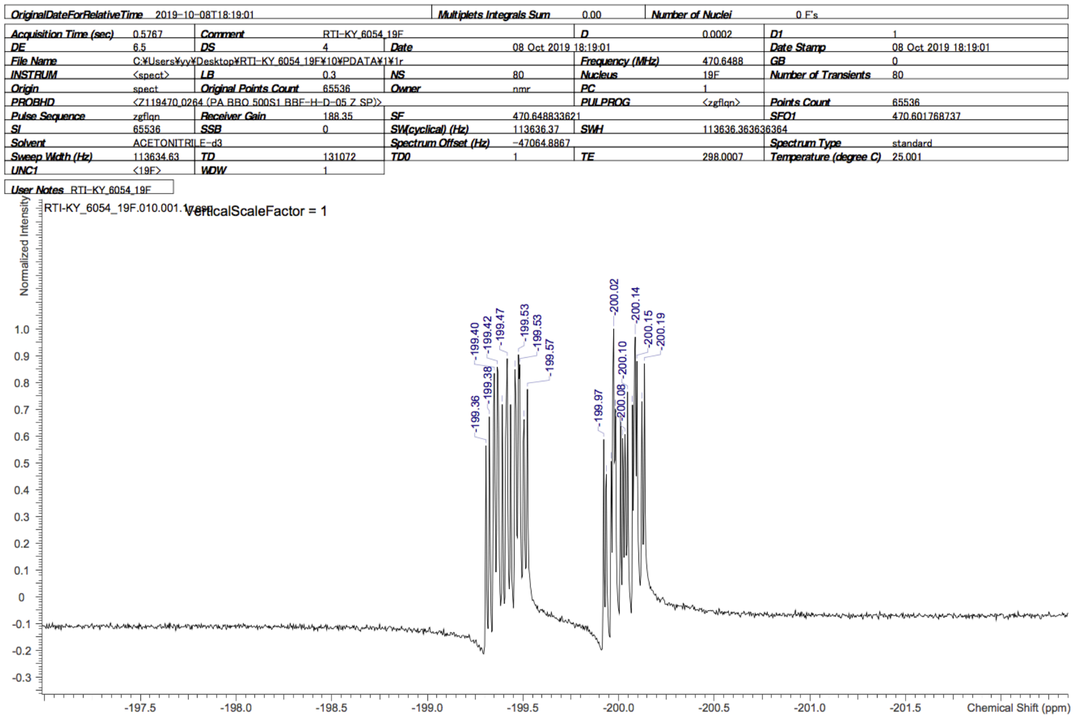

Compound **S1b**, ^{31}P-NMR (202 MHz, CD3CN-d_3)

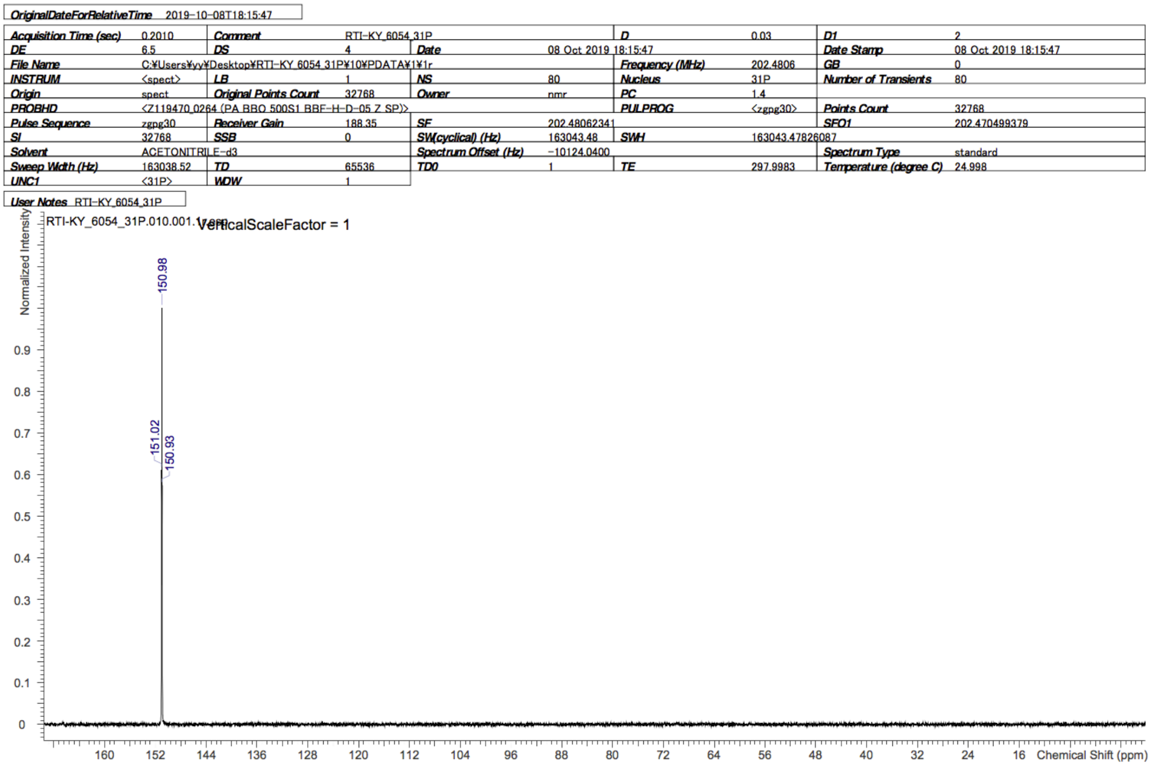

Compound **S2b**, ^{1}H-NMR (500 MHz, $CDCl_3$)

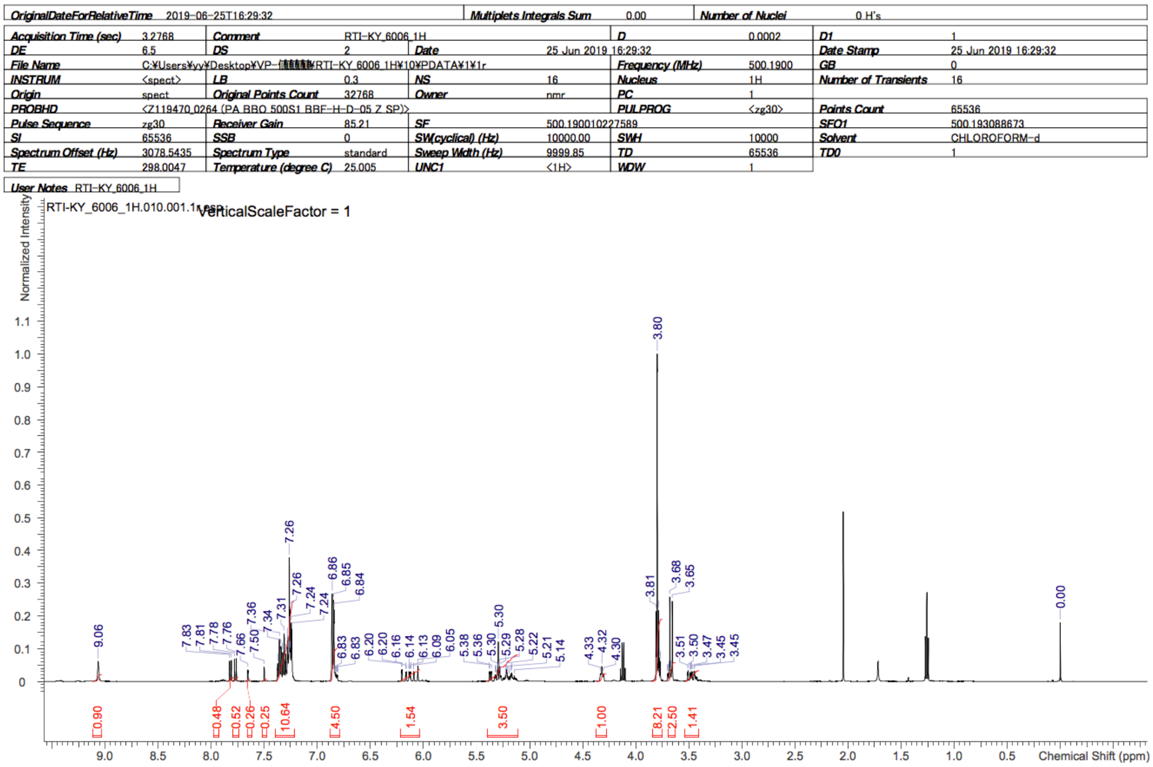

Compound **S2b**, ^{13}C-NMR (126 MHz, $CDCl_3$)

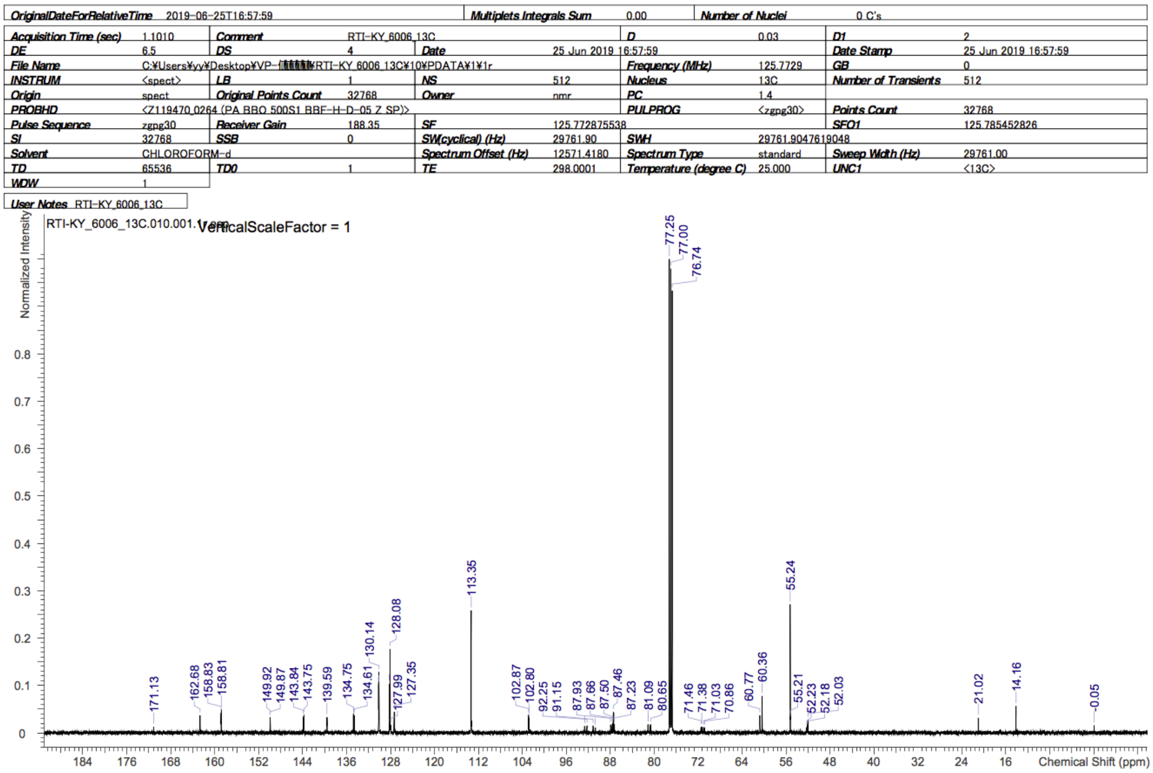

Compound **S2b**, ^{19}F-NMR (470 MHz, CD_3CN-d_3)

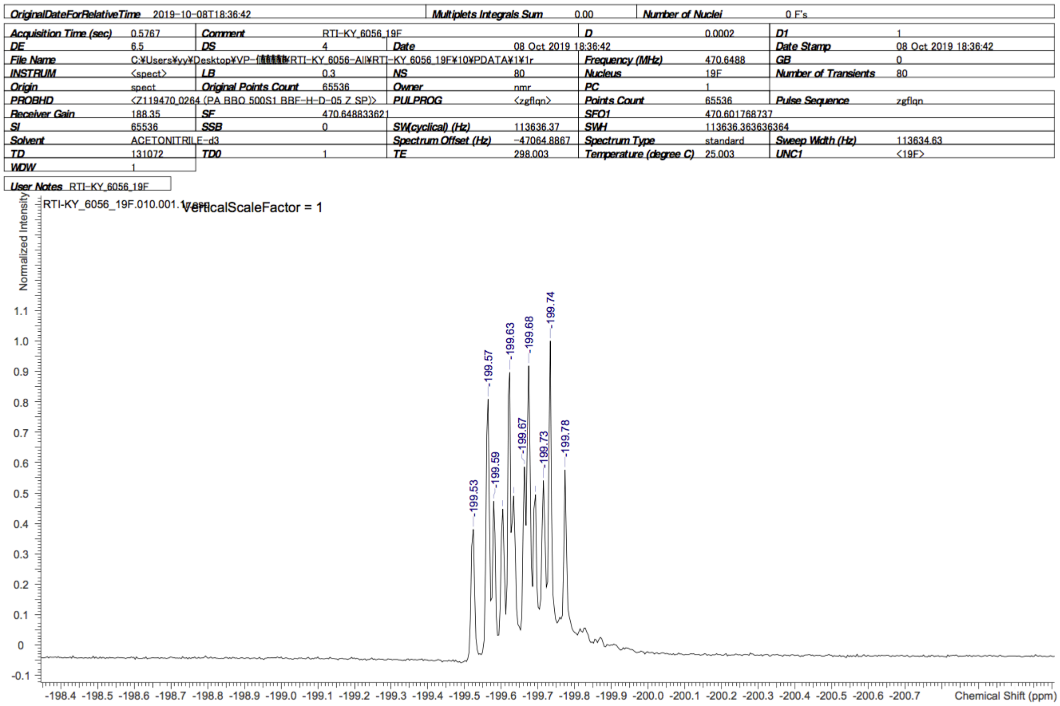

Compound **S2b**, ^{31}P-NMR (202 MHz, $CDCl_3$)

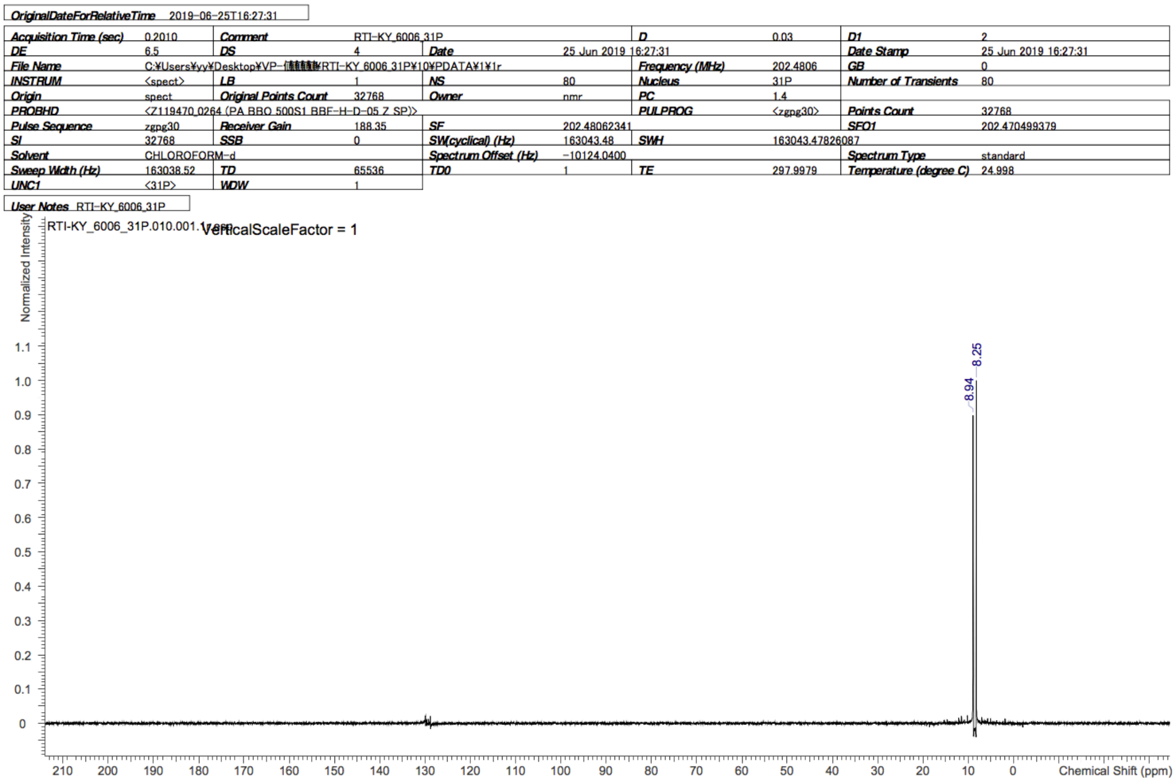

Compound **S1c**, ^{1}H-NMR (500 MHz, $CDCl_3$)

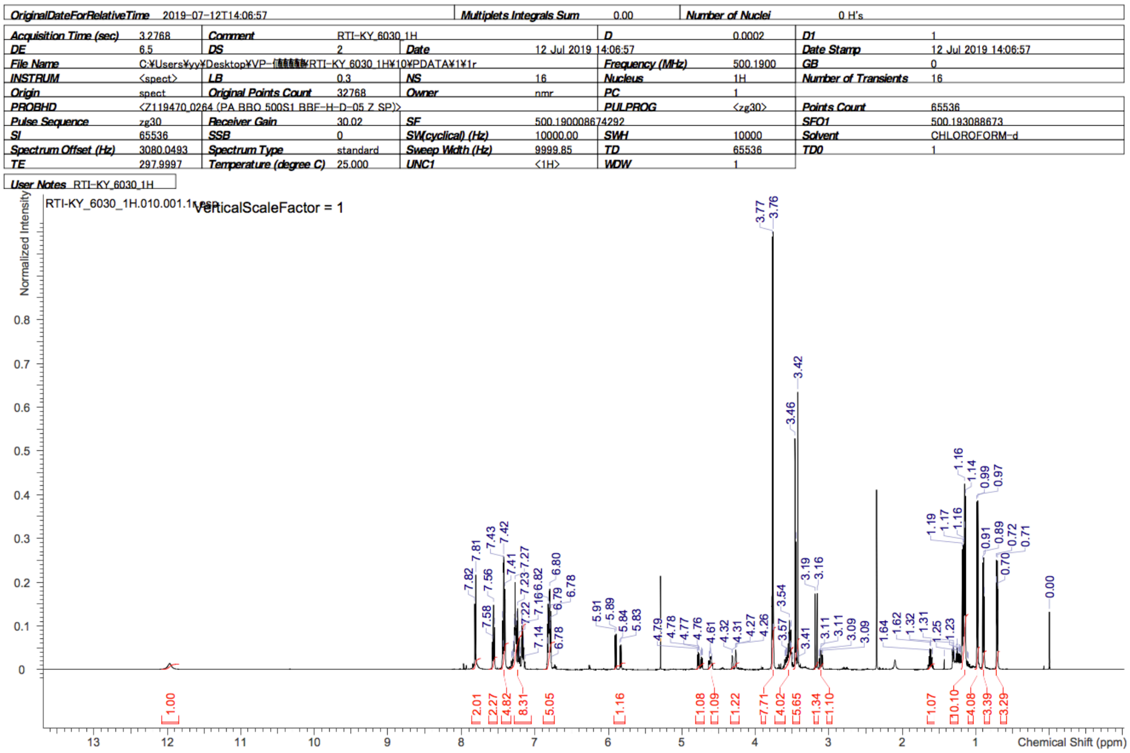

Compound **S1c**, ^{13}C-NMR (126 MHz, $CDCl_3$)

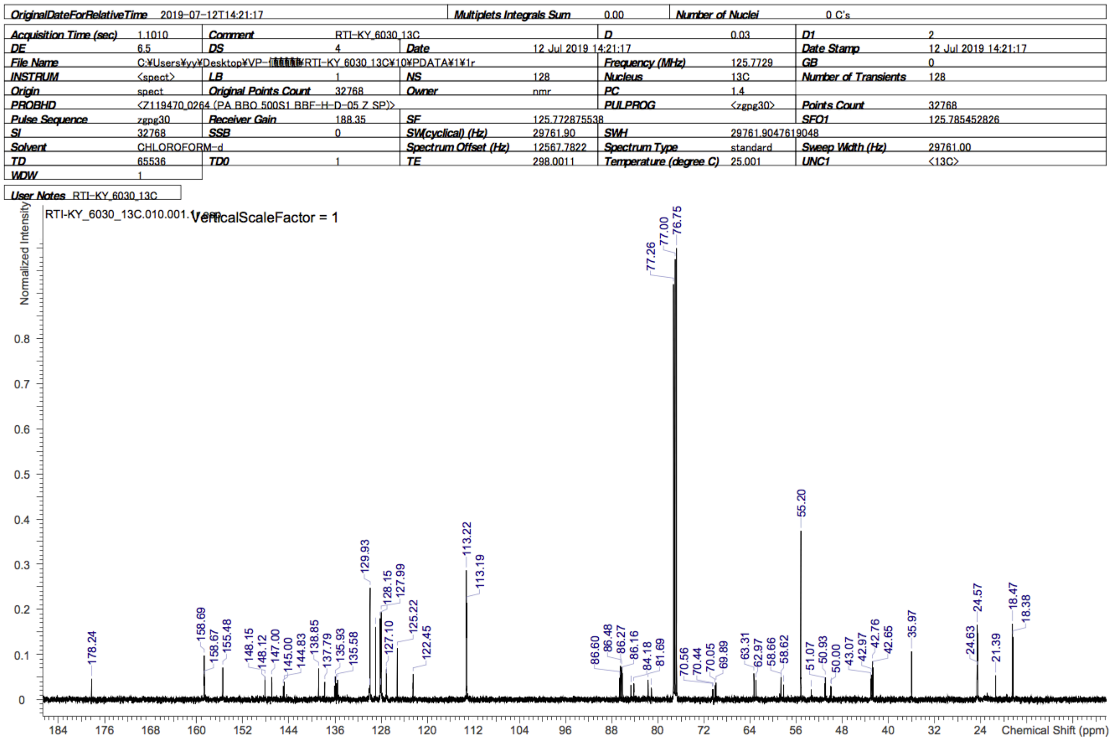

Compound **S1c**, ^{31}P-NMR (202 MHz, $CDCl_3$)

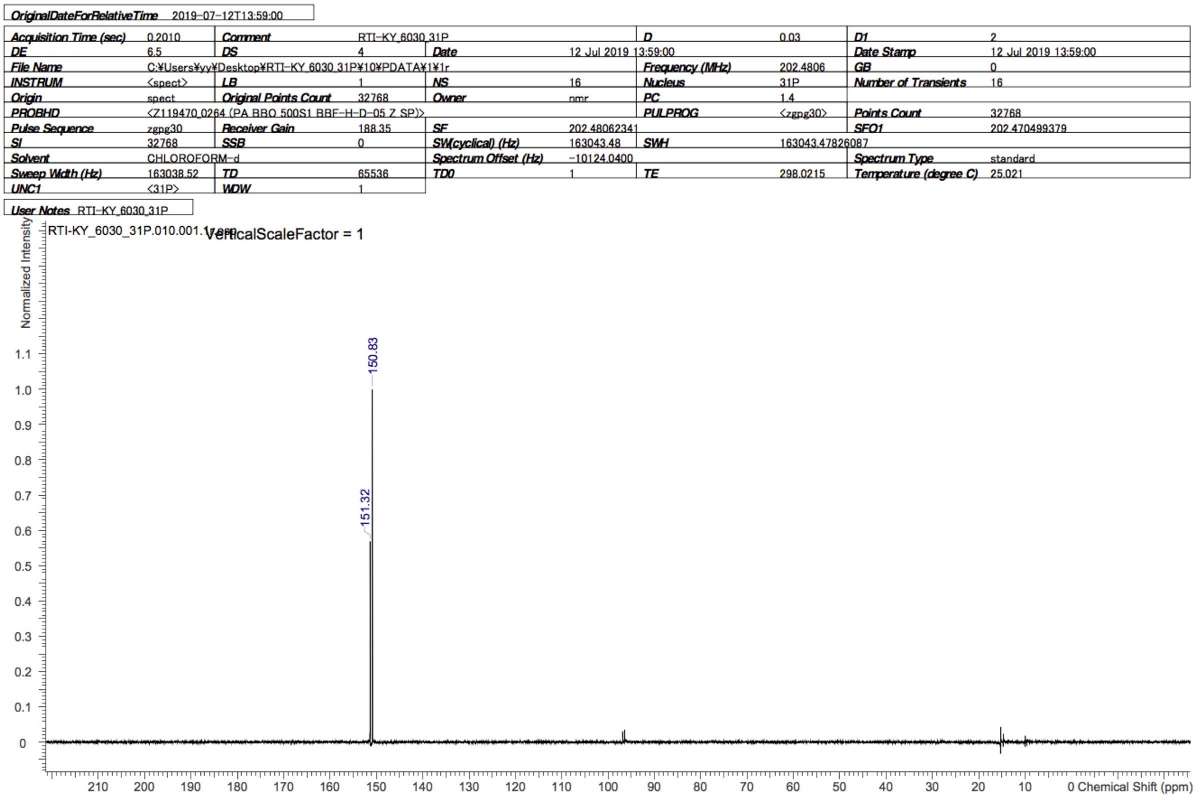

Compound **S2c**, ^{1}H-NMR (500 MHz, $CDCl_3$)

OriginalDateForRelativeTime	2019-07-12T21:31:27			*Multiplets Integrals Sum*	0.00	*Number of Nuclei*	0 H's
Acquisition Time (sec)	3.2768	*Comment*	RTI-KY_6032_1H	*D*	0.0002	*D1*	1
DE	6.5	*DS*	2	*Date*	12 Jul 2019 21:31:27	*Date Stamp*	12 Jul 2019 21:31:27
File Name	C:¥Users¥yy¥Desktop¥RTI-KY_6032_1H¥10¥PDATA¥1¥1r			*Frequency (MHz)*	500.1900	*GB*	0
INSTRUM	<spect>	*LB*	0.3	*NS*	16	*Nucleus*	1H
Number of Transients	16						
Origin	spect	*Original Points Count*	32768	*Owner*	nmr	*PC*	1
PROBHD	<Z119470_0264 (PA BBO 500S1 BBF-H-D-05 Z SP)>			*PULPROG*	<zg30>	*Points Count*	65536
Pulse Sequence	zg30	*Receiver Gain*	76.32	*SF*	500.190008215932	*SFO1*	500.193088673
SI	65536	*SSB*	0	*SW(cyclical) (Hz)*	10000.00	*SWH*	10000
Solvent	CHLOROFORM-d						
Spectrum Offset (Hz)	3080.5093	*Spectrum Type*	standard	*Sweep Width (Hz)*	9999.85	*TD*	65536
TD0	1						
TE	298.0068	*Temperature (degree C)*	25.007	*UNC1*	<1H>	*WDW*	1

User Notes RTI-KY_6032_1H

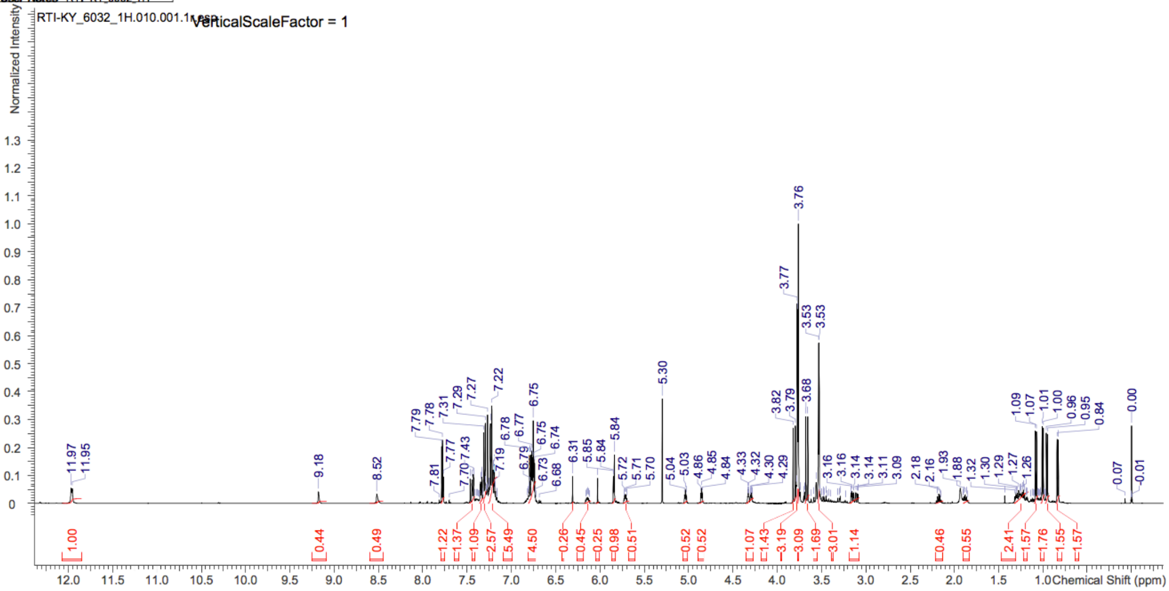

Compound **S2c**, ^{13}C-NMR (126 MHz, $CDCl_3$)

OriginalDateForRelativeTime	2019-07-12T22:06:00			*Multiplets Integrals Sum*	0.00	*Number of Nuclei*	0 C's
Acquisition Time (sec)	1.1010	*Comment*	RTI-KY_6032_13C	*D*	0.03	*D1*	2
DE	6.5	*DS*	4	*Date*	12 Jul 2019 22:06:00	*Date Stamp*	12 Jul 2019 22:06:00
File Name	C:¥Users¥yy¥Desktop¥RTI-KY_6032_13C¥10¥PDATA¥1¥1r			*Frequency (MHz)*	125.7729	*GB*	0
INSTRUM	<spect>	*LB*	1	*NS*	512	*Nucleus*	13C
Number of Transients	512						
Origin	spect	*Original Points Count*	32768	*Owner*	nmr	*PC*	1.4
PROBHD	<Z119470_0264 (PA BBO 500S1 BBF-H-D-05 Z SP)>			*PULPROG*	<zgpg30>	*Points Count*	32768
Pulse Sequence	zgpg30	*Receiver Gain*	188.35	*SF*	125.772875538	*SFO1*	125.785452826
SI	32768	*SSB*	0	*SW(cyclical) (Hz)*	29761.90	*SWH*	29761.9047619048
Solvent	CHLOROFORM-d			*Spectrum Offset (Hz)*	12571.4180	*Spectrum Type*	standard
Sweep Width (Hz)	29761.00						
TD	65536	*TD0*	1	*TE*	297.9993	*Temperature (degree C)*	24.999
UNC1	<13C>						
WDW	1						

User Notes RTI-KY_6032_13C

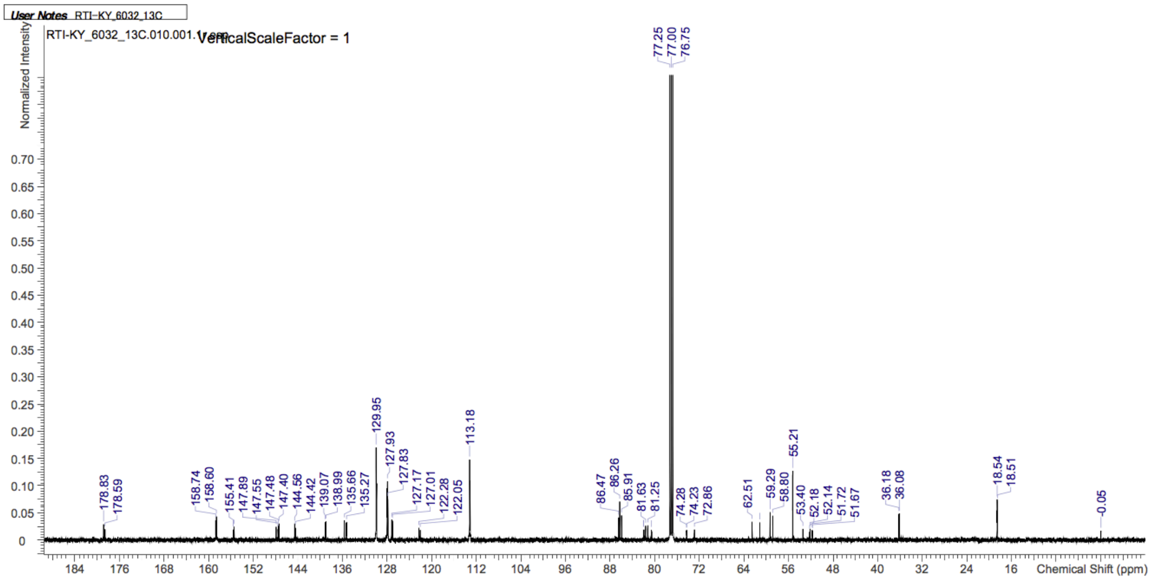

Compound **S2c**, ^{31}P-NMR (202 MHz, $CDCl_3$)

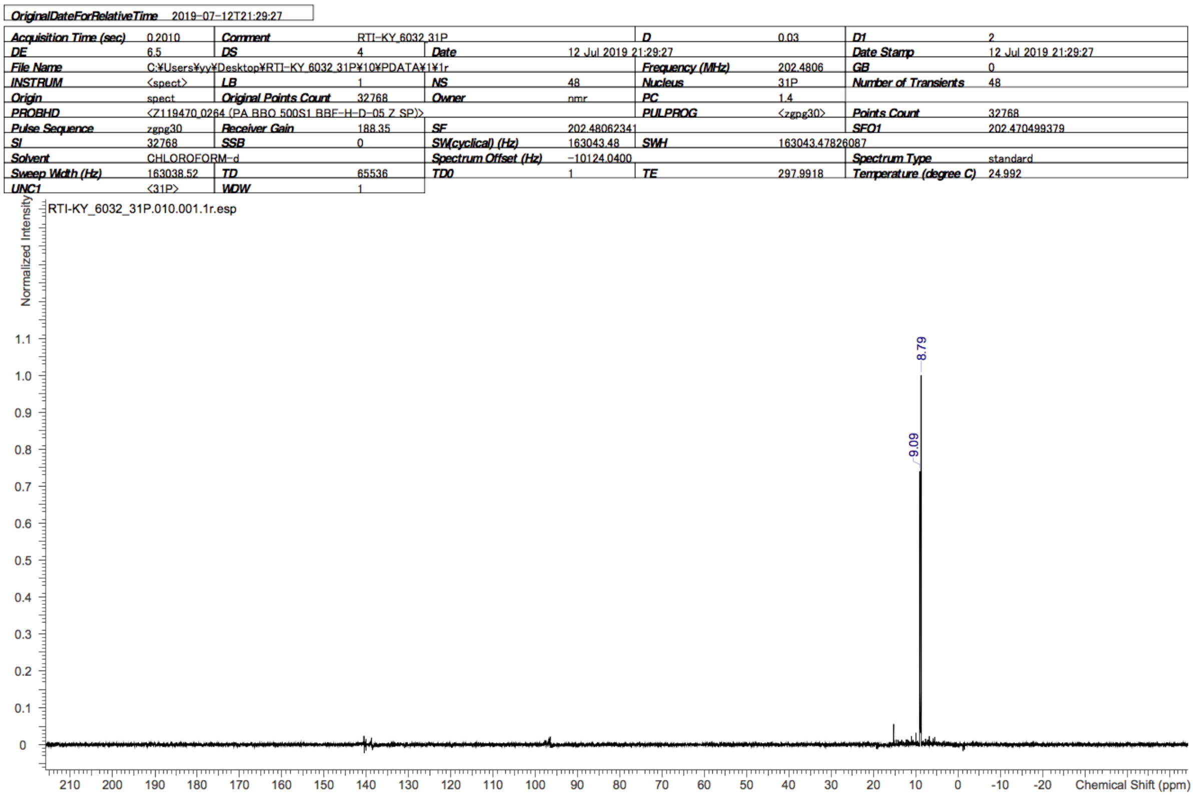

Supplement: gkab1126_Supplemental_File [file gkab1126_supplemental_file.pdf]
